# On-the-move heterogeneous face recognition in frequency and spatial domain using sparse representation

**DOI:** 10.1371/journal.pone.0308566

**Published:** 2024-10-04

**Authors:** Asif Raza Butt, Sajjad Manzoor, Asim Baig, Abid Imran, Ihsan Ullah, Wasif Syed Muhammad

**Affiliations:** 1 Department of Electrical Engineering, Mirpur University of Science and Technology, Mirpur, AJK, Pakistan; 2 Research Institute of Engineering and Technology, Hanyang University (ERICA), Ansan, South Korea; 3 Curious Thing AI, Sydney, New South Wales, Australia; 4 Department of Mechanical Engineering, Ghulam Ishaq Khan Institute of Engineering Sciences and Technology (GIKI), Swabi, KPK, Pakistan; 5 Department of Electrical Engineering, Comsats University Islamabad, Abbottabad Campus, Abbottabad, KPK, Pakistan; 6 Department of Electrical Engineering, University of Gujrat (UoG), Gujrat, Pakistan; GIET University, INDIA

## Abstract

Heterogeneity of a probe image is one of the most complex challenges faced by researchers and implementers of current surveillance systems. This is due to existence of multiple cameras working in different spectral ranges in a single surveillance setup. This paper proposes two different approaches including spatial sparse representations (SSR) and frequency sparse representation (FSR) to recognize on-the-move heterogeneous face images with database of single sample per person (SSPP). SCface database, with five visual and two Infrared (IR) cameras, is taken as a benchmark for experiments, which is further confirmed using CASIA NIR-VIS 2.0 face database with 17580 visual and IR images. Similarity, comparison is performed for different scenarios such as, variation of distances from a camera and variation in sizes of face images and various visual and infrared (IR) modalities. Least square minimization based approach for finding the solution is used to match face images as it makes the recognition process simpler. A side by side comparison of both the proposed approaches with the state-of-the-art, classical, principal component analysis (PCA), kernel fisher analysis (KFA) and coupled kernel embedding (CKE) methods, along with modern low-rank preserving projection via graph regularized reconstruction (LRPP-GRR) method, is also presented. Experimental results suggest that the proposed approaches achieve superior performance.

## Introduction

Real time heterogeneous facial recognition systems have become an important research topic of contact-less and non-intrusive technique for personal identification [1]. The face images can be taken by different sources such as sketches, infrared (IR), thermal and visual imagery. These images are commonly termed as heterogeneous face images [2]. Thermal cameras are suitable to capture events that occur in dark or severe environmental conditions. On the other hand, in order to acquire a 24 hours day and night processing, IR cameras can be more effective. The substantial intra-class variation among heterogeneous face photos and the scarcity of training sets of cross-modality face image pairs make heterogeneous face recognition problems more complex than those of common face recognition [3]. The substantial intra-class variation among heterogeneous face photos and the scarcity of training sets of cross-modality face image pairs make heterogeneous face recognition problems more complex than those of common face recognition.

In [4], the first attempt in the NIR-VIS area to develop a probability distribution learning for VIS-NIR matching approach is proposed, namely Wasserstein convolution neural networks to learn invariant features between near-infrared and visual face images. To tackle the NIR-VIS matching problem, purify identification information and de-tangle within-class variation information, [5] offers a unique method called dual adversarial disentanglement (DAD) and deep representation de-correlation (DRD). In [6], an innovative dual face alignment learning (DFAL) technique is developed for examining potential neutral face representations of cross-domain data that are unrelated to modality that suggest learning neutral face representations for NIR and VIS pictures is efficient at minimizing domain and residual variations. According to the research [7], the cross-modality matching problem can be effectively solved by boosting identity-discriminative feature learning in the feature space while suppressing modality-related components in the metric space.

The aim of a real world heterogeneous face recognition systems, that covers a large population, is to attain high accuracy in identification. In the real world, face images are acquired not only in presence of noise such as illumination variation, posing, aging, facial expression and in low resolution but also during night time. On the other side, the gallery images are generally of high resolution and acquired under controlled conditions. This contrast in quality of probe and gallery images makes it difficult to achieve high identification accuracy.

Currently the major challenges in recognizing face images in real time scenarios, that include: (i) higher image resolution in contrast to available low image resolutions, (ii) in the real world scenarios, often the surveillance cameras capture face images while the subjects are on move, causing pose, illumination and blurring effects, (iii) the inherent high dimensionality of face images is another challenge which makes face recognition a challenging task. In recent times sparse representation based approaches [8] have gained popularity particularly due to their ability to cop-up challenges, including higher dimensionality, poor image quality, and recognizing face images in unconstrained environments. In the current work we will use sparse transform based approach to recognize face images with multiple challenges including different image modalities and varying image resolutions.

### Related work

Traditionally, face recognition based research has focused mainly on changes across illumination and pose [9–12], however in recent years growing interest have been seen in handling poor resolution [13–15]. Multiple strategies have been tried to tackle the modality mismatch between the low quality probe images (acquired from the CCTV camera) and the high quality database image. One of the earliest attempts was based on developing a 3D model of the face from the database images and the model was then fit to the input probe image by changing the orientation and illumination of the model [16]. The problem with these types of techniques is that their high computational cost makes them unsuitable for real time applications. Another approach being tried by researchers is to develop a generative model that is able to separate illumination and texture data within the image. The texture of the images in the database is then down sampled to match the low quality input images [17, 18]. More recent research has been focused on the development of learning based approaches to measure likelihood of similarity between the database and probe imagery [19, 20]. In [21], Unified Face Image (UFI) approach based on samples from multiple surveillance cameras has been suggested to recognize face images. In [22], a composite-face based on images acquired from multiple frames is presented.

For the real time Face Recognition Systems (FRS), the main challenge is quality of face image [23]. A lot of approaches have been used to cop-up such challenges. In real time FRS, it is possible to select the multiple views of the face images and then select the good quality images which results into increased processing time and computational cost. The initial work in this scenario suggested robust Principal Component Analysis (PCA) by eliminating the low quality images which they consider to be an overhead [24]. However, this approach can be applied in real time FRS where image quality is low. Universal quality index is proposed in [25, 26] by taking a reference image and comparing it with the image distorted by illumination. Another approach [27] suggests a reference image and defines a patch base model. Another method suggested for pose estimation by using tree structure in order to access the image quality [28]. The effect of illumination and pose variations on image quality is important in term of performance which is analyses by Mahmood et al. [29] using Adaptive-boosting (AdaBoost) with linear discriminant analysis as weak learner, the PCA-based approach, and the Local Binary Pattern (LBP)-based approach. Although such approaches have achieved state-of-the-art performance, yet limited by use of reference images. A recent face recognition approach presented in [30] performs face image quality assessment by using Ranked Quality Scores (RQS). They used standard Surveillance Cameras face (SCface) database and provided the results for only good quality images, thus the approach is limited to good quality image only instead of entire database.

In order to recognize infrared (IR) face images, researchers focused on the use of well-known Eigen Faces [31–33]. Wavelets based feature extraction scheme for state recognition in children with autism has been proposed in [34], by using thermal images. Some researchers used Principal Components Analysis (PCA), Linear Discriminant Analysis (LDA) and their non-linear variants Kernel Principle Component Analysis (KPCA) and Kernel Fisher Analysis (KFA) for recognition of IR face images [35–37]. Additionally, PCA [38], LDA [39], independent component analysis (ICA) [40] based approaches have been suggested for heterogeneous face recognition. A discrete cosine transform (DCT) [41] and support vector machines (SVM) [42] have also been used for recognition of near IR face images. These methods fulfill the purpose however, work on low quality images with IR and various visual heterogeneous sill need to be done.

Researchers in the domains of signal processing, image processing, computer vision, and pattern recognition have given sparse representation a lot of attention. Sparse representation has developed into a core tool that is integrated into many learning systems and has seen significant advancements and previously unheard-of successes [43–45]. Both graph and projection learning techniques to get a combined optimization framework are used in [46] to develop a Low-Rank Preserving Projection Via Graph Regularized Reconstruction (LRPP-GRR) method for face recognition. Recently, the field of face recognition systems have witnessed significant advancements in the use of convolution neural network (CNN) [47, 48] and deep learning (DL)-based techniques [49–51] however such methods need large training data which is impossible for case where a single sample per person (SSPP) is taken into consideration. Sparse representation classifier (SRC) techniques can result in a good trade-off between verification accuracy and security, and the study [52] indicates that the SRC methodologies utilized in [53–55] can achieve high accuracy while simultaneously avoiding some inadequacies results in multiple biometrics.

In this study we propose two sparse representation based techniques to recognize SSPP) heterogeneous face images. These techniques include (i) Spatial Sparse Representation (SSR) and (ii) Frequency Sparse Representation (FSR). In SSR, face images are analyzed in spatial domain, while in FSR, the face images are transformed to frequency domain using Fast Fourier Transform (FFT). Keeping in view the existing approaches and to the best of our knowledge, this study is the first of its kind to consider heterogeneous face recognition using sparse representation. To evaluate the performance of sparse representation based approach an enrollment database with high quality IR and visual images and probe gallery with low quality IR and visual facial images is used. The matching performance is evaluated for Visual-to-Visual matching, IR-to-IR matching and Visual-to-IR matching to create heterogeneity among the images. This detailed evaluation provides an interesting insight into how the sparse representation based approach views the data and what is the ideal format to use with such type of approaches. Experimental results on benchmark databases, the SCface [56] and the CASIA NIR-VIS 2.0 [36], suggests the superior performance of opted method compared to some existing stat-of-the art classical approaches.

This paper is further organized as follows; in "Material and Methods" Section, the proposed sparse representation base face recognition methodology including feature extraction and various experimental scenarios is discussed. This section also gives face image databases and pre-processing system is also described here. In "Result" section, the results related to proposed algorithm using SCface and CASIA NIR-VIS 2.0 databases, with various scenarios is given. Finally in "Conclusion" section, the study is concluded with discussion. The future work is also proposed in this section.

## Materials and methods

In this research a sparse representation base algorithm is proposed for on-the-move heterogeneous face recognition. Sparse representation-based image matching has emerged as a effective technique in the field of image processing. This approach can represent, an image, as a linear combination of a relatively small fractions, taking into consideration that many natural images shows sparsity when suitably represented. One of the main feature of sparse representation-based face recognition is its inherent robustness to noise being uniformly spread across the data. As a result it mainly focuses on preserving important features of an image while attenuating noise.

### Proposed face recognition method

**Fig 1:**
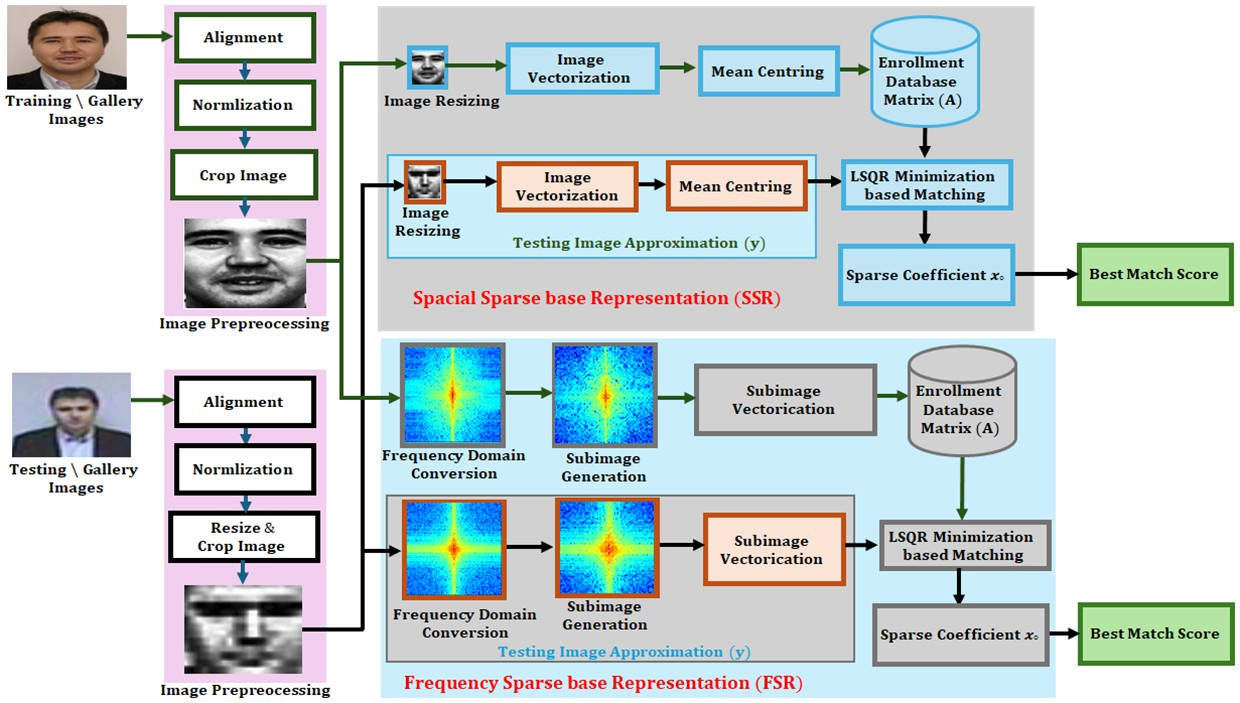
Architecture of proposed methodology for face recognition using FSR and SSR.

The proposed algorithm for on-the-move heterogeneous face recognition is given in Fig 1, where the individual in this manuscript has given written informed consent for publication. The training and testing images are initially preprocessed. Then using frequency and special domain conversion the face is recognized with FSR and SSR filters. In this research different scenarios are used in experiments, which are carried out utilizing the benchmark databases SCface and CASIA NIR-VIS 2.0. The proposed method is also compared with other state of the art methods for accuracy.

#### Image pre-processing

A number of unwanted variations are present in training (gallery) and testing (probe) face images. In order to mitigate the effects of such variations, following face normalization steps are is proposed;

1. Align the face images, vertically upright, based upon eye coordinates, extracted from the databases used in the research i.e. Scface ([56] and CASIA NIR-VIS 2.0 [36]). For this purpose the images are rotated in x-y plane. The face alignment is performed, such that inter-pupillary distance is same for all face images.
2. In order to remove color cast, convert all the face images from RGB to gray scale.
3. Use Histogram Equalization to normalize unwanted light variations.
4. Finally, all the face images are cropped and resized to 530x530 pixels.

#### Sparse representation based face recognition algorithm

Sparse representation based approaches works on the generalization of the nearest-subspace (NS) [8] approaches. Nearest-Subspace based classifiers are trained on samples which are best linearly represented for each class. The major difference between the two approaches is that NS approaches use the training samples as the face subspace whereas sparse representation based approaches use the complete enrollment dataset as training images for classification. This makes sparse representation based approaches more robust against illumination and pose variations. Although, smaller variations between faces of difference users can cause misclassification in this representation, however by representing images sparsely, the dimensionality of the data is effectively reduced, leading to computational efficiency in image matching processes. SSR and FSR are two types of sparse algorithms will be used two analyses face recognition in spacial and frequency domain.

*Spacial Sparse base Representation (SSR)*. In spacial domain sparse representation the images are represented directly into their original domain, i.e. to the pixel values of images without transforming to other domain, such as frequency or wavelet domains. Considering a training set *I* with *C* being the number of classes and *n* number of training image in each class, then sparse representation as a rule works such that for *i*^*th*^ class the sample set *I*_*i*_ can be given as follows [8];

$$\begin{array}{c} I_{i}=\left[V_{i,1},V_{i{,}_{2}},...,V_{i{,}_{n}}\right]\;\;\in R_{i}^{M\times n} \end{array}$$ (1)

where *V*_*i*,*n*_ is a vectorized (column vector) training image of the *i*^*th*^ class with *n* representing number of images in the database and M is the dimensionality of the training image. The vectorization of the *n*^*th*^ training image is achieved after its resizing through bilinear interpolation [57]. Each vectorized column vector is then mean centered to remove any bias in their pixel values.

For a real scenario the membership of the new image is unknown and to handle that, with *N* = *n* × *C* a new matrix A is defined that is formed by stacking the mean-centered vectorized images, and encompasses the entire enrollment database horizontally, and can be represented as;

$$\begin{array}{c} A=[I_{1},I_{2},\ldots \ldots \ldots \ldots \ldots \ldots .,I_{C}]\;\;\in R_{i}^{M\times N} \end{array}$$ (2)

The resizing, vectorization and mean centering is also performed for any new input test image *y* ∈ *R*^*n*^ of the same class. It will lie almost on the same linear subspace as that of *i*^*th*^ class and can be represented as;

$$\begin{array}{c} y=\alpha_{i,1}V_{i,1}+\alpha_{i,1}V_{i,2}+...+\alpha_{i,n}V_{i,n} \end{array}$$ (3)

where y is the approximation of the new input image, based on the existing training images and *α* is the coding coefficient. It can be seen that the more training examples exist, the better the representation of the new image.

In this case y can be written as;

$$\begin{array}{c} y=Ax_{\circ } \end{array}$$ (4)

where

$$\begin{array}{c} x_{\circ }=[0,\ldots ,0,\alpha_{i,1},\alpha_{i,2},\ldots ,\alpha_{i,ni},0,{\ldots 0]}^{T} \end{array}$$ (5)

represents a sparse coefficient vector with all zero values excluding for the ones linked with the *i*^*th*^ user. Eq (4) then represents an under-determined sparse linear system that can be solved for sparce coefficient vector *x*_∘_, using any of the possible optimization approaches such as *l*_1_-minimization or least square minimization approach, such that input image *y* can be approximated from linear combination of matrix A.

Although least square minimization based approaches are generally not considered to be as accurate as *l*_1_-minimization based approaches they tend to be simpler to implement and quicker in processing, as a result a least square minimization based approach is proposed. The implementation of sparse representation based approaches will be performed using in MATLAB with the help of LSQR function used to solve Eq (4) using linear least square minimization, such that $min_{x_{\circ }}\|Ax_{\circ }-y{\|}_{2}$. The matching results will be verified and the results will be shown for Rank Zero(0) matching only i.e. only the highest scoring enrollment image will be compared with the gallery image and marked as match or non-match. The SSR is summarized in Algorithm 1.

**Algorithm 1** An algorithm for spacial sparse base representation

**Input:** Pre-processed Training Set I (with C classes) and Testing Images

**Output:** Sparse coefficient vector *x*_∘_ and Matching Score

*Training Set Processing*:

 1. Image resizing and vectorization

 2. Mean centering of resized image

 3. Generation of enrollment database matrix “A”

*Testing Image Processing*:

 1. Image resizing and vectorization

 2. Mean centering of resized image to generate new input “y”

*Sparse Representation*:

 Linear least square minimization, such that $min_{x_{\circ }}\|Ax_{\circ }-y{\|}_{2}$ (to generate sparse coefficient vector and enrolled images list for match score).

*Frequency Sparse base Representation (FSR)*. In frequency sparse representation the images are first converted to frequency domain. Considering a training set *I* with *C* being the number of classes then the images of training dataset are first converted into frequency domain using Fast Fourier Transformed such that for *i*^*th*^ class, training image *f* of dimension *u* × *v* can be given as;

$$\begin{array}{c} F\left(p+1,q+1\right)=\sum_{j=0}^{v-1}\sum_{k=0}^{u-1}e^{-2\pi i/u}e^{-2\pi i/v}f_{n}\left(j+1,k+1\right) \end{array}$$ (6)

The resulting spectral images are centered and extracted to sub-images, from ordinates (*p*1, *q*1) to (*p*2, *q*2) such that;

$$\begin{array}{c} g(p,q)=f[p1:p2,q1:q2] \end{array}$$ (7)

where *f*[*p*1: *p*2, *q*1: *q*2] is array slicing operator. These subspace images are vectorized to column vector *V*_*i*,*n*_ to generate sample set *I*_*i*_ of Eq 1. Similar to SSR, the enrollment database matrix “A” of Eq 2 is generated by horizontally stacking all the frequency spectrum vectorized images.

Similar frequency conversion using FFT and vectorization of its sub-image is also performed for testing images. Then the sparse representation of test image is obtained by using Eq (4) and solving it for for sparce coefficient vector *x*_∘_. The implementation of frequency sparse representation based approaches will also be performed using in MATLAB with the help of LSQR function used to solve Eq (4) using linear least square minimization, such that $min_{x_{\circ }}\|Ax_{\circ }-y{\|}_{2}$. The highest scoring enrollment image will be compared with the training images. The FSR is summarized in Algorithm 2.

**Algorithm 2** An algorithm for frequency sparse base representation

**Input:** Pre-processed Training Set I (with C classes) and Testing Images

**Output:** Sparse coefficient vector *x*_∘_ and Matching Score

*Training Set Processing*:

 1. Frequency domain conversion using Fast Fourier Transform (FFT).

 2. Frequency spectrum centered

 3. Subspace image extraction using centered spectrum and its vectorization

 4. Horizontal concatenation of vectorized sub-images to generate matrix “A”

*Testing Image Processing*:

 1. Frequency domain conversion using Fast Fourier Transform (FFT).

 2. Subspace image extraction and vectorization to generate new input “y”

*Sparse Representation*:

 Linear least square minimization, such that $min_{x_{\circ }}\|Ax_{\circ }-y{\|}_{2}$ (to generate sparse coefficient vector, and enrolled images list for match scoring).

### Face image databases

SCface database is taken as benchmark for experimental setup and related evaluation of proposed method. It is further verified using the CASIA NIR-VIS 2.0.

#### SCface database

The database consisting of 4160 images (visual and IR) of 130 subjects has been used [56]. In this database images are taken in uncontrolled indoor environment by using different quality surveillance cameras and at different distances; this corresponds to real world scenario. In experimental setup seven cameras are used, where cameras from Cam1 to Cam5 are used for surveillance visual images. On the other hand cameras Cam6 and Cam7 are used for surveillance IR images.

**Fig 2:**
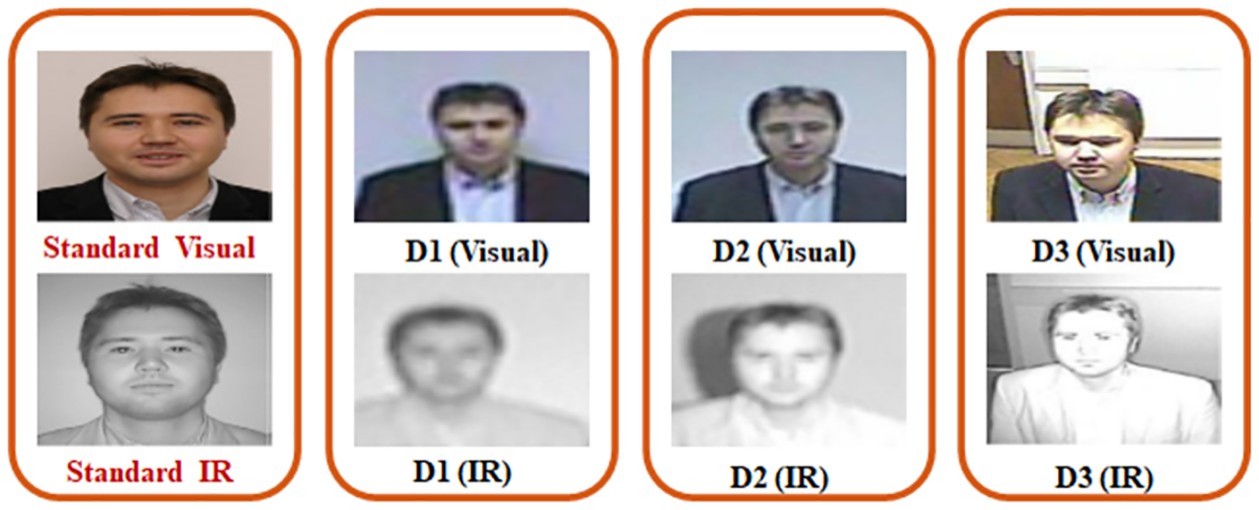
Illustration of visual and IR imagery, SCface database (The individual in this manuscript has given written informed consent for publication).

The key characteristics of probe face images subsets of SCface database are listed in Table 1, where pictures are taken for all the cameras are taken at D1 = 4.2 meter, D2 = 2.6 meter, and D3 = 1.0 meter. The eye coordinates along with the position of the nose tip and center of mouth are manually collected using a software specially developed for this purpose. Some sample face images from SCface databases are shown in Fig 2. Where both visual and IR images are shown at distance D1, D2, and D3.

**Table 1:** Key characteristics of probe face images subsets of SCface database.

Datasets	Scenario	No of cameras	Distances(meters)	Subjects	Images
D1	Visual	5	d1 = 4.20	130	650
	IR	2	d1 = 4.20	130	260
D2	Visual	5	d2 = 2.60	130	650
	IR	2	d2 = 2.60	130	260
D3	Visual	5	d3 = 1.00	130	650
	IR	2	d3 = 1.00	130	260

To evaluate the effect of image size on the matching process both SSR and FSR codes will be run multiple times with different size enrollment and probe images each time. This will allow us to evaluate the approaches (SSR and FSR) for different face image sizes. The sizes (s1,s2,s3,s4,s5,s6,s7, and s8) will be used, in SCface database. For SSR these sizes will be 8x8, 12x12, 15x15, 20x20, 25x25, 30x30, 35x35 and 40x40. While for FSR sizes will be 8x8, 12x12, 16x16, 20x20, 24x24, 32x32, 36x36, and 40x40.

**Fig 3:**
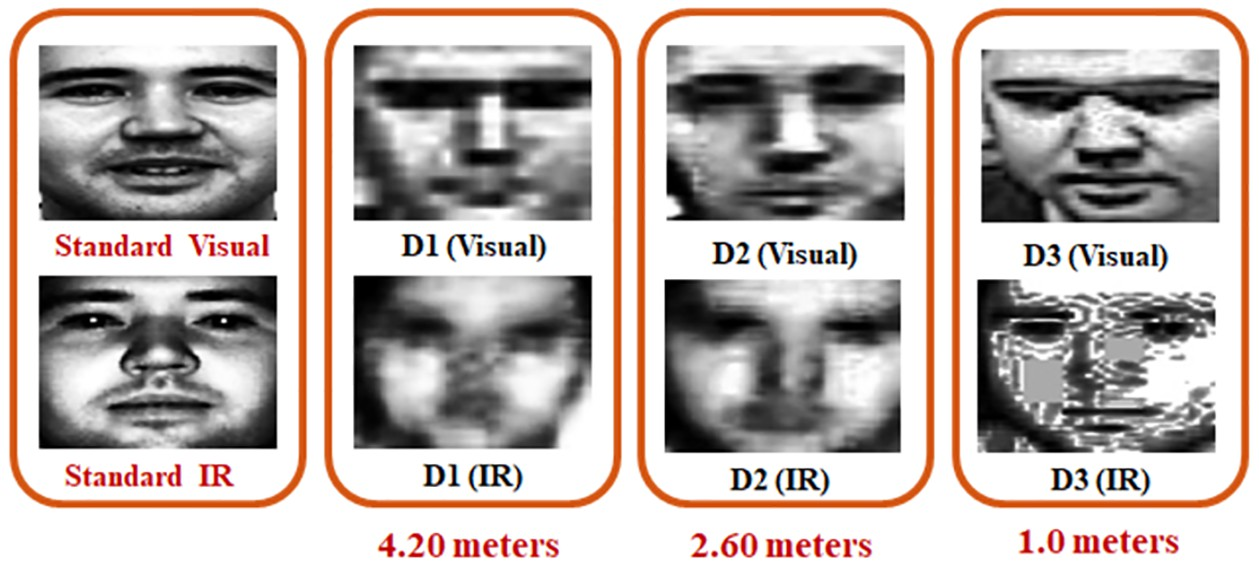
Illustration of preprocessed visual and IR images, SCface database (The individual in this manuscript has given written informed consent for publication).

In Fig 2 sample images from Scface database is given, where participant has provided consent for publication. Their sample preprocessed face images are shown in Fig 3. The images are aligned, normalized and cropped for preprocessing. So that they can be used for face recognition. The individual pictured in Figs 2 and 3 has provided written informed consent (as outlined in PLOS consent form) to publish their image alongside the manuscript.

#### CASIA NIR-VIS 2.0 database

In order to demonstrate the effectiveness of the suggested method, the CASIA NIR-VIS 2.0 [36] face database is also used as benchmark for the experimental setting and associated evaluation. The CASIA NIR-VIS 2.0 is a large database with 725 participants in total, using a similar image acquisition method and devices as that of heterogeneous face biometrics (HFB) face database [58]. Each participant has 1-22 visual (VIS) and 5-50 NIR facial photographs, totaling 17580 images. The raw VIS images are in JPEG format and that of NIR images in BMP format. Their resolutions are both 640. The images in the NIR-VIS 2.0 database were gathered over the course of four recording sessions with detail given in Table 2.

**Table 2:** Detail of image collection in CASIA NIR-VIS 2.0 database.

Session	Year of Collection	New Subjects	Old Subjects	Total Images
S1	Spring 2007	202	−	202
S2	Summer 2009	294	14 (S1)	308
S3	Fall 2009	177	1 (S1)	178
S4	Summer 2010	52	0	52
Total Original Subjects			725	
Total Images			17580	

The ages of all subjects range from infants to the elderly, facing the visual and NIR capture apparatus while seated in a chair. In order to get frontal photographs with neutral and grin expressions (or with and without spectacles) as well as two different distances, subjects are instructed to modify their expression and move closer to and farther away from the camera [58]. Therefore, the NIR-VIS 2.0 database has more changes in pose, eyewear, face expression, and distance; as a result, this database is closer to actual scenarios. The eye coordinates of the images are automatically labeled by an eye detector along with the manual correction of any error [36]. The experiments for CASIA NIR-VIS 2.0 database will be performed using single size.

### Experimental scenarios

The experiments will be conducted on two different datasets SCface and CASIA NIR-VIS 2.0 using the proposed methodology.

#### Experiments on SCface database

*Experiments on visual vs. visual*. In our first experiment, also called day time experiment, high quality frontal visual images of 130 subjects will be used as gallery set while visual datasets D1, D2, and D3 will be used as probe sets. We will start our evaluation with the day-time-experiments as described below;

1. Visual vs. visual scenario: The performance of proposed FSR and SSR methods for face image subsets D1, D2, and D3 will be evaluated and compared with state-of-the-art methods PCA, CKE, KFA [56, 59, 60] and recently developed LRPP-GRR method [46].
2. Varying image sizes scenario: The performance of proposed FSR and SSR methods will also be evaluated for different image sizes (s1, s2, s3, s4, s5, s6, s7, and, s8) for three image datasets D1, D2, and D3.

*Experiments on IR vs. IR*. In our second experiment, also called night time experiment, high quality frontal IR images of 130 subjects will be used as gallery set while IR datasets D1, D2, and D3 will be used as probe sets. We will start our evaluation with night-time-experiments as described below;

1. IR vs. IR scenario: The performance of proposed FSR and SSR methods for face image IR subsets D1, D2, and D3 will be evaluated and compared with state-of-the-art PCA method proposed in [56] as there will be no other research available for comparison as reported in [61]. KFA and LRPP-GRR is also used in experiments for comparison purpose.
2. Varying image sizes scenario: The performance of proposed FSR and SSR methods will also be evaluated for different image sizes (s1, s2, s3, s4, s5, s6, s7, and, s8) for three IR image datasets D1, D2, and D3.

*Experiments on visual vs. IR*. In our third experiment, also called day/ night time experiment, high quality frontal visual images of 130 subjects will be used as gallery set while IR datasets D1, D2, and D3 will be used as probe sets. We will start our evaluation with day/night-time-experiments as described below;

1. Visual vs. IR scenario: The performance of proposed FSR and SSR methods for face image IR subsets D1, D2, and D3 will be evaluated and compared with state-of-the-art method in [56] as there will be no other research available for comparison, as reported in [61]. KFA and LRPP-GRR is also used in experiments for comparison purpose.
2. Varying image sizes scenario: The performance of proposed FSR and SSR methods will also be evaluated for different image sizes (s1, s2, s3, s4, s5, s6, s7, and, s8) for three IR image datasets D1, D2, and D3.

#### Experiments on CASIA NIR-VIS 2.0 database

We have conducted most challenging experiment on visual vs. IR also called day/night on CASIA-VIS 2.0 consisting of 725 individuals with 17850 Visual and infrared images. One visual and IR image per person is randomly chosen for training and assessment process rather than several pictures per individual. This will be beneficial to assess the effectiveness of our proposed SSR and FSR techniques. Furthermore, it will also replicate real-world face recognition situations with a smaller training set and larger gallery as suggested in [62], which is also implemented for CASIA NIR-VIS 2.0 in [63, 64]. At the end the performance of proposed FSR and SSR methods for CASIA NIR-VIS 2.0 face image will be evaluated and compared with state-of the-art methods, PCA, LDA, KFA, OpenBR and LRPP-GRR, used in [46, 59, 65–67].

### Evaluation parameters

In all the experiments the results are compared with state of art methods for accuracy. Furthermore, performance metrics, such as precession, recall, F1-Score and Specificity is also taken to evaluate the recognition performance of the proposed model. Let TP, be true positive value, TN, true negative value, FP, false positive value and FN be the false negative value of a confusion matrix. Then the % recognition accuracy is given as;

$$\begin{array}{c} Accuracy=\frac{TP+TN}{TP+TN+FP+FN}\times 100\% \end{array}$$ (8)

The % precision value is given as;

$$\begin{array}{c} Precision=\frac{TP}{TP+FP}\times 100\% \end{array}$$ (9)

The % Recall value, for number of samples predicted correctly to be belonging to the positive class out of all the samples that actually belong to the positive class, is given as;

$$\begin{array}{c} Precision=\frac{TP}{TP+FN}\times 100\% \end{array}$$ (10)

F1 Score parameter is given as;

$$\begin{array}{c} F1\;Score=2\frac{Precision\times Recall}{Precision+Recall}\times 100\% \end{array}$$ (11)

and specificity, the number of samples predicted correctly to be in the negative class, is given as;

$$\begin{array}{c} Specificity=\frac{TN}{FP+TN}\times 100\% \end{array}$$ (12)

## Results

The sparse matching based approach in spatial and frequency domain for both visual and IR facial image sets has been presented in this paper. This section outlines the results and discusses the performance of the SSR and FSR at different distances and with different size images. The study takes a detailed look into the performance of proposed methods on multiple image sizes at multiple distances by multiple cameras to compare and evaluate it for real world applications. The first experiment evaluates the performance of proposed methods on day time scenario and results are compared with the existing benchmark and state-of-the-art algorithms. The results of the second experiment are compared to those of cutting-edge algorithms as it assesses the performance of suggested solutions in a nighttime environment. The second experiment evaluates the performance of proposed methods on night time scenario and their results are compared with the state-of-the-art algorithm as there was the only ones available in the literature. In third and last experiment, the performance of proposed methods is evaluated on day/night time scenario and results are compared with state-of -the-art algorithm as only one was available in the literature. This experiment is conducted for both SCface and CASIA NIR-VIZ 2.0 databases. The recognition accuracies clearly show that the proposed methods work better than the state-of-the-art methods. Furthermore, performance matrices such as precession, recall, F1-Score and Specificity is also given. In addition, evaluation and observation of the results provide us with interesting insights, some of which have been discussed in the current study.

### SCface database

#### Day-time experiment (visual vs. visual)

**Fig 4:**
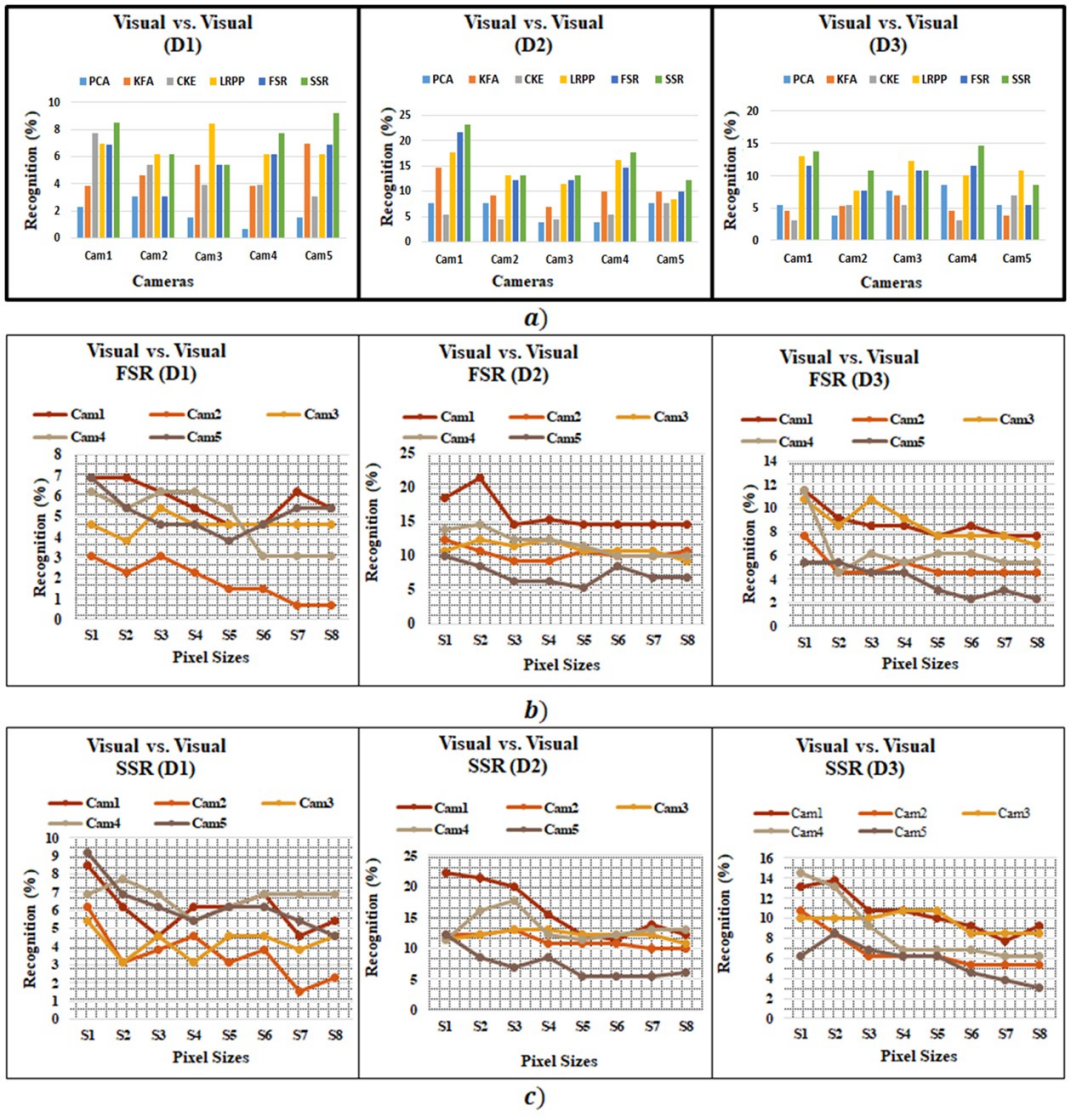
Visual vs. Visual Recognition (a) Accuracies of proposed and existing methods, (b) experiments for FSR Method of different image sizes, and (c) experiments for SSR Method of different image sizes.

We compare the recognition performance of proposed method with existing state-of-the-art methods given in [31, 59] and [60]. The benchmark PCA gives highest recognition accuracy for visual vs. visual as 7.7% while taking the complete dataset in consideration as suggested in [56]. Coupled kernel embedding (CKE) [60] method also considers the complete data set and reported recognition accuracies at distance 4.2m are 7.7%, 5.4%, 3.9%, 3.9% and 3.1% for cameras 1 to 5, respectively, which are consistently better than benchmark results as reported in [56]. The benchmark KFA [59] gives highest recognition accuracy for visual vs. visual at distance 2.60m with highest recognition is 14.62%It should be noted that result in [56] at distances 2.6m and 1.0m meter are not always superior to base line except for Cam 5. The RQS [30] gives recognition accuracy of 22.1% for highest quality images. The benchmark LRPP-GRR [46] gives highest recognition accuracy for visual vs. visual at distance 2.60m with highest recognition is 17.69%. On the other hand the proposed methods including SSR and FSR are implemented for all cameras and at all distances given in standard SCface database. As shown in Table 3 and Fig 4(a), SSR clearly outperforms for entire database, compared to base line results presented in [56] and CKE approach presented in [60] and result form KFA [59]. SSR and FSR give highest recognition accuracies of 23.1% and 21.6% at middle distance (d2 = 2.60m), respectively. However, SSR performs better than FSR for varying image size scenario in case of visual vs. visual match as shown in Fig 4(b) and 4(c) and Table 4.

**Table 3:** Recognition accuracies of proposed and existing methods for visual vs. visual.

Methods			Cam1	Cam2	Cam3	Cam4	Cam5
PCA		D1	2.3	3.1	1.5	0.7	1.5
		D2	7.7	7.7	3.9	3.9	7.7
		D3	5.4	3.9	7.7	8.5	5.4
CKE		D1	7.7	5.4	3.9	3.9	3.1
		D2	5.4	4.5	4.5	5.4	7.7
		D3	3.1	5.4	5.4	3.1	7
KFA		D1	3.85	4.62	4.28	3.85	5.92
		D2	14.62	9.23	6.92	10	10
		D3	4.62	5.38	6.92	4.62	3.85
LRPP-GRR		D1	6.92	6.15	8.46	6.15	6.15
		D2	17.69	13.7	11.53	16.15	8.46
		D3	13.07	7.69	12.3	10	10.76
Proposed	FSR	D1	6.9	3.1	5.4	6.2	6.9
		D2	21.5	12.3	12.3	14.6	10
		D3	11.5	7.7	10.8	11.5	5.4
	SSR	D1	8.5	6.2	5.4	7.7	9.2
		D2	23.1	13.1	13.1	17.7	12.3
		D3	13.8	10.8	10.8	14.6	8.5

**Table 4:** Recognition accuracies for FSR and SSR Method of different image sizes (visual vs. visual).

Methods			S1	S2	S3	S4	S5	S6	S7	S8
FSR	D1	Cam1	6.9	6.9	6.2	5.4	4.6	4.6	6.2	5.4
		Cam2	3.1	2.3	3.1	2.3	1.5	1.5	0.7	0.7
		Cam3	4.6	3.8	5.4	4.6	4.6	4.6	4.6	4.6
		Cam4	6.2	5.4	6.2	6.2	5.4	3.1	3.1	3.1
		Cam5	6.9	5.4	4.6	4.6	3.8	4.6	5.4	5.4
	D2	Cam1	18.5	21.5	14.6	15.4	14.6	14.6	14.6	14.6
		Cam2	12.3	10.8	9.2	9.2	10.8	10	10	10.8
		Cam3	10.8	12.3	11.5	12.3	10.8	10.8	10.8	9.2
		Cam4	13.8	14.6	12.3	12.3	11.5	10	10	10
		Cam5	10	8.5	6.2	6.2	5.4	8.5	6.9	6.9
	D3	Cam1	11.5	9.2	8.5	8.5	7.7	8.5	7.7	7.7
		Cam2	7.7	4.6	4.6	5.4	4.6	4.6	4.6	4.6
		Cam3	10.8	8.5	10.8	9.2	7.7	7.7	7.7	6.9
		Cam4	11.5	4.6	6.2	5.4	6.2	6.2	5.4	5.4
		Cam5	5.4	5.4	4.6	4.6	3.1	2.3	3.1	2.3
SSR	D1	Cam1	8.5	6.2	4.6	6.2	6.2	6.9	4.6	5.4
		Cam2	6.2	3.1	3.8	4.6	3.1	3.8	1.5	2.3
		Cam3	5.4	3.1	4.6	3.1	4.6	4.6	3.8	4.6
		Cam4	6.9	7.7	6.9	5.4	6.2	6.9	6.9	6.9
		Cam5	9.2	6.9	6.2	5.4	6.2	6.2	5.4	4.6
	D2	Cam1	23.1	21.5	20	15.4	12.3	11.5	13.8	12.3
		Cam2	12.3	12.3	13.1	10.8	10.8	10.8	10	10
		Cam3	11.5	12.3	13.1	13.1	12.3	12.3	12.3	10.8
		Cam4	11.5	16.2	17.7	12.3	11.5	12.3	13.1	13.1
		Cam5	12.3	8.5	6.9	8.5	5.4	5.4	5.4	6.2
	D3	Cam1	13.1	13.8	10.8	10.8	10	9.2	7.7	9.2
		Cam2	10.8	8.5	6.2	6.2	6.2	5.4	5.4	5.4
		Cam3	10	10	10	10.8	10.8	8.5	8.5	8.5
		Cam4	14.6	13.1	9.2	6.9	6.9	6.9	6.2	6.2
		Cam5	6.2	8.5	6.9	6.2	6.2	4.6	3.8	3.1

Another interesting observation is highest accuracy achieved at dataset D2 (middle distance) which is attributed to the ability of sparse representation based approach to cope well for small sized face images in face recognition scenarios [8, 68, 69], but it does have its limits. For larger distance (i.e., dataset D1) it would have the smallest possible face size and at that small face size sparse representation based approaches tend to fail more often than not. On the other side, at the nearest distance (dataset D3) the face images may be large enough for sparse representation based approaches to work effectively but as the image size increases so does the noise in the image. This means, although the image size may be more suitable for sparse representation based approach but the quality of the image also decreases significantly. This may be the reason that the proposed methods consistently provided better results at middle distance.

Fig 4(a) and 4(b) show the performance of FSR and SSR for different image sizes. It can be seen from the plots that the highest matching occurs for 8x8 or 12x12 image size. The matching consistently reduces as the image size increase. This is in line with the fact that as the image size increases the finer features as well as noise are more visible and therefore matching is reduced. In Table 5 quantitative evaluation metrics such as precession, recall, F1-Score and Specificity, for Visual vs. Visual experiments, are given. FSR gives precision of 14.6%, recall of 21.5%, F1-score of 15.76% and specificity of 99.4% for camera at distance d2. Similarly, SSR also shows precision of 12.94%, recall of 17.7%, F1-score of 13.84% and specificity of 99.36% for camera at distance d2. The specificity values are very high which mean that the model is good at avoiding false positives in the negative class. The test images are heterogeneous therefore accuracy and precision are low.

**Table 5:** Quantitative evaluation metrics of proposed methods (visual vs. visual).

Methods			Precision	Recall	F1-Score	Specificity
FSR	D1	Cam1	4.9	6.9	5.3	99.2
		Cam2	2.32	3.1	2.33	99.25
		Cam3	1.94	4.6	2.4	99.26
		Cam4	3.6	6.2	4.13	99.27
		Cam5	4.91	6.9	5.34	99.28
	D2	Cam1	14.6	21.5	15.76	99.4
		Cam2	8.58	12.3	9.18	99.3
		Cam3	9.73	12.3	10.28	99.32
		Cam4	10	14.6	11	99.34
		Cam5	6.05	10	6.94	99.3
	D3	Cam1	6.44	11.5	7.31	99.3
		Cam2	4.58	7.7	4.97	99.28
		Cam3	7.75	10.8	8.17	99.31
		Cam4	7.58	11.5	8.26	99.31
		Cam5	3.38	5.4	3.85	99.27
SSR	D1	Cam1	4	8.5	4.63	99.29
		Cam2	4.23	6.2	4.45	99.27
		Cam3	2.64	5.4	3.19	99.27
		Cam4	4.93	7.7	5.41	99.28
		Cam5	7.92	11.5	8.48	99.31
	D2	Cam1	15.4	23.1	9.16	99.41
		Cam2	8.58	13.1	9.42	99.33
		Cam3	8.58	13.1	9.42	99.33
		Cam4	12.94	17.7	13.84	99.36
		Cam5	7.48	12.3	8.45	99.31
	D3	Cam1	8.88	13.8	9.87	99.33
		Cam2	7.75	10.8	8.17	99.3
		Cam3	9.33	10.8	9.66	99.31
		Cam4	9.67	14.6	10.86	99.34
		Cam5	4	8.5	4.63	99.29

#### Night-time experiment (IR vs. IR)

**Fig 5:**
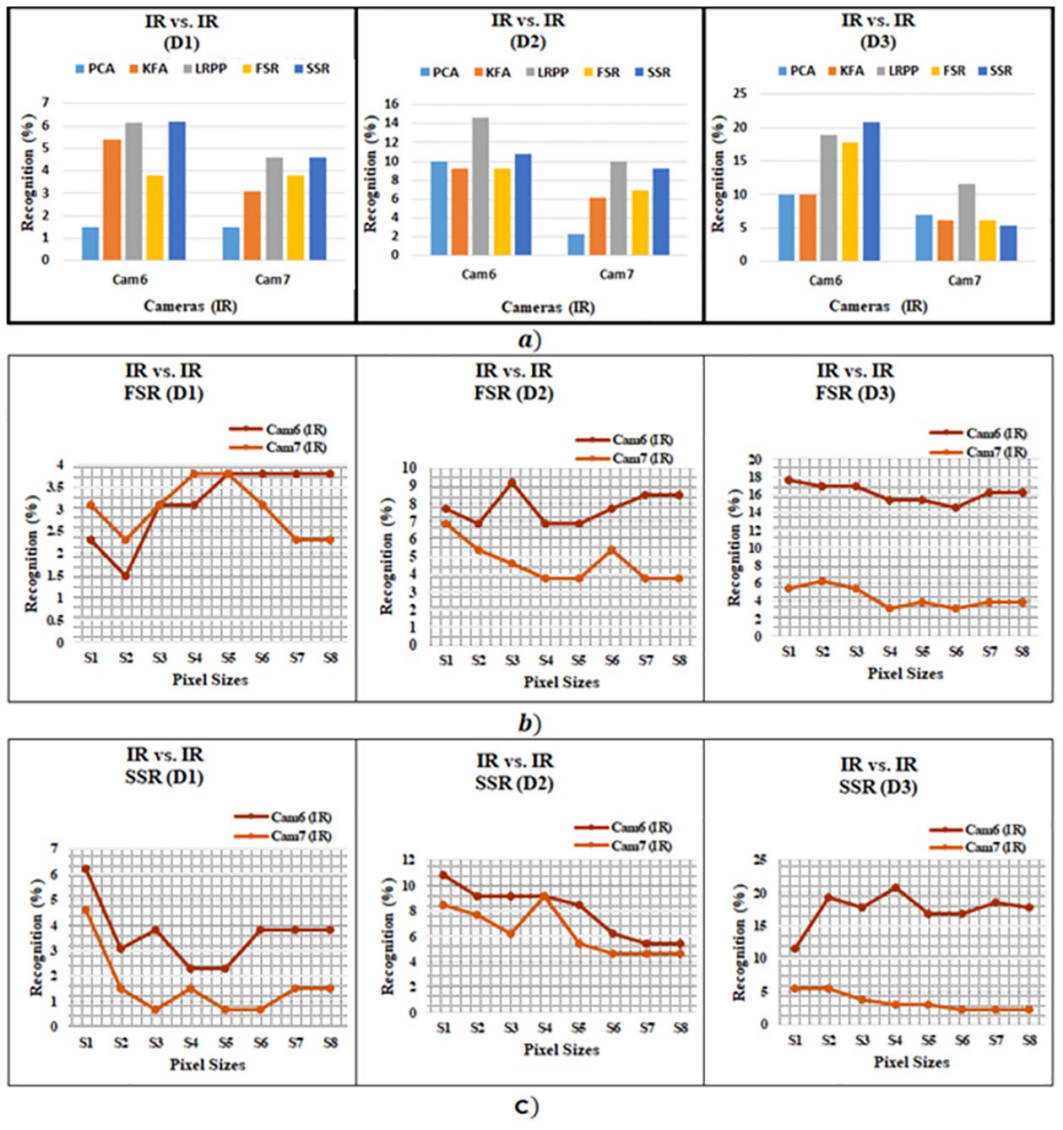
IR vs IR Recognition (a) Accuracies of proposed and existing methods, (b) experiments for FSR Method of different image sizes, and (c) experiments for SSR Method of different image sizes.

The next step in continuing to evaluate the performance of sparse representation based approaches for homogeneous facial recognition is to perform night time experiment. Night-time experiment is based on comparing high quality IR gallery images with a low quality IR probe image. The results are compared against the benchmark PCA based approach in [56], which is the only available results for complete SCface dataset as reported in [61]. The result is also compared with KFA based technique initially proposed in [59]. The Benchmark PCA and KFA give highest recognition accuracy for IR vs. IR as 10% [56], the benchmark LRPP-GRR [46] gives highest recognition accuracy at distance d3 with highest recognition is 18.9%. While by using SSR and FSR the highest recognition accuracies are 20.8% and 17.7%, respectively. It can be observed that SSR have consistently higher recognition performance as compared to benchmark PCA and KFA, except for Cam7 at the farthest distance. On the other hand, FSR has better recognition accuracy than the benchmark for most cameras except for Cam6 at middle distance and Cam7 at near distance, as shown in Table 6 and Fig 5(a).

**Table 6:** Recognition accuracies of proposed and existing methods for IR vs. IR.

Methods			Cam6	Cam7
PCA		D1	1.5	1.5
		D2	10	2.3
		D3	10	7.0
KFA		D1	5.38	3.08
		D2	9.23	6.15
		D3	10	6.15
LRPP-GRR		D1	6.15	4.61
		D2	14.6	10
		D3	18.9	11.53
Proposed	FSR	D1	3.8	3.8
		D2	9.2	6.9
		D3	17.7	6.2
	SSR	D1	6.2	4.6
		D2	10.8	9.2
		D3	20.8	5.4

The performance of FSR and SSR is also analyses in terms of size of the images. In Fig 5(b) and 5(c), as well as in Table 7, it can be observed that Cam6 is consistently performing better than Cam7 in terms of number of matches at different image sizes. The best possible matching occurs at the image size of 16x16 and 20x20 pixels. This is different for day time matching scenario, which worked best at smaller sizes of images. This is due to the reason that IR images tend to be blurry and low in quality as compared to standard visual imagery. At smaller face size the features, therefore, are not as clearly visible in IR as they would be in their counterpart visual images. In case of images with farthest distance, the smaller sized images seem to perform better but there is a visible improvement in matching for images with 16x16 and/or 20x20 image size.

**Table 7:** Recognition accuracies for FSR and SSR Method of different image sizes (IR vs. IR).

Methods			S1	S2	S3	S4	S5	S6	S7	S8
FSR	D1	Cam6	2.3	1.5	3.1	3.1	3.8	3.8	3.8	3.8
		Cam7	3.1	2.3	3.1	3.8	3.8	3.1	2.3	2.3
	D2	Cam6	7.7	6.9	9.2	6.9	6.9	7.7	8.5	8.5
		Cam7	6.9	5.4	4.6	3.8	3.8	5.4	3.8	3.8
	D3	Cam6	17.7	16.9	16.9	15.4	15.4	14.6	16.2	16.2
		Cam7	5.4	6.2	5.4	3.1	3.8	3.1	3.8	3.8
SSR	D1	Cam6	6.2	3.1	3.8	2.3	2.3	3.8	3.8	3.8
		Cam7	4.6	1.5	0.7	1.5	0.7	0.7	1.5	1.5
	D2	Cam6	10.8	9.2	9.2	9.2	8.5	6.2	5.4	5.4
		Cam7	8.5	7.7	6.2	9.2	5.4	4.6	4.6	4.6
	D3	Cam6	11.5	19.2	17.7	20.8	16.9	16.9	18.5	17.7
		Cam7	5.4	5.4	3.8	3.1	3.1	2.3	2.3	2.3

In Table 8 quantitative evaluation metrics such as precession, recall, F1-Score and Specificity, for IR vs. IR experiments, are given. FSR gives precision of 11.6%, recall of 17.7%, F1-score of 12.74% and specificity of 99.36% for Cam6 at distance d2. Similarly, SSR also shows precision of 13.33%, recall of 20%, F1-score of 14.6% and specificity of 99.38% for Cam6 at distance d3. A high specificity values mean that the model avoids false positives efficiently. The test images are heterogeneous therefore accuracy and precision are low even then that of Visual vs. Visual experiments.

**Table 8:** Quantitative evaluation metrics of proposed methods (IR vs. IR).

Methods			Precision	Recall	F1-Score	Specificity
FSR	D1	Cam6	1.56	3.1	1.84	99.25
		Cam7	2.37	3.8	2.74	99.25
	D2	Cam6	3.96	9.2	5.07	99.3
		Cam7	3.92	6.9	4.34	99.28
	D3	Cam6	11.66	17.7	12.74	99.36
		Cam7	2.78	6.2	3.6	99.27
SSR	D1	Cam6	2.78	6.2	3.6	99.27
		Cam7	1.9	4.6	2.4	99.25
	D2	Cam6	3.59	10.8	4.9	99.31
		Cam7	4.38	9.2	5.11	99.3
	D3	Cam6	13.33	20.8	14.6	99.38
		Cam7	3.48	5.4	3.89	99.27

#### Day / night-time experiment (Visual vs. IR)

The most interesting and challenging task is the day vs. night recognition i.e. heterogeneous face recognition. This particular experiment implies matching a high quality visual gallery image with a low quality IR probe image. As mentioned above, it is one of the more common real world scenarios that occur when the CCTV camera is IR based and the gallery database is visual e.g. the national identification card database or immigration database. In such cases the heterogeneous facial recognition is taking place. It is one of the more recently identified problems and as such not much literature is available on this problem. The benchmark system does perform heterogeneous face recognition on SCface database but it seems to have been done more for sake of completion then to develop a formal approach. On the other hand the matching in this paper has been performed in order to develop a formal system for heterogeneous facial recognition with low quality imagery.

**Fig 6:**
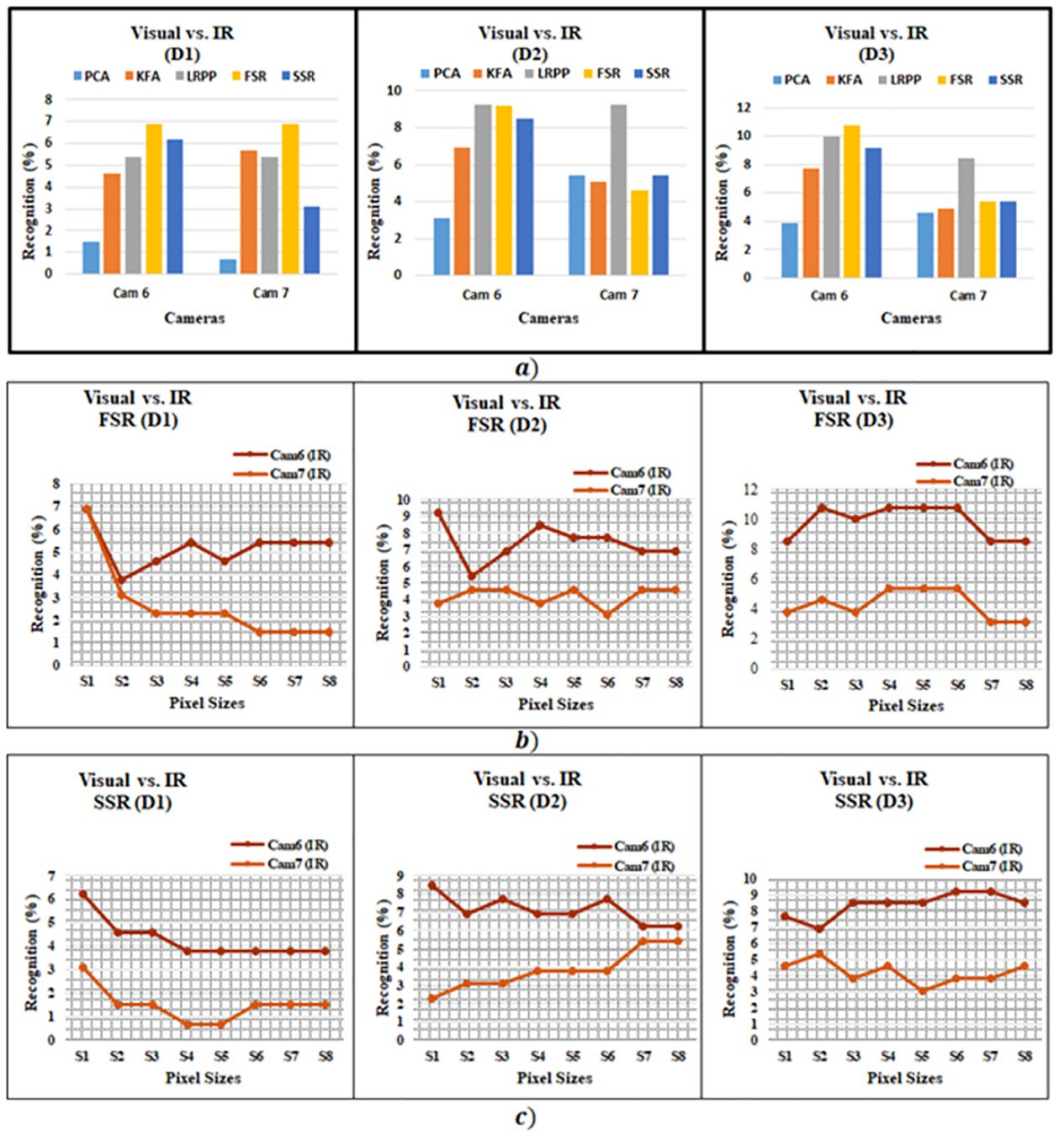
Visual vs. IR recognition (a) Accuracies of proposed and existing methods, (b) experiments for FSR Method of different image sizes, and (c) experiments for SSR Method of different image sizes.

Table 9 and Fig 6(a) compares the benchmark PCA, KFA and LRPP-GRR, against our proposed methods. For PCA the highest recognition accuracy for Visual vs. IR is 5.4% at distance d2 using Cam7. For KFA the highest recognition accuracy is 7.69% at distance d3 using Cam6. While LRPP-GRR shows an accuracy of 10% on d2, Cam6. SSR on the other hand has the highest recognition accuracy of 9.2% at distance d2 using Cam6. It is interesting to note that both the PCA, KFA and SSR provide the similar accuracy for distance d2 and Cam7. The highest recognition accuracy for FSR is 10.8%. It is interesting to note that FSR performs consistently better than the PCA, KFA and proposed SSR except for Cam7 at middle distance. On the other hand although LRPP-GRR shows maximum accuracy of 10% which is lower than that of FSR, it performs better Than other algorithms for Cam7. One of the reasons for such a low matching results is attributed to the fact that both images are in different modality and therefore have different visual features.

**Table 9:** Recognition accuracies of proposed and existing methods for visual vs. IR.

Methods			Cam6	Cam7
PCA		D1	1.5	0.7
		D2	3.1	5.4
		D3	3.9	4.6
KFA		D1	4.62	5.69
		D2	6.92	5.1
		D3	7.69	4.9
LRPP-GRR		D1	5.38	5.38
		D2	9.2	9.2
		D3	10	8.46
Proposed	FSR	D1	6.9	6.9
		D2	9.2	4.6
		D3	10.8	5.4
	SSR	D1	6.2	3.1
		D2	8.5	5.4
		D3	9.2	5.4

Table 10 along with Fig 6(b) and 6(c) show the effect of image size on recognition performance. The figures show that the results for Cam6 are consistently better than that of Cam7 for both SSR and FSR. In Table 11 precession, recall, F1-Score and Specificity, for Visual vs. IR experiments, are given. FSR gives precision of 5.95%, recall of 10.8%, F1-score of 6.83% and specificity of 99.31% for Cam6 at distance d3. Similarly, SSR also shows precision of 5.5%, recall of 9.2%, F1-score of 6% and specificity of 99.3% for Cam6 at distance d3.

**Table 10:** Recognition experiments for FSR and SSR method of different image sizes (visual vs. IR).

Methods			S1	S2	S3	S4	S5	S6	S7	S8
FSR	D1	Cam6	6.9	3.8	4.6	5.4	4.6	5.4	5.4	5.4
		Cam7	6.9	3.1	2.3	2.3	2.3	1.5	1.5	1.5
	D2	Cam6	9.2	5.4	6.9	8.5	7.7	7.7	6.9	6.9
		Cam7	3.8	4.6	4.6	3.8	4.6	3.1	4.6	4.6
	D3	Cam6	8.5	10.8	10	10.8	10.8	10.8	8.5	8.5
		Cam7	3.8	4.6	3.8	5.4	5.4	5.4	3.1	3.1
SSR	D1	Cam6	6.2	4.6	4.6	3.8	3.8	3.8	3.8	3.8
		Cam7	3.1	1.5	1.5	0.7	0.7	1.5	1.5	1.5
	D2	Cam6	8.5	6.9	7.7	6.9	6.9	7.7	6.2	6.2
		Cam7	2.3	3.1	3.1	3.8	3.8	3.8	5.4	5.4
	D3	Cam6	7.7	6.9	8.5	8.5	8.5	9.2	9.2	8.5
		Cam7	4.6	5.4	3.8	4.6	3.1	3.8	3.8	4.6

**Table 11:** Quantitative evaluation metrics of proposed methods (visual vs. IR).

Methods			Precision	Recall	F1-Score	Specificity
FSR	D1	Cam6	2.83	6.9	3.46	99.28
		Cam7	2.25	6.9	3.06	99.28
	D2	Cam6	4.95	9.2	5.49	99.3
		Cam7	2.29	4.6	2.71	99.26
	D3	Cam6	5.95	10.8	6.83	99.31
		Cam7	4.7	5.4	4.8	99.27
SSR	D1	Cam6	2.83	6.9	3.7	99.28
		Cam7	0.8	3.1	1.19	99.25
	D2	Cam6	4.97	8.5	5.5.7	99.29
		Cam7	4.7	5.4	4.8	99.27
	D3	Cam6	5.5	9.2	6.02	99.3
		Cam7	4.7	5.4	4.8	99.27

The results from all the tests with SCface database clearly indicate that performance of all the approaches (benchmark, KFA, CKE, SSR and FSR) is lowest for the imagery acquired from Cam7 (Cam5 in case of visual). It would be interesting to evaluate these images in detail in order to identify the reasons as they may lead to overall improvement in performance.

### CASIA NIR-VIS 2.0 face database (Visual vs. IR)

**Fig 7:**
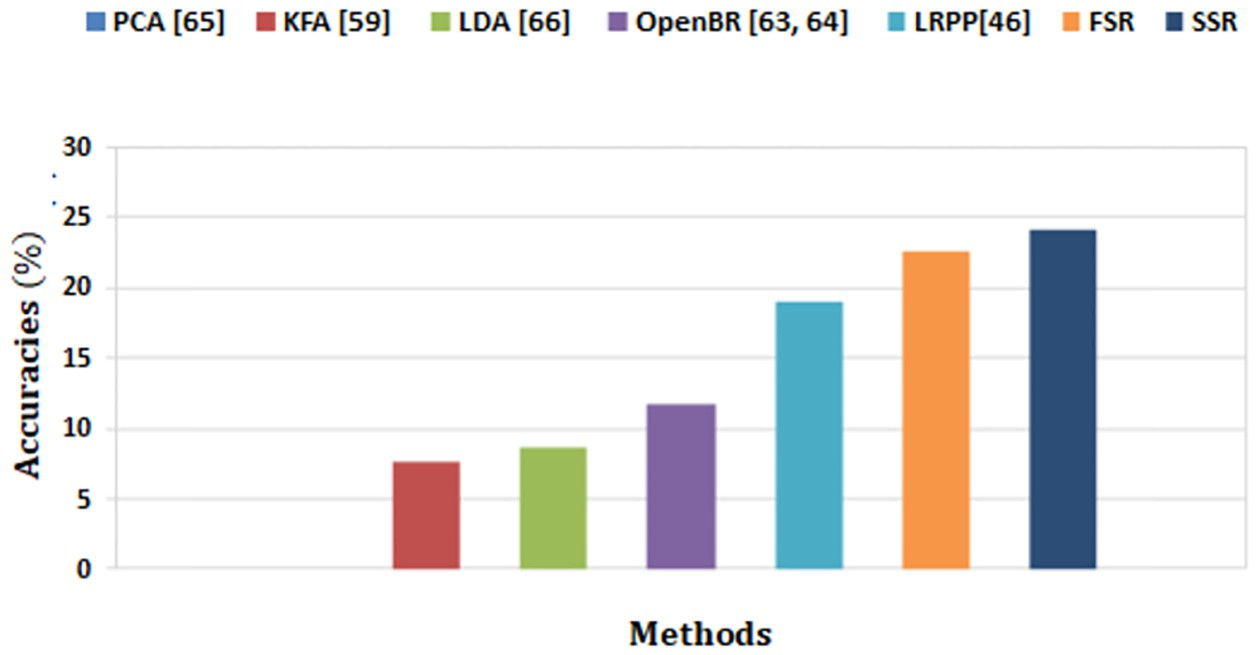
Recognition accuracies of proposed and existing methods for CASIA NIR-VIS 2.0.

The proposed methods is investigated by using NIR-VIS data sets for the most intriguing and difficult challenge, which is heterogeneous face recognition in day/night experiment (Visual vs. IR). In this experiment, a randomly selected different visual gallery image is desired to be matched with a changing IR probe image taking into consideration the frequent real-world case where a person only has a single image in the national database to match with an IR image captured at night. The result of suggested approaches is compared with the outcomes of the state-of-the-art algorithms PCA [65], LDA [66], OpenBR [63, 64], KFA [59] and LRPP-GRR [46]. It can be observed in Fig 7 that the highest Rank-0 recognition with the proposed FSR and SSR is 22.62% and 24.14% respectively as compared to the Rank-1 recognition accuracies in PCA is 0.0%, LDA is 8.72%, OpenBR is 11.76 % reported in [63, 64, 70], while that of KFA is 7.69%. The LRPP-GRR algorithm gives an accuracy of 19.05 % for this database. The result verifies that the proposed method with Rank-0 recognition performs better when compared with the state of art methods with Rank-1 recognition. It can also be observed that the proposed method gives similar results with both SCface and CASIA NIR-VIS 2.0 databases in similar conditions.

## Conclusion

In this paper, we have implemented sparse representation based methods on standard SCface and CASIA NIR-VIS 2.0 face databases for low quality visual and IR imagery in order to investigate heterogeneous low quality face recognition. We have proved that proposed methods work better than the state-of-the-art techniques used for low quality, single sample per person, facial recognition. The performance of these sparse representation based matching approaches has been evaluated on real world scenarios with different modality through CCTV images provided in benchmark SCface and CASIA NIR-VIS 2.0 face databases with images having variation in pose, expression, eyeglasses and distance. Firstly, the performance evaluation results have shown that the proposed methods work consistently better than the state-of-the-art methods across both modalities i.e. (visual vs. visual and IR vs. IR). Secondly, these methods also give superior performance in more complex situation of heterogeneous matching scenarios (i.e., visual vs. IR) using benchmark SCface and CASIA NIR-VIS 2.0 face databases. It is observed that for heterogeneous recognition, it is better to transform both gallery and probe images to a different domain (Frequency domain in current study). The future research direction may transform the heterogeneous gallery and probe image into other domains (such as gradient domain or polynomial domain) and evaluate how such approaches outperform. The evaluation of the dataset to identify the reasons for drop in recognition accuracy will also be performed.

## Supporting information

S1 File (DOCX)
