# Supplementary material for: On-the-move heterogeneous face recognition in frequency and spatial domain using sparse representation
Source: PLoS One. 2024 Oct 4;19(10):e0308566. doi: 10.1371/journal.pone.0308566 (PMC11451977; doi:10.1371/journal.pone.0308566)
Supplement: S1 File — (DOCX) [file pone.0308566.s001.docx]

**Final Score For Various Experiments in Scface Database**

**Final Score Scface SSR (IR) cam6-d1**

**Actual Image Recognized Image Score**

**001_cam6_1.jpg 001_cam8.jpg 0.442780**

**002_cam6_1.jpg 105_cam8.jpg 0.324714**

**003_cam6_1.jpg 013_cam8.jpg 0.689489**

**004_cam6_1.jpg 041_cam8.jpg 0.470426**

**005_cam6_1.jpg 051_cam8.jpg 0.698554**

**006_cam6_1.jpg 001_cam8.jpg 0.603263**

**007_cam6_1.jpg 097_cam8.jpg 0.416503**

**008_cam6_1.jpg 024_cam8.jpg 0.493871**

**009_cam6_1.jpg 033_cam8.jpg 0.478851**

**010_cam6_1.jpg 001_cam8.jpg 0.530763**

**011_cam6_1.jpg 013_cam8.jpg 0.582707**

**012_cam6_1.jpg 097_cam8.jpg 0.486082**

**013_cam6_1.jpg 013_cam8.jpg 0.624521**

**014_cam6_1.jpg 107_cam8.jpg 0.708495**

**015_cam6_1.jpg 041_cam8.jpg 0.431976**

**016_cam6_1.jpg 051_cam8.jpg 0.495752**

**017_cam6_1.jpg 087_cam8.jpg 0.466898**

**018_cam6_1.jpg 063_cam8.jpg 0.525581**

**019_cam6_1.jpg 021_cam8.jpg 0.342012**

**020_cam6_1.jpg 083_cam8.jpg 0.448329**

**021_cam6_1.jpg 021_cam8.jpg 0.523711**

**022_cam6_1.jpg 034_cam8.jpg 0.508800**

**023_cam6_1.jpg 040_cam8.jpg 0.413493**

**024_cam6_1.jpg 063_cam8.jpg 0.397820**

**025_cam6_1.jpg 103_cam8.jpg 0.265231**

**026_cam6_1.jpg 055_cam8.jpg 0.476601**

**027_cam6_1.jpg 104_cam8.jpg 0.265459**

**028_cam6_1.jpg 041_cam8.jpg 0.481833**

**029_cam6_1.jpg 063_cam8.jpg 0.390065**

**030_cam6_1.jpg 030_cam8.jpg 0.779629**

**031_cam6_1.jpg 065_cam8.jpg 0.410056**

**032_cam6_1.jpg 097_cam8.jpg 0.445013**

**033_cam6_1.jpg 128_cam8.jpg 0.553846**

**034_cam6_1.jpg 040_cam8.jpg 0.343171**

**035_cam6_1.jpg 043_cam8.jpg 0.562488**

**036_cam6_1.jpg 012_cam8.jpg 0.706766**

**037_cam6_1.jpg 008_cam8.jpg 0.430256**

**038_cam6_1.jpg 053_cam8.jpg 0.503619**

**039_cam6_1.jpg 049_cam8.jpg 0.465615**

**040_cam6_1.jpg 122_cam8.jpg 0.714585**

**041_cam6_1.jpg 041_cam8.jpg 0.502022**

**042_cam6_1.jpg 097_cam8.jpg 0.433236**

**043_cam6_1.jpg 043_cam8.jpg 0.472615**

**044_cam6_1.jpg 001_cam8.jpg 0.458521**

**045_cam6_1.jpg 124_cam8.jpg 0.415311**

**046_cam6_1.jpg 043_cam8.jpg 0.615972**

**047_cam6_1.jpg 041_cam8.jpg 0.359708**

**048_cam6_1.jpg 009_cam8.jpg 0.622321**

**049_cam6_1.jpg 034_cam8.jpg 0.449920**

**050_cam6_1.jpg 080_cam8.jpg 0.483479**

**051_cam6_1.jpg 013_cam8.jpg 0.333352**

**052_cam6_1.jpg 013_cam8.jpg 0.429731**

**053_cam6_1.jpg 001_cam8.jpg 0.434960**

**054_cam6_1.jpg 060_cam8.jpg 0.544289**

**055_cam6_1.jpg 034_cam8.jpg 0.666056**

**056_cam6_1.jpg 065_cam8.jpg 0.455791**

**057_cam6_1.jpg 097_cam8.jpg 0.566100**

**058_cam6_1.jpg 097_cam8.jpg 0.488091**

**059_cam6_1.jpg 063_cam8.jpg 0.331858**

**060_cam6_1.jpg 038_cam8.jpg 0.322584**

**061_cam6_1.jpg 001_cam8.jpg 0.453903**

**062_cam6_1.jpg 008_cam8.jpg 0.296629**

**063_cam6_1.jpg 001_cam8.jpg 0.433549**

**064_cam6_1.jpg 001_cam8.jpg 0.446241**

**065_cam6_1.jpg 097_cam8.jpg 0.378665**

**066_cam6_1.jpg 037_cam8.jpg 0.638491**

**067_cam6_1.jpg 038_cam8.jpg 0.334013**

**068_cam6_1.jpg 043_cam8.jpg 0.573775**

**069_cam6_1.jpg 093_cam8.jpg 0.335619**

**070_cam6_1.jpg 013_cam8.jpg 0.440467**

**071_cam6_1.jpg 112_cam8.jpg 0.665739**

**072_cam6_1.jpg 067_cam8.jpg 0.348233**

**073_cam6_1.jpg 063_cam8.jpg 0.465088**

**074_cam6_1.jpg 076_cam8.jpg 0.674901**

**075_cam6_1.jpg 114_cam8.jpg 0.372213**

**076_cam6_1.jpg 060_cam8.jpg 0.610623**

**077_cam6_1.jpg 065_cam8.jpg 0.573136**

**078_cam6_1.jpg 055_cam8.jpg 0.522465**

**079_cam6_1.jpg 063_cam8.jpg 0.418967**

**080_cam6_1.jpg 013_cam8.jpg 0.634318**

**081_cam6_1.jpg 013_cam8.jpg 0.453256**

**082_cam6_1.jpg 097_cam8.jpg 0.467940**

**083_cam6_1.jpg 048_cam8.jpg 0.602646**

**084_cam6_1.jpg 043_cam8.jpg 0.630550**

**085_cam6_1.jpg 008_cam8.jpg 0.694469**

**086_cam6_1.jpg 037_cam8.jpg 0.376362**

**087_cam6_1.jpg 100_cam8.jpg 0.488435**

**088_cam6_1.jpg 123_cam8.jpg 0.641141**

**089_cam6_1.jpg 060_cam8.jpg 0.400783**

**090_cam6_1.jpg 001_cam8.jpg 0.496319**

**091_cam6_1.jpg 040_cam8.jpg 0.592873**

**092_cam6_1.jpg 043_cam8.jpg 0.518376**

**093_cam6_1.jpg 083_cam8.jpg 0.821485**

**094_cam6_1.jpg 013_cam8.jpg 0.931169**

**095_cam6_1.jpg 104_cam8.jpg 0.427917**

**096_cam6_1.jpg 051_cam8.jpg 0.545983**

**097_cam6_1.jpg 038_cam8.jpg 0.399654**

**098_cam6_1.jpg 037_cam8.jpg 0.626589**

**099_cam6_1.jpg 013_cam8.jpg 0.408996**

**100_cam6_1.jpg 096_cam8.jpg 0.345432**

**101_cam6_1.jpg 065_cam8.jpg 0.378932**

**102_cam6_1.jpg 049_cam8.jpg 0.488498**

**103_cam6_1.jpg 104_cam8.jpg 0.470955**

**104_cam6_1.jpg 060_cam8.jpg 0.445335**

**105_cam6_1.jpg 002_cam8.jpg 0.746452**

**106_cam6_1.jpg 013_cam8.jpg 0.561149**

**107_cam6_1.jpg 013_cam8.jpg 0.559333**

**108_cam6_1.jpg 001_cam8.jpg 0.534354**

**109_cam6_1.jpg 043_cam8.jpg 0.448206**

**110_cam6_1.jpg 065_cam8.jpg 0.492379**

**111_cam6_1.jpg 063_cam8.jpg 0.372312**

**112_cam6_1.jpg 013_cam8.jpg 0.638307**

**113_cam6_1.jpg 114_cam8.jpg 0.289738**

**114_cam6_1.jpg 038_cam8.jpg 0.345507**

**115_cam6_1.jpg 115_cam8.jpg 0.496719**

**116_cam6_1.jpg 013_cam8.jpg 0.444353**

**117_cam6_1.jpg 063_cam8.jpg 0.354956**

**118_cam6_1.jpg 097_cam8.jpg 0.514813**

**119_cam6_1.jpg 037_cam8.jpg 0.439075**

**120_cam6_1.jpg 054_cam8.jpg 0.481895**

**121_cam6_1.jpg 060_cam8.jpg 0.383009**

**122_cam6_1.jpg 067_cam8.jpg 0.717389**

**123_cam6_1.jpg 100_cam8.jpg 0.441670**

**124_cam6_1.jpg 071_cam8.jpg 0.495034**

**125_cam6_1.jpg 040_cam8.jpg 0.660881**

**126_cam6_1.jpg 001_cam8.jpg 0.321986**

**127_cam6_1.jpg 020_cam8.jpg 0.501724**

**128_cam6_1.jpg 057_cam8.jpg 0.330499**

**129_cam6_1.jpg 063_cam8.jpg 0.400341**

**130_cam6_1.jpg 051_cam8.jpg 0.504792**

**Final Score Scface SSR (IR) cam6-d2**

**Actual Image Recognized Image Score**

**001_cam6_2.jpg 071_cam8.jpg 0.392694**

**002_cam6_2.jpg 067_cam8.jpg 0.364075**

**003_cam6_2.jpg 063_cam8.jpg 0.602789**

**004_cam6_2.jpg 038_cam8.jpg 0.257057**

**005_cam6_2.jpg 038_cam8.jpg 0.448558**

**006_cam6_2.jpg 030_cam8.jpg 0.429128**

**007_cam6_2.jpg 049_cam8.jpg 0.649200**

**008_cam6_2.jpg 062_cam8.jpg 0.729978**

**009_cam6_2.jpg 049_cam8.jpg 0.425709**

**010_cam6_2.jpg 013_cam8.jpg 0.287741**

**011_cam6_2.jpg 083_cam8.jpg 0.339870**

**012_cam6_2.jpg 049_cam8.jpg 0.496382**

**013_cam6_2.jpg 037_cam8.jpg 0.408183**

**014_cam6_2.jpg 039_cam8.jpg 0.447691**

**015_cam6_2.jpg 063_cam8.jpg 0.381653**

**016_cam6_2.jpg 063_cam8.jpg 0.487991**

**017_cam6_2.jpg 038_cam8.jpg 0.490525**

**018_cam6_2.jpg 054_cam8.jpg 0.278646**

**019_cam6_2.jpg 129_cam8.jpg 0.401787**

**020_cam6_2.jpg 071_cam8.jpg 0.527246**

**021_cam6_2.jpg 038_cam8.jpg 0.582250**

**022_cam6_2.jpg 045_cam8.jpg 0.615250**

**023_cam6_2.jpg 109_cam8.jpg 0.398683**

**024_cam6_2.jpg 049_cam8.jpg 0.331331**

**025_cam6_2.jpg 012_cam8.jpg 0.274267**

**026_cam6_2.jpg 038_cam8.jpg 0.255934**

**027_cam6_2.jpg 071_cam8.jpg 0.426621**

**028_cam6_2.jpg 063_cam8.jpg 0.534403**

**029_cam6_2.jpg 034_cam8.jpg 0.361052**

**030_cam6_2.jpg 097_cam8.jpg 0.211085**

**031_cam6_2.jpg 079_cam8.jpg 0.455600**

**032_cam6_2.jpg 097_cam8.jpg 0.461932**

**033_cam6_2.jpg 071_cam8.jpg 0.305548**

**034_cam6_2.jpg 071_cam8.jpg 0.331263**

**035_cam6_2.jpg 001_cam8.jpg 0.331783**

**036_cam6_2.jpg 037_cam8.jpg 0.509879**

**037_cam6_2.jpg 037_cam8.jpg 0.563331**

**038_cam6_2.jpg 038_cam8.jpg 0.310600**

**039_cam6_2.jpg 039_cam8.jpg 0.404110**

**040_cam6_2.jpg 049_cam8.jpg 0.352343**

**041_cam6_2.jpg 038_cam8.jpg 0.461886**

**042_cam6_2.jpg 049_cam8.jpg 0.406586**

**043_cam6_2.jpg 043_cam8.jpg 0.339404**

**044_cam6_2.jpg 001_cam8.jpg 0.364682**

**045_cam6_2.jpg 045_cam8.jpg 0.566978**

**046_cam6_2.jpg 083_cam8.jpg 0.375880**

**047_cam6_2.jpg 041_cam8.jpg 0.415023**

**048_cam6_2.jpg 045_cam8.jpg 0.570347**

**049_cam6_2.jpg 049_cam8.jpg 0.481802**

**050_cam6_2.jpg 049_cam8.jpg 0.540281**

**051_cam6_2.jpg 113_cam8.jpg 0.639813**

**052_cam6_2.jpg 049_cam8.jpg 0.393503**

**053_cam6_2.jpg 034_cam8.jpg 0.344022**

**054_cam6_2.jpg 017_cam8.jpg 0.291978**

**055_cam6_2.jpg 079_cam8.jpg 0.371987**

**056_cam6_2.jpg 129_cam8.jpg 0.486222**

**057_cam6_2.jpg 033_cam8.jpg 0.312812**

**058_cam6_2.jpg 079_cam8.jpg 0.609239**

**059_cam6_2.jpg 097_cam8.jpg 0.252420**

**060_cam6_2.jpg 074_cam8.jpg 0.296321**

**061_cam6_2.jpg 060_cam8.jpg 0.427926**

**062_cam6_2.jpg 063_cam8.jpg 0.577809**

**063_cam6_2.jpg 063_cam8.jpg 0.605275**

**064_cam6_2.jpg 030_cam8.jpg 0.382869**

**065_cam6_2.jpg 041_cam8.jpg 0.295451**

**066_cam6_2.jpg 001_cam8.jpg 0.366869**

**067_cam6_2.jpg 049_cam8.jpg 0.333102**

**068_cam6_2.jpg 071_cam8.jpg 0.464121**

**069_cam6_2.jpg 016_cam8.jpg 0.220037**

**070_cam6_2.jpg 001_cam8.jpg 0.375320**

**071_cam6_2.jpg 071_cam8.jpg 0.547097**

**072_cam6_2.jpg 049_cam8.jpg 0.299412**

**073_cam6_2.jpg 038_cam8.jpg 0.439564**

**074_cam6_2.jpg 038_cam8.jpg 0.505303**

**075_cam6_2.jpg 043_cam8.jpg 0.275972**

**076_cam6_2.jpg 083_cam8.jpg 0.385910**

**077_cam6_2.jpg 037_cam8.jpg 0.461813**

**078_cam6_2.jpg 078_cam8.jpg 0.319681**

**079_cam6_2.jpg 038_cam8.jpg 0.345013**

**080_cam6_2.jpg 079_cam8.jpg 0.427069**

**081_cam6_2.jpg 078_cam8.jpg 0.294044**

**082_cam6_2.jpg 038_cam8.jpg 0.487478**

**083_cam6_2.jpg 013_cam8.jpg 0.738436**

**084_cam6_2.jpg 034_cam8.jpg 0.369243**

**085_cam6_2.jpg 063_cam8.jpg 0.559892**

**086_cam6_2.jpg 034_cam8.jpg 0.239678**

**087_cam6_2.jpg 063_cam8.jpg 0.430802**

**088_cam6_2.jpg 012_cam8.jpg 0.431949**

**089_cam6_2.jpg 049_cam8.jpg 0.310482**

**090_cam6_2.jpg 016_cam8.jpg 0.314421**

**091_cam6_2.jpg 041_cam8.jpg 0.375150**

**092_cam6_2.jpg 038_cam8.jpg 0.418154**

**093_cam6_2.jpg 129_cam8.jpg 0.581701**

**094_cam6_2.jpg 013_cam8.jpg 0.444071**

**095_cam6_2.jpg 079_cam8.jpg 0.211425**

**096_cam6_2.jpg 093_cam8.jpg 0.249865**

**097_cam6_2.jpg 097_cam8.jpg 0.440113**

**098_cam6_2.jpg 067_cam8.jpg 0.335940**

**099_cam6_2.jpg 071_cam8.jpg 0.354018**

**100_cam6_2.jpg 028_cam8.jpg 0.493487**

**101_cam6_2.jpg 049_cam8.jpg 0.288660**

**102_cam6_2.jpg 043_cam8.jpg 0.485335**

**103_cam6_2.jpg 038_cam8.jpg 0.367712**

**104_cam6_2.jpg 049_cam8.jpg 0.427589**

**105_cam6_2.jpg 033_cam8.jpg 0.301928**

**106_cam6_2.jpg 001_cam8.jpg 0.354623**

**107_cam6_2.jpg 032_cam8.jpg 0.486115**

**108_cam6_2.jpg 097_cam8.jpg 0.420802**

**109_cam6_2.jpg 109_cam8.jpg 0.280529**

**110_cam6_2.jpg 013_cam8.jpg 0.344419**

**111_cam6_2.jpg 100_cam8.jpg 0.281204**

**112_cam6_2.jpg 049_cam8.jpg 0.273642**

**113_cam6_2.jpg 113_cam8.jpg 0.602230**

**114_cam6_2.jpg 038_cam8.jpg 0.640664**

**115_cam6_2.jpg 063_cam8.jpg 0.390263**

**116_cam6_2.jpg 012_cam8.jpg 0.283878**

**117_cam6_2.jpg 038_cam8.jpg 0.524039**

**118_cam6_2.jpg 041_cam8.jpg 0.471703**

**119_cam6_2.jpg 013_cam8.jpg 0.361625**

**120_cam6_2.jpg 079_cam8.jpg 0.355331**

**121_cam6_2.jpg 104_cam8.jpg 0.398591**

**122_cam6_2.jpg 071_cam8.jpg 0.443646**

**123_cam6_2.jpg 072_cam8.jpg 0.391088**

**124_cam6_2.jpg 006_cam8.jpg 0.289638**

**125_cam6_2.jpg 125_cam8.jpg 0.484253**

**126_cam6_2.jpg 074_cam8.jpg 0.409263**

**127_cam6_2.jpg 037_cam8.jpg 0.428886**

**128_cam6_2.jpg 013_cam8.jpg 0.291435**

**129_cam6_2.jpg 129_cam8.jpg 0.365892**

**130_cam6_2.jpg 038_cam8.jpg 0.331970**

**Final Score Scface SSR (IR) cam6-d3**

**Actual Image Recognized Image Score**

**001_cam6_3.jpg 001_cam8.jpg 0.331234**

**002_cam6_3.jpg 067_cam8.jpg 0.433485**

**003_cam6_3.jpg 072_cam8.jpg 0.349384**

**004_cam6_3.jpg 013_cam8.jpg 0.621759**

**005_cam6_3.jpg 029_cam8.jpg 0.427696**

**006_cam6_3.jpg 072_cam8.jpg 0.583498**

**007_cam6_3.jpg 029_cam8.jpg 0.702523**

**008_cam6_3.jpg 008_cam8.jpg 0.707011**

**009_cam6_3.jpg 120_cam8.jpg 0.340266**

**010_cam6_3.jpg 029_cam8.jpg 0.757957**

**011_cam6_3.jpg 013_cam8.jpg 0.532999**

**012_cam6_3.jpg 049_cam8.jpg 0.322941**

**013_cam6_3.jpg 013_cam8.jpg 0.603562**

**014_cam6_3.jpg 014_cam8.jpg 0.706904**

**015_cam6_3.jpg 013_cam8.jpg 0.373972**

**016_cam6_3.jpg 095_cam8.jpg 0.444621**

**017_cam6_3.jpg 039_cam8.jpg 0.467679**

**018_cam6_3.jpg 013_cam8.jpg 0.441432**

**019_cam6_3.jpg 080_cam8.jpg 0.372033**

**020_cam6_3.jpg 080_cam8.jpg 0.280892**

**021_cam6_3.jpg 013_cam8.jpg 0.518037**

**022_cam6_3.jpg 097_cam8.jpg 0.380501**

**023_cam6_3.jpg 049_cam8.jpg 0.328510**

**024_cam6_3.jpg 119_cam8.jpg 0.472689**

**025_cam6_3.jpg 035_cam8.jpg 0.421896**

**026_cam6_3.jpg 001_cam8.jpg 0.380235**

**027_cam6_3.jpg 072_cam8.jpg 0.364855**

**028_cam6_3.jpg 047_cam8.jpg 0.428787**

**029_cam6_3.jpg 029_cam8.jpg 0.471320**

**030_cam6_3.jpg 013_cam8.jpg 0.441977**

**031_cam6_3.jpg 020_cam8.jpg 0.556063**

**032_cam6_3.jpg 039_cam8.jpg 0.381132**

**033_cam6_3.jpg 013_cam8.jpg 0.570843**

**034_cam6_3.jpg 035_cam8.jpg 0.371987**

**035_cam6_3.jpg 080_cam8.jpg 0.357703**

**036_cam6_3.jpg 013_cam8.jpg 0.412091**

**037_cam6_3.jpg 101_cam8.jpg 0.402617**

**038_cam6_3.jpg 038_cam8.jpg 0.359557**

**039_cam6_3.jpg 029_cam8.jpg 0.547897**

**040_cam6_3.jpg 013_cam8.jpg 0.328999**

**041_cam6_3.jpg 060_cam8.jpg 0.477583**

**042_cam6_3.jpg 067_cam8.jpg 0.270434**

**043_cam6_3.jpg 013_cam8.jpg 0.295999**

**044_cam6_3.jpg 013_cam8.jpg 0.325855**

**045_cam6_3.jpg 045_cam8.jpg 0.747866**

**046_cam6_3.jpg 001_cam8.jpg 0.437040**

**047_cam6_3.jpg 097_cam8.jpg 0.375441**

**048_cam6_3.jpg 048_cam8.jpg 0.521785**

**049_cam6_3.jpg 049_cam8.jpg 0.437395**

**050_cam6_3.jpg 013_cam8.jpg 0.414325**

**051_cam6_3.jpg 074_cam8.jpg 0.463806**

**052_cam6_3.jpg 052_cam8.jpg 0.486649**

**053_cam6_3.jpg 040_cam8.jpg 0.365122**

**054_cam6_3.jpg 013_cam8.jpg 0.601919**

**055_cam6_3.jpg 071_cam8.jpg 0.329972**

**056_cam6_3.jpg 021_cam8.jpg 0.475343**

**057_cam6_3.jpg 021_cam8.jpg 0.503842**

**058_cam6_3.jpg 032_cam8.jpg 0.394819**

**059_cam6_3.jpg 097_cam8.jpg 0.426270**

**060_cam6_3.jpg 038_cam8.jpg 0.356934**

**061_cam6_3.jpg 013_cam8.jpg 0.365879**

**062_cam6_3.jpg 062_cam8.jpg 0.527178**

**063_cam6_3.jpg 063_cam8.jpg 0.496956**

**064_cam6_3.jpg 019_cam8.jpg 0.424606**

**065_cam6_3.jpg 013_cam8.jpg 0.598466**

**066_cam6_3.jpg 079_cam8.jpg 0.382190**

**067_cam6_3.jpg 067_cam8.jpg 0.539821**

**068_cam6_3.jpg 080_cam8.jpg 0.348759**

**069_cam6_3.jpg 034_cam8.jpg 0.354007**

**070_cam6_3.jpg 057_cam8.jpg 0.399810**

**071_cam6_3.jpg 013_cam8.jpg 0.572208**

**072_cam6_3.jpg 013_cam8.jpg 0.428735**

**073_cam6_3.jpg 013_cam8.jpg 0.404082**

**074_cam6_3.jpg 074_cam8.jpg 0.484636**

**075_cam6_3.jpg 020_cam8.jpg 0.553610**

**076_cam6_3.jpg 038_cam8.jpg 0.483798**

**077_cam6_3.jpg 039_cam8.jpg 0.430699**

**078_cam6_3.jpg 078_cam8.jpg 0.362933**

**079_cam6_3.jpg 079_cam8.jpg 0.296980**

**080_cam6_3.jpg 080_cam8.jpg 0.417025**

**081_cam6_3.jpg 039_cam8.jpg 0.347735**

**082_cam6_3.jpg 013_cam8.jpg 0.322357**

**083_cam6_3.jpg 074_cam8.jpg 0.332911**

**084_cam6_3.jpg 013_cam8.jpg 0.441141**

**085_cam6_3.jpg 064_cam8.jpg 0.564981**

**086_cam6_3.jpg 013_cam8.jpg 0.445596**

**087_cam6_3.jpg 013_cam8.jpg 0.515888**

**088_cam6_3.jpg 067_cam8.jpg 0.586350**

**089_cam6_3.jpg 013_cam8.jpg 0.520562**

**090_cam6_3.jpg 042_cam8.jpg 0.387600**

**091_cam6_3.jpg 091_cam8.jpg 0.260456**

**092_cam6_3.jpg 016_cam8.jpg 0.286811**

**093_cam6_3.jpg 005_cam8.jpg 0.361228**

**094_cam6_3.jpg 013_cam8.jpg 0.378125**

**095_cam6_3.jpg 095_cam8.jpg 0.360119**

**096_cam6_3.jpg 013_cam8.jpg 0.488529**

**097_cam6_3.jpg 097_cam8.jpg 0.451224**

**098_cam6_3.jpg 097_cam8.jpg 0.337419**

**099_cam6_3.jpg 013_cam8.jpg 0.372619**

**100_cam6_3.jpg 125_cam8.jpg 0.602612**

**101_cam6_3.jpg 021_cam8.jpg 0.387374**

**102_cam6_3.jpg 072_cam8.jpg 0.404471**

**103_cam6_3.jpg 013_cam8.jpg 0.497475**

**104_cam6_3.jpg 049_cam8.jpg 0.469706**

**105_cam6_3.jpg 105_cam8.jpg 0.418806**

**106_cam6_3.jpg 097_cam8.jpg 0.358220**

**107_cam6_3.jpg 097_cam8.jpg 0.471056**

**108_cam6_3.jpg 001_cam8.jpg 0.440758**

**109_cam6_3.jpg 109_cam8.jpg 0.525026**

**110_cam6_3.jpg 013_cam8.jpg 0.349953**

**111_cam6_3.jpg 093_cam8.jpg 0.339529**

**112_cam6_3.jpg 025_cam8.jpg 0.386925**

**113_cam6_3.jpg 113_cam8.jpg 0.622464**

**114_cam6_3.jpg 013_cam8.jpg 0.357203**

**115_cam6_3.jpg 098_cam8.jpg 0.364168**

**116_cam6_3.jpg 116_cam8.jpg 0.482951**

**117_cam6_3.jpg 110_cam8.jpg 0.381220**

**118_cam6_3.jpg 013_cam8.jpg 0.268736**

**119_cam6_3.jpg 013_cam8.jpg 0.347024**

**120_cam6_3.jpg 120_cam8.jpg 0.346371**

**121_cam6_3.jpg 021_cam8.jpg 0.430327**

**122_cam6_3.jpg 034_cam8.jpg 0.418241**

**123_cam6_3.jpg 039_cam8.jpg 0.381963**

**124_cam6_3.jpg 124_cam8.jpg 0.448763**

**125_cam6_3.jpg 098_cam8.jpg 0.288332**

**126_cam6_3.jpg 060_cam8.jpg 0.629581**

**127_cam6_3.jpg 039_cam8.jpg 0.363763**

**128_cam6_3.jpg 120_cam8.jpg 0.292832**

**129_cam6_3.jpg 125_cam8.jpg 0.491664**

**130_cam6_3.jpg 011_cam8.jpg 0.350339**

**Final Score Scface SSR (IR) cam7-d1**

**Actual Image Recognized Image Score**

**001_cam6_3.jpg 001_cam8.jpg 0.331234**

**002_cam6_3.jpg 067_cam8.jpg 0.433485**

**003_cam6_3.jpg 072_cam8.jpg 0.349384**

**004_cam6_3.jpg 013_cam8.jpg 0.621759**

**005_cam6_3.jpg 029_cam8.jpg 0.427696**

**006_cam6_3.jpg 072_cam8.jpg 0.583498**

**007_cam6_3.jpg 029_cam8.jpg 0.702523**

**008_cam6_3.jpg 008_cam8.jpg 0.707011**

**009_cam6_3.jpg 120_cam8.jpg 0.340266**

**010_cam6_3.jpg 029_cam8.jpg 0.757957**

**011_cam6_3.jpg 013_cam8.jpg 0.532999**

**012_cam6_3.jpg 049_cam8.jpg 0.322941**

**013_cam6_3.jpg 013_cam8.jpg 0.603562**

**014_cam6_3.jpg 014_cam8.jpg 0.706904**

**015_cam6_3.jpg 013_cam8.jpg 0.373972**

**016_cam6_3.jpg 095_cam8.jpg 0.444621**

**017_cam6_3.jpg 039_cam8.jpg 0.467679**

**018_cam6_3.jpg 013_cam8.jpg 0.441432**

**019_cam6_3.jpg 080_cam8.jpg 0.372033**

**020_cam6_3.jpg 080_cam8.jpg 0.280892**

**021_cam6_3.jpg 013_cam8.jpg 0.518037**

**022_cam6_3.jpg 097_cam8.jpg 0.380501**

**023_cam6_3.jpg 049_cam8.jpg 0.328510**

**024_cam6_3.jpg 119_cam8.jpg 0.472689**

**025_cam6_3.jpg 035_cam8.jpg 0.421896**

**026_cam6_3.jpg 001_cam8.jpg 0.380235**

**027_cam6_3.jpg 072_cam8.jpg 0.364855**

**028_cam6_3.jpg 047_cam8.jpg 0.428787**

**029_cam6_3.jpg 029_cam8.jpg 0.471320**

**030_cam6_3.jpg 013_cam8.jpg 0.441977**

**031_cam6_3.jpg 020_cam8.jpg 0.556063**

**032_cam6_3.jpg 039_cam8.jpg 0.381132**

**033_cam6_3.jpg 013_cam8.jpg 0.570843**

**034_cam6_3.jpg 035_cam8.jpg 0.371987**

**035_cam6_3.jpg 080_cam8.jpg 0.357703**

**036_cam6_3.jpg 013_cam8.jpg 0.412091**

**037_cam6_3.jpg 101_cam8.jpg 0.402617**

**038_cam6_3.jpg 038_cam8.jpg 0.359557**

**039_cam6_3.jpg 029_cam8.jpg 0.547897**

**040_cam6_3.jpg 013_cam8.jpg 0.328999**

**041_cam6_3.jpg 060_cam8.jpg 0.477583**

**042_cam6_3.jpg 067_cam8.jpg 0.270434**

**043_cam6_3.jpg 013_cam8.jpg 0.295999**

**044_cam6_3.jpg 013_cam8.jpg 0.325855**

**045_cam6_3.jpg 045_cam8.jpg 0.747866**

**046_cam6_3.jpg 001_cam8.jpg 0.437040**

**047_cam6_3.jpg 097_cam8.jpg 0.375441**

**048_cam6_3.jpg 048_cam8.jpg 0.521785**

**049_cam6_3.jpg 049_cam8.jpg 0.437395**

**050_cam6_3.jpg 013_cam8.jpg 0.414325**

**051_cam6_3.jpg 074_cam8.jpg 0.463806**

**052_cam6_3.jpg 052_cam8.jpg 0.486649**

**053_cam6_3.jpg 040_cam8.jpg 0.365122**

**054_cam6_3.jpg 013_cam8.jpg 0.601919**

**055_cam6_3.jpg 071_cam8.jpg 0.329972**

**056_cam6_3.jpg 021_cam8.jpg 0.475343**

**057_cam6_3.jpg 021_cam8.jpg 0.503842**

**058_cam6_3.jpg 032_cam8.jpg 0.394819**

**059_cam6_3.jpg 097_cam8.jpg 0.426270**

**060_cam6_3.jpg 038_cam8.jpg 0.356934**

**061_cam6_3.jpg 013_cam8.jpg 0.365879**

**062_cam6_3.jpg 062_cam8.jpg 0.527178**

**063_cam6_3.jpg 063_cam8.jpg 0.496956**

**064_cam6_3.jpg 019_cam8.jpg 0.424606**

**065_cam6_3.jpg 013_cam8.jpg 0.598466**

**066_cam6_3.jpg 079_cam8.jpg 0.382190**

**067_cam6_3.jpg 067_cam8.jpg 0.539821**

**068_cam6_3.jpg 080_cam8.jpg 0.348759**

**069_cam6_3.jpg 034_cam8.jpg 0.354007**

**070_cam6_3.jpg 057_cam8.jpg 0.399810**

**071_cam6_3.jpg 013_cam8.jpg 0.572208**

**072_cam6_3.jpg 013_cam8.jpg 0.428735**

**073_cam6_3.jpg 013_cam8.jpg 0.404082**

**074_cam6_3.jpg 074_cam8.jpg 0.484636**

**075_cam6_3.jpg 020_cam8.jpg 0.553610**

**076_cam6_3.jpg 038_cam8.jpg 0.483798**

**077_cam6_3.jpg 039_cam8.jpg 0.430699**

**078_cam6_3.jpg 078_cam8.jpg 0.362933**

**079_cam6_3.jpg 079_cam8.jpg 0.296980**

**080_cam6_3.jpg 080_cam8.jpg 0.417025**

**081_cam6_3.jpg 039_cam8.jpg 0.347735**

**082_cam6_3.jpg 013_cam8.jpg 0.322357**

**083_cam6_3.jpg 074_cam8.jpg 0.332911**

**084_cam6_3.jpg 013_cam8.jpg 0.441141**

**085_cam6_3.jpg 064_cam8.jpg 0.564981**

**086_cam6_3.jpg 013_cam8.jpg 0.445596**

**087_cam6_3.jpg 013_cam8.jpg 0.515888**

**088_cam6_3.jpg 067_cam8.jpg 0.586350**

**089_cam6_3.jpg 013_cam8.jpg 0.520562**

**090_cam6_3.jpg 042_cam8.jpg 0.387600**

**091_cam6_3.jpg 091_cam8.jpg 0.260456**

**092_cam6_3.jpg 016_cam8.jpg 0.286811**

**093_cam6_3.jpg 005_cam8.jpg 0.361228**

**094_cam6_3.jpg 013_cam8.jpg 0.378125**

**095_cam6_3.jpg 095_cam8.jpg 0.360119**

**096_cam6_3.jpg 013_cam8.jpg 0.488529**

**097_cam6_3.jpg 097_cam8.jpg 0.451224**

**098_cam6_3.jpg 097_cam8.jpg 0.337419**

**099_cam6_3.jpg 013_cam8.jpg 0.372619**

**100_cam6_3.jpg 125_cam8.jpg 0.602612**

**101_cam6_3.jpg 021_cam8.jpg 0.387374**

**102_cam6_3.jpg 072_cam8.jpg 0.404471**

**103_cam6_3.jpg 013_cam8.jpg 0.497475**

**104_cam6_3.jpg 049_cam8.jpg 0.469706**

**105_cam6_3.jpg 105_cam8.jpg 0.418806**

**106_cam6_3.jpg 097_cam8.jpg 0.358220**

**107_cam6_3.jpg 097_cam8.jpg 0.471056**

**108_cam6_3.jpg 001_cam8.jpg 0.440758**

**109_cam6_3.jpg 109_cam8.jpg 0.525026**

**110_cam6_3.jpg 013_cam8.jpg 0.349953**

**111_cam6_3.jpg 093_cam8.jpg 0.339529**

**112_cam6_3.jpg 025_cam8.jpg 0.386925**

**113_cam6_3.jpg 113_cam8.jpg 0.622464**

**114_cam6_3.jpg 013_cam8.jpg 0.357203**

**115_cam6_3.jpg 098_cam8.jpg 0.364168**

**116_cam6_3.jpg 116_cam8.jpg 0.482951**

**117_cam6_3.jpg 110_cam8.jpg 0.381220**

**118_cam6_3.jpg 013_cam8.jpg 0.268736**

**119_cam6_3.jpg 013_cam8.jpg 0.347024**

**120_cam6_3.jpg 120_cam8.jpg 0.346371**

**121_cam6_3.jpg 021_cam8.jpg 0.430327**

**122_cam6_3.jpg 034_cam8.jpg 0.418241**

**123_cam6_3.jpg 039_cam8.jpg 0.381963**

**124_cam6_3.jpg 124_cam8.jpg 0.448763**

**125_cam6_3.jpg 098_cam8.jpg 0.288332**

**126_cam6_3.jpg 060_cam8.jpg 0.629581**

**127_cam6_3.jpg 039_cam8.jpg 0.363763**

**128_cam6_3.jpg 120_cam8.jpg 0.292832**

**129_cam6_3.jpg 125_cam8.jpg 0.491664**

**130_cam6_3.jpg 011_cam8.jpg 0.350339**

**Final Score Scface SSR (IR) cam7-d2**

**Actual Image Recognized Image Score**

**001_cam7_2.jpg 013_cam8.jpg 0.686619**

**002_cam7_2.jpg 075_cam8.jpg 0.405038**

**003_cam7_2.jpg 013_cam8.jpg 0.754215**

**004_cam7_2.jpg 080_cam8.jpg 0.531626**

**005_cam7_2.jpg 109_cam8.jpg 0.623141**

**006_cam7_2.jpg 123_cam8.jpg 0.526770**

**007_cam7_2.jpg 074_cam8.jpg 0.655493**

**008_cam7_2.jpg 008_cam8.jpg 0.657478**

**009_cam7_2.jpg 029_cam8.jpg 0.605071**

**010_cam7_2.jpg 095_cam8.jpg 0.360676**

**011_cam7_2.jpg 013_cam8.jpg 0.598252**

**012_cam7_2.jpg 019_cam8.jpg 0.434974**

**013_cam7_2.jpg 013_cam8.jpg 0.749627**

**014_cam7_2.jpg 014_cam8.jpg 0.645016**

**015_cam7_2.jpg 103_cam8.jpg 0.484638**

**016_cam7_2.jpg 075_cam8.jpg 0.508758**

**017_cam7_2.jpg 039_cam8.jpg 0.480890**

**018_cam7_2.jpg 013_cam8.jpg 0.693616**

**019_cam7_2.jpg 086_cam8.jpg 0.583480**

**020_cam7_2.jpg 034_cam8.jpg 0.612871**

**021_cam7_2.jpg 013_cam8.jpg 0.471984**

**022_cam7_2.jpg 073_cam8.jpg 0.410363**

**023_cam7_2.jpg 031_cam8.jpg 0.491939**

**024_cam7_2.jpg 120_cam8.jpg 0.381365**

**025_cam7_2.jpg 011_cam8.jpg 0.415973**

**026_cam7_2.jpg 039_cam8.jpg 0.474607**

**027_cam7_2.jpg 013_cam8.jpg 0.671469**

**028_cam7_2.jpg 124_cam8.jpg 0.455218**

**029_cam7_2.jpg 035_cam8.jpg 0.453173**

**030_cam7_2.jpg 013_cam8.jpg 0.667160**

**031_cam7_2.jpg 047_cam8.jpg 0.520248**

**032_cam7_2.jpg 011_cam8.jpg 0.433972**

**033_cam7_2.jpg 013_cam8.jpg 0.920608**

**034_cam7_2.jpg 005_cam8.jpg 0.364564**

**035_cam7_2.jpg 017_cam8.jpg 0.384974**

**036_cam7_2.jpg 034_cam8.jpg 0.397403**

**037_cam7_2.jpg 097_cam8.jpg 0.675412**

**038_cam7_2.jpg 013_cam8.jpg 0.402582**

**039_cam7_2.jpg 039_cam8.jpg 0.619388**

**040_cam7_2.jpg 019_cam8.jpg 0.527070**

**041_cam7_2.jpg 040_cam8.jpg 0.464083**

**042_cam7_2.jpg 013_cam8.jpg 0.672841**

**043_cam7_2.jpg 083_cam8.jpg 0.416550**

**044_cam7_2.jpg 130_cam8.jpg 0.644794**

**045_cam7_2.jpg 045_cam8.jpg 0.471742**

**046_cam7_2.jpg 047_cam8.jpg 0.411143**

**047_cam7_2.jpg 047_cam8.jpg 0.534081**

**048_cam7_2.jpg 013_cam8.jpg 0.733754**

**049_cam7_2.jpg 060_cam8.jpg 0.532140**

**050_cam7_2.jpg 123_cam8.jpg 0.428102**

**051_cam7_2.jpg 094_cam8.jpg 0.842909**

**052_cam7_2.jpg 013_cam8.jpg 0.665207**

**053_cam7_2.jpg 067_cam8.jpg 0.506796**

**054_cam7_2.jpg 013_cam8.jpg 0.501784**

**055_cam7_2.jpg 013_cam8.jpg 0.617446**

**056_cam7_2.jpg 086_cam8.jpg 0.595632**

**057_cam7_2.jpg 097_cam8.jpg 0.612930**

**058_cam7_2.jpg 039_cam8.jpg 0.573880**

**059_cam7_2.jpg 013_cam8.jpg 0.509896**

**060_cam7_2.jpg 040_cam8.jpg 0.565511**

**061_cam7_2.jpg 091_cam8.jpg 0.461514**

**062_cam7_2.jpg 126_cam8.jpg 0.369230**

**063_cam7_2.jpg 037_cam8.jpg 0.493105**

**064_cam7_2.jpg 013_cam8.jpg 0.710132**

**065_cam7_2.jpg 013_cam8.jpg 0.723193**

**066_cam7_2.jpg 117_cam8.jpg 0.433877**

**067_cam7_2.jpg 013_cam8.jpg 0.627873**

**068_cam7_2.jpg 067_cam8.jpg 0.499675**

**069_cam7_2.jpg 097_cam8.jpg 0.410693**

**070_cam7_2.jpg 013_cam8.jpg 0.671830**

**071_cam7_2.jpg 039_cam8.jpg 0.722462**

**072_cam7_2.jpg 120_cam8.jpg 0.361819**

**073_cam7_2.jpg 086_cam8.jpg 0.575727**

**074_cam7_2.jpg 124_cam8.jpg 0.493861**

**075_cam7_2.jpg 075_cam8.jpg 0.423788**

**076_cam7_2.jpg 086_cam8.jpg 0.429660**

**077_cam7_2.jpg 019_cam8.jpg 0.385275**

**078_cam7_2.jpg 078_cam8.jpg 0.629018**

**079_cam7_2.jpg 019_cam8.jpg 0.373583**

**080_cam7_2.jpg 080_cam8.jpg 0.402004**

**081_cam7_2.jpg 039_cam8.jpg 0.500545**

**082_cam7_2.jpg 078_cam8.jpg 0.503855**

**083_cam7_2.jpg 013_cam8.jpg 0.495511**

**084_cam7_2.jpg 080_cam8.jpg 0.497245**

**085_cam7_2.jpg 019_cam8.jpg 0.410016**

**086_cam7_2.jpg 013_cam8.jpg 0.696550**

**087_cam7_2.jpg 013_cam8.jpg 0.597863**

**088_cam7_2.jpg 080_cam8.jpg 0.451471**

**089_cam7_2.jpg 053_cam8.jpg 0.366860**

**090_cam7_2.jpg 080_cam8.jpg 0.530120**

**091_cam7_2.jpg 013_cam8.jpg 0.551424**

**092_cam7_2.jpg 116_cam8.jpg 0.322080**

**093_cam7_2.jpg 068_cam8.jpg 0.555526**

**094_cam7_2.jpg 013_cam8.jpg 0.538247**

**095_cam7_2.jpg 013_cam8.jpg 0.543478**

**096_cam7_2.jpg 120_cam8.jpg 0.460250**

**097_cam7_2.jpg 097_cam8.jpg 0.418013**

**098_cam7_2.jpg 013_cam8.jpg 0.632369**

**099_cam7_2.jpg 086_cam8.jpg 0.527926**

**100_cam7_2.jpg 013_cam8.jpg 0.548673**

**101_cam7_2.jpg 047_cam8.jpg 0.541289**

**102_cam7_2.jpg 047_cam8.jpg 0.516217**

**103_cam7_2.jpg 013_cam8.jpg 0.712652**

**104_cam7_2.jpg 104_cam8.jpg 0.639382**

**105_cam7_2.jpg 013_cam8.jpg 0.741906**

**106_cam7_2.jpg 034_cam8.jpg 0.566166**

**107_cam7_2.jpg 108_cam8.jpg 0.383239**

**108_cam7_2.jpg 039_cam8.jpg 0.491850**

**109_cam7_2.jpg 039_cam8.jpg 0.578602**

**110_cam7_2.jpg 080_cam8.jpg 0.559125**

**111_cam7_2.jpg 068_cam8.jpg 0.312035**

**112_cam7_2.jpg 086_cam8.jpg 0.445911**

**113_cam7_2.jpg 025_cam8.jpg 0.526270**

**114_cam7_2.jpg 097_cam8.jpg 0.361598**

**115_cam7_2.jpg 033_cam8.jpg 0.388873**

**116_cam7_2.jpg 038_cam8.jpg 0.428592**

**117_cam7_2.jpg 120_cam8.jpg 0.499874**

**118_cam7_2.jpg 120_cam8.jpg 0.451380**

**119_cam7_2.jpg 060_cam8.jpg 0.464420**

**120_cam7_2.jpg 120_cam8.jpg 0.407426**

**121_cam7_2.jpg 056_cam8.jpg 0.336724**

**122_cam7_2.jpg 039_cam8.jpg 0.540170**

**123_cam7_2.jpg 039_cam8.jpg 0.625280**

**124_cam7_2.jpg 013_cam8.jpg 0.537548**

**125_cam7_2.jpg 037_cam8.jpg 0.629729**

**126_cam7_2.jpg 035_cam8.jpg 0.401298**

**127_cam7_2.jpg 013_cam8.jpg 0.613007**

**128_cam7_2.jpg 013_cam8.jpg 0.622688**

**129_cam7_2.jpg 019_cam8.jpg 0.364853**

**130_cam7_2.jpg 100_cam8.jpg 0.515559**

**Final Score Scface SSR (IR) cam7-d3**

**Actual Image Recognized Image Score**

**001_cam7_3.jpg 097_cam8.jpg 0.525473**

**002_cam7_3.jpg 021_cam8.jpg 0.478600**

**003_cam7_3.jpg 049_cam8.jpg 0.529696**

**004_cam7_3.jpg 013_cam8.jpg 0.523241**

**005_cam7_3.jpg 079_cam8.jpg 0.763433**

**006_cam7_3.jpg 072_cam8.jpg 0.607811**

**007_cam7_3.jpg 029_cam8.jpg 0.576685**

**008_cam7_3.jpg 013_cam8.jpg 0.771162**

**009_cam7_3.jpg 067_cam8.jpg 0.525202**

**010_cam7_3.jpg 013_cam8.jpg 0.826130**

**011_cam7_3.jpg 013_cam8.jpg 0.527639**

**012_cam7_3.jpg 072_cam8.jpg 0.490260**

**013_cam7_3.jpg 013_cam8.jpg 0.642881**

**014_cam7_3.jpg 085_cam8.jpg 0.490588**

**015_cam7_3.jpg 008_cam8.jpg 0.455350**

**016_cam7_3.jpg 021_cam8.jpg 0.513676**

**017_cam7_3.jpg 013_cam8.jpg 0.567869**

**018_cam7_3.jpg 013_cam8.jpg 0.655770**

**019_cam7_3.jpg 061_cam8.jpg 0.385432**

**020_cam7_3.jpg 029_cam8.jpg 0.572957**

**021_cam7_3.jpg 039_cam8.jpg 0.558481**

**022_cam7_3.jpg 013_cam8.jpg 0.617962**

**023_cam7_3.jpg 053_cam8.jpg 0.718952**

**024_cam7_3.jpg 120_cam8.jpg 0.482462**

**025_cam7_3.jpg 108_cam8.jpg 0.607179**

**026_cam7_3.jpg 068_cam8.jpg 0.674105**

**027_cam7_3.jpg 116_cam8.jpg 0.552045**

**028_cam7_3.jpg 013_cam8.jpg 0.741327**

**029_cam7_3.jpg 013_cam8.jpg 0.634703**

**030_cam7_3.jpg 041_cam8.jpg 0.763333**

**031_cam7_3.jpg 013_cam8.jpg 0.697999**

**032_cam7_3.jpg 071_cam8.jpg 0.571126**

**033_cam7_3.jpg 114_cam8.jpg 0.583666**

**034_cam7_3.jpg 013_cam8.jpg 0.645457**

**035_cam7_3.jpg 098_cam8.jpg 0.541109**

**036_cam7_3.jpg 117_cam8.jpg 0.535169**

**037_cam7_3.jpg 064_cam8.jpg 0.516497**

**038_cam7_3.jpg 063_cam8.jpg 0.425073**

**039_cam7_3.jpg 029_cam8.jpg 0.568422**

**040_cam7_3.jpg 067_cam8.jpg 0.458877**

**041_cam7_3.jpg 072_cam8.jpg 0.437609**

**042_cam7_3.jpg 013_cam8.jpg 0.609941**

**043_cam7_3.jpg 013_cam8.jpg 0.387667**

**044_cam7_3.jpg 097_cam8.jpg 0.471696**

**045_cam7_3.jpg 045_cam8.jpg 0.647526**

**046_cam7_3.jpg 025_cam8.jpg 0.677884**

**047_cam7_3.jpg 079_cam8.jpg 0.504164**

**048_cam7_3.jpg 047_cam8.jpg 0.720672**

**049_cam7_3.jpg 013_cam8.jpg 0.422603**

**050_cam7_3.jpg 021_cam8.jpg 0.621837**

**051_cam7_3.jpg 113_cam8.jpg 0.424462**

**052_cam7_3.jpg 013_cam8.jpg 0.455371**

**053_cam7_3.jpg 013_cam8.jpg 0.643852**

**054_cam7_3.jpg 080_cam8.jpg 0.682934**

**055_cam7_3.jpg 071_cam8.jpg 0.437925**

**056_cam7_3.jpg 013_cam8.jpg 0.963167**

**057_cam7_3.jpg 126_cam8.jpg 0.510577**

**058_cam7_3.jpg 082_cam8.jpg 0.558089**

**059_cam7_3.jpg 123_cam8.jpg 0.450118**

**060_cam7_3.jpg 028_cam8.jpg 0.390359**

**061_cam7_3.jpg 097_cam8.jpg 0.545598**

**062_cam7_3.jpg 034_cam8.jpg 0.506230**

**063_cam7_3.jpg 063_cam8.jpg 0.547393**

**064_cam7_3.jpg 120_cam8.jpg 0.448661**

**065_cam7_3.jpg 013_cam8.jpg 0.760446**

**066_cam7_3.jpg 123_cam8.jpg 0.640232**

**067_cam7_3.jpg 083_cam8.jpg 0.441486**

**068_cam7_3.jpg 120_cam8.jpg 0.575562**

**069_cam7_3.jpg 120_cam8.jpg 0.522446**

**070_cam7_3.jpg 108_cam8.jpg 0.501675**

**071_cam7_3.jpg 074_cam8.jpg 0.493402**

**072_cam7_3.jpg 001_cam8.jpg 0.481962**

**073_cam7_3.jpg 013_cam8.jpg 0.636292**

**074_cam7_3.jpg 013_cam8.jpg 0.959882**

**075_cam7_3.jpg 109_cam8.jpg 0.663387**

**076_cam7_3.jpg 120_cam8.jpg 0.563788**

**077_cam7_3.jpg 013_cam8.jpg 1.020287**

**078_cam7_3.jpg 078_cam8.jpg 0.695417**

**079_cam7_3.jpg 123_cam8.jpg 0.383880**

**080_cam7_3.jpg 013_cam8.jpg 0.439601**

**081_cam7_3.jpg 013_cam8.jpg 0.671447**

**082_cam7_3.jpg 013_cam8.jpg 0.939421**

**083_cam7_3.jpg 120_cam8.jpg 0.472426**

**084_cam7_3.jpg 013_cam8.jpg 0.583673**

**085_cam7_3.jpg 104_cam8.jpg 0.500699**

**086_cam7_3.jpg 013_cam8.jpg 0.740846**

**087_cam7_3.jpg 006_cam8.jpg 0.648724**

**088_cam7_3.jpg 012_cam8.jpg 0.558608**

**089_cam7_3.jpg 124_cam8.jpg 0.466679**

**090_cam7_3.jpg 024_cam8.jpg 0.439069**

**091_cam7_3.jpg 013_cam8.jpg 0.579454**

**092_cam7_3.jpg 101_cam8.jpg 0.462668**

**093_cam7_3.jpg 114_cam8.jpg 0.436602**

**094_cam7_3.jpg 013_cam8.jpg 0.626099**

**095_cam7_3.jpg 029_cam8.jpg 0.545265**

**096_cam7_3.jpg 042_cam8.jpg 0.500384**

**097_cam7_3.jpg 053_cam8.jpg 0.386731**

**098_cam7_3.jpg 024_cam8.jpg 0.628192**

**099_cam7_3.jpg 021_cam8.jpg 0.632626**

**100_cam7_3.jpg 071_cam8.jpg 0.565761**

**101_cam7_3.jpg 013_cam8.jpg 0.497187**

**102_cam7_3.jpg 025_cam8.jpg 0.474719**

**103_cam7_3.jpg 041_cam8.jpg 0.505131**

**104_cam7_3.jpg 013_cam8.jpg 0.556478**

**105_cam7_3.jpg 053_cam8.jpg 0.472345**

**106_cam7_3.jpg 013_cam8.jpg 0.510697**

**107_cam7_3.jpg 097_cam8.jpg 0.565370**

**108_cam7_3.jpg 021_cam8.jpg 0.431448**

**109_cam7_3.jpg 078_cam8.jpg 0.387745**

**110_cam7_3.jpg 025_cam8.jpg 0.353746**

**111_cam7_3.jpg 013_cam8.jpg 0.454547**

**112_cam7_3.jpg 112_cam8.jpg 0.495290**

**113_cam7_3.jpg 113_cam8.jpg 0.545381**

**114_cam7_3.jpg 013_cam8.jpg 0.407265**

**115_cam7_3.jpg 115_cam8.jpg 0.413996**

**116_cam7_3.jpg 123_cam8.jpg 0.564433**

**117_cam7_3.jpg 013_cam8.jpg 0.683484**

**118_cam7_3.jpg 082_cam8.jpg 0.393835**

**119_cam7_3.jpg 072_cam8.jpg 0.511677**

**120_cam7_3.jpg 104_cam8.jpg 0.498673**

**121_cam7_3.jpg 042_cam8.jpg 0.583779**

**122_cam7_3.jpg 080_cam8.jpg 0.416086**

**123_cam7_3.jpg 013_cam8.jpg 0.608797**

**124_cam7_3.jpg 105_cam8.jpg 0.589043**

**125_cam7_3.jpg 039_cam8.jpg 0.571091**

**126_cam7_3.jpg 124_cam8.jpg 0.424416**

**127_cam7_3.jpg 080_cam8.jpg 0.467128**

**128_cam7_3.jpg 080_cam8.jpg 0.632939**

**129_cam7_3.jpg 116_cam8.jpg 0.350580**

**130_cam7_3.jpg 073_cam8.jpg 0.498105**

**Final Score Scface SSR (Vis) cam1-d1**

**Actual Image Recognized Image Score**

**001_cam1_1.jpg 096_frontal.jpg 0.478759**

**002_cam1_1.jpg 114_frontal.jpg 0.630452**

**003_cam1_1.jpg 042_frontal.jpg 0.322195**

**004_cam1_1.jpg 014_frontal.jpg 0.332253**

**005_cam1_1.jpg 005_frontal.jpg 0.487899**

**006_cam1_1.jpg 089_frontal.jpg 0.485658**

**007_cam1_1.jpg 044_frontal.jpg 0.582686**

**008_cam1_1.jpg 063_frontal.jpg 0.620805**

**009_cam1_1.jpg 047_frontal.jpg 0.302377**

**010_cam1_1.jpg 015_frontal.jpg 0.487984**

**011_cam1_1.jpg 126_frontal.jpg 0.499415**

**012_cam1_1.jpg 012_frontal.jpg 0.483268**

**013_cam1_1.jpg 115_frontal.jpg 0.625978**

**014_cam1_1.jpg 117_frontal.jpg 0.543967**

**015_cam1_1.jpg 099_frontal.jpg 0.506270**

**016_cam1_1.jpg 059_frontal.jpg 0.622255**

**017_cam1_1.jpg 042_frontal.jpg 0.500653**

**018_cam1_1.jpg 050_frontal.jpg 0.357593**

**019_cam1_1.jpg 096_frontal.jpg 0.464086**

**020_cam1_1.jpg 115_frontal.jpg 0.418231**

**021_cam1_1.jpg 015_frontal.jpg 0.420445**

**022_cam1_1.jpg 093_frontal.jpg 0.454582**

**023_cam1_1.jpg 117_frontal.jpg 0.413816**

**024_cam1_1.jpg 049_frontal.jpg 0.565226**

**025_cam1_1.jpg 012_frontal.jpg 0.413517**

**026_cam1_1.jpg 096_frontal.jpg 0.524360**

**027_cam1_1.jpg 027_frontal.jpg 0.635024**

**028_cam1_1.jpg 005_frontal.jpg 0.579594**

**029_cam1_1.jpg 126_frontal.jpg 0.676563**

**030_cam1_1.jpg 027_frontal.jpg 0.486560**

**031_cam1_1.jpg 039_frontal.jpg 0.440345**

**032_cam1_1.jpg 096_frontal.jpg 0.515115**

**033_cam1_1.jpg 012_frontal.jpg 0.606574**

**034_cam1_1.jpg 016_frontal.jpg 0.351506**

**035_cam1_1.jpg 115_frontal.jpg 0.669694**

**036_cam1_1.jpg 096_frontal.jpg 0.555388**

**037_cam1_1.jpg 027_frontal.jpg 0.665319**

**038_cam1_1.jpg 096_frontal.jpg 0.483587**

**039_cam1_1.jpg 005_frontal.jpg 0.363436**

**040_cam1_1.jpg 027_frontal.jpg 0.502121**

**041_cam1_1.jpg 042_frontal.jpg 0.577204**

**042_cam1_1.jpg 096_frontal.jpg 0.648779**

**043_cam1_1.jpg 120_frontal.jpg 0.579724**

**044_cam1_1.jpg 042_frontal.jpg 0.342687**

**045_cam1_1.jpg 045_frontal.jpg 0.465184**

**046_cam1_1.jpg 096_frontal.jpg 0.380320**

**047_cam1_1.jpg 099_frontal.jpg 0.399635**

**048_cam1_1.jpg 048_frontal.jpg 0.533521**

**049_cam1_1.jpg 049_frontal.jpg 0.460765**

**050_cam1_1.jpg 120_frontal.jpg 0.409202**

**051_cam1_1.jpg 033_frontal.jpg 0.565823**

**052_cam1_1.jpg 096_frontal.jpg 0.520697**

**053_cam1_1.jpg 096_frontal.jpg 0.460650**

**054_cam1_1.jpg 005_frontal.jpg 0.614421**

**055_cam1_1.jpg 126_frontal.jpg 0.578979**

**056_cam1_1.jpg 036_frontal.jpg 0.463831**

**057_cam1_1.jpg 027_frontal.jpg 0.786924**

**058_cam1_1.jpg 096_frontal.jpg 0.704360**

**059_cam1_1.jpg 096_frontal.jpg 0.627993**

**060_cam1_1.jpg 042_frontal.jpg 0.765856**

**061_cam1_1.jpg 088_frontal.jpg 0.222860**

**062_cam1_1.jpg 012_frontal.jpg 0.516349**

**063_cam1_1.jpg 096_frontal.jpg 0.504382**

**064_cam1_1.jpg 027_frontal.jpg 0.765665**

**065_cam1_1.jpg 101_frontal.jpg 0.394768**

**066_cam1_1.jpg 115_frontal.jpg 0.609444**

**067_cam1_1.jpg 088_frontal.jpg 0.639115**

**068_cam1_1.jpg 068_frontal.jpg 0.814257**

**069_cam1_1.jpg 096_frontal.jpg 0.486283**

**070_cam1_1.jpg 042_frontal.jpg 0.290961**

**071_cam1_1.jpg 051_frontal.jpg 0.454776**

**072_cam1_1.jpg 115_frontal.jpg 0.484440**

**073_cam1_1.jpg 027_frontal.jpg 0.589367**

**074_cam1_1.jpg 126_frontal.jpg 0.373712**

**075_cam1_1.jpg 005_frontal.jpg 0.334711**

**076_cam1_1.jpg 042_frontal.jpg 0.604681**

**077_cam1_1.jpg 096_frontal.jpg 0.582739**

**078_cam1_1.jpg 101_frontal.jpg 0.424617**

**079_cam1_1.jpg 088_frontal.jpg 0.335356**

**080_cam1_1.jpg 042_frontal.jpg 0.388464**

**081_cam1_1.jpg 099_frontal.jpg 0.549982**

**082_cam1_1.jpg 126_frontal.jpg 0.417108**

**083_cam1_1.jpg 027_frontal.jpg 0.549073**

**084_cam1_1.jpg 005_frontal.jpg 0.371172**

**085_cam1_1.jpg 059_frontal.jpg 0.619496**

**086_cam1_1.jpg 012_frontal.jpg 0.550972**

**087_cam1_1.jpg 096_frontal.jpg 0.455909**

**088_cam1_1.jpg 112_frontal.jpg 0.408876**

**089_cam1_1.jpg 115_frontal.jpg 0.489487**

**090_cam1_1.jpg 047_frontal.jpg 0.224532**

**091_cam1_1.jpg 027_frontal.jpg 0.500801**

**092_cam1_1.jpg 101_frontal.jpg 0.491466**

**093_cam1_1.jpg 059_frontal.jpg 0.661924**

**094_cam1_1.jpg 027_frontal.jpg 0.542903**

**095_cam1_1.jpg 099_frontal.jpg 0.655481**

**096_cam1_1.jpg 115_frontal.jpg 0.433267**

**097_cam1_1.jpg 032_frontal.jpg 0.627122**

**098_cam1_1.jpg 049_frontal.jpg 0.697290**

**099_cam1_1.jpg 027_frontal.jpg 0.424957**

**100_cam1_1.jpg 097_frontal.jpg 0.582933**

**101_cam1_1.jpg 115_frontal.jpg 0.570749**

**102_cam1_1.jpg 027_frontal.jpg 0.588235**

**103_cam1_1.jpg 027_frontal.jpg 0.430694**

**104_cam1_1.jpg 049_frontal.jpg 0.459550**

**105_cam1_1.jpg 027_frontal.jpg 0.331960**

**106_cam1_1.jpg 096_frontal.jpg 0.493280**

**107_cam1_1.jpg 115_frontal.jpg 0.432147**

**108_cam1_1.jpg 049_frontal.jpg 0.490279**

**109_cam1_1.jpg 109_frontal.jpg 0.544085**

**110_cam1_1.jpg 126_frontal.jpg 0.814621**

**111_cam1_1.jpg 027_frontal.jpg 0.671775**

**112_cam1_1.jpg 112_frontal.jpg 0.531516**

**113_cam1_1.jpg 025_frontal.jpg 0.396371**

**114_cam1_1.jpg 005_frontal.jpg 0.425419**

**115_cam1_1.jpg 115_frontal.jpg 0.830934**

**116_cam1_1.jpg 049_frontal.jpg 0.381821**

**117_cam1_1.jpg 115_frontal.jpg 0.470595**

**118_cam1_1.jpg 115_frontal.jpg 0.454512**

**119_cam1_1.jpg 096_frontal.jpg 0.647152**

**120_cam1_1.jpg 096_frontal.jpg 0.606921**

**121_cam1_1.jpg 027_frontal.jpg 0.504004**

**122_cam1_1.jpg 126_frontal.jpg 0.399984**

**123_cam1_1.jpg 027_frontal.jpg 0.905059**

**124_cam1_1.jpg 101_frontal.jpg 0.310337**

**125_cam1_1.jpg 042_frontal.jpg 0.403063**

**126_cam1_1.jpg 126_frontal.jpg 0.683507**

**127_cam1_1.jpg 059_frontal.jpg 0.423142**

**128_cam1_1.jpg 027_frontal.jpg 0.553761**

**129_cam1_1.jpg 059_frontal.jpg 0.517078**

**130_cam1_1.jpg 025_frontal.jpg 0.443865**

**Final Score Scface SSR (Vis) cam1-d2**

**Actual Image Recognized Image Score**

**001_cam1_2.jpg 042_frontal.jpg 0.285070**

**002_cam1_2.jpg 071_frontal.jpg 0.387793**

**003_cam1_2.jpg 057_frontal.jpg 0.385091**

**004_cam1_2.jpg 049_frontal.jpg 0.318491**

**005_cam1_2.jpg 062_frontal.jpg 0.510769**

**006_cam1_2.jpg 013_frontal.jpg 0.491768**

**007_cam1_2.jpg 027_frontal.jpg 0.377000**

**008_cam1_2.jpg 008_frontal.jpg 0.914454**

**009_cam1_2.jpg 042_frontal.jpg 0.428825**

**010_cam1_2.jpg 115_frontal.jpg 0.256552**

**011_cam1_2.jpg 059_frontal.jpg 0.389381**

**012_cam1_2.jpg 112_frontal.jpg 0.379372**

**013_cam1_2.jpg 013_frontal.jpg 0.543827**

**014_cam1_2.jpg 014_frontal.jpg 0.669069**

**015_cam1_2.jpg 042_frontal.jpg 0.646004**

**016_cam1_2.jpg 016_frontal.jpg 0.602280**

**017_cam1_2.jpg 015_frontal.jpg 0.532451**

**018_cam1_2.jpg 053_frontal.jpg 0.330681**

**019_cam1_2.jpg 042_frontal.jpg 0.252550**

**020_cam1_2.jpg 049_frontal.jpg 0.470714**

**021_cam1_2.jpg 015_frontal.jpg 0.517484**

**022_cam1_2.jpg 022_frontal.jpg 0.374001**

**023_cam1_2.jpg 053_frontal.jpg 0.281405**

**024_cam1_2.jpg 112_frontal.jpg 0.270926**

**025_cam1_2.jpg 025_frontal.jpg 0.434908**

**026_cam1_2.jpg 062_frontal.jpg 0.463341**

**027_cam1_2.jpg 027_frontal.jpg 0.556432**

**028_cam1_2.jpg 115_frontal.jpg 0.340181**

**029_cam1_2.jpg 062_frontal.jpg 0.478222**

**030_cam1_2.jpg 013_frontal.jpg 0.360137**

**031_cam1_2.jpg 062_frontal.jpg 0.560653**

**032_cam1_2.jpg 032_frontal.jpg 0.371850**

**033_cam1_2.jpg 115_frontal.jpg 0.533251**

**034_cam1_2.jpg 030_frontal.jpg 0.405279**

**035_cam1_2.jpg 115_frontal.jpg 0.319718**

**036_cam1_2.jpg 012_frontal.jpg 0.443187**

**037_cam1_2.jpg 037_frontal.jpg 0.389894**

**038_cam1_2.jpg 096_frontal.jpg 0.393783**

**039_cam1_2.jpg 049_frontal.jpg 0.312741**

**040_cam1_2.jpg 049_frontal.jpg 0.442562**

**041_cam1_2.jpg 016_frontal.jpg 0.362136**

**042_cam1_2.jpg 042_frontal.jpg 0.379864**

**043_cam1_2.jpg 115_frontal.jpg 0.338048**

**044_cam1_2.jpg 044_frontal.jpg 0.440250**

**045_cam1_2.jpg 045_frontal.jpg 0.448121**

**046_cam1_2.jpg 117_frontal.jpg 0.324981**

**047_cam1_2.jpg 011_frontal.jpg 0.357436**

**048_cam1_2.jpg 048_frontal.jpg 0.372230**

**049_cam1_2.jpg 049_frontal.jpg 0.526838**

**050_cam1_2.jpg 057_frontal.jpg 0.237261**

**051_cam1_2.jpg 059_frontal.jpg 0.478554**

**052_cam1_2.jpg 071_frontal.jpg 0.401007**

**053_cam1_2.jpg 053_frontal.jpg 0.507644**

**054_cam1_2.jpg 112_frontal.jpg 0.339782**

**055_cam1_2.jpg 016_frontal.jpg 0.310595**

**056_cam1_2.jpg 056_frontal.jpg 0.397348**

**057_cam1_2.jpg 030_frontal.jpg 0.408186**

**058_cam1_2.jpg 016_frontal.jpg 0.411071**

**059_cam1_2.jpg 053_frontal.jpg 0.435055**

**060_cam1_2.jpg 012_frontal.jpg 0.461454**

**061_cam1_2.jpg 042_frontal.jpg 0.290504**

**062_cam1_2.jpg 062_frontal.jpg 0.666067**

**063_cam1_2.jpg 063_frontal.jpg 0.712740**

**064_cam1_2.jpg 112_frontal.jpg 0.436947**

**065_cam1_2.jpg 065_frontal.jpg 0.374107**

**066_cam1_2.jpg 066_frontal.jpg 0.387964**

**067_cam1_2.jpg 067_frontal.jpg 0.395253**

**068_cam1_2.jpg 068_frontal.jpg 0.570006**

**069_cam1_2.jpg 019_frontal.jpg 0.343795**

**070_cam1_2.jpg 112_frontal.jpg 0.305143**

**071_cam1_2.jpg 071_frontal.jpg 0.354311**

**072_cam1_2.jpg 096_frontal.jpg 0.385902**

**073_cam1_2.jpg 049_frontal.jpg 0.444261**

**074_cam1_2.jpg 074_frontal.jpg 0.411947**

**075_cam1_2.jpg 115_frontal.jpg 0.402047**

**076_cam1_2.jpg 031_frontal.jpg 0.325093**

**077_cam1_2.jpg 115_frontal.jpg 0.396999**

**078_cam1_2.jpg 115_frontal.jpg 0.499158**

**079_cam1_2.jpg 062_frontal.jpg 0.286660**

**080_cam1_2.jpg 115_frontal.jpg 0.352895**

**081_cam1_2.jpg 062_frontal.jpg 0.305786**

**082_cam1_2.jpg 115_frontal.jpg 0.450653**

**083_cam1_2.jpg 083_frontal.jpg 0.499510**

**084_cam1_2.jpg 015_frontal.jpg 0.438260**

**085_cam1_2.jpg 027_frontal.jpg 0.367589**

**086_cam1_2.jpg 030_frontal.jpg 0.313899**

**087_cam1_2.jpg 115_frontal.jpg 0.342353**

**088_cam1_2.jpg 031_frontal.jpg 0.545430**

**089_cam1_2.jpg 115_frontal.jpg 0.347015**

**090_cam1_2.jpg 042_frontal.jpg 0.239438**

**091_cam1_2.jpg 032_frontal.jpg 0.248655**

**092_cam1_2.jpg 049_frontal.jpg 0.431543**

**093_cam1_2.jpg 031_frontal.jpg 0.402556**

**094_cam1_2.jpg 062_frontal.jpg 0.432447**

**095_cam1_2.jpg 115_frontal.jpg 0.372606**

**096_cam1_2.jpg 071_frontal.jpg 0.355791**

**097_cam1_2.jpg 049_frontal.jpg 0.335937**

**098_cam1_2.jpg 058_frontal.jpg 0.262620**

**099_cam1_2.jpg 099_frontal.jpg 0.302782**

**100_cam1_2.jpg 015_frontal.jpg 0.350613**

**101_cam1_2.jpg 016_frontal.jpg 0.392399**

**102_cam1_2.jpg 115_frontal.jpg 0.402901**

**103_cam1_2.jpg 074_frontal.jpg 0.341692**

**104_cam1_2.jpg 042_frontal.jpg 0.371024**

**105_cam1_2.jpg 096_frontal.jpg 0.383568**

**106_cam1_2.jpg 016_frontal.jpg 0.297142**

**107_cam1_2.jpg 107_frontal.jpg 0.388538**

**108_cam1_2.jpg 049_frontal.jpg 0.323331**

**109_cam1_2.jpg 067_frontal.jpg 0.292149**

**110_cam1_2.jpg 115_frontal.jpg 0.429143**

**111_cam1_2.jpg 027_frontal.jpg 0.279580**

**112_cam1_2.jpg 112_frontal.jpg 0.388573**

**113_cam1_2.jpg 071_frontal.jpg 0.355809**

**114_cam1_2.jpg 048_frontal.jpg 0.275012**

**115_cam1_2.jpg 115_frontal.jpg 0.641965**

**116_cam1_2.jpg 112_frontal.jpg 0.375438**

**117_cam1_2.jpg 044_frontal.jpg 0.437493**

**118_cam1_2.jpg 057_frontal.jpg 0.369421**

**119_cam1_2.jpg 115_frontal.jpg 0.399845**

**120_cam1_2.jpg 074_frontal.jpg 0.416889**

**121_cam1_2.jpg 089_frontal.jpg 0.318179**

**122_cam1_2.jpg 084_frontal.jpg 0.302019**

**123_cam1_2.jpg 096_frontal.jpg 0.383084**

**124_cam1_2.jpg 019_frontal.jpg 0.318775**

**125_cam1_2.jpg 079_frontal.jpg 0.339250**

**126_cam1_2.jpg 126_frontal.jpg 0.542157**

**127_cam1_2.jpg 062_frontal.jpg 0.303868**

**128_cam1_2.jpg 015_frontal.jpg 0.372316**

**129_cam1_2.jpg 049_frontal.jpg 0.347040**

**130_cam1_2.jpg 130_frontal.jpg 0.321159**

**Final Score Scface SSR (Vis) cam1-d3**

**Actual Image Recognized Image Score**

**001_cam1_3.jpg 079_frontal.jpg 0.423081**

**002_cam1_3.jpg 011_frontal.jpg 0.366037**

**003_cam1_3.jpg 083_frontal.jpg 0.379174**

**004_cam1_3.jpg 033_frontal.jpg 0.356116**

**005_cam1_3.jpg 117_frontal.jpg 0.418080**

**006_cam1_3.jpg 096_frontal.jpg 0.456074**

**007_cam1_3.jpg 027_frontal.jpg 0.547559**

**008_cam1_3.jpg 008_frontal.jpg 0.817890**

**009_cam1_3.jpg 096_frontal.jpg 0.325448**

**010_cam1_3.jpg 039_frontal.jpg 0.435633**

**011_cam1_3.jpg 096_frontal.jpg 0.469162**

**012_cam1_3.jpg 011_frontal.jpg 0.356547**

**013_cam1_3.jpg 039_frontal.jpg 0.273846**

**014_cam1_3.jpg 014_frontal.jpg 0.657585**

**015_cam1_3.jpg 045_frontal.jpg 0.541906**

**016_cam1_3.jpg 011_frontal.jpg 0.369967**

**017_cam1_3.jpg 050_frontal.jpg 0.314484**

**018_cam1_3.jpg 053_frontal.jpg 0.323051**

**019_cam1_3.jpg 129_frontal.jpg 0.512341**

**020_cam1_3.jpg 096_frontal.jpg 0.496437**

**021_cam1_3.jpg 053_frontal.jpg 0.285711**

**022_cam1_3.jpg 047_frontal.jpg 0.388132**

**023_cam1_3.jpg 003_frontal.jpg 0.310518**

**024_cam1_3.jpg 049_frontal.jpg 0.334685**

**025_cam1_3.jpg 025_frontal.jpg 0.449732**

**026_cam1_3.jpg 042_frontal.jpg 0.392427**

**027_cam1_3.jpg 027_frontal.jpg 0.428785**

**028_cam1_3.jpg 053_frontal.jpg 0.346443**

**029_cam1_3.jpg 022_frontal.jpg 0.433066**

**030_cam1_3.jpg 108_frontal.jpg 0.544415**

**031_cam1_3.jpg 011_frontal.jpg 0.370560**

**032_cam1_3.jpg 096_frontal.jpg 0.424711**

**033_cam1_3.jpg 050_frontal.jpg 0.342473**

**034_cam1_3.jpg 096_frontal.jpg 0.322245**

**035_cam1_3.jpg 053_frontal.jpg 0.352828**

**036_cam1_3.jpg 097_frontal.jpg 0.360264**

**037_cam1_3.jpg 016_frontal.jpg 0.382853**

**038_cam1_3.jpg 109_frontal.jpg 0.686801**

**039_cam1_3.jpg 057_frontal.jpg 0.284734**

**040_cam1_3.jpg 074_frontal.jpg 0.306317**

**041_cam1_3.jpg 096_frontal.jpg 0.476341**

**042_cam1_3.jpg 096_frontal.jpg 0.431173**

**043_cam1_3.jpg 108_frontal.jpg 0.378975**

**044_cam1_3.jpg 097_frontal.jpg 0.282239**

**045_cam1_3.jpg 120_frontal.jpg 0.346299**

**046_cam1_3.jpg 047_frontal.jpg 0.433407**

**047_cam1_3.jpg 047_frontal.jpg 0.483961**

**048_cam1_3.jpg 099_frontal.jpg 0.533172**

**049_cam1_3.jpg 005_frontal.jpg 0.418975**

**050_cam1_3.jpg 096_frontal.jpg 0.370587**

**051_cam1_3.jpg 059_frontal.jpg 0.462746**

**052_cam1_3.jpg 115_frontal.jpg 0.451941**

**053_cam1_3.jpg 047_frontal.jpg 0.460680**

**054_cam1_3.jpg 115_frontal.jpg 0.438743**

**055_cam1_3.jpg 115_frontal.jpg 0.363034**

**056_cam1_3.jpg 042_frontal.jpg 0.494157**

**057_cam1_3.jpg 057_frontal.jpg 0.301252**

**058_cam1_3.jpg 096_frontal.jpg 0.410438**

**059_cam1_3.jpg 101_frontal.jpg 0.398772**

**060_cam1_3.jpg 049_frontal.jpg 0.455805**

**061_cam1_3.jpg 082_frontal.jpg 0.279266**

**062_cam1_3.jpg 069_frontal.jpg 0.383456**

**063_cam1_3.jpg 063_frontal.jpg 0.469501**

**064_cam1_3.jpg 064_frontal.jpg 0.522085**

**065_cam1_3.jpg 012_frontal.jpg 0.292106**

**066_cam1_3.jpg 115_frontal.jpg 0.487660**

**067_cam1_3.jpg 015_frontal.jpg 0.307338**

**068_cam1_3.jpg 033_frontal.jpg 0.503568**

**069_cam1_3.jpg 057_frontal.jpg 0.443723**

**070_cam1_3.jpg 056_frontal.jpg 0.312791**

**071_cam1_3.jpg 074_frontal.jpg 0.252010**

**072_cam1_3.jpg 044_frontal.jpg 0.275599**

**073_cam1_3.jpg 056_frontal.jpg 0.352500**

**074_cam1_3.jpg 049_frontal.jpg 0.438005**

**075_cam1_3.jpg 117_frontal.jpg 0.350800**

**076_cam1_3.jpg 049_frontal.jpg 0.364922**

**077_cam1_3.jpg 047_frontal.jpg 0.497765**

**078_cam1_3.jpg 078_frontal.jpg 0.367874**

**079_cam1_3.jpg 079_frontal.jpg 0.347038**

**080_cam1_3.jpg 053_frontal.jpg 0.370900**

**081_cam1_3.jpg 031_frontal.jpg 0.307052**

**082_cam1_3.jpg 112_frontal.jpg 0.441700**

**083_cam1_3.jpg 083_frontal.jpg 0.438909**

**084_cam1_3.jpg 005_frontal.jpg 0.495188**

**085_cam1_3.jpg 059_frontal.jpg 0.453728**

**086_cam1_3.jpg 005_frontal.jpg 0.505095**

**087_cam1_3.jpg 120_frontal.jpg 0.415223**

**088_cam1_3.jpg 059_frontal.jpg 0.396190**

**089_cam1_3.jpg 096_frontal.jpg 0.325268**

**090_cam1_3.jpg 047_frontal.jpg 0.437608**

**091_cam1_3.jpg 097_frontal.jpg 0.353360**

**092_cam1_3.jpg 040_frontal.jpg 0.313732**

**093_cam1_3.jpg 074_frontal.jpg 0.351210**

**094_cam1_3.jpg 047_frontal.jpg 0.362988**

**095_cam1_3.jpg 109_frontal.jpg 0.511690**

**096_cam1_3.jpg 096_frontal.jpg 0.310629**

**097_cam1_3.jpg 096_frontal.jpg 0.419036**

**098_cam1_3.jpg 058_frontal.jpg 0.421953**

**099_cam1_3.jpg 050_frontal.jpg 0.365863**

**100_cam1_3.jpg 100_frontal.jpg 0.538666**

**101_cam1_3.jpg 120_frontal.jpg 0.377836**

**102_cam1_3.jpg 115_frontal.jpg 0.356963**

**103_cam1_3.jpg 027_frontal.jpg 0.700188**

**104_cam1_3.jpg 005_frontal.jpg 0.368524**

**105_cam1_3.jpg 012_frontal.jpg 0.387471**

**106_cam1_3.jpg 059_frontal.jpg 0.337718**

**107_cam1_3.jpg 107_frontal.jpg 0.450179**

**108_cam1_3.jpg 044_frontal.jpg 0.405175**

**109_cam1_3.jpg 109_frontal.jpg 0.557992**

**110_cam1_3.jpg 096_frontal.jpg 0.424216**

**111_cam1_3.jpg 011_frontal.jpg 0.340519**

**112_cam1_3.jpg 112_frontal.jpg 0.413075**

**113_cam1_3.jpg 112_frontal.jpg 0.453527**

**114_cam1_3.jpg 074_frontal.jpg 0.389455**

**115_cam1_3.jpg 062_frontal.jpg 0.447339**

**116_cam1_3.jpg 112_frontal.jpg 0.657662**

**117_cam1_3.jpg 035_frontal.jpg 0.411115**

**118_cam1_3.jpg 015_frontal.jpg 0.360339**

**119_cam1_3.jpg 115_frontal.jpg 0.457191**

**120_cam1_3.jpg 120_frontal.jpg 0.578574**

**121_cam1_3.jpg 033_frontal.jpg 0.379269**

**122_cam1_3.jpg 044_frontal.jpg 0.397056**

**123_cam1_3.jpg 096_frontal.jpg 0.292130**

**124_cam1_3.jpg 005_frontal.jpg 0.465865**

**125_cam1_3.jpg 005_frontal.jpg 0.488250**

**126_cam1_3.jpg 126_frontal.jpg 0.367335**

**127_cam1_3.jpg 058_frontal.jpg 0.318090**

**128_cam1_3.jpg 083_frontal.jpg 0.254425**

**129_cam1_3.jpg 119_frontal.jpg 0.378319**

**130_cam1_3.jpg 096_frontal.jpg 0.559560**

**Final Score Scface SSR (Vis) cam2-d1**

**Actual Image Recognized Image Score**

**001_cam2_1.jpg 089_frontal.jpg 0.333434**

**002_cam2_1.jpg 096_frontal.jpg 0.373200**

**003_cam2_1.jpg 049_frontal.jpg 0.647169**

**004_cam2_1.jpg 074_frontal.jpg 0.343527**

**005_cam2_1.jpg 096_frontal.jpg 0.511715**

**006_cam2_1.jpg 049_frontal.jpg 0.495645**

**007_cam2_1.jpg 035_frontal.jpg 0.461401**

**008_cam2_1.jpg 044_frontal.jpg 0.505230**

**009_cam2_1.jpg 096_frontal.jpg 0.336634**

**010_cam2_1.jpg 076_frontal.jpg 0.366616**

**011_cam2_1.jpg 059_frontal.jpg 0.445710**

**012_cam2_1.jpg 022_frontal.jpg 0.387447**

**013_cam2_1.jpg 027_frontal.jpg 0.505970**

**014_cam2_1.jpg 126_frontal.jpg 0.596992**

**015_cam2_1.jpg 049_frontal.jpg 0.588494**

**016_cam2_1.jpg 096_frontal.jpg 0.758283**

**017_cam2_1.jpg 005_frontal.jpg 0.279731**

**018_cam2_1.jpg 096_frontal.jpg 0.366957**

**019_cam2_1.jpg 096_frontal.jpg 0.665972**

**020_cam2_1.jpg 096_frontal.jpg 0.426123**

**021_cam2_1.jpg 005_frontal.jpg 0.324474**

**022_cam2_1.jpg 096_frontal.jpg 0.394166**

**023_cam2_1.jpg 096_frontal.jpg 0.496492**

**024_cam2_1.jpg 096_frontal.jpg 0.671872**

**025_cam2_1.jpg 078_frontal.jpg 0.521665**

**026_cam2_1.jpg 096_frontal.jpg 0.358148**

**027_cam2_1.jpg 027_frontal.jpg 0.427632**

**028_cam2_1.jpg 028_frontal.jpg 0.455633**

**029_cam2_1.jpg 053_frontal.jpg 0.463495**

**030_cam2_1.jpg 012_frontal.jpg 0.375304**

**031_cam2_1.jpg 089_frontal.jpg 0.571513**

**032_cam2_1.jpg 096_frontal.jpg 0.737733**

**033_cam2_1.jpg 005_frontal.jpg 0.334791**

**034_cam2_1.jpg 089_frontal.jpg 0.349196**

**035_cam2_1.jpg 096_frontal.jpg 0.629857**

**036_cam2_1.jpg 042_frontal.jpg 0.360163**

**037_cam2_1.jpg 012_frontal.jpg 0.446764**

**038_cam2_1.jpg 096_frontal.jpg 0.639638**

**039_cam2_1.jpg 022_frontal.jpg 0.260433**

**040_cam2_1.jpg 096_frontal.jpg 0.496325**

**041_cam2_1.jpg 059_frontal.jpg 0.334979**

**042_cam2_1.jpg 096_frontal.jpg 0.494011**

**043_cam2_1.jpg 096_frontal.jpg 0.844600**

**044_cam2_1.jpg 059_frontal.jpg 0.584836**

**045_cam2_1.jpg 096_frontal.jpg 0.392932**

**046_cam2_1.jpg 049_frontal.jpg 0.364971**

**047_cam2_1.jpg 027_frontal.jpg 0.618111**

**048_cam2_1.jpg 096_frontal.jpg 0.664720**

**049_cam2_1.jpg 096_frontal.jpg 0.440456**

**050_cam2_1.jpg 078_frontal.jpg 0.419029**

**051_cam2_1.jpg 096_frontal.jpg 0.688657**

**052_cam2_1.jpg 115_frontal.jpg 0.399662**

**053_cam2_1.jpg 096_frontal.jpg 0.518136**

**054_cam2_1.jpg 096_frontal.jpg 0.662306**

**055_cam2_1.jpg 042_frontal.jpg 0.403309**

**056_cam2_1.jpg 089_frontal.jpg 0.423344**

**057_cam2_1.jpg 126_frontal.jpg 0.438639**

**058_cam2_1.jpg 096_frontal.jpg 0.462529**

**059_cam2_1.jpg 096_frontal.jpg 0.652271**

**060_cam2_1.jpg 096_frontal.jpg 0.369917**

**061_cam2_1.jpg 096_frontal.jpg 0.364139**

**062_cam2_1.jpg 062_frontal.jpg 0.553098**

**063_cam2_1.jpg 063_frontal.jpg 0.693629**

**064_cam2_1.jpg 012_frontal.jpg 0.544443**

**065_cam2_1.jpg 076_frontal.jpg 0.470814**

**066_cam2_1.jpg 074_frontal.jpg 0.547634**

**067_cam2_1.jpg 049_frontal.jpg 0.341976**

**068_cam2_1.jpg 068_frontal.jpg 0.711359**

**069_cam2_1.jpg 078_frontal.jpg 0.442612**

**070_cam2_1.jpg 022_frontal.jpg 0.393528**

**071_cam2_1.jpg 096_frontal.jpg 0.741357**

**072_cam2_1.jpg 096_frontal.jpg 0.692011**

**073_cam2_1.jpg 027_frontal.jpg 0.371445**

**074_cam2_1.jpg 015_frontal.jpg 0.400202**

**075_cam2_1.jpg 015_frontal.jpg 0.576953**

**076_cam2_1.jpg 076_frontal.jpg 0.401824**

**077_cam2_1.jpg 016_frontal.jpg 0.470832**

**078_cam2_1.jpg 096_frontal.jpg 0.648173**

**079_cam2_1.jpg 096_frontal.jpg 0.386276**

**080_cam2_1.jpg 016_frontal.jpg 0.316659**

**081_cam2_1.jpg 096_frontal.jpg 0.701677**

**082_cam2_1.jpg 100_frontal.jpg 0.359172**

**083_cam2_1.jpg 096_frontal.jpg 0.491134**

**084_cam2_1.jpg 096_frontal.jpg 0.470036**

**085_cam2_1.jpg 096_frontal.jpg 0.487754**

**086_cam2_1.jpg 005_frontal.jpg 0.650368**

**087_cam2_1.jpg 074_frontal.jpg 0.303826**

**088_cam2_1.jpg 074_frontal.jpg 0.484749**

**089_cam2_1.jpg 096_frontal.jpg 0.509876**

**090_cam2_1.jpg 059_frontal.jpg 0.414111**

**091_cam2_1.jpg 077_frontal.jpg 0.371294**

**092_cam2_1.jpg 025_frontal.jpg 0.456720**

**093_cam2_1.jpg 120_frontal.jpg 0.465612**

**094_cam2_1.jpg 049_frontal.jpg 0.325111**

**095_cam2_1.jpg 096_frontal.jpg 0.768266**

**096_cam2_1.jpg 096_frontal.jpg 0.739074**

**097_cam2_1.jpg 126_frontal.jpg 0.616417**

**098_cam2_1.jpg 096_frontal.jpg 0.531074**

**099_cam2_1.jpg 096_frontal.jpg 0.314299**

**100_cam2_1.jpg 048_frontal.jpg 0.379426**

**101_cam2_1.jpg 096_frontal.jpg 0.440093**

**102_cam2_1.jpg 115_frontal.jpg 0.472167**

**103_cam2_1.jpg 007_frontal.jpg 0.612343**

**104_cam2_1.jpg 096_frontal.jpg 0.891817**

**105_cam2_1.jpg 096_frontal.jpg 0.349949**

**106_cam2_1.jpg 049_frontal.jpg 0.464768**

**107_cam2_1.jpg 044_frontal.jpg 0.343865**

**108_cam2_1.jpg 005_frontal.jpg 0.533816**

**109_cam2_1.jpg 109_frontal.jpg 0.388020**

**110_cam2_1.jpg 027_frontal.jpg 0.420996**

**111_cam2_1.jpg 115_frontal.jpg 0.402263**

**112_cam2_1.jpg 049_frontal.jpg 0.325021**

**113_cam2_1.jpg 074_frontal.jpg 0.557130**

**114_cam2_1.jpg 014_frontal.jpg 0.440412**

**115_cam2_1.jpg 012_frontal.jpg 0.483355**

**116_cam2_1.jpg 042_frontal.jpg 0.338000**

**117_cam2_1.jpg 027_frontal.jpg 0.540349**

**118_cam2_1.jpg 096_frontal.jpg 0.748156**

**119_cam2_1.jpg 096_frontal.jpg 0.520314**

**120_cam2_1.jpg 065_frontal.jpg 0.412993**

**121_cam2_1.jpg 096_frontal.jpg 0.700859**

**122_cam2_1.jpg 096_frontal.jpg 0.404123**

**123_cam2_1.jpg 012_frontal.jpg 0.635700**

**124_cam2_1.jpg 101_frontal.jpg 0.374619**

**125_cam2_1.jpg 096_frontal.jpg 0.534132**

**126_cam2_1.jpg 096_frontal.jpg 0.720969**

**127_cam2_1.jpg 074_frontal.jpg 0.362064**

**128_cam2_1.jpg 005_frontal.jpg 0.416481**

**129_cam2_1.jpg 059_frontal.jpg 0.555870**

**130_cam2_1.jpg 027_frontal.jpg 0.575433**

**Final Score Scface SSR (Vis) cam2-d2**

**Actual Image Recognized Image Score**

**001_cam2_1.jpg 089_frontal.jpg 0.333434**

**002_cam2_1.jpg 096_frontal.jpg 0.373200**

**003_cam2_1.jpg 049_frontal.jpg 0.647169**

**004_cam2_1.jpg 074_frontal.jpg 0.343527**

**005_cam2_1.jpg 096_frontal.jpg 0.511715**

**006_cam2_1.jpg 049_frontal.jpg 0.495645**

**007_cam2_1.jpg 035_frontal.jpg 0.461401**

**008_cam2_1.jpg 044_frontal.jpg 0.505230**

**009_cam2_1.jpg 096_frontal.jpg 0.336634**

**010_cam2_1.jpg 076_frontal.jpg 0.366616**

**011_cam2_1.jpg 059_frontal.jpg 0.445710**

**012_cam2_1.jpg 022_frontal.jpg 0.387447**

**013_cam2_1.jpg 027_frontal.jpg 0.505970**

**014_cam2_1.jpg 126_frontal.jpg 0.596992**

**015_cam2_1.jpg 049_frontal.jpg 0.588494**

**016_cam2_1.jpg 096_frontal.jpg 0.758283**

**017_cam2_1.jpg 005_frontal.jpg 0.279731**

**018_cam2_1.jpg 096_frontal.jpg 0.366957**

**019_cam2_1.jpg 096_frontal.jpg 0.665972**

**020_cam2_1.jpg 096_frontal.jpg 0.426123**

**021_cam2_1.jpg 005_frontal.jpg 0.324474**

**022_cam2_1.jpg 096_frontal.jpg 0.394166**

**023_cam2_1.jpg 096_frontal.jpg 0.496492**

**024_cam2_1.jpg 096_frontal.jpg 0.671872**

**025_cam2_1.jpg 078_frontal.jpg 0.521665**

**026_cam2_1.jpg 096_frontal.jpg 0.358148**

**027_cam2_1.jpg 027_frontal.jpg 0.427632**

**028_cam2_1.jpg 028_frontal.jpg 0.455633**

**029_cam2_1.jpg 053_frontal.jpg 0.463495**

**030_cam2_1.jpg 012_frontal.jpg 0.375304**

**031_cam2_1.jpg 089_frontal.jpg 0.571513**

**032_cam2_1.jpg 096_frontal.jpg 0.737733**

**033_cam2_1.jpg 005_frontal.jpg 0.334791**

**034_cam2_1.jpg 089_frontal.jpg 0.349196**

**035_cam2_1.jpg 096_frontal.jpg 0.629857**

**036_cam2_1.jpg 042_frontal.jpg 0.360163**

**037_cam2_1.jpg 012_frontal.jpg 0.446764**

**038_cam2_1.jpg 096_frontal.jpg 0.639638**

**039_cam2_1.jpg 022_frontal.jpg 0.260433**

**040_cam2_1.jpg 096_frontal.jpg 0.496325**

**041_cam2_1.jpg 059_frontal.jpg 0.334979**

**042_cam2_1.jpg 096_frontal.jpg 0.494011**

**043_cam2_1.jpg 096_frontal.jpg 0.844600**

**044_cam2_1.jpg 059_frontal.jpg 0.584836**

**045_cam2_1.jpg 096_frontal.jpg 0.392932**

**046_cam2_1.jpg 049_frontal.jpg 0.364971**

**047_cam2_1.jpg 027_frontal.jpg 0.618111**

**048_cam2_1.jpg 096_frontal.jpg 0.664720**

**049_cam2_1.jpg 096_frontal.jpg 0.440456**

**050_cam2_1.jpg 078_frontal.jpg 0.419029**

**051_cam2_1.jpg 096_frontal.jpg 0.688657**

**052_cam2_1.jpg 115_frontal.jpg 0.399662**

**053_cam2_1.jpg 096_frontal.jpg 0.518136**

**054_cam2_1.jpg 096_frontal.jpg 0.662306**

**055_cam2_1.jpg 042_frontal.jpg 0.403309**

**056_cam2_1.jpg 089_frontal.jpg 0.423344**

**057_cam2_1.jpg 126_frontal.jpg 0.438639**

**058_cam2_1.jpg 096_frontal.jpg 0.462529**

**059_cam2_1.jpg 096_frontal.jpg 0.652271**

**060_cam2_1.jpg 096_frontal.jpg 0.369917**

**061_cam2_1.jpg 096_frontal.jpg 0.364139**

**062_cam2_1.jpg 062_frontal.jpg 0.553098**

**063_cam2_1.jpg 063_frontal.jpg 0.693629**

**064_cam2_1.jpg 012_frontal.jpg 0.544443**

**065_cam2_1.jpg 076_frontal.jpg 0.470814**

**066_cam2_1.jpg 074_frontal.jpg 0.547634**

**067_cam2_1.jpg 049_frontal.jpg 0.341976**

**068_cam2_1.jpg 068_frontal.jpg 0.711359**

**069_cam2_1.jpg 078_frontal.jpg 0.442612**

**070_cam2_1.jpg 022_frontal.jpg 0.393528**

**071_cam2_1.jpg 096_frontal.jpg 0.741357**

**072_cam2_1.jpg 096_frontal.jpg 0.692011**

**073_cam2_1.jpg 027_frontal.jpg 0.371445**

**074_cam2_1.jpg 015_frontal.jpg 0.400202**

**075_cam2_1.jpg 015_frontal.jpg 0.576953**

**076_cam2_1.jpg 076_frontal.jpg 0.401824**

**077_cam2_1.jpg 016_frontal.jpg 0.470832**

**078_cam2_1.jpg 096_frontal.jpg 0.648173**

**079_cam2_1.jpg 096_frontal.jpg 0.386276**

**080_cam2_1.jpg 016_frontal.jpg 0.316659**

**081_cam2_1.jpg 096_frontal.jpg 0.701677**

**082_cam2_1.jpg 100_frontal.jpg 0.359172**

**083_cam2_1.jpg 096_frontal.jpg 0.491134**

**084_cam2_1.jpg 096_frontal.jpg 0.470036**

**085_cam2_1.jpg 096_frontal.jpg 0.487754**

**086_cam2_1.jpg 005_frontal.jpg 0.650368**

**087_cam2_1.jpg 074_frontal.jpg 0.303826**

**088_cam2_1.jpg 074_frontal.jpg 0.484749**

**089_cam2_1.jpg 096_frontal.jpg 0.509876**

**090_cam2_1.jpg 059_frontal.jpg 0.414111**

**091_cam2_1.jpg 077_frontal.jpg 0.371294**

**092_cam2_1.jpg 025_frontal.jpg 0.456720**

**093_cam2_1.jpg 120_frontal.jpg 0.465612**

**094_cam2_1.jpg 049_frontal.jpg 0.325111**

**095_cam2_1.jpg 096_frontal.jpg 0.768266**

**096_cam2_1.jpg 096_frontal.jpg 0.739074**

**097_cam2_1.jpg 126_frontal.jpg 0.616417**

**098_cam2_1.jpg 096_frontal.jpg 0.531074**

**099_cam2_1.jpg 096_frontal.jpg 0.314299**

**100_cam2_1.jpg 048_frontal.jpg 0.379426**

**101_cam2_1.jpg 096_frontal.jpg 0.440093**

**102_cam2_1.jpg 115_frontal.jpg 0.472167**

**103_cam2_1.jpg 007_frontal.jpg 0.612343**

**104_cam2_1.jpg 096_frontal.jpg 0.891817**

**105_cam2_1.jpg 096_frontal.jpg 0.349949**

**106_cam2_1.jpg 049_frontal.jpg 0.464768**

**107_cam2_1.jpg 044_frontal.jpg 0.343865**

**108_cam2_1.jpg 005_frontal.jpg 0.533816**

**109_cam2_1.jpg 109_frontal.jpg 0.388020**

**110_cam2_1.jpg 027_frontal.jpg 0.420996**

**111_cam2_1.jpg 115_frontal.jpg 0.402263**

**112_cam2_1.jpg 049_frontal.jpg 0.325021**

**113_cam2_1.jpg 074_frontal.jpg 0.557130**

**114_cam2_1.jpg 014_frontal.jpg 0.440412**

**115_cam2_1.jpg 012_frontal.jpg 0.483355**

**116_cam2_1.jpg 042_frontal.jpg 0.338000**

**117_cam2_1.jpg 027_frontal.jpg 0.540349**

**118_cam2_1.jpg 096_frontal.jpg 0.748156**

**119_cam2_1.jpg 096_frontal.jpg 0.520314**

**120_cam2_1.jpg 065_frontal.jpg 0.412993**

**121_cam2_1.jpg 096_frontal.jpg 0.700859**

**122_cam2_1.jpg 096_frontal.jpg 0.404123**

**123_cam2_1.jpg 012_frontal.jpg 0.635700**

**124_cam2_1.jpg 101_frontal.jpg 0.374619**

**125_cam2_1.jpg 096_frontal.jpg 0.534132**

**126_cam2_1.jpg 096_frontal.jpg 0.720969**

**127_cam2_1.jpg 074_frontal.jpg 0.362064**

**128_cam2_1.jpg 005_frontal.jpg 0.416481**

**129_cam2_1.jpg 059_frontal.jpg 0.555870**

**130_cam2_1.jpg 027_frontal.jpg 0.575433**

**Final Score Scface SSR (Vis) cam2-d3**

**Actual Image Recognized Image Score**

**001_cam2_3.jpg 001_frontal.jpg 0.231103**

**002_cam2_3.jpg 096_frontal.jpg 0.272750**

**003_cam2_3.jpg 022_frontal.jpg 0.322629**

**004_cam2_3.jpg 096_frontal.jpg 0.319920**

**005_cam2_3.jpg 096_frontal.jpg 0.311561**

**006_cam2_3.jpg 001_frontal.jpg 0.372533**

**007_cam2_3.jpg 074_frontal.jpg 0.457437**

**008_cam2_3.jpg 008_frontal.jpg 0.683455**

**009_cam2_3.jpg 125_frontal.jpg 0.312055**

**010_cam2_3.jpg 096_frontal.jpg 0.402142**

**011_cam2_3.jpg 096_frontal.jpg 0.471791**

**012_cam2_3.jpg 049_frontal.jpg 0.299349**

**013_cam2_3.jpg 096_frontal.jpg 0.307919**

**014_cam2_3.jpg 014_frontal.jpg 0.617144**

**015_cam2_3.jpg 057_frontal.jpg 0.562162**

**016_cam2_3.jpg 096_frontal.jpg 0.423348**

**017_cam2_3.jpg 017_frontal.jpg 0.385089**

**018_cam2_3.jpg 050_frontal.jpg 0.232372**

**019_cam2_3.jpg 083_frontal.jpg 0.340769**

**020_cam2_3.jpg 096_frontal.jpg 0.250888**

**021_cam2_3.jpg 115_frontal.jpg 0.200614**

**022_cam2_3.jpg 011_frontal.jpg 0.333572**

**023_cam2_3.jpg 038_frontal.jpg 0.218026**

**024_cam2_3.jpg 016_frontal.jpg 0.338204**

**025_cam2_3.jpg 025_frontal.jpg 0.361726**

**026_cam2_3.jpg 115_frontal.jpg 0.238624**

**027_cam2_3.jpg 117_frontal.jpg 0.352366**

**028_cam2_3.jpg 016_frontal.jpg 0.322326**

**029_cam2_3.jpg 022_frontal.jpg 0.360942**

**030_cam2_3.jpg 078_frontal.jpg 0.270683**

**031_cam2_3.jpg 016_frontal.jpg 0.289579**

**032_cam2_3.jpg 096_frontal.jpg 0.373137**

**033_cam2_3.jpg 005_frontal.jpg 0.306461**

**034_cam2_3.jpg 050_frontal.jpg 0.238429**

**035_cam2_3.jpg 053_frontal.jpg 0.241018**

**036_cam2_3.jpg 096_frontal.jpg 0.315087**

**037_cam2_3.jpg 016_frontal.jpg 0.699949**

**038_cam2_3.jpg 096_frontal.jpg 0.659367**

**039_cam2_3.jpg 049_frontal.jpg 0.275653**

**040_cam2_3.jpg 021_frontal.jpg 0.207413**

**041_cam2_3.jpg 019_frontal.jpg 0.309975**

**042_cam2_3.jpg 096_frontal.jpg 0.375086**

**043_cam2_3.jpg 074_frontal.jpg 0.353058**

**044_cam2_3.jpg 059_frontal.jpg 0.316647**

**045_cam2_3.jpg 037_frontal.jpg 0.359055**

**046_cam2_3.jpg 096_frontal.jpg 0.396334**

**047_cam2_3.jpg 019_frontal.jpg 0.274962**

**048_cam2_3.jpg 101_frontal.jpg 0.346537**

**049_cam2_3.jpg 126_frontal.jpg 0.274256**

**050_cam2_3.jpg 016_frontal.jpg 0.252383**

**051_cam2_3.jpg 049_frontal.jpg 0.331158**

**052_cam2_3.jpg 115_frontal.jpg 0.327590**

**053_cam2_3.jpg 096_frontal.jpg 0.472792**

**054_cam2_3.jpg 115_frontal.jpg 0.428863**

**055_cam2_3.jpg 062_frontal.jpg 0.354492**

**056_cam2_3.jpg 096_frontal.jpg 0.188119**

**057_cam2_3.jpg 005_frontal.jpg 0.303965**

**058_cam2_3.jpg 059_frontal.jpg 0.322561**

**059_cam2_3.jpg 005_frontal.jpg 0.426070**

**060_cam2_3.jpg 126_frontal.jpg 0.378111**

**061_cam2_3.jpg 049_frontal.jpg 0.268791**

**062_cam2_3.jpg 062_frontal.jpg 0.620975**

**063_cam2_3.jpg 016_frontal.jpg 0.340636**

**064_cam2_3.jpg 059_frontal.jpg 0.375743**

**065_cam2_3.jpg 042_frontal.jpg 0.426259**

**066_cam2_3.jpg 074_frontal.jpg 0.432405**

**067_cam2_3.jpg 015_frontal.jpg 0.288020**

**068_cam2_3.jpg 115_frontal.jpg 0.395881**

**069_cam2_3.jpg 057_frontal.jpg 0.372264**

**070_cam2_3.jpg 022_frontal.jpg 0.235846**

**071_cam2_3.jpg 074_frontal.jpg 0.415147**

**072_cam2_3.jpg 059_frontal.jpg 0.270538**

**073_cam2_3.jpg 096_frontal.jpg 0.473021**

**074_cam2_3.jpg 096_frontal.jpg 0.412815**

**075_cam2_3.jpg 066_frontal.jpg 0.318394**

**076_cam2_3.jpg 015_frontal.jpg 0.185696**

**077_cam2_3.jpg 016_frontal.jpg 0.356391**

**078_cam2_3.jpg 078_frontal.jpg 0.252208**

**079_cam2_3.jpg 062_frontal.jpg 0.275025**

**080_cam2_3.jpg 115_frontal.jpg 0.202886**

**081_cam2_3.jpg 016_frontal.jpg 0.298664**

**082_cam2_3.jpg 059_frontal.jpg 0.363682**

**083_cam2_3.jpg 083_frontal.jpg 0.501426**

**084_cam2_3.jpg 096_frontal.jpg 0.325753**

**085_cam2_3.jpg 062_frontal.jpg 0.392120**

**086_cam2_3.jpg 115_frontal.jpg 0.341810**

**087_cam2_3.jpg 096_frontal.jpg 0.262232**

**088_cam2_3.jpg 057_frontal.jpg 0.343887**

**089_cam2_3.jpg 089_frontal.jpg 0.247734**

**090_cam2_3.jpg 016_frontal.jpg 0.426355**

**091_cam2_3.jpg 016_frontal.jpg 0.256348**

**092_cam2_3.jpg 016_frontal.jpg 0.367379**

**093_cam2_3.jpg 093_frontal.jpg 0.264393**

**094_cam2_3.jpg 117_frontal.jpg 0.410265**

**095_cam2_3.jpg 049_frontal.jpg 0.417646**

**096_cam2_3.jpg 096_frontal.jpg 0.258261**

**097_cam2_3.jpg 035_frontal.jpg 0.315621**

**098_cam2_3.jpg 117_frontal.jpg 0.295531**

**099_cam2_3.jpg 019_frontal.jpg 0.268824**

**100_cam2_3.jpg 100_frontal.jpg 0.350773**

**101_cam2_3.jpg 101_frontal.jpg 0.426956**

**102_cam2_3.jpg 115_frontal.jpg 0.454849**

**103_cam2_3.jpg 115_frontal.jpg 0.287104**

**104_cam2_3.jpg 049_frontal.jpg 0.390314**

**105_cam2_3.jpg 096_frontal.jpg 0.302825**

**106_cam2_3.jpg 023_frontal.jpg 0.242938**

**107_cam2_3.jpg 062_frontal.jpg 0.461441**

**108_cam2_3.jpg 032_frontal.jpg 0.383869**

**109_cam2_3.jpg 042_frontal.jpg 0.365518**

**110_cam2_3.jpg 096_frontal.jpg 0.334930**

**111_cam2_3.jpg 117_frontal.jpg 0.285986**

**112_cam2_3.jpg 112_frontal.jpg 0.219823**

**113_cam2_3.jpg 019_frontal.jpg 0.344118**

**114_cam2_3.jpg 096_frontal.jpg 0.334607**

**115_cam2_3.jpg 115_frontal.jpg 0.355197**

**116_cam2_3.jpg 003_frontal.jpg 0.345648**

**117_cam2_3.jpg 074_frontal.jpg 0.231388**

**118_cam2_3.jpg 096_frontal.jpg 0.362234**

**119_cam2_3.jpg 096_frontal.jpg 0.218550**

**120_cam2_3.jpg 096_frontal.jpg 0.339830**

**121_cam2_3.jpg 027_frontal.jpg 0.385074**

**122_cam2_3.jpg 096_frontal.jpg 0.340213**

**123_cam2_3.jpg 064_frontal.jpg 0.278953**

**124_cam2_3.jpg 017_frontal.jpg 0.418494**

**125_cam2_3.jpg 126_frontal.jpg 0.435610**

**126_cam2_3.jpg 096_frontal.jpg 0.481965**

**127_cam2_3.jpg 083_frontal.jpg 0.290745**

**128_cam2_3.jpg 057_frontal.jpg 0.250870**

**129_cam2_3.jpg 043_frontal.jpg 0.321284**

**130_cam2_3.jpg 096_frontal.jpg 0.317602**

**Final Score Scface SSR (Vis) cam3-d1**

**Actual Image Recognized Image Score**

**001_cam3_1.jpg 049_frontal.jpg 0.446346**

**002_cam3_1.jpg 005_frontal.jpg 0.356333**

**003_cam3_1.jpg 096_frontal.jpg 0.711852**

**004_cam3_1.jpg 096_frontal.jpg 0.504708**

**005_cam3_1.jpg 096_frontal.jpg 0.753607**

**006_cam3_1.jpg 049_frontal.jpg 0.454631**

**007_cam3_1.jpg 017_frontal.jpg 0.497840**

**008_cam3_1.jpg 063_frontal.jpg 0.495411**

**009_cam3_1.jpg 096_frontal.jpg 0.808557**

**010_cam3_1.jpg 096_frontal.jpg 0.824244**

**011_cam3_1.jpg 059_frontal.jpg 0.325255**

**012_cam3_1.jpg 089_frontal.jpg 0.443120**

**013_cam3_1.jpg 005_frontal.jpg 0.572895**

**014_cam3_1.jpg 014_frontal.jpg 0.614411**

**015_cam3_1.jpg 096_frontal.jpg 0.560267**

**016_cam3_1.jpg 096_frontal.jpg 0.807711**

**017_cam3_1.jpg 096_frontal.jpg 0.315578**

**018_cam3_1.jpg 096_frontal.jpg 0.679734**

**019_cam3_1.jpg 077_frontal.jpg 0.400444**

**020_cam3_1.jpg 096_frontal.jpg 0.469436**

**021_cam3_1.jpg 033_frontal.jpg 0.327986**

**022_cam3_1.jpg 014_frontal.jpg 0.724080**

**023_cam3_1.jpg 096_frontal.jpg 0.460608**

**024_cam3_1.jpg 096_frontal.jpg 0.703367**

**025_cam3_1.jpg 014_frontal.jpg 0.486110**

**026_cam3_1.jpg 115_frontal.jpg 0.314202**

**027_cam3_1.jpg 027_frontal.jpg 0.546515**

**028_cam3_1.jpg 117_frontal.jpg 0.491254**

**029_cam3_1.jpg 049_frontal.jpg 0.513733**

**030_cam3_1.jpg 039_frontal.jpg 0.394156**

**031_cam3_1.jpg 044_frontal.jpg 0.499946**

**032_cam3_1.jpg 096_frontal.jpg 0.738222**

**033_cam3_1.jpg 049_frontal.jpg 0.325528**

**034_cam3_1.jpg 076_frontal.jpg 0.409687**

**035_cam3_1.jpg 096_frontal.jpg 0.511517**

**036_cam3_1.jpg 016_frontal.jpg 0.334381**

**037_cam3_1.jpg 027_frontal.jpg 0.711616**

**038_cam3_1.jpg 042_frontal.jpg 0.567342**

**039_cam3_1.jpg 005_frontal.jpg 0.404340**

**040_cam3_1.jpg 005_frontal.jpg 0.381843**

**041_cam3_1.jpg 078_frontal.jpg 0.424115**

**042_cam3_1.jpg 096_frontal.jpg 0.386081**

**043_cam3_1.jpg 096_frontal.jpg 0.364192**

**044_cam3_1.jpg 100_frontal.jpg 0.449978**

**045_cam3_1.jpg 124_frontal.jpg 0.257182**

**046_cam3_1.jpg 100_frontal.jpg 0.377211**

**047_cam3_1.jpg 019_frontal.jpg 0.434604**

**048_cam3_1.jpg 126_frontal.jpg 0.840959**

**049_cam3_1.jpg 096_frontal.jpg 0.748784**

**050_cam3_1.jpg 096_frontal.jpg 0.619852**

**051_cam3_1.jpg 022_frontal.jpg 0.542219**

**052_cam3_1.jpg 096_frontal.jpg 0.675881**

**053_cam3_1.jpg 096_frontal.jpg 0.482288**

**054_cam3_1.jpg 126_frontal.jpg 0.620563**

**055_cam3_1.jpg 096_frontal.jpg 0.763165**

**056_cam3_1.jpg 096_frontal.jpg 0.396028**

**057_cam3_1.jpg 126_frontal.jpg 0.380593**

**058_cam3_1.jpg 096_frontal.jpg 0.401561**

**059_cam3_1.jpg 096_frontal.jpg 0.467700**

**060_cam3_1.jpg 042_frontal.jpg 0.513692**

**061_cam3_1.jpg 099_frontal.jpg 0.348957**

**062_cam3_1.jpg 062_frontal.jpg 0.455650**

**063_cam3_1.jpg 096_frontal.jpg 0.370156**

**064_cam3_1.jpg 027_frontal.jpg 0.511185**

**065_cam3_1.jpg 099_frontal.jpg 0.392292**

**066_cam3_1.jpg 109_frontal.jpg 0.500219**

**067_cam3_1.jpg 089_frontal.jpg 0.365108**

**068_cam3_1.jpg 033_frontal.jpg 0.685077**

**069_cam3_1.jpg 096_frontal.jpg 0.554486**

**070_cam3_1.jpg 096_frontal.jpg 0.831683**

**071_cam3_1.jpg 096_frontal.jpg 0.593536**

**072_cam3_1.jpg 014_frontal.jpg 0.391622**

**073_cam3_1.jpg 096_frontal.jpg 0.392358**

**074_cam3_1.jpg 074_frontal.jpg 0.399641**

**075_cam3_1.jpg 089_frontal.jpg 0.415572**

**076_cam3_1.jpg 027_frontal.jpg 0.466936**

**077_cam3_1.jpg 039_frontal.jpg 0.366436**

**078_cam3_1.jpg 096_frontal.jpg 0.595498**

**079_cam3_1.jpg 089_frontal.jpg 0.419773**

**080_cam3_1.jpg 016_frontal.jpg 0.233567**

**081_cam3_1.jpg 049_frontal.jpg 0.424422**

**082_cam3_1.jpg 115_frontal.jpg 0.451077**

**083_cam3_1.jpg 096_frontal.jpg 0.464522**

**084_cam3_1.jpg 115_frontal.jpg 0.479371**

**085_cam3_1.jpg 096_frontal.jpg 0.445646**

**086_cam3_1.jpg 017_frontal.jpg 0.566107**

**087_cam3_1.jpg 115_frontal.jpg 0.441568**

**088_cam3_1.jpg 027_frontal.jpg 0.560938**

**089_cam3_1.jpg 089_frontal.jpg 0.442278**

**090_cam3_1.jpg 059_frontal.jpg 0.599549**

**091_cam3_1.jpg 074_frontal.jpg 0.558958**

**092_cam3_1.jpg 096_frontal.jpg 0.510133**

**093_cam3_1.jpg 093_frontal.jpg 0.445215**

**094_cam3_1.jpg 077_frontal.jpg 0.359330**

**095_cam3_1.jpg 065_frontal.jpg 0.484164**

**096_cam3_1.jpg 019_frontal.jpg 0.595777**

**097_cam3_1.jpg 126_frontal.jpg 0.472714**

**098_cam3_1.jpg 049_frontal.jpg 0.320991**

**099_cam3_1.jpg 101_frontal.jpg 0.373395**

**100_cam3_1.jpg 096_frontal.jpg 0.627991**

**101_cam3_1.jpg 096_frontal.jpg 0.370575**

**102_cam3_1.jpg 077_frontal.jpg 0.562676**

**103_cam3_1.jpg 096_frontal.jpg 0.639859**

**104_cam3_1.jpg 044_frontal.jpg 0.480648**

**105_cam3_1.jpg 096_frontal.jpg 0.608445**

**106_cam3_1.jpg 096_frontal.jpg 0.629554**

**107_cam3_1.jpg 109_frontal.jpg 0.441960**

**108_cam3_1.jpg 096_frontal.jpg 0.458410**

**109_cam3_1.jpg 109_frontal.jpg 0.318038**

**110_cam3_1.jpg 118_frontal.jpg 0.354491**

**111_cam3_1.jpg 027_frontal.jpg 0.382201**

**112_cam3_1.jpg 115_frontal.jpg 0.334275**

**113_cam3_1.jpg 048_frontal.jpg 0.554946**

**114_cam3_1.jpg 044_frontal.jpg 0.514966**

**115_cam3_1.jpg 126_frontal.jpg 0.557517**

**116_cam3_1.jpg 022_frontal.jpg 0.475121**

**117_cam3_1.jpg 115_frontal.jpg 0.484369**

**118_cam3_1.jpg 065_frontal.jpg 0.403478**

**119_cam3_1.jpg 096_frontal.jpg 0.599103**

**120_cam3_1.jpg 078_frontal.jpg 0.370596**

**121_cam3_1.jpg 096_frontal.jpg 0.679545**

**122_cam3_1.jpg 049_frontal.jpg 0.508945**

**123_cam3_1.jpg 012_frontal.jpg 0.545074**

**124_cam3_1.jpg 017_frontal.jpg 0.394161**

**125_cam3_1.jpg 027_frontal.jpg 0.452520**

**126_cam3_1.jpg 032_frontal.jpg 0.498867**

**127_cam3_1.jpg 096_frontal.jpg 0.290095**

**128_cam3_1.jpg 096_frontal.jpg 0.591437**

**129_cam3_1.jpg 126_frontal.jpg 0.478811**

**130_cam3_1.jpg 118_frontal.jpg 0.343334**

**Final Score Scface SSR (Vis) cam3-d2**

**Actual Image Recognized Image Score**

**001_cam3_2.jpg 049_frontal.jpg 0.624772**

**002_cam3_2.jpg 126_frontal.jpg 0.529556**

**003_cam3_2.jpg 049_frontal.jpg 0.450662**

**004_cam3_2.jpg 011_frontal.jpg 0.561849**

**005_cam3_2.jpg 072_frontal.jpg 0.780983**

**006_cam3_2.jpg 091_frontal.jpg 0.605573**

**007_cam3_2.jpg 005_frontal.jpg 0.365725**

**008_cam3_2.jpg 005_frontal.jpg 0.670884**

**009_cam3_2.jpg 104_frontal.jpg 0.589221**

**010_cam3_2.jpg 043_frontal.jpg 0.489958**

**011_cam3_2.jpg 057_frontal.jpg 0.503707**

**012_cam3_2.jpg 096_frontal.jpg 0.532775**

**013_cam3_2.jpg 030_frontal.jpg 0.706564**

**014_cam3_2.jpg 062_frontal.jpg 0.747287**

**015_cam3_2.jpg 103_frontal.jpg 0.543521**

**016_cam3_2.jpg 016_frontal.jpg 0.516026**

**017_cam3_2.jpg 005_frontal.jpg 0.638898**

**018_cam3_2.jpg 049_frontal.jpg 0.509629**

**019_cam3_2.jpg 019_frontal.jpg 0.467468**

**020_cam3_2.jpg 037_frontal.jpg 0.551690**

**021_cam3_2.jpg 084_frontal.jpg 0.609015**

**022_cam3_2.jpg 003_frontal.jpg 0.585258**

**023_cam3_2.jpg 005_frontal.jpg 0.454782**

**024_cam3_2.jpg 047_frontal.jpg 0.542821**

**025_cam3_2.jpg 064_frontal.jpg 0.471720**

**026_cam3_2.jpg 026_frontal.jpg 0.607541**

**027_cam3_2.jpg 126_frontal.jpg 0.559573**

**028_cam3_2.jpg 062_frontal.jpg 0.602506**

**029_cam3_2.jpg 057_frontal.jpg 0.501797**

**030_cam3_2.jpg 006_frontal.jpg 0.511037**

**031_cam3_2.jpg 057_frontal.jpg 0.391526**

**032_cam3_2.jpg 032_frontal.jpg 0.589003**

**033_cam3_2.jpg 011_frontal.jpg 0.399613**

**034_cam3_2.jpg 049_frontal.jpg 0.521709**

**035_cam3_2.jpg 005_frontal.jpg 0.525386**

**036_cam3_2.jpg 031_frontal.jpg 0.574083**

**037_cam3_2.jpg 037_frontal.jpg 0.607046**

**038_cam3_2.jpg 003_frontal.jpg 0.654358**

**039_cam3_2.jpg 089_frontal.jpg 0.599297**

**040_cam3_2.jpg 006_frontal.jpg 0.463566**

**041_cam3_2.jpg 077_frontal.jpg 0.420900**

**042_cam3_2.jpg 030_frontal.jpg 0.408704**

**043_cam3_2.jpg 057_frontal.jpg 0.522888**

**044_cam3_2.jpg 015_frontal.jpg 0.658388**

**045_cam3_2.jpg 108_frontal.jpg 0.362806**

**046_cam3_2.jpg 043_frontal.jpg 0.456384**

**047_cam3_2.jpg 011_frontal.jpg 0.576949**

**048_cam3_2.jpg 048_frontal.jpg 0.614658**

**049_cam3_2.jpg 049_frontal.jpg 0.569380**

**050_cam3_2.jpg 059_frontal.jpg 0.513043**

**051_cam3_2.jpg 016_frontal.jpg 0.496559**

**052_cam3_2.jpg 027_frontal.jpg 0.399045**

**053_cam3_2.jpg 005_frontal.jpg 0.568246**

**054_cam3_2.jpg 049_frontal.jpg 0.477373**

**055_cam3_2.jpg 096_frontal.jpg 0.523907**

**056_cam3_2.jpg 015_frontal.jpg 0.497311**

**057_cam3_2.jpg 124_frontal.jpg 0.434627**

**058_cam3_2.jpg 031_frontal.jpg 0.740501**

**059_cam3_2.jpg 059_frontal.jpg 0.791481**

**060_cam3_2.jpg 071_frontal.jpg 0.578291**

**061_cam3_2.jpg 071_frontal.jpg 0.408541**

**062_cam3_2.jpg 126_frontal.jpg 0.465674**

**063_cam3_2.jpg 063_frontal.jpg 0.745537**

**064_cam3_2.jpg 126_frontal.jpg 0.682629**

**065_cam3_2.jpg 057_frontal.jpg 0.483808**

**066_cam3_2.jpg 089_frontal.jpg 0.640524**

**067_cam3_2.jpg 031_frontal.jpg 0.623568**

**068_cam3_2.jpg 068_frontal.jpg 0.537305**

**069_cam3_2.jpg 126_frontal.jpg 0.496353**

**070_cam3_2.jpg 057_frontal.jpg 0.323604**

**071_cam3_2.jpg 071_frontal.jpg 0.463885**

**072_cam3_2.jpg 059_frontal.jpg 0.521002**

**073_cam3_2.jpg 047_frontal.jpg 0.493685**

**074_cam3_2.jpg 096_frontal.jpg 0.467013**

**075_cam3_2.jpg 047_frontal.jpg 0.630112**

**076_cam3_2.jpg 071_frontal.jpg 0.591857**

**077_cam3_2.jpg 047_frontal.jpg 0.700826**

**078_cam3_2.jpg 105_frontal.jpg 0.447168**

**079_cam3_2.jpg 049_frontal.jpg 0.333464**

**080_cam3_2.jpg 080_frontal.jpg 0.431261**

**081_cam3_2.jpg 019_frontal.jpg 0.446845**

**082_cam3_2.jpg 006_frontal.jpg 0.487908**

**083_cam3_2.jpg 011_frontal.jpg 0.478756**

**084_cam3_2.jpg 096_frontal.jpg 0.627434**

**085_cam3_2.jpg 005_frontal.jpg 0.604133**

**086_cam3_2.jpg 047_frontal.jpg 0.785257**

**087_cam3_2.jpg 096_frontal.jpg 0.582925**

**088_cam3_2.jpg 108_frontal.jpg 0.717451**

**089_cam3_2.jpg 047_frontal.jpg 0.532142**

**090_cam3_2.jpg 047_frontal.jpg 0.684500**

**091_cam3_2.jpg 019_frontal.jpg 0.478316**

**092_cam3_2.jpg 092_frontal.jpg 0.516623**

**093_cam3_2.jpg 003_frontal.jpg 0.649891**

**094_cam3_2.jpg 096_frontal.jpg 0.408289**

**095_cam3_2.jpg 059_frontal.jpg 0.551280**

**096_cam3_2.jpg 047_frontal.jpg 0.596158**

**097_cam3_2.jpg 015_frontal.jpg 0.474950**

**098_cam3_2.jpg 079_frontal.jpg 0.362941**

**099_cam3_2.jpg 050_frontal.jpg 0.766398**

**100_cam3_2.jpg 035_frontal.jpg 0.574503**

**101_cam3_2.jpg 059_frontal.jpg 0.514669**

**102_cam3_2.jpg 027_frontal.jpg 0.510943**

**103_cam3_2.jpg 047_frontal.jpg 0.530055**

**104_cam3_2.jpg 051_frontal.jpg 0.345782**

**105_cam3_2.jpg 042_frontal.jpg 0.358213**

**106_cam3_2.jpg 059_frontal.jpg 0.618059**

**107_cam3_2.jpg 116_frontal.jpg 0.467802**

**108_cam3_2.jpg 127_frontal.jpg 0.417630**

**109_cam3_2.jpg 097_frontal.jpg 0.523129**

**110_cam3_2.jpg 003_frontal.jpg 0.491187**

**111_cam3_2.jpg 126_frontal.jpg 0.457981**

**112_cam3_2.jpg 047_frontal.jpg 0.757835**

**113_cam3_2.jpg 016_frontal.jpg 0.505488**

**114_cam3_2.jpg 108_frontal.jpg 0.399243**

**115_cam3_2.jpg 074_frontal.jpg 0.512566**

**116_cam3_2.jpg 016_frontal.jpg 0.358979**

**117_cam3_2.jpg 019_frontal.jpg 0.386551**

**118_cam3_2.jpg 057_frontal.jpg 0.453905**

**119_cam3_2.jpg 096_frontal.jpg 0.526742**

**120_cam3_2.jpg 120_frontal.jpg 0.665378**

**121_cam3_2.jpg 121_frontal.jpg 0.446349**

**122_cam3_2.jpg 006_frontal.jpg 0.563509**

**123_cam3_2.jpg 071_frontal.jpg 0.614000**

**124_cam3_2.jpg 089_frontal.jpg 0.725174**

**125_cam3_2.jpg 102_frontal.jpg 0.501079**

**126_cam3_2.jpg 126_frontal.jpg 0.583984**

**127_cam3_2.jpg 127_frontal.jpg 0.538060**

**128_cam3_2.jpg 097_frontal.jpg 0.341932**

**129_cam3_2.jpg 047_frontal.jpg 0.715316**

**130_cam3_2.jpg 109_frontal.jpg 0.438587**

**Final Score Scface SSR (Vis) cam3-d3**

**Actual Image Recognized Image Score**

**001_cam3_3.jpg 042_frontal.jpg 0.404638**

**002_cam3_3.jpg 049_frontal.jpg 0.505476**

**003_cam3_3.jpg 003_frontal.jpg 0.353688**

**004_cam3_3.jpg 035_frontal.jpg 0.450903**

**005_cam3_3.jpg 050_frontal.jpg 0.510645**

**006_cam3_3.jpg 121_frontal.jpg 0.521763**

**007_cam3_3.jpg 050_frontal.jpg 0.503595**

**008_cam3_3.jpg 008_frontal.jpg 0.892271**

**009_cam3_3.jpg 057_frontal.jpg 0.354422**

**010_cam3_3.jpg 019_frontal.jpg 0.481893**

**011_cam3_3.jpg 092_frontal.jpg 0.545938**

**012_cam3_3.jpg 049_frontal.jpg 0.445606**

**013_cam3_3.jpg 006_frontal.jpg 0.387197**

**014_cam3_3.jpg 014_frontal.jpg 0.445989**

**015_cam3_3.jpg 059_frontal.jpg 0.554026**

**016_cam3_3.jpg 096_frontal.jpg 0.459402**

**017_cam3_3.jpg 096_frontal.jpg 0.462448**

**018_cam3_3.jpg 047_frontal.jpg 0.493496**

**019_cam3_3.jpg 058_frontal.jpg 0.332689**

**020_cam3_3.jpg 089_frontal.jpg 0.754074**

**021_cam3_3.jpg 053_frontal.jpg 0.413397**

**022_cam3_3.jpg 049_frontal.jpg 0.396440**

**023_cam3_3.jpg 003_frontal.jpg 0.435909**

**024_cam3_3.jpg 024_frontal.jpg 0.337513**

**025_cam3_3.jpg 014_frontal.jpg 0.545763**

**026_cam3_3.jpg 105_frontal.jpg 0.367774**

**027_cam3_3.jpg 094_frontal.jpg 0.446320**

**028_cam3_3.jpg 015_frontal.jpg 0.348101**

**029_cam3_3.jpg 057_frontal.jpg 0.436737**

**030_cam3_3.jpg 126_frontal.jpg 0.500480**

**031_cam3_3.jpg 005_frontal.jpg 0.431003**

**032_cam3_3.jpg 126_frontal.jpg 0.445449**

**033_cam3_3.jpg 005_frontal.jpg 0.462136**

**034_cam3_3.jpg 040_frontal.jpg 0.356038**

**035_cam3_3.jpg 053_frontal.jpg 0.358641**

**036_cam3_3.jpg 097_frontal.jpg 0.340649**

**037_cam3_3.jpg 035_frontal.jpg 0.604236**

**038_cam3_3.jpg 109_frontal.jpg 0.578777**

**039_cam3_3.jpg 053_frontal.jpg 0.446404**

**040_cam3_3.jpg 047_frontal.jpg 0.405425**

**041_cam3_3.jpg 003_frontal.jpg 0.543545**

**042_cam3_3.jpg 096_frontal.jpg 0.451177**

**043_cam3_3.jpg 057_frontal.jpg 0.730029**

**044_cam3_3.jpg 059_frontal.jpg 0.476683**

**045_cam3_3.jpg 097_frontal.jpg 0.393173**

**046_cam3_3.jpg 059_frontal.jpg 0.436827**

**047_cam3_3.jpg 109_frontal.jpg 0.479092**

**048_cam3_3.jpg 096_frontal.jpg 0.580582**

**049_cam3_3.jpg 032_frontal.jpg 0.360271**

**050_cam3_3.jpg 031_frontal.jpg 0.327042**

**051_cam3_3.jpg 059_frontal.jpg 0.450984**

**052_cam3_3.jpg 071_frontal.jpg 0.448708**

**053_cam3_3.jpg 096_frontal.jpg 0.591726**

**054_cam3_3.jpg 040_frontal.jpg 0.466586**

**055_cam3_3.jpg 012_frontal.jpg 0.455585**

**056_cam3_3.jpg 058_frontal.jpg 0.458263**

**057_cam3_3.jpg 041_frontal.jpg 0.483022**

**058_cam3_3.jpg 116_frontal.jpg 0.631294**

**059_cam3_3.jpg 117_frontal.jpg 0.455670**

**060_cam3_3.jpg 066_frontal.jpg 0.420798**

**061_cam3_3.jpg 047_frontal.jpg 0.335123**

**062_cam3_3.jpg 062_frontal.jpg 0.506099**

**063_cam3_3.jpg 063_frontal.jpg 0.463313**

**064_cam3_3.jpg 064_frontal.jpg 0.504957**

**065_cam3_3.jpg 030_frontal.jpg 0.408298**

**066_cam3_3.jpg 050_frontal.jpg 0.548016**

**067_cam3_3.jpg 096_frontal.jpg 0.283514**

**068_cam3_3.jpg 036_frontal.jpg 0.602673**

**069_cam3_3.jpg 003_frontal.jpg 0.550133**

**070_cam3_3.jpg 096_frontal.jpg 0.448870**

**071_cam3_3.jpg 059_frontal.jpg 0.530670**

**072_cam3_3.jpg 059_frontal.jpg 0.444911**

**073_cam3_3.jpg 005_frontal.jpg 0.326663**

**074_cam3_3.jpg 096_frontal.jpg 0.397266**

**075_cam3_3.jpg 019_frontal.jpg 0.423972**

**076_cam3_3.jpg 005_frontal.jpg 0.420881**

**077_cam3_3.jpg 047_frontal.jpg 0.564999**

**078_cam3_3.jpg 082_frontal.jpg 0.445587**

**079_cam3_3.jpg 016_frontal.jpg 0.511619**

**080_cam3_3.jpg 096_frontal.jpg 0.401500**

**081_cam3_3.jpg 124_frontal.jpg 0.441871**

**082_cam3_3.jpg 059_frontal.jpg 0.542603**

**083_cam3_3.jpg 057_frontal.jpg 0.273668**

**084_cam3_3.jpg 071_frontal.jpg 0.578253**

**085_cam3_3.jpg 059_frontal.jpg 0.501280**

**086_cam3_3.jpg 086_frontal.jpg 0.442571**

**087_cam3_3.jpg 118_frontal.jpg 0.292797**

**088_cam3_3.jpg 042_frontal.jpg 0.357192**

**089_cam3_3.jpg 096_frontal.jpg 0.395862**

**090_cam3_3.jpg 103_frontal.jpg 0.385924**

**091_cam3_3.jpg 035_frontal.jpg 0.403075**

**092_cam3_3.jpg 092_frontal.jpg 0.468944**

**093_cam3_3.jpg 032_frontal.jpg 0.579662**

**094_cam3_3.jpg 003_frontal.jpg 0.475080**

**095_cam3_3.jpg 047_frontal.jpg 0.567015**

**096_cam3_3.jpg 071_frontal.jpg 0.534976**

**097_cam3_3.jpg 096_frontal.jpg 0.483530**

**098_cam3_3.jpg 057_frontal.jpg 0.494352**

**099_cam3_3.jpg 003_frontal.jpg 0.438973**

**100_cam3_3.jpg 100_frontal.jpg 0.464423**

**101_cam3_3.jpg 101_frontal.jpg 0.402174**

**102_cam3_3.jpg 102_frontal.jpg 0.441253**

**103_cam3_3.jpg 003_frontal.jpg 0.615109**

**104_cam3_3.jpg 094_frontal.jpg 0.417169**

**105_cam3_3.jpg 071_frontal.jpg 0.495652**

**106_cam3_3.jpg 047_frontal.jpg 0.286170**

**107_cam3_3.jpg 107_frontal.jpg 0.560790**

**108_cam3_3.jpg 059_frontal.jpg 0.614998**

**109_cam3_3.jpg 103_frontal.jpg 0.297729**

**110_cam3_3.jpg 096_frontal.jpg 0.525992**

**111_cam3_3.jpg 049_frontal.jpg 0.538529**

**112_cam3_3.jpg 074_frontal.jpg 0.526107**

**113_cam3_3.jpg 073_frontal.jpg 0.437461**

**114_cam3_3.jpg 074_frontal.jpg 0.393040**

**115_cam3_3.jpg 047_frontal.jpg 0.355008**

**116_cam3_3.jpg 091_frontal.jpg 0.497531**

**117_cam3_3.jpg 126_frontal.jpg 0.351166**

**118_cam3_3.jpg 003_frontal.jpg 0.537419**

**119_cam3_3.jpg 103_frontal.jpg 0.305534**

**120_cam3_3.jpg 120_frontal.jpg 0.642987**

**121_cam3_3.jpg 126_frontal.jpg 0.517689**

**122_cam3_3.jpg 089_frontal.jpg 0.323964**

**123_cam3_3.jpg 038_frontal.jpg 0.363222**

**124_cam3_3.jpg 047_frontal.jpg 0.517570**

**125_cam3_3.jpg 027_frontal.jpg 0.516862**

**126_cam3_3.jpg 096_frontal.jpg 0.697761**

**127_cam3_3.jpg 071_frontal.jpg 0.398438**

**128_cam3_3.jpg 057_frontal.jpg 0.323242**

**129_cam3_3.jpg 019_frontal.jpg 0.555349**

**130_cam3_3.jpg 096_frontal.jpg 0.519817**

**Final Score Scface SSR (Vis) cam4-d1**

**Actual Image Recognized Image Score**

**001_cam4_1.jpg 058_frontal.jpg 0.450887**

**002_cam4_1.jpg 108_frontal.jpg 0.486550**

**003_cam4_1.jpg 037_frontal.jpg 0.440410**

**004_cam4_1.jpg 059_frontal.jpg 0.700980**

**005_cam4_1.jpg 096_frontal.jpg 0.466509**

**006_cam4_1.jpg 036_frontal.jpg 0.464917**

**007_cam4_1.jpg 099_frontal.jpg 0.878031**

**008_cam4_1.jpg 117_frontal.jpg 0.529998**

**009_cam4_1.jpg 047_frontal.jpg 0.644876**

**010_cam4_1.jpg 096_frontal.jpg 0.571756**

**011_cam4_1.jpg 115_frontal.jpg 0.438042**

**012_cam4_1.jpg 115_frontal.jpg 0.467569**

**013_cam4_1.jpg 077_frontal.jpg 0.431751**

**014_cam4_1.jpg 014_frontal.jpg 0.700075**

**015_cam4_1.jpg 015_frontal.jpg 0.793762**

**016_cam4_1.jpg 047_frontal.jpg 0.791377**

**017_cam4_1.jpg 096_frontal.jpg 0.507173**

**018_cam4_1.jpg 042_frontal.jpg 0.470021**

**019_cam4_1.jpg 077_frontal.jpg 0.773246**

**020_cam4_1.jpg 058_frontal.jpg 0.481478**

**021_cam4_1.jpg 050_frontal.jpg 0.380934**

**022_cam4_1.jpg 045_frontal.jpg 0.485460**

**023_cam4_1.jpg 096_frontal.jpg 0.555236**

**024_cam4_1.jpg 047_frontal.jpg 0.772656**

**025_cam4_1.jpg 100_frontal.jpg 0.582715**

**026_cam4_1.jpg 116_frontal.jpg 0.697472**

**027_cam4_1.jpg 027_frontal.jpg 0.607759**

**028_cam4_1.jpg 117_frontal.jpg 0.533025**

**029_cam4_1.jpg 077_frontal.jpg 0.634969**

**030_cam4_1.jpg 044_frontal.jpg 0.415207**

**031_cam4_1.jpg 005_frontal.jpg 0.674618**

**032_cam4_1.jpg 027_frontal.jpg 0.532171**

**033_cam4_1.jpg 109_frontal.jpg 0.611756**

**034_cam4_1.jpg 088_frontal.jpg 0.629727**

**035_cam4_1.jpg 055_frontal.jpg 0.350843**

**036_cam4_1.jpg 042_frontal.jpg 0.560136**

**037_cam4_1.jpg 115_frontal.jpg 0.606194**

**038_cam4_1.jpg 096_frontal.jpg 0.640408**

**039_cam4_1.jpg 005_frontal.jpg 0.553890**

**040_cam4_1.jpg 044_frontal.jpg 0.522153**

**041_cam4_1.jpg 096_frontal.jpg 0.575390**

**042_cam4_1.jpg 044_frontal.jpg 0.457712**

**043_cam4_1.jpg 005_frontal.jpg 0.543189**

**044_cam4_1.jpg 096_frontal.jpg 0.469628**

**045_cam4_1.jpg 005_frontal.jpg 0.387310**

**046_cam4_1.jpg 059_frontal.jpg 0.520001**

**047_cam4_1.jpg 047_frontal.jpg 0.609061**

**048_cam4_1.jpg 116_frontal.jpg 0.796842**

**049_cam4_1.jpg 058_frontal.jpg 0.487430**

**050_cam4_1.jpg 115_frontal.jpg 0.552587**

**051_cam4_1.jpg 105_frontal.jpg 0.621152**

**052_cam4_1.jpg 115_frontal.jpg 0.369842**

**053_cam4_1.jpg 005_frontal.jpg 0.738975**

**054_cam4_1.jpg 027_frontal.jpg 0.467067**

**055_cam4_1.jpg 055_frontal.jpg 0.455940**

**056_cam4_1.jpg 005_frontal.jpg 0.633179**

**057_cam4_1.jpg 005_frontal.jpg 0.532883**

**058_cam4_1.jpg 096_frontal.jpg 0.605874**

**059_cam4_1.jpg 044_frontal.jpg 0.712578**

**060_cam4_1.jpg 049_frontal.jpg 0.575259**

**061_cam4_1.jpg 096_frontal.jpg 0.569250**

**062_cam4_1.jpg 058_frontal.jpg 0.576132**

**063_cam4_1.jpg 063_frontal.jpg 0.517233**

**064_cam4_1.jpg 117_frontal.jpg 0.788528**

**065_cam4_1.jpg 078_frontal.jpg 0.486410**

**066_cam4_1.jpg 127_frontal.jpg 0.599737**

**067_cam4_1.jpg 005_frontal.jpg 0.418025**

**068_cam4_1.jpg 033_frontal.jpg 0.639544**

**069_cam4_1.jpg 046_frontal.jpg 0.330812**

**070_cam4_1.jpg 058_frontal.jpg 0.557594**

**071_cam4_1.jpg 071_frontal.jpg 0.427640**

**072_cam4_1.jpg 047_frontal.jpg 0.634041**

**073_cam4_1.jpg 044_frontal.jpg 0.562687**

**074_cam4_1.jpg 096_frontal.jpg 0.552504**

**075_cam4_1.jpg 064_frontal.jpg 0.519441**

**076_cam4_1.jpg 047_frontal.jpg 0.480637**

**077_cam4_1.jpg 053_frontal.jpg 0.518118**

**078_cam4_1.jpg 005_frontal.jpg 0.581410**

**079_cam4_1.jpg 096_frontal.jpg 0.568670**

**080_cam4_1.jpg 025_frontal.jpg 0.311910**

**081_cam4_1.jpg 053_frontal.jpg 0.462234**

**082_cam4_1.jpg 047_frontal.jpg 0.618145**

**083_cam4_1.jpg 089_frontal.jpg 0.531609**

**084_cam4_1.jpg 047_frontal.jpg 0.568778**

**085_cam4_1.jpg 032_frontal.jpg 0.417201**

**086_cam4_1.jpg 005_frontal.jpg 0.543008**

**087_cam4_1.jpg 032_frontal.jpg 0.575879**

**088_cam4_1.jpg 103_frontal.jpg 0.430172**

**089_cam4_1.jpg 047_frontal.jpg 0.620867**

**090_cam4_1.jpg 097_frontal.jpg 0.475463**

**091_cam4_1.jpg 059_frontal.jpg 0.653471**

**092_cam4_1.jpg 005_frontal.jpg 0.653517**

**093_cam4_1.jpg 059_frontal.jpg 0.626345**

**094_cam4_1.jpg 074_frontal.jpg 0.554319**

**095_cam4_1.jpg 023_frontal.jpg 0.397010**

**096_cam4_1.jpg 096_frontal.jpg 0.523667**

**097_cam4_1.jpg 092_frontal.jpg 0.497731**

**098_cam4_1.jpg 005_frontal.jpg 0.926353**

**099_cam4_1.jpg 099_frontal.jpg 0.531093**

**100_cam4_1.jpg 004_frontal.jpg 0.547286**

**101_cam4_1.jpg 047_frontal.jpg 0.499157**

**102_cam4_1.jpg 108_frontal.jpg 0.541492**

**103_cam4_1.jpg 047_frontal.jpg 0.525039**

**104_cam4_1.jpg 025_frontal.jpg 0.535977**

**105_cam4_1.jpg 092_frontal.jpg 0.465653**

**106_cam4_1.jpg 059_frontal.jpg 0.500359**

**107_cam4_1.jpg 107_frontal.jpg 0.365075**

**108_cam4_1.jpg 053_frontal.jpg 0.481560**

**109_cam4_1.jpg 058_frontal.jpg 0.486672**

**110_cam4_1.jpg 126_frontal.jpg 0.749611**

**111_cam4_1.jpg 115_frontal.jpg 0.564981**

**112_cam4_1.jpg 105_frontal.jpg 0.754177**

**113_cam4_1.jpg 025_frontal.jpg 0.386724**

**114_cam4_1.jpg 105_frontal.jpg 0.530847**

**115_cam4_1.jpg 044_frontal.jpg 0.524366**

**116_cam4_1.jpg 022_frontal.jpg 0.433051**

**117_cam4_1.jpg 044_frontal.jpg 0.520276**

**118_cam4_1.jpg 047_frontal.jpg 0.582211**

**119_cam4_1.jpg 047_frontal.jpg 0.526210**

**120_cam4_1.jpg 016_frontal.jpg 0.478332**

**121_cam4_1.jpg 044_frontal.jpg 0.561007**

**122_cam4_1.jpg 033_frontal.jpg 0.515634**

**123_cam4_1.jpg 100_frontal.jpg 0.403013**

**124_cam4_1.jpg 097_frontal.jpg 0.417873**

**125_cam4_1.jpg 027_frontal.jpg 0.549838**

**126_cam4_1.jpg 051_frontal.jpg 0.473595**

**127_cam4_1.jpg 025_frontal.jpg 0.426063**

**128_cam4_1.jpg 044_frontal.jpg 0.569332**

**129_cam4_1.jpg 126_frontal.jpg 0.454935**

**130_cam4_1.jpg 033_frontal.jpg 0.511969**

**Final Score Scface SSR (Vis) cam4-d2**

**Actual Image Recognized Image Score**

**001_cam4_2.jpg 117_frontal.jpg 0.471416**

**002_cam4_2.jpg 071_frontal.jpg 0.582497**

**003_cam4_2.jpg 096_frontal.jpg 0.384223**

**004_cam4_2.jpg 011_frontal.jpg 0.408854**

**005_cam4_2.jpg 064_frontal.jpg 0.372244**

**006_cam4_2.jpg 096_frontal.jpg 0.446593**

**007_cam4_2.jpg 049_frontal.jpg 0.522445**

**008_cam4_2.jpg 008_frontal.jpg 0.674086**

**009_cam4_2.jpg 104_frontal.jpg 0.379598**

**010_cam4_2.jpg 015_frontal.jpg 0.375422**

**011_cam4_2.jpg 011_frontal.jpg 0.611766**

**012_cam4_2.jpg 019_frontal.jpg 0.447453**

**013_cam4_2.jpg 013_frontal.jpg 0.370489**

**014_cam4_2.jpg 127_frontal.jpg 0.581119**

**015_cam4_2.jpg 047_frontal.jpg 0.360468**

**016_cam4_2.jpg 049_frontal.jpg 0.519400**

**017_cam4_2.jpg 049_frontal.jpg 0.542090**

**018_cam4_2.jpg 049_frontal.jpg 0.420120**

**019_cam4_2.jpg 036_frontal.jpg 0.553522**

**020_cam4_2.jpg 042_frontal.jpg 0.602445**

**021_cam4_2.jpg 084_frontal.jpg 0.677131**

**022_cam4_2.jpg 059_frontal.jpg 0.488175**

**023_cam4_2.jpg 069_frontal.jpg 0.499460**

**024_cam4_2.jpg 059_frontal.jpg 0.487611**

**025_cam4_2.jpg 025_frontal.jpg 0.452654**

**026_cam4_2.jpg 026_frontal.jpg 0.643831**

**027_cam4_2.jpg 043_frontal.jpg 0.551376**

**028_cam4_2.jpg 025_frontal.jpg 0.421902**

**029_cam4_2.jpg 071_frontal.jpg 0.575336**

**030_cam4_2.jpg 005_frontal.jpg 0.519842**

**031_cam4_2.jpg 053_frontal.jpg 0.290511**

**032_cam4_2.jpg 032_frontal.jpg 0.353911**

**033_cam4_2.jpg 049_frontal.jpg 0.460655**

**034_cam4_2.jpg 030_frontal.jpg 0.502326**

**035_cam4_2.jpg 035_frontal.jpg 0.398278**

**036_cam4_2.jpg 071_frontal.jpg 0.560315**

**037_cam4_2.jpg 037_frontal.jpg 0.477527**

**038_cam4_2.jpg 116_frontal.jpg 0.595315**

**039_cam4_2.jpg 011_frontal.jpg 0.473237**

**040_cam4_2.jpg 016_frontal.jpg 0.445771**

**041_cam4_2.jpg 019_frontal.jpg 0.356277**

**042_cam4_2.jpg 126_frontal.jpg 0.480651**

**043_cam4_2.jpg 097_frontal.jpg 0.416015**

**044_cam4_2.jpg 059_frontal.jpg 0.522782**

**045_cam4_2.jpg 129_frontal.jpg 0.358795**

**046_cam4_2.jpg 059_frontal.jpg 0.629202**

**047_cam4_2.jpg 047_frontal.jpg 0.443735**

**048_cam4_2.jpg 059_frontal.jpg 0.640733**

**049_cam4_2.jpg 005_frontal.jpg 0.453620**

**050_cam4_2.jpg 059_frontal.jpg 0.563494**

**051_cam4_2.jpg 112_frontal.jpg 0.433734**

**052_cam4_2.jpg 071_frontal.jpg 0.587780**

**053_cam4_2.jpg 037_frontal.jpg 0.690999**

**054_cam4_2.jpg 016_frontal.jpg 0.412727**

**055_cam4_2.jpg 096_frontal.jpg 0.458632**

**056_cam4_2.jpg 047_frontal.jpg 0.460176**

**057_cam4_2.jpg 003_frontal.jpg 0.357487**

**058_cam4_2.jpg 049_frontal.jpg 0.629100**

**059_cam4_2.jpg 047_frontal.jpg 0.485311**

**060_cam4_2.jpg 049_frontal.jpg 0.693837**

**061_cam4_2.jpg 050_frontal.jpg 0.356344**

**062_cam4_2.jpg 062_frontal.jpg 0.721854**

**063_cam4_2.jpg 063_frontal.jpg 0.615838**

**064_cam4_2.jpg 019_frontal.jpg 0.639044**

**065_cam4_2.jpg 065_frontal.jpg 0.440678**

**066_cam4_2.jpg 066_frontal.jpg 0.434762**

**067_cam4_2.jpg 047_frontal.jpg 0.495980**

**068_cam4_2.jpg 099_frontal.jpg 0.599019**

**069_cam4_2.jpg 051_frontal.jpg 0.417750**

**070_cam4_2.jpg 057_frontal.jpg 0.468854**

**071_cam4_2.jpg 071_frontal.jpg 0.583311**

**072_cam4_2.jpg 042_frontal.jpg 0.511454**

**073_cam4_2.jpg 071_frontal.jpg 0.502549**

**074_cam4_2.jpg 064_frontal.jpg 0.419509**

**075_cam4_2.jpg 075_frontal.jpg 0.533378**

**076_cam4_2.jpg 049_frontal.jpg 0.396588**

**077_cam4_2.jpg 049_frontal.jpg 0.389160**

**078_cam4_2.jpg 096_frontal.jpg 0.378699**

**079_cam4_2.jpg 064_frontal.jpg 0.359986**

**080_cam4_2.jpg 071_frontal.jpg 0.516081**

**081_cam4_2.jpg 074_frontal.jpg 0.335825**

**082_cam4_2.jpg 003_frontal.jpg 0.482583**

**083_cam4_2.jpg 079_frontal.jpg 0.301534**

**084_cam4_2.jpg 096_frontal.jpg 0.515425**

**085_cam4_2.jpg 085_frontal.jpg 0.514111**

**086_cam4_2.jpg 108_frontal.jpg 0.356251**

**087_cam4_2.jpg 043_frontal.jpg 0.301355**

**088_cam4_2.jpg 112_frontal.jpg 0.426543**

**089_cam4_2.jpg 049_frontal.jpg 0.409719**

**090_cam4_2.jpg 097_frontal.jpg 0.387180**

**091_cam4_2.jpg 022_frontal.jpg 0.460358**

**092_cam4_2.jpg 047_frontal.jpg 0.542017**

**093_cam4_2.jpg 053_frontal.jpg 0.570961**

**094_cam4_2.jpg 057_frontal.jpg 0.566155**

**095_cam4_2.jpg 096_frontal.jpg 0.406129**

**096_cam4_2.jpg 039_frontal.jpg 0.396675**

**097_cam4_2.jpg 097_frontal.jpg 0.586882**

**098_cam4_2.jpg 117_frontal.jpg 0.475287**

**099_cam4_2.jpg 033_frontal.jpg 0.350477**

**100_cam4_2.jpg 016_frontal.jpg 0.637914**

**101_cam4_2.jpg 120_frontal.jpg 0.349440**

**102_cam4_2.jpg 102_frontal.jpg 0.396346**

**103_cam4_2.jpg 096_frontal.jpg 0.597596**

**104_cam4_2.jpg 022_frontal.jpg 0.460407**

**105_cam4_2.jpg 042_frontal.jpg 0.356984**

**106_cam4_2.jpg 112_frontal.jpg 0.332292**

**107_cam4_2.jpg 107_frontal.jpg 0.478667**

**108_cam4_2.jpg 043_frontal.jpg 0.343882**

**109_cam4_2.jpg 005_frontal.jpg 0.462642**

**110_cam4_2.jpg 096_frontal.jpg 0.430963**

**111_cam4_2.jpg 071_frontal.jpg 0.392416**

**112_cam4_2.jpg 057_frontal.jpg 0.643933**

**113_cam4_2.jpg 096_frontal.jpg 0.461402**

**114_cam4_2.jpg 070_frontal.jpg 0.433558**

**115_cam4_2.jpg 115_frontal.jpg 0.544787**

**116_cam4_2.jpg 116_frontal.jpg 0.377053**

**117_cam4_2.jpg 096_frontal.jpg 0.705547**

**118_cam4_2.jpg 057_frontal.jpg 0.448233**

**119_cam4_2.jpg 043_frontal.jpg 0.410851**

**120_cam4_2.jpg 096_frontal.jpg 0.650718**

**121_cam4_2.jpg 071_frontal.jpg 0.297413**

**122_cam4_2.jpg 071_frontal.jpg 0.605488**

**123_cam4_2.jpg 064_frontal.jpg 0.399850**

**124_cam4_2.jpg 005_frontal.jpg 0.503947**

**125_cam4_2.jpg 016_frontal.jpg 0.425052**

**126_cam4_2.jpg 126_frontal.jpg 0.409607**

**127_cam4_2.jpg 047_frontal.jpg 0.345184**

**128_cam4_2.jpg 097_frontal.jpg 0.394684**

**129_cam4_2.jpg 003_frontal.jpg 0.449700**

**130_cam4_2.jpg 096_frontal.jpg 0.504478**

**Final Score Scface SSR (Vis) cam4-d3**

**Actual Image Recognized Image Score**

**001_cam4_3.jpg 096_frontal.jpg 0.289586**

**002_cam4_3.jpg 115_frontal.jpg 0.284826**

**003_cam4_3.jpg 096_frontal.jpg 0.344146**

**004_cam4_3.jpg 096_frontal.jpg 0.415952**

**005_cam4_3.jpg 050_frontal.jpg 0.425809**

**006_cam4_3.jpg 112_frontal.jpg 0.421867**

**007_cam4_3.jpg 050_frontal.jpg 0.369592**

**008_cam4_3.jpg 008_frontal.jpg 0.735306**

**009_cam4_3.jpg 083_frontal.jpg 0.334960**

**010_cam4_3.jpg 125_frontal.jpg 0.376943**

**011_cam4_3.jpg 011_frontal.jpg 0.447088**

**012_cam4_3.jpg 042_frontal.jpg 0.454713**

**013_cam4_3.jpg 083_frontal.jpg 0.420926**

**014_cam4_3.jpg 014_frontal.jpg 0.640512**

**015_cam4_3.jpg 047_frontal.jpg 0.275793**

**016_cam4_3.jpg 016_frontal.jpg 0.334579**

**017_cam4_3.jpg 017_frontal.jpg 0.341872**

**018_cam4_3.jpg 042_frontal.jpg 0.339014**

**019_cam4_3.jpg 083_frontal.jpg 0.259160**

**020_cam4_3.jpg 096_frontal.jpg 0.491472**

**021_cam4_3.jpg 099_frontal.jpg 0.263147**

**022_cam4_3.jpg 014_frontal.jpg 0.395714**

**023_cam4_3.jpg 038_frontal.jpg 0.287138**

**024_cam4_3.jpg 049_frontal.jpg 0.337226**

**025_cam4_3.jpg 025_frontal.jpg 0.380372**

**026_cam4_3.jpg 042_frontal.jpg 0.233869**

**027_cam4_3.jpg 012_frontal.jpg 0.416001**

**028_cam4_3.jpg 016_frontal.jpg 0.261164**

**029_cam4_3.jpg 057_frontal.jpg 0.325435**

**030_cam4_3.jpg 042_frontal.jpg 0.318522**

**031_cam4_3.jpg 053_frontal.jpg 0.267110**

**032_cam4_3.jpg 032_frontal.jpg 0.359948**

**033_cam4_3.jpg 096_frontal.jpg 0.395536**

**034_cam4_3.jpg 115_frontal.jpg 0.226930**

**035_cam4_3.jpg 053_frontal.jpg 0.265747**

**036_cam4_3.jpg 097_frontal.jpg 0.345306**

**037_cam4_3.jpg 016_frontal.jpg 0.480194**

**038_cam4_3.jpg 019_frontal.jpg 0.375107**

**039_cam4_3.jpg 115_frontal.jpg 0.312576**

**040_cam4_3.jpg 059_frontal.jpg 0.398310**

**041_cam4_3.jpg 019_frontal.jpg 0.365270**

**042_cam4_3.jpg 096_frontal.jpg 0.370460**

**043_cam4_3.jpg 083_frontal.jpg 0.258479**

**044_cam4_3.jpg 059_frontal.jpg 0.467591**

**045_cam4_3.jpg 045_frontal.jpg 0.329595**

**046_cam4_3.jpg 117_frontal.jpg 0.507943**

**047_cam4_3.jpg 019_frontal.jpg 0.317470**

**048_cam4_3.jpg 059_frontal.jpg 0.404182**

**049_cam4_3.jpg 049_frontal.jpg 0.378291**

**050_cam4_3.jpg 049_frontal.jpg 0.255679**

**051_cam4_3.jpg 075_frontal.jpg 0.323913**

**052_cam4_3.jpg 115_frontal.jpg 0.398020**

**053_cam4_3.jpg 096_frontal.jpg 0.412696**

**054_cam4_3.jpg 101_frontal.jpg 0.277818**

**055_cam4_3.jpg 089_frontal.jpg 0.233127**

**056_cam4_3.jpg 012_frontal.jpg 0.259965**

**057_cam4_3.jpg 071_frontal.jpg 0.283364**

**058_cam4_3.jpg 059_frontal.jpg 0.415216**

**059_cam4_3.jpg 059_frontal.jpg 0.309849**

**060_cam4_3.jpg 049_frontal.jpg 0.417098**

**061_cam4_3.jpg 049_frontal.jpg 0.249206**

**062_cam4_3.jpg 062_frontal.jpg 0.587287**

**063_cam4_3.jpg 063_frontal.jpg 0.511618**

**064_cam4_3.jpg 064_frontal.jpg 0.458945**

**065_cam4_3.jpg 044_frontal.jpg 0.338762**

**066_cam4_3.jpg 115_frontal.jpg 0.350272**

**067_cam4_3.jpg 016_frontal.jpg 0.261753**

**068_cam4_3.jpg 068_frontal.jpg 0.451249**

**069_cam4_3.jpg 038_frontal.jpg 0.347993**

**070_cam4_3.jpg 096_frontal.jpg 0.408244**

**071_cam4_3.jpg 041_frontal.jpg 0.237542**

**072_cam4_3.jpg 059_frontal.jpg 0.346511**

**073_cam4_3.jpg 096_frontal.jpg 0.290992**

**074_cam4_3.jpg 083_frontal.jpg 0.274446**

**075_cam4_3.jpg 115_frontal.jpg 0.321930**

**076_cam4_3.jpg 074_frontal.jpg 0.252882**

**077_cam4_3.jpg 039_frontal.jpg 0.292628**

**078_cam4_3.jpg 097_frontal.jpg 0.251097**

**079_cam4_3.jpg 039_frontal.jpg 0.245008**

**080_cam4_3.jpg 041_frontal.jpg 0.237450**

**081_cam4_3.jpg 096_frontal.jpg 0.281842**

**082_cam4_3.jpg 059_frontal.jpg 0.406341**

**083_cam4_3.jpg 096_frontal.jpg 0.385053**

**084_cam4_3.jpg 096_frontal.jpg 0.347711**

**085_cam4_3.jpg 062_frontal.jpg 0.342268**

**086_cam4_3.jpg 115_frontal.jpg 0.301326**

**087_cam4_3.jpg 103_frontal.jpg 0.223310**

**088_cam4_3.jpg 096_frontal.jpg 0.373271**

**089_cam4_3.jpg 059_frontal.jpg 0.330235**

**090_cam4_3.jpg 047_frontal.jpg 0.228417**

**091_cam4_3.jpg 074_frontal.jpg 0.344004**

**092_cam4_3.jpg 115_frontal.jpg 0.295458**

**093_cam4_3.jpg 100_frontal.jpg 0.361658**

**094_cam4_3.jpg 039_frontal.jpg 0.306731**

**095_cam4_3.jpg 059_frontal.jpg 0.335489**

**096_cam4_3.jpg 071_frontal.jpg 0.313789**

**097_cam4_3.jpg 097_frontal.jpg 0.364306**

**098_cam4_3.jpg 117_frontal.jpg 0.404052**

**099_cam4_3.jpg 099_frontal.jpg 0.333811**

**100_cam4_3.jpg 100_frontal.jpg 0.433939**

**101_cam4_3.jpg 099_frontal.jpg 0.246292**

**102_cam4_3.jpg 115_frontal.jpg 0.411054**

**103_cam4_3.jpg 011_frontal.jpg 0.293270**

**104_cam4_3.jpg 059_frontal.jpg 0.303958**

**105_cam4_3.jpg 039_frontal.jpg 0.333813**

**106_cam4_3.jpg 025_frontal.jpg 0.277658**

**107_cam4_3.jpg 107_frontal.jpg 0.274326**

**108_cam4_3.jpg 059_frontal.jpg 0.533024**

**109_cam4_3.jpg 097_frontal.jpg 0.262039**

**110_cam4_3.jpg 096_frontal.jpg 0.311372**

**111_cam4_3.jpg 039_frontal.jpg 0.266176**

**112_cam4_3.jpg 074_frontal.jpg 0.324094**

**113_cam4_3.jpg 099_frontal.jpg 0.276664**

**114_cam4_3.jpg 115_frontal.jpg 0.373431**

**115_cam4_3.jpg 115_frontal.jpg 0.466860**

**116_cam4_3.jpg 003_frontal.jpg 0.457987**

**117_cam4_3.jpg 096_frontal.jpg 0.354278**

**118_cam4_3.jpg 096_frontal.jpg 0.279047**

**119_cam4_3.jpg 074_frontal.jpg 0.268777**

**120_cam4_3.jpg 059_frontal.jpg 0.231601**

**121_cam4_3.jpg 099_frontal.jpg 0.514878**

**122_cam4_3.jpg 038_frontal.jpg 0.318301**

**123_cam4_3.jpg 074_frontal.jpg 0.289346**

**124_cam4_3.jpg 096_frontal.jpg 0.444850**

**125_cam4_3.jpg 003_frontal.jpg 0.472465**

**126_cam4_3.jpg 126_frontal.jpg 0.389225**

**127_cam4_3.jpg 096_frontal.jpg 0.549945**

**128_cam4_3.jpg 083_frontal.jpg 0.336006**

**129_cam4_3.jpg 043_frontal.jpg 0.262343**

**130_cam4_3.jpg 069_frontal.jpg 0.203626**

**Final Score Scface SSR (Vis) cam5-d1**

**Actual Image Recognized Image Score**

**001_cam5_1.jpg 015_frontal.jpg 0.439223**

**002_cam5_1.jpg 077_frontal.jpg 0.496488**

**003_cam5_1.jpg 096_frontal.jpg 0.477979**

**004_cam5_1.jpg 125_frontal.jpg 0.549467**

**005_cam5_1.jpg 022_frontal.jpg 0.396339**

**006_cam5_1.jpg 096_frontal.jpg 0.819894**

**007_cam5_1.jpg 017_frontal.jpg 0.673541**

**008_cam5_1.jpg 098_frontal.jpg 0.629008**

**009_cam5_1.jpg 100_frontal.jpg 0.308862**

**010_cam5_1.jpg 096_frontal.jpg 0.505872**

**011_cam5_1.jpg 096_frontal.jpg 0.520962**

**012_cam5_1.jpg 115_frontal.jpg 0.525048**

**013_cam5_1.jpg 027_frontal.jpg 0.591965**

**014_cam5_1.jpg 117_frontal.jpg 0.734419**

**015_cam5_1.jpg 015_frontal.jpg 0.314211**

**016_cam5_1.jpg 011_frontal.jpg 0.511023**

**017_cam5_1.jpg 096_frontal.jpg 0.307050**

**018_cam5_1.jpg 089_frontal.jpg 0.424795**

**019_cam5_1.jpg 077_frontal.jpg 0.540055**

**020_cam5_1.jpg 096_frontal.jpg 0.409642**

**021_cam5_1.jpg 077_frontal.jpg 0.447484**

**022_cam5_1.jpg 096_frontal.jpg 0.457320**

**023_cam5_1.jpg 022_frontal.jpg 0.518388**

**024_cam5_1.jpg 011_frontal.jpg 0.524837**

**025_cam5_1.jpg 025_frontal.jpg 0.419283**

**026_cam5_1.jpg 026_frontal.jpg 0.353527**

**027_cam5_1.jpg 027_frontal.jpg 0.415410**

**028_cam5_1.jpg 028_frontal.jpg 0.364690**

**029_cam5_1.jpg 085_frontal.jpg 0.497533**

**030_cam5_1.jpg 030_frontal.jpg 0.939698**

**031_cam5_1.jpg 089_frontal.jpg 0.529517**

**032_cam5_1.jpg 100_frontal.jpg 0.411434**

**033_cam5_1.jpg 044_frontal.jpg 0.392950**

**034_cam5_1.jpg 039_frontal.jpg 0.376193**

**035_cam5_1.jpg 115_frontal.jpg 0.653032**

**036_cam5_1.jpg 096_frontal.jpg 0.470429**

**037_cam5_1.jpg 096_frontal.jpg 0.715507**

**038_cam5_1.jpg 096_frontal.jpg 0.520595**

**039_cam5_1.jpg 049_frontal.jpg 0.309337**

**040_cam5_1.jpg 096_frontal.jpg 0.481370**

**041_cam5_1.jpg 096_frontal.jpg 0.373050**

**042_cam5_1.jpg 042_frontal.jpg 0.512626**

**043_cam5_1.jpg 114_frontal.jpg 0.480547**

**044_cam5_1.jpg 078_frontal.jpg 0.435546**

**045_cam5_1.jpg 045_frontal.jpg 0.459823**

**046_cam5_1.jpg 096_frontal.jpg 0.454492**

**047_cam5_1.jpg 115_frontal.jpg 0.715509**

**048_cam5_1.jpg 096_frontal.jpg 0.547510**

**049_cam5_1.jpg 115_frontal.jpg 0.414839**

**050_cam5_1.jpg 049_frontal.jpg 0.479951**

**051_cam5_1.jpg 076_frontal.jpg 0.593248**

**052_cam5_1.jpg 115_frontal.jpg 0.722810**

**053_cam5_1.jpg 096_frontal.jpg 0.746161**

**054_cam5_1.jpg 005_frontal.jpg 0.549692**

**055_cam5_1.jpg 115_frontal.jpg 0.338405**

**056_cam5_1.jpg 096_frontal.jpg 0.718403**

**057_cam5_1.jpg 078_frontal.jpg 0.585096**

**058_cam5_1.jpg 096_frontal.jpg 0.559049**

**059_cam5_1.jpg 078_frontal.jpg 0.760751**

**060_cam5_1.jpg 049_frontal.jpg 0.531987**

**061_cam5_1.jpg 059_frontal.jpg 0.479510**

**062_cam5_1.jpg 062_frontal.jpg 0.554455**

**063_cam5_1.jpg 063_frontal.jpg 0.564687**

**064_cam5_1.jpg 027_frontal.jpg 0.493373**

**065_cam5_1.jpg 099_frontal.jpg 0.482183**

**066_cam5_1.jpg 028_frontal.jpg 0.633496**

**067_cam5_1.jpg 096_frontal.jpg 0.595308**

**068_cam5_1.jpg 097_frontal.jpg 0.665505**

**069_cam5_1.jpg 097_frontal.jpg 0.446309**

**070_cam5_1.jpg 096_frontal.jpg 0.721288**

**071_cam5_1.jpg 071_frontal.jpg 0.272614**

**072_cam5_1.jpg 065_frontal.jpg 0.433388**

**073_cam5_1.jpg 096_frontal.jpg 0.726871**

**074_cam5_1.jpg 096_frontal.jpg 0.462727**

**075_cam5_1.jpg 120_frontal.jpg 0.421308**

**076_cam5_1.jpg 115_frontal.jpg 0.494015**

**077_cam5_1.jpg 078_frontal.jpg 0.458831**

**078_cam5_1.jpg 120_frontal.jpg 0.577659**

**079_cam5_1.jpg 044_frontal.jpg 0.402817**

**080_cam5_1.jpg 096_frontal.jpg 0.551318**

**081_cam5_1.jpg 096_frontal.jpg 0.549661**

**082_cam5_1.jpg 039_frontal.jpg 0.528974**

**083_cam5_1.jpg 083_frontal.jpg 0.371344**

**084_cam5_1.jpg 027_frontal.jpg 0.285508**

**085_cam5_1.jpg 096_frontal.jpg 0.661850**

**086_cam5_1.jpg 118_frontal.jpg 0.337453**

**087_cam5_1.jpg 115_frontal.jpg 0.449746**

**088_cam5_1.jpg 075_frontal.jpg 0.374825**

**089_cam5_1.jpg 015_frontal.jpg 0.525351**

**090_cam5_1.jpg 049_frontal.jpg 0.425505**

**091_cam5_1.jpg 059_frontal.jpg 0.326998**

**092_cam5_1.jpg 101_frontal.jpg 0.388003**

**093_cam5_1.jpg 100_frontal.jpg 0.290256**

**094_cam5_1.jpg 096_frontal.jpg 0.408905**

**095_cam5_1.jpg 059_frontal.jpg 0.646421**

**096_cam5_1.jpg 096_frontal.jpg 0.543643**

**097_cam5_1.jpg 065_frontal.jpg 0.691441**

**098_cam5_1.jpg 059_frontal.jpg 0.371315**

**099_cam5_1.jpg 096_frontal.jpg 0.553482**

**100_cam5_1.jpg 028_frontal.jpg 0.433086**

**101_cam5_1.jpg 017_frontal.jpg 0.632951**

**102_cam5_1.jpg 115_frontal.jpg 0.444269**

**103_cam5_1.jpg 047_frontal.jpg 0.417572**

**104_cam5_1.jpg 049_frontal.jpg 0.479979**

**105_cam5_1.jpg 096_frontal.jpg 0.552274**

**106_cam5_1.jpg 096_frontal.jpg 0.639198**

**107_cam5_1.jpg 062_frontal.jpg 0.487208**

**108_cam5_1.jpg 049_frontal.jpg 0.449481**

**109_cam5_1.jpg 049_frontal.jpg 0.256975**

**110_cam5_1.jpg 062_frontal.jpg 0.624086**

**111_cam5_1.jpg 027_frontal.jpg 0.457707**

**112_cam5_1.jpg 044_frontal.jpg 0.630999**

**113_cam5_1.jpg 113_frontal.jpg 0.617282**

**114_cam5_1.jpg 096_frontal.jpg 0.405886**

**115_cam5_1.jpg 115_frontal.jpg 0.676294**

**116_cam5_1.jpg 074_frontal.jpg 0.362860**

**117_cam5_1.jpg 096_frontal.jpg 0.597111**

**118_cam5_1.jpg 096_frontal.jpg 0.682951**

**119_cam5_1.jpg 096_frontal.jpg 0.572087**

**120_cam5_1.jpg 126_frontal.jpg 0.442596**

**121_cam5_1.jpg 059_frontal.jpg 0.318914**

**122_cam5_1.jpg 049_frontal.jpg 0.500541**

**123_cam5_1.jpg 027_frontal.jpg 0.583415**

**124_cam5_1.jpg 096_frontal.jpg 0.363585**

**125_cam5_1.jpg 005_frontal.jpg 0.653136**

**126_cam5_1.jpg 049_frontal.jpg 0.398247**

**127_cam5_1.jpg 096_frontal.jpg 0.743754**

**128_cam5_1.jpg 005_frontal.jpg 0.338216**

**129_cam5_1.jpg 126_frontal.jpg 0.423639**

**130_cam5_1.jpg 077_frontal.jpg 0.387834**

**Final Score Scface SSR (Vis) cam5-d2**

**Actual Image Recognized Image Score**

**001_cam5_2.jpg 011_frontal.jpg 0.337887**

**002_cam5_2.jpg 126_frontal.jpg 0.580029**

**003_cam5_2.jpg 012_frontal.jpg 0.478685**

**004_cam5_2.jpg 039_frontal.jpg 0.351740**

**005_cam5_2.jpg 120_frontal.jpg 0.473245**

**006_cam5_2.jpg 027_frontal.jpg 0.395583**

**007_cam5_2.jpg 115_frontal.jpg 0.387347**

**008_cam5_2.jpg 008_frontal.jpg 0.698761**

**009_cam5_2.jpg 019_frontal.jpg 0.452805**

**010_cam5_2.jpg 049_frontal.jpg 0.453116**

**011_cam5_2.jpg 011_frontal.jpg 0.432533**

**012_cam5_2.jpg 011_frontal.jpg 0.292884**

**013_cam5_2.jpg 062_frontal.jpg 0.415367**

**014_cam5_2.jpg 062_frontal.jpg 0.838992**

**015_cam5_2.jpg 012_frontal.jpg 0.503321**

**016_cam5_2.jpg 015_frontal.jpg 0.440250**

**017_cam5_2.jpg 049_frontal.jpg 0.467200**

**018_cam5_2.jpg 126_frontal.jpg 0.410251**

**019_cam5_2.jpg 022_frontal.jpg 0.420564**

**020_cam5_2.jpg 042_frontal.jpg 0.564862**

**021_cam5_2.jpg 031_frontal.jpg 0.357004**

**022_cam5_2.jpg 022_frontal.jpg 0.340948**

**023_cam5_2.jpg 016_frontal.jpg 0.350298**

**024_cam5_2.jpg 009_frontal.jpg 0.290801**

**025_cam5_2.jpg 025_frontal.jpg 0.539849**

**026_cam5_2.jpg 062_frontal.jpg 0.550778**

**027_cam5_2.jpg 115_frontal.jpg 0.385869**

**028_cam5_2.jpg 115_frontal.jpg 0.470717**

**029_cam5_2.jpg 096_frontal.jpg 0.559822**

**030_cam5_2.jpg 057_frontal.jpg 0.409615**

**031_cam5_2.jpg 042_frontal.jpg 0.435983**

**032_cam5_2.jpg 096_frontal.jpg 0.461022**

**033_cam5_2.jpg 096_frontal.jpg 0.440984**

**034_cam5_2.jpg 078_frontal.jpg 0.588295**

**035_cam5_2.jpg 115_frontal.jpg 0.473808**

**036_cam5_2.jpg 049_frontal.jpg 0.387877**

**037_cam5_2.jpg 037_frontal.jpg 0.634183**

**038_cam5_2.jpg 096_frontal.jpg 0.425392**

**039_cam5_2.jpg 015_frontal.jpg 0.408048**

**040_cam5_2.jpg 042_frontal.jpg 0.590531**

**041_cam5_2.jpg 077_frontal.jpg 0.278487**

**042_cam5_2.jpg 015_frontal.jpg 0.388634**

**043_cam5_2.jpg 115_frontal.jpg 0.326876**

**044_cam5_2.jpg 062_frontal.jpg 0.407939**

**045_cam5_2.jpg 045_frontal.jpg 0.304795**

**046_cam5_2.jpg 042_frontal.jpg 0.657035**

**047_cam5_2.jpg 026_frontal.jpg 0.324058**

**048_cam5_2.jpg 048_frontal.jpg 0.419557**

**049_cam5_2.jpg 126_frontal.jpg 0.422983**

**050_cam5_2.jpg 071_frontal.jpg 0.254630**

**051_cam5_2.jpg 112_frontal.jpg 0.284147**

**052_cam5_2.jpg 126_frontal.jpg 0.741890**

**053_cam5_2.jpg 107_frontal.jpg 0.460094**

**054_cam5_2.jpg 101_frontal.jpg 0.334129**

**055_cam5_2.jpg 016_frontal.jpg 0.363993**

**056_cam5_2.jpg 015_frontal.jpg 0.477347**

**057_cam5_2.jpg 030_frontal.jpg 0.400385**

**058_cam5_2.jpg 012_frontal.jpg 0.453161**

**059_cam5_2.jpg 053_frontal.jpg 0.406449**

**060_cam5_2.jpg 016_frontal.jpg 0.402701**

**061_cam5_2.jpg 028_frontal.jpg 0.374680**

**062_cam5_2.jpg 062_frontal.jpg 0.615961**

**063_cam5_2.jpg 096_frontal.jpg 0.351506**

**064_cam5_2.jpg 096_frontal.jpg 0.347200**

**065_cam5_2.jpg 013_frontal.jpg 0.483681**

**066_cam5_2.jpg 066_frontal.jpg 0.415173**

**067_cam5_2.jpg 047_frontal.jpg 0.416366**

**068_cam5_2.jpg 068_frontal.jpg 0.522868**

**069_cam5_2.jpg 126_frontal.jpg 0.482346**

**070_cam5_2.jpg 115_frontal.jpg 0.296496**

**071_cam5_2.jpg 071_frontal.jpg 0.318313**

**072_cam5_2.jpg 015_frontal.jpg 0.324059**

**073_cam5_2.jpg 057_frontal.jpg 0.533975**

**074_cam5_2.jpg 016_frontal.jpg 0.382129**

**075_cam5_2.jpg 115_frontal.jpg 0.459411**

**076_cam5_2.jpg 058_frontal.jpg 0.381778**

**077_cam5_2.jpg 115_frontal.jpg 0.537680**

**078_cam5_2.jpg 115_frontal.jpg 0.657365**

**079_cam5_2.jpg 096_frontal.jpg 0.735290**

**080_cam5_2.jpg 115_frontal.jpg 0.412066**

**081_cam5_2.jpg 074_frontal.jpg 0.425915**

**082_cam5_2.jpg 096_frontal.jpg 0.314997**

**083_cam5_2.jpg 112_frontal.jpg 0.438071**

**084_cam5_2.jpg 057_frontal.jpg 0.365559**

**085_cam5_2.jpg 045_frontal.jpg 0.361263**

**086_cam5_2.jpg 011_frontal.jpg 0.408543**

**087_cam5_2.jpg 115_frontal.jpg 0.575299**

**088_cam5_2.jpg 019_frontal.jpg 0.300657**

**089_cam5_2.jpg 115_frontal.jpg 0.401691**

**090_cam5_2.jpg 015_frontal.jpg 0.258044**

**091_cam5_2.jpg 120_frontal.jpg 0.266438**

**092_cam5_2.jpg 003_frontal.jpg 0.380646**

**093_cam5_2.jpg 099_frontal.jpg 0.409686**

**094_cam5_2.jpg 013_frontal.jpg 0.374839**

**095_cam5_2.jpg 042_frontal.jpg 0.384834**

**096_cam5_2.jpg 096_frontal.jpg 0.453132**

**097_cam5_2.jpg 115_frontal.jpg 0.287312**

**098_cam5_2.jpg 013_frontal.jpg 0.582568**

**099_cam5_2.jpg 096_frontal.jpg 0.533264**

**100_cam5_2.jpg 016_frontal.jpg 0.440681**

**101_cam5_2.jpg 096_frontal.jpg 0.296276**

**102_cam5_2.jpg 067_frontal.jpg 0.265756**

**103_cam5_2.jpg 115_frontal.jpg 0.475097**

**104_cam5_2.jpg 049_frontal.jpg 0.330261**

**105_cam5_2.jpg 049_frontal.jpg 0.314124**

**106_cam5_2.jpg 028_frontal.jpg 0.247831**

**107_cam5_2.jpg 107_frontal.jpg 0.418059**

**108_cam5_2.jpg 115_frontal.jpg 0.256594**

**109_cam5_2.jpg 120_frontal.jpg 0.371303**

**110_cam5_2.jpg 078_frontal.jpg 0.361028**

**111_cam5_2.jpg 019_frontal.jpg 0.334399**

**112_cam5_2.jpg 112_frontal.jpg 0.343729**

**113_cam5_2.jpg 113_frontal.jpg 0.359476**

**114_cam5_2.jpg 062_frontal.jpg 0.424332**

**115_cam5_2.jpg 074_frontal.jpg 0.538957**

**116_cam5_2.jpg 083_frontal.jpg 0.476114**

**117_cam5_2.jpg 013_frontal.jpg 0.527009**

**118_cam5_2.jpg 057_frontal.jpg 0.685527**

**119_cam5_2.jpg 022_frontal.jpg 0.403973**

**120_cam5_2.jpg 078_frontal.jpg 0.328057**

**121_cam5_2.jpg 042_frontal.jpg 0.453246**

**122_cam5_2.jpg 005_frontal.jpg 0.261668**

**123_cam5_2.jpg 115_frontal.jpg 0.424992**

**124_cam5_2.jpg 096_frontal.jpg 0.427720**

**125_cam5_2.jpg 047_frontal.jpg 0.364580**

**126_cam5_2.jpg 126_frontal.jpg 0.387130**

**127_cam5_2.jpg 062_frontal.jpg 0.342957**

**128_cam5_2.jpg 028_frontal.jpg 0.399597**

**129_cam5_2.jpg 005_frontal.jpg 0.458815**

**130_cam5_2.jpg 112_frontal.jpg 0.337202**

**Final Score Scface SSR (Vis) cam5-d3**

**Actual Image Recognized Image Score**

**001_cam5_3.jpg 045_frontal.jpg 0.306481**

**002_cam5_3.jpg 115_frontal.jpg 0.374210**

**003_cam5_3.jpg 129_frontal.jpg 0.545449**

**004_cam5_3.jpg 056_frontal.jpg 0.607638**

**005_cam5_3.jpg 122_frontal.jpg 0.500790**

**006_cam5_3.jpg 078_frontal.jpg 0.517921**

**007_cam5_3.jpg 027_frontal.jpg 0.475673**

**008_cam5_3.jpg 008_frontal.jpg 0.695374**

**009_cam5_3.jpg 129_frontal.jpg 0.750438**

**010_cam5_3.jpg 079_frontal.jpg 0.419277**

**011_cam5_3.jpg 096_frontal.jpg 0.654536**

**012_cam5_3.jpg 049_frontal.jpg 0.522889**

**013_cam5_3.jpg 074_frontal.jpg 0.613097**

**014_cam5_3.jpg 014_frontal.jpg 0.528145**

**015_cam5_3.jpg 096_frontal.jpg 0.518205**

**016_cam5_3.jpg 015_frontal.jpg 0.446656**

**017_cam5_3.jpg 042_frontal.jpg 0.543232**

**018_cam5_3.jpg 108_frontal.jpg 0.589316**

**019_cam5_3.jpg 084_frontal.jpg 0.537822**

**020_cam5_3.jpg 056_frontal.jpg 0.692228**

**021_cam5_3.jpg 057_frontal.jpg 0.516456**

**022_cam5_3.jpg 015_frontal.jpg 0.486549**

**023_cam5_3.jpg 101_frontal.jpg 0.416692**

**024_cam5_3.jpg 108_frontal.jpg 0.350983**

**025_cam5_3.jpg 108_frontal.jpg 0.531215**

**026_cam5_3.jpg 070_frontal.jpg 0.465345**

**027_cam5_3.jpg 096_frontal.jpg 0.432454**

**028_cam5_3.jpg 064_frontal.jpg 0.388711**

**029_cam5_3.jpg 022_frontal.jpg 0.546829**

**030_cam5_3.jpg 108_frontal.jpg 0.483920**

**031_cam5_3.jpg 011_frontal.jpg 0.642977**

**032_cam5_3.jpg 015_frontal.jpg 0.391442**

**033_cam5_3.jpg 015_frontal.jpg 0.546552**

**034_cam5_3.jpg 064_frontal.jpg 0.467676**

**035_cam5_3.jpg 129_frontal.jpg 0.476628**

**036_cam5_3.jpg 108_frontal.jpg 0.568664**

**037_cam5_3.jpg 015_frontal.jpg 0.419637**

**038_cam5_3.jpg 027_frontal.jpg 0.587012**

**039_cam5_3.jpg 042_frontal.jpg 0.350545**

**040_cam5_3.jpg 096_frontal.jpg 0.443627**

**041_cam5_3.jpg 112_frontal.jpg 0.430352**

**042_cam5_3.jpg 015_frontal.jpg 0.651545**

**043_cam5_3.jpg 108_frontal.jpg 0.522981**

**044_cam5_3.jpg 059_frontal.jpg 0.438273**

**045_cam5_3.jpg 045_frontal.jpg 0.515381**

**046_cam5_3.jpg 059_frontal.jpg 0.464704**

**047_cam5_3.jpg 047_frontal.jpg 0.578917**

**048_cam5_3.jpg 048_frontal.jpg 0.490674**

**049_cam5_3.jpg 096_frontal.jpg 0.434880**

**050_cam5_3.jpg 108_frontal.jpg 0.399718**

**051_cam5_3.jpg 059_frontal.jpg 0.601017**

**052_cam5_3.jpg 126_frontal.jpg 0.438324**

**053_cam5_3.jpg 053_frontal.jpg 0.588803**

**054_cam5_3.jpg 101_frontal.jpg 0.496819**

**055_cam5_3.jpg 005_frontal.jpg 0.487044**

**056_cam5_3.jpg 011_frontal.jpg 0.521407**

**057_cam5_3.jpg 129_frontal.jpg 0.388373**

**058_cam5_3.jpg 096_frontal.jpg 0.440515**

**059_cam5_3.jpg 059_frontal.jpg 0.558548**

**060_cam5_3.jpg 012_frontal.jpg 0.320361**

**061_cam5_3.jpg 082_frontal.jpg 0.409053**

**062_cam5_3.jpg 062_frontal.jpg 0.480912**

**063_cam5_3.jpg 092_frontal.jpg 0.569059**

**064_cam5_3.jpg 077_frontal.jpg 0.435073**

**065_cam5_3.jpg 129_frontal.jpg 0.403447**

**066_cam5_3.jpg 015_frontal.jpg 0.478578**

**067_cam5_3.jpg 042_frontal.jpg 0.453917**

**068_cam5_3.jpg 027_frontal.jpg 0.640780**

**069_cam5_3.jpg 083_frontal.jpg 0.424121**

**070_cam5_3.jpg 056_frontal.jpg 0.448094**

**071_cam5_3.jpg 112_frontal.jpg 0.384691**

**072_cam5_3.jpg 115_frontal.jpg 0.364726**

**073_cam5_3.jpg 096_frontal.jpg 0.477872**

**074_cam5_3.jpg 099_frontal.jpg 0.500309**

**075_cam5_3.jpg 117_frontal.jpg 0.439440**

**076_cam5_3.jpg 111_frontal.jpg 0.439366**

**077_cam5_3.jpg 047_frontal.jpg 0.484439**

**078_cam5_3.jpg 011_frontal.jpg 0.457382**

**079_cam5_3.jpg 104_frontal.jpg 0.341666**

**080_cam5_3.jpg 040_frontal.jpg 0.439258**

**081_cam5_3.jpg 091_frontal.jpg 0.407906**

**082_cam5_3.jpg 059_frontal.jpg 0.418301**

**083_cam5_3.jpg 129_frontal.jpg 0.526191**

**084_cam5_3.jpg 129_frontal.jpg 0.492183**

**085_cam5_3.jpg 027_frontal.jpg 0.730630**

**086_cam5_3.jpg 011_frontal.jpg 0.401126**

**087_cam5_3.jpg 120_frontal.jpg 0.405068**

**088_cam5_3.jpg 039_frontal.jpg 0.543338**

**089_cam5_3.jpg 058_frontal.jpg 0.300652**

**090_cam5_3.jpg 047_frontal.jpg 0.491948**

**091_cam5_3.jpg 042_frontal.jpg 0.436057**

**092_cam5_3.jpg 042_frontal.jpg 0.595792**

**093_cam5_3.jpg 128_frontal.jpg 0.438432**

**094_cam5_3.jpg 129_frontal.jpg 0.821083**

**095_cam5_3.jpg 059_frontal.jpg 0.542155**

**096_cam5_3.jpg 015_frontal.jpg 0.450893**

**097_cam5_3.jpg 096_frontal.jpg 0.506300**

**098_cam5_3.jpg 096_frontal.jpg 0.512900**

**099_cam5_3.jpg 073_frontal.jpg 0.404718**

**100_cam5_3.jpg 115_frontal.jpg 0.454409**

**101_cam5_3.jpg 108_frontal.jpg 0.465354**

**102_cam5_3.jpg 115_frontal.jpg 0.474292**

**103_cam5_3.jpg 096_frontal.jpg 0.521459**

**104_cam5_3.jpg 104_frontal.jpg 0.509801**

**105_cam5_3.jpg 117_frontal.jpg 0.604964**

**106_cam5_3.jpg 096_frontal.jpg 0.395438**

**107_cam5_3.jpg 011_frontal.jpg 0.433850**

**108_cam5_3.jpg 047_frontal.jpg 0.373452**

**109_cam5_3.jpg 015_frontal.jpg 0.312897**

**110_cam5_3.jpg 096_frontal.jpg 0.515209**

**111_cam5_3.jpg 011_frontal.jpg 0.528774**

**112_cam5_3.jpg 112_frontal.jpg 0.617063**

**113_cam5_3.jpg 112_frontal.jpg 0.550119**

**114_cam5_3.jpg 115_frontal.jpg 0.474292**

**115_cam5_3.jpg 050_frontal.jpg 0.509141**

**116_cam5_3.jpg 112_frontal.jpg 0.464446**

**117_cam5_3.jpg 129_frontal.jpg 0.573946**

**118_cam5_3.jpg 096_frontal.jpg 0.435312**

**119_cam5_3.jpg 015_frontal.jpg 0.523306**

**120_cam5_3.jpg 011_frontal.jpg 0.440460**

**121_cam5_3.jpg 096_frontal.jpg 0.542646**

**122_cam5_3.jpg 101_frontal.jpg 0.331056**

**123_cam5_3.jpg 015_frontal.jpg 0.529569**

**124_cam5_3.jpg 005_frontal.jpg 0.518705**

**125_cam5_3.jpg 033_frontal.jpg 0.423112**

**126_cam5_3.jpg 011_frontal.jpg 0.456070**

**127_cam5_3.jpg 096_frontal.jpg 0.347679**

**128_cam5_3.jpg 057_frontal.jpg 0.408485**

**129_cam5_3.jpg 119_frontal.jpg 0.415835**

**130_cam5_3.jpg 096_frontal.jpg 0.490287**

**Final Score Scface SSR (V-IR) cam6-d1**

**Actual Image Recognized Image Score**

**001_cam6_1.jpg 072_frontal.jpg 0.378007**

**002_cam6_1.jpg 015_frontal.jpg 0.520927**

**003_cam6_1.jpg 015_frontal.jpg 0.541565**

**004_cam6_1.jpg 056_frontal.jpg 0.618208**

**005_cam6_1.jpg 096_frontal.jpg 0.483830**

**006_cam6_1.jpg 046_frontal.jpg 0.517993**

**007_cam6_1.jpg 096_frontal.jpg 0.363807**

**008_cam6_1.jpg 045_frontal.jpg 0.496487**

**009_cam6_1.jpg 077_frontal.jpg 0.508566**

**010_cam6_1.jpg 097_frontal.jpg 0.397064**

**011_cam6_1.jpg 076_frontal.jpg 0.590837**

**012_cam6_1.jpg 065_frontal.jpg 0.406028**

**013_cam6_1.jpg 117_frontal.jpg 0.571780**

**014_cam6_1.jpg 085_frontal.jpg 0.598948**

**015_cam6_1.jpg 015_frontal.jpg 0.524061**

**016_cam6_1.jpg 096_frontal.jpg 0.526051**

**017_cam6_1.jpg 126_frontal.jpg 0.510700**

**018_cam6_1.jpg 046_frontal.jpg 0.611594**

**019_cam6_1.jpg 027_frontal.jpg 0.571581**

**020_cam6_1.jpg 097_frontal.jpg 0.578534**

**021_cam6_1.jpg 015_frontal.jpg 0.735165**

**022_cam6_1.jpg 040_frontal.jpg 0.472646**

**023_cam6_1.jpg 019_frontal.jpg 0.419583**

**024_cam6_1.jpg 077_frontal.jpg 0.380947**

**025_cam6_1.jpg 093_frontal.jpg 0.471012**

**026_cam6_1.jpg 033_frontal.jpg 0.434167**

**027_cam6_1.jpg 027_frontal.jpg 0.403432**

**028_cam6_1.jpg 076_frontal.jpg 0.530291**

**029_cam6_1.jpg 046_frontal.jpg 0.639301**

**030_cam6_1.jpg 099_frontal.jpg 0.629530**

**031_cam6_1.jpg 002_frontal.jpg 0.512326**

**032_cam6_1.jpg 040_frontal.jpg 0.398799**

**033_cam6_1.jpg 077_frontal.jpg 0.588604**

**034_cam6_1.jpg 069_frontal.jpg 0.385238**

**035_cam6_1.jpg 015_frontal.jpg 0.481751**

**036_cam6_1.jpg 056_frontal.jpg 0.597615**

**037_cam6_1.jpg 040_frontal.jpg 0.414712**

**038_cam6_1.jpg 038_frontal.jpg 0.490807**

**039_cam6_1.jpg 078_frontal.jpg 0.321136**

**040_cam6_1.jpg 117_frontal.jpg 0.554422**

**041_cam6_1.jpg 015_frontal.jpg 0.483275**

**042_cam6_1.jpg 065_frontal.jpg 0.398962**

**043_cam6_1.jpg 027_frontal.jpg 0.357740**

**044_cam6_1.jpg 077_frontal.jpg 0.453101**

**045_cam6_1.jpg 045_frontal.jpg 0.454408**

**046_cam6_1.jpg 006_frontal.jpg 0.466850**

**047_cam6_1.jpg 015_frontal.jpg 0.352822**

**048_cam6_1.jpg 096_frontal.jpg 0.564513**

**049_cam6_1.jpg 056_frontal.jpg 0.564502**

**050_cam6_1.jpg 093_frontal.jpg 0.396642**

**051_cam6_1.jpg 076_frontal.jpg 0.433439**

**052_cam6_1.jpg 076_frontal.jpg 0.624433**

**053_cam6_1.jpg 072_frontal.jpg 0.457783**

**054_cam6_1.jpg 089_frontal.jpg 0.506880**

**055_cam6_1.jpg 040_frontal.jpg 0.641162**

**056_cam6_1.jpg 065_frontal.jpg 0.459249**

**057_cam6_1.jpg 100_frontal.jpg 0.567530**

**058_cam6_1.jpg 021_frontal.jpg 0.421421**

**059_cam6_1.jpg 065_frontal.jpg 0.426777**

**060_cam6_1.jpg 072_frontal.jpg 0.435351**

**061_cam6_1.jpg 014_frontal.jpg 0.376259**

**062_cam6_1.jpg 093_frontal.jpg 0.470091**

**063_cam6_1.jpg 063_frontal.jpg 0.475350**

**064_cam6_1.jpg 043_frontal.jpg 0.435903**

**065_cam6_1.jpg 065_frontal.jpg 0.448401**

**066_cam6_1.jpg 016_frontal.jpg 0.654645**

**067_cam6_1.jpg 076_frontal.jpg 0.464907**

**068_cam6_1.jpg 068_frontal.jpg 0.588507**

**069_cam6_1.jpg 006_frontal.jpg 0.382690**

**070_cam6_1.jpg 050_frontal.jpg 0.341384**

**071_cam6_1.jpg 015_frontal.jpg 0.567068**

**072_cam6_1.jpg 076_frontal.jpg 0.502330**

**073_cam6_1.jpg 104_frontal.jpg 0.510268**

**074_cam6_1.jpg 096_frontal.jpg 0.606979**

**075_cam6_1.jpg 015_frontal.jpg 0.478967**

**076_cam6_1.jpg 099_frontal.jpg 0.544909**

**077_cam6_1.jpg 033_frontal.jpg 0.446396**

**078_cam6_1.jpg 068_frontal.jpg 0.545221**

**079_cam6_1.jpg 027_frontal.jpg 0.466906**

**080_cam6_1.jpg 057_frontal.jpg 0.529202**

**081_cam6_1.jpg 046_frontal.jpg 0.493865**

**082_cam6_1.jpg 040_frontal.jpg 0.474252**

**083_cam6_1.jpg 046_frontal.jpg 0.675090**

**084_cam6_1.jpg 021_frontal.jpg 0.579487**

**085_cam6_1.jpg 085_frontal.jpg 0.572488**

**086_cam6_1.jpg 017_frontal.jpg 0.378185**

**087_cam6_1.jpg 093_frontal.jpg 0.444526**

**088_cam6_1.jpg 014_frontal.jpg 0.661046**

**089_cam6_1.jpg 015_frontal.jpg 0.513341**

**090_cam6_1.jpg 099_frontal.jpg 0.506929**

**091_cam6_1.jpg 015_frontal.jpg 0.612455**

**092_cam6_1.jpg 040_frontal.jpg 0.521528**

**093_cam6_1.jpg 087_frontal.jpg 0.501301**

**094_cam6_1.jpg 076_frontal.jpg 0.512156**

**095_cam6_1.jpg 076_frontal.jpg 0.405920**

**096_cam6_1.jpg 046_frontal.jpg 0.664332**

**097_cam6_1.jpg 072_frontal.jpg 0.473609**

**098_cam6_1.jpg 082_frontal.jpg 0.427193**

**099_cam6_1.jpg 109_frontal.jpg 0.409122**

**100_cam6_1.jpg 072_frontal.jpg 0.347792**

**101_cam6_1.jpg 109_frontal.jpg 0.501688**

**102_cam6_1.jpg 015_frontal.jpg 0.466190**

**103_cam6_1.jpg 043_frontal.jpg 0.448474**

**104_cam6_1.jpg 063_frontal.jpg 0.421261**

**105_cam6_1.jpg 117_frontal.jpg 0.699288**

**106_cam6_1.jpg 043_frontal.jpg 0.446661**

**107_cam6_1.jpg 054_frontal.jpg 0.434939**

**108_cam6_1.jpg 096_frontal.jpg 0.409621**

**109_cam6_1.jpg 109_frontal.jpg 0.506476**

**110_cam6_1.jpg 056_frontal.jpg 0.478019**

**111_cam6_1.jpg 040_frontal.jpg 0.436645**

**112_cam6_1.jpg 109_frontal.jpg 0.545561**

**113_cam6_1.jpg 028_frontal.jpg 0.384617**

**114_cam6_1.jpg 096_frontal.jpg 0.375729**

**115_cam6_1.jpg 076_frontal.jpg 0.359639**

**116_cam6_1.jpg 015_frontal.jpg 0.424539**

**117_cam6_1.jpg 077_frontal.jpg 0.413756**

**118_cam6_1.jpg 050_frontal.jpg 0.407279**

**119_cam6_1.jpg 093_frontal.jpg 0.450393**

**120_cam6_1.jpg 100_frontal.jpg 0.522416**

**121_cam6_1.jpg 076_frontal.jpg 0.612974**

**122_cam6_1.jpg 099_frontal.jpg 0.522662**

**123_cam6_1.jpg 046_frontal.jpg 0.636832**

**124_cam6_1.jpg 089_frontal.jpg 0.439046**

**125_cam6_1.jpg 109_frontal.jpg 0.765824**

**126_cam6_1.jpg 027_frontal.jpg 0.397302**

**127_cam6_1.jpg 006_frontal.jpg 0.484249**

**128_cam6_1.jpg 076_frontal.jpg 0.400076**

**129_cam6_1.jpg 006_frontal.jpg 0.388085**

**130_cam6_1.jpg 006_frontal.jpg 0.522930**

**Final Score Scface SSR (V-IR) cam6-d2**

**Actual Image Recognized Image Score**

**001_cam6_2.jpg 077_frontal.jpg 0.334362**

**002_cam6_2.jpg 047_frontal.jpg 0.458239**

**003_cam6_2.jpg 096_frontal.jpg 0.568085**

**004_cam6_2.jpg 076_frontal.jpg 0.501686**

**005_cam6_2.jpg 096_frontal.jpg 0.423394**

**006_cam6_2.jpg 065_frontal.jpg 0.408737**

**007_cam6_2.jpg 025_frontal.jpg 0.436391**

**008_cam6_2.jpg 063_frontal.jpg 0.723647**

**009_cam6_2.jpg 065_frontal.jpg 0.304033**

**010_cam6_2.jpg 015_frontal.jpg 0.288030**

**011_cam6_2.jpg 093_frontal.jpg 0.684245**

**012_cam6_2.jpg 076_frontal.jpg 0.410329**

**013_cam6_2.jpg 093_frontal.jpg 0.513996**

**014_cam6_2.jpg 085_frontal.jpg 0.494984**

**015_cam6_2.jpg 076_frontal.jpg 0.547702**

**016_cam6_2.jpg 042_frontal.jpg 0.472336**

**017_cam6_2.jpg 005_frontal.jpg 0.455176**

**018_cam6_2.jpg 046_frontal.jpg 0.390313**

**019_cam6_2.jpg 076_frontal.jpg 0.517060**

**020_cam6_2.jpg 001_frontal.jpg 0.370774**

**021_cam6_2.jpg 096_frontal.jpg 0.668813**

**022_cam6_2.jpg 006_frontal.jpg 0.618695**

**023_cam6_2.jpg 023_frontal.jpg 0.501686**

**024_cam6_2.jpg 015_frontal.jpg 0.487276**

**025_cam6_2.jpg 025_frontal.jpg 0.408203**

**026_cam6_2.jpg 006_frontal.jpg 0.416350**

**027_cam6_2.jpg 100_frontal.jpg 0.422144**

**028_cam6_2.jpg 040_frontal.jpg 0.589854**

**029_cam6_2.jpg 042_frontal.jpg 0.415947**

**030_cam6_2.jpg 065_frontal.jpg 0.261688**

**031_cam6_2.jpg 042_frontal.jpg 0.632611**

**032_cam6_2.jpg 040_frontal.jpg 0.523015**

**033_cam6_2.jpg 100_frontal.jpg 0.614744**

**034_cam6_2.jpg 015_frontal.jpg 0.401044**

**035_cam6_2.jpg 015_frontal.jpg 0.489288**

**036_cam6_2.jpg 093_frontal.jpg 0.590797**

**037_cam6_2.jpg 037_frontal.jpg 0.532593**

**038_cam6_2.jpg 076_frontal.jpg 0.386432**

**039_cam6_2.jpg 096_frontal.jpg 0.365705**

**040_cam6_2.jpg 064_frontal.jpg 0.307494**

**041_cam6_2.jpg 076_frontal.jpg 0.308332**

**042_cam6_2.jpg 076_frontal.jpg 0.445747**

**043_cam6_2.jpg 093_frontal.jpg 0.322292**

**044_cam6_2.jpg 064_frontal.jpg 0.317247**

**045_cam6_2.jpg 045_frontal.jpg 0.468278**

**046_cam6_2.jpg 076_frontal.jpg 0.373236**

**047_cam6_2.jpg 093_frontal.jpg 0.390211**

**048_cam6_2.jpg 100_frontal.jpg 0.543239**

**049_cam6_2.jpg 015_frontal.jpg 0.453314**

**050_cam6_2.jpg 059_frontal.jpg 0.449251**

**051_cam6_2.jpg 076_frontal.jpg 0.542745**

**052_cam6_2.jpg 093_frontal.jpg 0.337814**

**053_cam6_2.jpg 093_frontal.jpg 0.478153**

**054_cam6_2.jpg 093_frontal.jpg 0.370722**

**055_cam6_2.jpg 096_frontal.jpg 0.604249**

**056_cam6_2.jpg 076_frontal.jpg 0.493606**

**057_cam6_2.jpg 005_frontal.jpg 0.412468**

**058_cam6_2.jpg 112_frontal.jpg 0.502007**

**059_cam6_2.jpg 100_frontal.jpg 0.581399**

**060_cam6_2.jpg 015_frontal.jpg 0.361576**

**061_cam6_2.jpg 093_frontal.jpg 0.771944**

**062_cam6_2.jpg 100_frontal.jpg 0.566055**

**063_cam6_2.jpg 063_frontal.jpg 0.779444**

**064_cam6_2.jpg 064_frontal.jpg 0.537614**

**065_cam6_2.jpg 065_frontal.jpg 0.388150**

**066_cam6_2.jpg 077_frontal.jpg 0.448606**

**067_cam6_2.jpg 076_frontal.jpg 0.411032**

**068_cam6_2.jpg 068_frontal.jpg 0.495647**

**069_cam6_2.jpg 015_frontal.jpg 0.314234**

**070_cam6_2.jpg 072_frontal.jpg 0.307351**

**071_cam6_2.jpg 001_frontal.jpg 0.447054**

**072_cam6_2.jpg 100_frontal.jpg 0.637353**

**073_cam6_2.jpg 104_frontal.jpg 0.498414**

**074_cam6_2.jpg 096_frontal.jpg 0.530467**

**075_cam6_2.jpg 076_frontal.jpg 0.386396**

**076_cam6_2.jpg 096_frontal.jpg 0.565819**

**077_cam6_2.jpg 093_frontal.jpg 0.394175**

**078_cam6_2.jpg 093_frontal.jpg 0.497298**

**079_cam6_2.jpg 096_frontal.jpg 0.304226**

**080_cam6_2.jpg 015_frontal.jpg 0.284132**

**081_cam6_2.jpg 015_frontal.jpg 0.312294**

**082_cam6_2.jpg 096_frontal.jpg 0.476152**

**083_cam6_2.jpg 089_frontal.jpg 0.533396**

**084_cam6_2.jpg 046_frontal.jpg 0.484205**

**085_cam6_2.jpg 042_frontal.jpg 0.470938**

**086_cam6_2.jpg 093_frontal.jpg 0.416238**

**087_cam6_2.jpg 005_frontal.jpg 0.335849**

**088_cam6_2.jpg 093_frontal.jpg 0.442692**

**089_cam6_2.jpg 015_frontal.jpg 0.557422**

**090_cam6_2.jpg 015_frontal.jpg 0.454308**

**091_cam6_2.jpg 076_frontal.jpg 0.446751**

**092_cam6_2.jpg 093_frontal.jpg 0.691362**

**093_cam6_2.jpg 093_frontal.jpg 0.771627**

**094_cam6_2.jpg 040_frontal.jpg 0.457275**

**095_cam6_2.jpg 093_frontal.jpg 0.419137**

**096_cam6_2.jpg 076_frontal.jpg 0.294522**

**097_cam6_2.jpg 042_frontal.jpg 0.391667**

**098_cam6_2.jpg 015_frontal.jpg 0.381194**

**099_cam6_2.jpg 061_frontal.jpg 0.279694**

**100_cam6_2.jpg 100_frontal.jpg 0.563970**

**101_cam6_2.jpg 089_frontal.jpg 0.397779**

**102_cam6_2.jpg 046_frontal.jpg 0.361101**

**103_cam6_2.jpg 047_frontal.jpg 0.397680**

**104_cam6_2.jpg 008_frontal.jpg 0.298465**

**105_cam6_2.jpg 093_frontal.jpg 0.354815**

**106_cam6_2.jpg 005_frontal.jpg 0.237236**

**107_cam6_2.jpg 050_frontal.jpg 0.325867**

**108_cam6_2.jpg 015_frontal.jpg 0.325466**

**109_cam6_2.jpg 009_frontal.jpg 0.480048**

**110_cam6_2.jpg 089_frontal.jpg 0.358567**

**111_cam6_2.jpg 093_frontal.jpg 0.529180**

**112_cam6_2.jpg 039_frontal.jpg 0.426621**

**113_cam6_2.jpg 113_frontal.jpg 0.474889**

**114_cam6_2.jpg 061_frontal.jpg 0.587212**

**115_cam6_2.jpg 093_frontal.jpg 0.547076**

**116_cam6_2.jpg 099_frontal.jpg 0.414525**

**117_cam6_2.jpg 096_frontal.jpg 0.491338**

**118_cam6_2.jpg 063_frontal.jpg 0.322156**

**119_cam6_2.jpg 076_frontal.jpg 0.363800**

**120_cam6_2.jpg 015_frontal.jpg 0.359150**

**121_cam6_2.jpg 089_frontal.jpg 0.399609**

**122_cam6_2.jpg 042_frontal.jpg 0.462371**

**123_cam6_2.jpg 093_frontal.jpg 0.577777**

**124_cam6_2.jpg 017_frontal.jpg 0.453562**

**125_cam6_2.jpg 005_frontal.jpg 0.410762**

**126_cam6_2.jpg 025_frontal.jpg 0.323572**

**127_cam6_2.jpg 093_frontal.jpg 0.630463**

**128_cam6_2.jpg 093_frontal.jpg 0.321687**

**129_cam6_2.jpg 076_frontal.jpg 0.406961**

**130_cam6_2.jpg 015_frontal.jpg 0.476598**

**Final Score Scface SSR (V-IR) cam6-d3**

**Actual Image Recognized Image Score**

**001_cam6_3.jpg 042_frontal.jpg 0.491778**

**002_cam6_3.jpg 011_frontal.jpg 0.451848**

**003_cam6_3.jpg 011_frontal.jpg 0.579717**

**004_cam6_3.jpg 042_frontal.jpg 0.390051**

**005_cam6_3.jpg 050_frontal.jpg 0.366459**

**006_cam6_3.jpg 076_frontal.jpg 0.415794**

**007_cam6_3.jpg 025_frontal.jpg 0.485893**

**008_cam6_3.jpg 008_frontal.jpg 0.738312**

**009_cam6_3.jpg 046_frontal.jpg 0.335650**

**010_cam6_3.jpg 006_frontal.jpg 0.532908**

**011_cam6_3.jpg 011_frontal.jpg 0.637817**

**012_cam6_3.jpg 011_frontal.jpg 0.505659**

**013_cam6_3.jpg 027_frontal.jpg 0.409388**

**014_cam6_3.jpg 014_frontal.jpg 0.546207**

**015_cam6_3.jpg 042_frontal.jpg 0.489494**

**016_cam6_3.jpg 012_frontal.jpg 0.553778**

**017_cam6_3.jpg 042_frontal.jpg 0.567371**

**018_cam6_3.jpg 027_frontal.jpg 0.396776**

**019_cam6_3.jpg 011_frontal.jpg 0.410213**

**020_cam6_3.jpg 011_frontal.jpg 0.430888**

**021_cam6_3.jpg 005_frontal.jpg 0.412864**

**022_cam6_3.jpg 042_frontal.jpg 0.392948**

**023_cam6_3.jpg 004_frontal.jpg 0.388249**

**024_cam6_3.jpg 074_frontal.jpg 0.289250**

**025_cam6_3.jpg 025_frontal.jpg 0.587083**

**026_cam6_3.jpg 026_frontal.jpg 0.393301**

**027_cam6_3.jpg 011_frontal.jpg 0.567647**

**028_cam6_3.jpg 042_frontal.jpg 0.484626**

**029_cam6_3.jpg 041_frontal.jpg 0.316103**

**030_cam6_3.jpg 006_frontal.jpg 0.608288**

**031_cam6_3.jpg 042_frontal.jpg 0.513760**

**032_cam6_3.jpg 004_frontal.jpg 0.330818**

**033_cam6_3.jpg 042_frontal.jpg 0.419823**

**034_cam6_3.jpg 027_frontal.jpg 0.378160**

**035_cam6_3.jpg 046_frontal.jpg 0.364722**

**036_cam6_3.jpg 042_frontal.jpg 0.540821**

**037_cam6_3.jpg 077_frontal.jpg 0.340379**

**038_cam6_3.jpg 005_frontal.jpg 0.297901**

**039_cam6_3.jpg 042_frontal.jpg 0.466562**

**040_cam6_3.jpg 036_frontal.jpg 0.358224**

**041_cam6_3.jpg 027_frontal.jpg 0.421430**

**042_cam6_3.jpg 042_frontal.jpg 0.360994**

**043_cam6_3.jpg 010_frontal.jpg 0.389348**

**044_cam6_3.jpg 042_frontal.jpg 0.418567**

**045_cam6_3.jpg 027_frontal.jpg 0.382145**

**046_cam6_3.jpg 046_frontal.jpg 0.429075**

**047_cam6_3.jpg 042_frontal.jpg 0.492655**

**048_cam6_3.jpg 065_frontal.jpg 0.354419**

**049_cam6_3.jpg 011_frontal.jpg 0.337413**

**050_cam6_3.jpg 011_frontal.jpg 0.418572**

**051_cam6_3.jpg 061_frontal.jpg 0.517106**

**052_cam6_3.jpg 118_frontal.jpg 0.440107**

**053_cam6_3.jpg 046_frontal.jpg 0.393997**

**054_cam6_3.jpg 089_frontal.jpg 0.428152**

**055_cam6_3.jpg 092_frontal.jpg 0.452032**

**056_cam6_3.jpg 005_frontal.jpg 0.486227**

**057_cam6_3.jpg 042_frontal.jpg 0.457236**

**058_cam6_3.jpg 042_frontal.jpg 0.370271**

**059_cam6_3.jpg 042_frontal.jpg 0.363737**

**060_cam6_3.jpg 042_frontal.jpg 0.579256**

**061_cam6_3.jpg 025_frontal.jpg 0.379202**

**062_cam6_3.jpg 065_frontal.jpg 0.423930**

**063_cam6_3.jpg 063_frontal.jpg 0.452503**

**064_cam6_3.jpg 100_frontal.jpg 0.408684**

**065_cam6_3.jpg 042_frontal.jpg 0.502148**

**066_cam6_3.jpg 042_frontal.jpg 0.660566**

**067_cam6_3.jpg 036_frontal.jpg 0.428458**

**068_cam6_3.jpg 011_frontal.jpg 0.406108**

**069_cam6_3.jpg 120_frontal.jpg 0.334751**

**070_cam6_3.jpg 011_frontal.jpg 0.344692**

**071_cam6_3.jpg 027_frontal.jpg 0.501739**

**072_cam6_3.jpg 042_frontal.jpg 0.517212**

**073_cam6_3.jpg 027_frontal.jpg 0.397063**

**074_cam6_3.jpg 006_frontal.jpg 0.411298**

**075_cam6_3.jpg 089_frontal.jpg 0.465304**

**076_cam6_3.jpg 011_frontal.jpg 0.542071**

**077_cam6_3.jpg 006_frontal.jpg 0.460077**

**078_cam6_3.jpg 042_frontal.jpg 0.456300**

**079_cam6_3.jpg 108_frontal.jpg 0.470285**

**080_cam6_3.jpg 011_frontal.jpg 0.350052**

**081_cam6_3.jpg 077_frontal.jpg 0.375754**

**082_cam6_3.jpg 077_frontal.jpg 0.452196**

**083_cam6_3.jpg 046_frontal.jpg 0.352392**

**084_cam6_3.jpg 124_frontal.jpg 0.444378**

**085_cam6_3.jpg 085_frontal.jpg 0.525865**

**086_cam6_3.jpg 027_frontal.jpg 0.478704**

**087_cam6_3.jpg 042_frontal.jpg 0.559806**

**088_cam6_3.jpg 025_frontal.jpg 0.540791**

**089_cam6_3.jpg 042_frontal.jpg 0.572806**

**090_cam6_3.jpg 027_frontal.jpg 0.412552**

**091_cam6_3.jpg 027_frontal.jpg 0.389363**

**092_cam6_3.jpg 046_frontal.jpg 0.535874**

**093_cam6_3.jpg 064_frontal.jpg 0.431164**

**094_cam6_3.jpg 027_frontal.jpg 0.428664**

**095_cam6_3.jpg 027_frontal.jpg 0.473630**

**096_cam6_3.jpg 027_frontal.jpg 0.527807**

**097_cam6_3.jpg 042_frontal.jpg 0.395251**

**098_cam6_3.jpg 042_frontal.jpg 0.512044**

**099_cam6_3.jpg 042_frontal.jpg 0.322376**

**100_cam6_3.jpg 100_frontal.jpg 0.674428**

**101_cam6_3.jpg 011_frontal.jpg 0.469255**

**102_cam6_3.jpg 006_frontal.jpg 0.452092**

**103_cam6_3.jpg 042_frontal.jpg 0.434065**

**104_cam6_3.jpg 036_frontal.jpg 0.346566**

**105_cam6_3.jpg 027_frontal.jpg 0.474918**

**106_cam6_3.jpg 042_frontal.jpg 0.416152**

**107_cam6_3.jpg 055_frontal.jpg 0.367607**

**108_cam6_3.jpg 042_frontal.jpg 0.389609**

**109_cam6_3.jpg 036_frontal.jpg 0.423915**

**110_cam6_3.jpg 036_frontal.jpg 0.344324**

**111_cam6_3.jpg 005_frontal.jpg 0.340084**

**112_cam6_3.jpg 077_frontal.jpg 0.582568**

**113_cam6_3.jpg 113_frontal.jpg 0.418403**

**114_cam6_3.jpg 042_frontal.jpg 0.504741**

**115_cam6_3.jpg 050_frontal.jpg 0.379716**

**116_cam6_3.jpg 111_frontal.jpg 0.291675**

**117_cam6_3.jpg 100_frontal.jpg 0.374793**

**118_cam6_3.jpg 042_frontal.jpg 0.444207**

**119_cam6_3.jpg 027_frontal.jpg 0.283098**

**120_cam6_3.jpg 046_frontal.jpg 0.457057**

**121_cam6_3.jpg 005_frontal.jpg 0.434288**

**122_cam6_3.jpg 124_frontal.jpg 0.282238**

**123_cam6_3.jpg 121_frontal.jpg 0.365235**

**124_cam6_3.jpg 124_frontal.jpg 0.464396**

**125_cam6_3.jpg 011_frontal.jpg 0.419324**

**126_cam6_3.jpg 006_frontal.jpg 0.503396**

**127_cam6_3.jpg 011_frontal.jpg 0.369819**

**128_cam6_3.jpg 011_frontal.jpg 0.502792**

**129_cam6_3.jpg 039_frontal.jpg 0.406740**

**130_cam6_3.jpg 036_frontal.jpg 0.395072**

**Final Score Scface SSR (V-IR) cam7-d1**

**Actual Image Recognized Image Score**

**001_cam7_1.jpg 032_frontal.jpg 0.766554**

**002_cam7_1.jpg 043_frontal.jpg 0.782491**

**003_cam7_1.jpg 043_frontal.jpg 0.643453**

**004_cam7_1.jpg 040_frontal.jpg 0.670759**

**005_cam7_1.jpg 087_frontal.jpg 0.671795**

**006_cam7_1.jpg 042_frontal.jpg 0.552202**

**007_cam7_1.jpg 043_frontal.jpg 0.431155**

**008_cam7_1.jpg 027_frontal.jpg 0.439919**

**009_cam7_1.jpg 100_frontal.jpg 0.455380**

**010_cam7_1.jpg 057_frontal.jpg 0.493898**

**011_cam7_1.jpg 088_frontal.jpg 0.626751**

**012_cam7_1.jpg 102_frontal.jpg 0.473116**

**013_cam7_1.jpg 114_frontal.jpg 0.608189**

**014_cam7_1.jpg 014_frontal.jpg 0.610975**

**015_cam7_1.jpg 076_frontal.jpg 0.418602**

**016_cam7_1.jpg 015_frontal.jpg 0.412846**

**017_cam7_1.jpg 093_frontal.jpg 0.513860**

**018_cam7_1.jpg 040_frontal.jpg 0.552307**

**019_cam7_1.jpg 112_frontal.jpg 0.623624**

**020_cam7_1.jpg 059_frontal.jpg 0.521630**

**021_cam7_1.jpg 087_frontal.jpg 0.561426**

**022_cam7_1.jpg 040_frontal.jpg 0.570012**

**023_cam7_1.jpg 072_frontal.jpg 0.797795**

**024_cam7_1.jpg 009_frontal.jpg 0.533218**

**025_cam7_1.jpg 021_frontal.jpg 0.549774**

**026_cam7_1.jpg 102_frontal.jpg 0.637141**

**027_cam7_1.jpg 027_frontal.jpg 0.718654**

**028_cam7_1.jpg 028_frontal.jpg 0.600994**

**029_cam7_1.jpg 076_frontal.jpg 0.571207**

**030_cam7_1.jpg 006_frontal.jpg 0.489729**

**031_cam7_1.jpg 040_frontal.jpg 0.636037**

**032_cam7_1.jpg 040_frontal.jpg 0.405589**

**033_cam7_1.jpg 065_frontal.jpg 0.590426**

**034_cam7_1.jpg 063_frontal.jpg 0.373337**

**035_cam7_1.jpg 100_frontal.jpg 0.501000**

**036_cam7_1.jpg 042_frontal.jpg 0.445893**

**037_cam7_1.jpg 087_frontal.jpg 0.662418**

**038_cam7_1.jpg 076_frontal.jpg 0.602144**

**039_cam7_1.jpg 068_frontal.jpg 0.434752**

**040_cam7_1.jpg 087_frontal.jpg 0.785192**

**041_cam7_1.jpg 117_frontal.jpg 0.670154**

**042_cam7_1.jpg 087_frontal.jpg 0.375669**

**043_cam7_1.jpg 043_frontal.jpg 0.597359**

**044_cam7_1.jpg 043_frontal.jpg 0.672138**

**045_cam7_1.jpg 040_frontal.jpg 0.465883**

**046_cam7_1.jpg 015_frontal.jpg 0.673636**

**047_cam7_1.jpg 043_frontal.jpg 0.503035**

**048_cam7_1.jpg 088_frontal.jpg 0.751254**

**049_cam7_1.jpg 042_frontal.jpg 0.812673**

**050_cam7_1.jpg 043_frontal.jpg 0.424964**

**051_cam7_1.jpg 063_frontal.jpg 0.533185**

**052_cam7_1.jpg 077_frontal.jpg 0.578869**

**053_cam7_1.jpg 100_frontal.jpg 0.484794**

**054_cam7_1.jpg 028_frontal.jpg 0.461887**

**055_cam7_1.jpg 046_frontal.jpg 0.547612**

**056_cam7_1.jpg 088_frontal.jpg 0.562074**

**057_cam7_1.jpg 001_frontal.jpg 0.579573**

**058_cam7_1.jpg 068_frontal.jpg 0.397731**

**059_cam7_1.jpg 028_frontal.jpg 0.369022**

**060_cam7_1.jpg 015_frontal.jpg 0.470685**

**061_cam7_1.jpg 077_frontal.jpg 0.431295**

**062_cam7_1.jpg 077_frontal.jpg 0.601471**

**063_cam7_1.jpg 107_frontal.jpg 0.570647**

**064_cam7_1.jpg 043_frontal.jpg 0.397623**

**065_cam7_1.jpg 043_frontal.jpg 0.696781**

**066_cam7_1.jpg 001_frontal.jpg 0.666107**

**067_cam7_1.jpg 088_frontal.jpg 0.825829**

**068_cam7_1.jpg 076_frontal.jpg 0.746267**

**069_cam7_1.jpg 006_frontal.jpg 0.501914**

**070_cam7_1.jpg 043_frontal.jpg 0.429208**

**071_cam7_1.jpg 006_frontal.jpg 0.504295**

**072_cam7_1.jpg 088_frontal.jpg 0.569228**

**073_cam7_1.jpg 027_frontal.jpg 0.468992**

**074_cam7_1.jpg 029_frontal.jpg 0.482187**

**075_cam7_1.jpg 015_frontal.jpg 0.561857**

**076_cam7_1.jpg 039_frontal.jpg 0.676437**

**077_cam7_1.jpg 076_frontal.jpg 0.667055**

**078_cam7_1.jpg 068_frontal.jpg 0.503767**

**079_cam7_1.jpg 088_frontal.jpg 0.597099**

**080_cam7_1.jpg 027_frontal.jpg 0.586702**

**081_cam7_1.jpg 068_frontal.jpg 0.579033**

**082_cam7_1.jpg 102_frontal.jpg 0.492004**

**083_cam7_1.jpg 027_frontal.jpg 0.330103**

**084_cam7_1.jpg 068_frontal.jpg 0.487441**

**085_cam7_1.jpg 002_frontal.jpg 0.546846**

**086_cam7_1.jpg 014_frontal.jpg 0.603216**

**087_cam7_1.jpg 002_frontal.jpg 0.602878**

**088_cam7_1.jpg 043_frontal.jpg 0.665518**

**089_cam7_1.jpg 102_frontal.jpg 0.651600**

**090_cam7_1.jpg 043_frontal.jpg 0.546309**

**091_cam7_1.jpg 076_frontal.jpg 0.620108**

**092_cam7_1.jpg 025_frontal.jpg 0.366309**

**093_cam7_1.jpg 062_frontal.jpg 0.549149**

**094_cam7_1.jpg 077_frontal.jpg 0.474296**

**095_cam7_1.jpg 059_frontal.jpg 0.530152**

**096_cam7_1.jpg 112_frontal.jpg 0.637847**

**097_cam7_1.jpg 042_frontal.jpg 0.568540**

**098_cam7_1.jpg 043_frontal.jpg 1.090670**

**099_cam7_1.jpg 068_frontal.jpg 0.466051**

**100_cam7_1.jpg 039_frontal.jpg 0.776714**

**101_cam7_1.jpg 075_frontal.jpg 0.417173**

**102_cam7_1.jpg 088_frontal.jpg 0.402303**

**103_cam7_1.jpg 009_frontal.jpg 0.847202**

**104_cam7_1.jpg 002_frontal.jpg 0.485220**

**105_cam7_1.jpg 088_frontal.jpg 0.668393**

**106_cam7_1.jpg 006_frontal.jpg 0.837910**

**107_cam7_1.jpg 056_frontal.jpg 0.495940**

**108_cam7_1.jpg 040_frontal.jpg 0.579296**

**109_cam7_1.jpg 032_frontal.jpg 0.383720**

**110_cam7_1.jpg 027_frontal.jpg 0.416908**

**111_cam7_1.jpg 093_frontal.jpg 0.393521**

**112_cam7_1.jpg 088_frontal.jpg 0.407927**

**113_cam7_1.jpg 087_frontal.jpg 0.653239**

**114_cam7_1.jpg 088_frontal.jpg 0.830685**

**115_cam7_1.jpg 063_frontal.jpg 0.715368**

**116_cam7_1.jpg 104_frontal.jpg 0.999829**

**117_cam7_1.jpg 001_frontal.jpg 0.846814**

**118_cam7_1.jpg 044_frontal.jpg 0.487143**

**119_cam7_1.jpg 076_frontal.jpg 0.426052**

**120_cam7_1.jpg 043_frontal.jpg 0.620386**

**121_cam7_1.jpg 088_frontal.jpg 0.584159**

**122_cam7_1.jpg 076_frontal.jpg 0.532143**

**123_cam7_1.jpg 076_frontal.jpg 0.971635**

**124_cam7_1.jpg 102_frontal.jpg 0.528998**

**125_cam7_1.jpg 056_frontal.jpg 0.528449**

**126_cam7_1.jpg 107_frontal.jpg 0.621998**

**127_cam7_1.jpg 102_frontal.jpg 0.557845**

**128_cam7_1.jpg 015_frontal.jpg 0.523644**

**129_cam7_1.jpg 027_frontal.jpg 0.514422**

**130_cam7_1.jpg 043_frontal.jpg 0.663310**

**Final Score Scface SSR (V-IR) cam7-d2**

**Actual Image Recognized Image Score**

**001_cam7_2.jpg 042_frontal.jpg 0.458989**

**002_cam7_2.jpg 027_frontal.jpg 0.466495**

**003_cam7_2.jpg 027_frontal.jpg 0.456553**

**004_cam7_2.jpg 011_frontal.jpg 0.361562**

**005_cam7_2.jpg 042_frontal.jpg 0.631932**

**006_cam7_2.jpg 040_frontal.jpg 0.387627**

**007_cam7_2.jpg 124_frontal.jpg 0.432562**

**008_cam7_2.jpg 103_frontal.jpg 0.506124**

**009_cam7_2.jpg 009_frontal.jpg 0.316733**

**010_cam7_2.jpg 010_frontal.jpg 0.393878**

**011_cam7_2.jpg 027_frontal.jpg 0.452133**

**012_cam7_2.jpg 042_frontal.jpg 0.595625**

**013_cam7_2.jpg 042_frontal.jpg 0.513429**

**014_cam7_2.jpg 014_frontal.jpg 0.492162**

**015_cam7_2.jpg 121_frontal.jpg 0.464722**

**016_cam7_2.jpg 011_frontal.jpg 0.499259**

**017_cam7_2.jpg 092_frontal.jpg 0.384187**

**018_cam7_2.jpg 054_frontal.jpg 0.409252**

**019_cam7_2.jpg 054_frontal.jpg 0.498700**

**020_cam7_2.jpg 093_frontal.jpg 0.410018**

**021_cam7_2.jpg 027_frontal.jpg 0.547046**

**022_cam7_2.jpg 027_frontal.jpg 0.381088**

**023_cam7_2.jpg 027_frontal.jpg 0.587438**

**024_cam7_2.jpg 019_frontal.jpg 0.424410**

**025_cam7_2.jpg 025_frontal.jpg 0.558943**

**026_cam7_2.jpg 027_frontal.jpg 0.723712**

**027_cam7_2.jpg 027_frontal.jpg 0.684708**

**028_cam7_2.jpg 011_frontal.jpg 0.418599**

**029_cam7_2.jpg 077_frontal.jpg 0.351945**

**030_cam7_2.jpg 054_frontal.jpg 0.399022**

**031_cam7_2.jpg 077_frontal.jpg 0.527861**

**032_cam7_2.jpg 042_frontal.jpg 0.419748**

**033_cam7_2.jpg 027_frontal.jpg 0.633495**

**034_cam7_2.jpg 027_frontal.jpg 0.471502**

**035_cam7_2.jpg 072_frontal.jpg 0.437651**

**036_cam7_2.jpg 027_frontal.jpg 0.397485**

**037_cam7_2.jpg 054_frontal.jpg 0.550248**

**038_cam7_2.jpg 011_frontal.jpg 0.410426**

**039_cam7_2.jpg 042_frontal.jpg 0.459216**

**040_cam7_2.jpg 010_frontal.jpg 0.530478**

**041_cam7_2.jpg 027_frontal.jpg 0.537905**

**042_cam7_2.jpg 027_frontal.jpg 0.410437**

**043_cam7_2.jpg 036_frontal.jpg 0.505623**

**044_cam7_2.jpg 011_frontal.jpg 0.525325**

**045_cam7_2.jpg 042_frontal.jpg 0.567990**

**046_cam7_2.jpg 006_frontal.jpg 0.527822**

**047_cam7_2.jpg 046_frontal.jpg 0.443662**

**048_cam7_2.jpg 042_frontal.jpg 0.513906**

**049_cam7_2.jpg 027_frontal.jpg 0.462849**

**050_cam7_2.jpg 011_frontal.jpg 0.456465**

**051_cam7_2.jpg 019_frontal.jpg 0.407637**

**052_cam7_2.jpg 042_frontal.jpg 0.525584**

**053_cam7_2.jpg 006_frontal.jpg 0.465105**

**054_cam7_2.jpg 027_frontal.jpg 0.549084**

**055_cam7_2.jpg 042_frontal.jpg 0.434535**

**056_cam7_2.jpg 005_frontal.jpg 0.505626**

**057_cam7_2.jpg 075_frontal.jpg 0.354457**

**058_cam7_2.jpg 027_frontal.jpg 0.682060**

**059_cam7_2.jpg 065_frontal.jpg 0.579165**

**060_cam7_2.jpg 042_frontal.jpg 0.532471**

**061_cam7_2.jpg 025_frontal.jpg 0.407133**

**062_cam7_2.jpg 092_frontal.jpg 0.371975**

**063_cam7_2.jpg 047_frontal.jpg 0.505089**

**064_cam7_2.jpg 077_frontal.jpg 0.519412**

**065_cam7_2.jpg 054_frontal.jpg 0.476427**

**066_cam7_2.jpg 011_frontal.jpg 0.443096**

**067_cam7_2.jpg 027_frontal.jpg 0.526952**

**068_cam7_2.jpg 027_frontal.jpg 0.491724**

**069_cam7_2.jpg 011_frontal.jpg 0.404368**

**070_cam7_2.jpg 042_frontal.jpg 0.548591**

**071_cam7_2.jpg 032_frontal.jpg 0.565314**

**072_cam7_2.jpg 042_frontal.jpg 0.350246**

**073_cam7_2.jpg 042_frontal.jpg 0.458537**

**074_cam7_2.jpg 042_frontal.jpg 0.430956**

**075_cam7_2.jpg 027_frontal.jpg 0.445138**

**076_cam7_2.jpg 072_frontal.jpg 0.335668**

**077_cam7_2.jpg 065_frontal.jpg 0.478348**

**078_cam7_2.jpg 027_frontal.jpg 0.501665**

**079_cam7_2.jpg 011_frontal.jpg 0.482949**

**080_cam7_2.jpg 065_frontal.jpg 0.368253**

**081_cam7_2.jpg 011_frontal.jpg 0.558078**

**082_cam7_2.jpg 004_frontal.jpg 0.448474**

**083_cam7_2.jpg 108_frontal.jpg 0.446215**

**084_cam7_2.jpg 011_frontal.jpg 0.356223**

**085_cam7_2.jpg 059_frontal.jpg 0.492180**

**086_cam7_2.jpg 072_frontal.jpg 0.431608**

**087_cam7_2.jpg 010_frontal.jpg 0.455523**

**088_cam7_2.jpg 011_frontal.jpg 0.468578**

**089_cam7_2.jpg 072_frontal.jpg 0.421267**

**090_cam7_2.jpg 036_frontal.jpg 0.466341**

**091_cam7_2.jpg 027_frontal.jpg 0.622619**

**092_cam7_2.jpg 117_frontal.jpg 0.415114**

**093_cam7_2.jpg 106_frontal.jpg 0.467723**

**094_cam7_2.jpg 054_frontal.jpg 0.506903**

**095_cam7_2.jpg 027_frontal.jpg 0.605463**

**096_cam7_2.jpg 027_frontal.jpg 0.490480**

**097_cam7_2.jpg 027_frontal.jpg 0.442984**

**098_cam7_2.jpg 027_frontal.jpg 0.493065**

**099_cam7_2.jpg 010_frontal.jpg 0.379969**

**100_cam7_2.jpg 065_frontal.jpg 0.482551**

**101_cam7_2.jpg 108_frontal.jpg 0.446407**

**102_cam7_2.jpg 042_frontal.jpg 0.665816**

**103_cam7_2.jpg 013_frontal.jpg 0.370014**

**104_cam7_2.jpg 104_frontal.jpg 0.447167**

**105_cam7_2.jpg 042_frontal.jpg 0.612766**

**106_cam7_2.jpg 006_frontal.jpg 0.405537**

**107_cam7_2.jpg 027_frontal.jpg 0.325414**

**108_cam7_2.jpg 027_frontal.jpg 0.458837**

**109_cam7_2.jpg 042_frontal.jpg 0.471033**

**110_cam7_2.jpg 089_frontal.jpg 0.336158**

**111_cam7_2.jpg 046_frontal.jpg 0.325211**

**112_cam7_2.jpg 027_frontal.jpg 0.536619**

**113_cam7_2.jpg 011_frontal.jpg 0.479210**

**114_cam7_2.jpg 011_frontal.jpg 0.529624**

**115_cam7_2.jpg 127_frontal.jpg 0.346634**

**116_cam7_2.jpg 011_frontal.jpg 0.567594**

**117_cam7_2.jpg 011_frontal.jpg 0.417848**

**118_cam7_2.jpg 092_frontal.jpg 0.329199**

**119_cam7_2.jpg 015_frontal.jpg 0.525704**

**120_cam7_2.jpg 065_frontal.jpg 0.373118**

**121_cam7_2.jpg 010_frontal.jpg 0.540667**

**122_cam7_2.jpg 011_frontal.jpg 0.498580**

**123_cam7_2.jpg 006_frontal.jpg 0.527246**

**124_cam7_2.jpg 027_frontal.jpg 0.638678**

**125_cam7_2.jpg 121_frontal.jpg 0.521448**

**126_cam7_2.jpg 042_frontal.jpg 0.517376**

**127_cam7_2.jpg 011_frontal.jpg 0.575378**

**128_cam7_2.jpg 027_frontal.jpg 0.511606**

**129_cam7_2.jpg 036_frontal.jpg 0.384016**

**130_cam7_2.jpg 042_frontal.jpg 0.460169**

**Final Score Scface SSR (V-IR) cam7-d3**

**Actual Image Recognized Image Score**

**001_cam7_3.jpg 077_frontal.jpg 0.597287**

**002_cam7_3.jpg 027_frontal.jpg 0.730018**

**003_cam7_3.jpg 121_frontal.jpg 0.492415**

**004_cam7_3.jpg 054_frontal.jpg 0.453621**

**005_cam7_3.jpg 078_frontal.jpg 0.593174**

**006_cam7_3.jpg 054_frontal.jpg 0.602282**

**007_cam7_3.jpg 054_frontal.jpg 0.603434**

**008_cam7_3.jpg 008_frontal.jpg 0.561062**

**009_cam7_3.jpg 108_frontal.jpg 0.379364**

**010_cam7_3.jpg 054_frontal.jpg 0.493196**

**011_cam7_3.jpg 011_frontal.jpg 0.623641**

**012_cam7_3.jpg 078_frontal.jpg 0.417902**

**013_cam7_3.jpg 040_frontal.jpg 0.832195**

**014_cam7_3.jpg 014_frontal.jpg 0.584007**

**015_cam7_3.jpg 011_frontal.jpg 0.518901**

**016_cam7_3.jpg 056_frontal.jpg 0.397818**

**017_cam7_3.jpg 042_frontal.jpg 0.648182**

**018_cam7_3.jpg 015_frontal.jpg 0.485727**

**019_cam7_3.jpg 027_frontal.jpg 0.576232**

**020_cam7_3.jpg 015_frontal.jpg 0.620359**

**021_cam7_3.jpg 050_frontal.jpg 0.491201**

**022_cam7_3.jpg 077_frontal.jpg 0.429638**

**023_cam7_3.jpg 040_frontal.jpg 0.779402**

**024_cam7_3.jpg 120_frontal.jpg 0.508811**

**025_cam7_3.jpg 011_frontal.jpg 0.513105**

**026_cam7_3.jpg 121_frontal.jpg 0.644761**

**027_cam7_3.jpg 103_frontal.jpg 0.436979**

**028_cam7_3.jpg 054_frontal.jpg 0.623037**

**029_cam7_3.jpg 029_frontal.jpg 0.521648**

**030_cam7_3.jpg 028_frontal.jpg 0.574109**

**031_cam7_3.jpg 040_frontal.jpg 0.634128**

**032_cam7_3.jpg 059_frontal.jpg 0.513356**

**033_cam7_3.jpg 015_frontal.jpg 0.687561**

**034_cam7_3.jpg 075_frontal.jpg 0.477352**

**035_cam7_3.jpg 075_frontal.jpg 0.471269**

**036_cam7_3.jpg 027_frontal.jpg 0.597404**

**037_cam7_3.jpg 050_frontal.jpg 0.474233**

**038_cam7_3.jpg 011_frontal.jpg 0.495767**

**039_cam7_3.jpg 078_frontal.jpg 0.480503**

**040_cam7_3.jpg 040_frontal.jpg 0.766797**

**041_cam7_3.jpg 077_frontal.jpg 0.403179**

**042_cam7_3.jpg 074_frontal.jpg 0.491712**

**043_cam7_3.jpg 010_frontal.jpg 0.393004**

**044_cam7_3.jpg 040_frontal.jpg 0.612960**

**045_cam7_3.jpg 054_frontal.jpg 0.509048**

**046_cam7_3.jpg 006_frontal.jpg 0.509494**

**047_cam7_3.jpg 077_frontal.jpg 0.622628**

**048_cam7_3.jpg 040_frontal.jpg 0.581402**

**049_cam7_3.jpg 011_frontal.jpg 0.520857**

**050_cam7_3.jpg 091_frontal.jpg 0.589286**

**051_cam7_3.jpg 080_frontal.jpg 0.465762**

**052_cam7_3.jpg 097_frontal.jpg 0.446211**

**053_cam7_3.jpg 078_frontal.jpg 0.568168**

**054_cam7_3.jpg 005_frontal.jpg 0.569925**

**055_cam7_3.jpg 077_frontal.jpg 0.470150**

**056_cam7_3.jpg 097_frontal.jpg 0.579297**

**057_cam7_3.jpg 092_frontal.jpg 0.529273**

**058_cam7_3.jpg 064_frontal.jpg 0.472514**

**059_cam7_3.jpg 120_frontal.jpg 0.653349**

**060_cam7_3.jpg 019_frontal.jpg 0.391345**

**061_cam7_3.jpg 011_frontal.jpg 0.714182**

**062_cam7_3.jpg 005_frontal.jpg 0.436334**

**063_cam7_3.jpg 092_frontal.jpg 0.527960**

**064_cam7_3.jpg 040_frontal.jpg 0.561079**

**065_cam7_3.jpg 054_frontal.jpg 0.670310**

**066_cam7_3.jpg 077_frontal.jpg 0.667564**

**067_cam7_3.jpg 108_frontal.jpg 0.403144**

**068_cam7_3.jpg 029_frontal.jpg 0.590776**

**069_cam7_3.jpg 077_frontal.jpg 0.537652**

**070_cam7_3.jpg 108_frontal.jpg 0.408688**

**071_cam7_3.jpg 040_frontal.jpg 0.524538**

**072_cam7_3.jpg 098_frontal.jpg 0.625808**

**073_cam7_3.jpg 011_frontal.jpg 0.492308**

**074_cam7_3.jpg 015_frontal.jpg 0.510508**

**075_cam7_3.jpg 005_frontal.jpg 0.449033**

**076_cam7_3.jpg 027_frontal.jpg 0.505086**

**077_cam7_3.jpg 108_frontal.jpg 0.519475**

**078_cam7_3.jpg 050_frontal.jpg 0.454615**

**079_cam7_3.jpg 019_frontal.jpg 0.484061**

**080_cam7_3.jpg 108_frontal.jpg 0.508876**

**081_cam7_3.jpg 011_frontal.jpg 0.557106**

**082_cam7_3.jpg 027_frontal.jpg 0.672125**

**083_cam7_3.jpg 040_frontal.jpg 0.531957**

**084_cam7_3.jpg 124_frontal.jpg 0.607498**

**085_cam7_3.jpg 108_frontal.jpg 0.470127**

**086_cam7_3.jpg 027_frontal.jpg 0.464009**

**087_cam7_3.jpg 072_frontal.jpg 0.601469**

**088_cam7_3.jpg 047_frontal.jpg 0.652695**

**089_cam7_3.jpg 097_frontal.jpg 0.489485**

**090_cam7_3.jpg 010_frontal.jpg 0.467227**

**091_cam7_3.jpg 108_frontal.jpg 0.549522**

**092_cam7_3.jpg 077_frontal.jpg 0.584374**

**093_cam7_3.jpg 006_frontal.jpg 0.551940**

**094_cam7_3.jpg 054_frontal.jpg 0.472529**

**095_cam7_3.jpg 078_frontal.jpg 0.519042**

**096_cam7_3.jpg 027_frontal.jpg 0.519645**

**097_cam7_3.jpg 077_frontal.jpg 0.501081**

**098_cam7_3.jpg 108_frontal.jpg 0.494294**

**099_cam7_3.jpg 027_frontal.jpg 0.896124**

**100_cam7_3.jpg 040_frontal.jpg 0.517258**

**101_cam7_3.jpg 078_frontal.jpg 0.484219**

**102_cam7_3.jpg 077_frontal.jpg 0.804904**

**103_cam7_3.jpg 015_frontal.jpg 0.546522**

**104_cam7_3.jpg 097_frontal.jpg 0.510217**

**105_cam7_3.jpg 027_frontal.jpg 0.406511**

**106_cam7_3.jpg 097_frontal.jpg 0.412226**

**107_cam7_3.jpg 077_frontal.jpg 0.425647**

**108_cam7_3.jpg 004_frontal.jpg 0.518625**

**109_cam7_3.jpg 027_frontal.jpg 0.408075**

**110_cam7_3.jpg 012_frontal.jpg 0.462104**

**111_cam7_3.jpg 027_frontal.jpg 0.533414**

**112_cam7_3.jpg 077_frontal.jpg 0.776338**

**113_cam7_3.jpg 011_frontal.jpg 0.414524**

**114_cam7_3.jpg 010_frontal.jpg 0.387326**

**115_cam7_3.jpg 077_frontal.jpg 0.511051**

**116_cam7_3.jpg 108_frontal.jpg 0.422327**

**117_cam7_3.jpg 097_frontal.jpg 0.721727**

**118_cam7_3.jpg 026_frontal.jpg 0.504358**

**119_cam7_3.jpg 040_frontal.jpg 0.394936**

**120_cam7_3.jpg 040_frontal.jpg 0.484063**

**121_cam7_3.jpg 077_frontal.jpg 0.649004**

**122_cam7_3.jpg 011_frontal.jpg 0.394009**

**123_cam7_3.jpg 027_frontal.jpg 0.585950**

**124_cam7_3.jpg 027_frontal.jpg 0.727761**

**125_cam7_3.jpg 075_frontal.jpg 0.593242**

**126_cam7_3.jpg 036_frontal.jpg 0.414441**

**127_cam7_3.jpg 073_frontal.jpg 0.363722**

**128_cam7_3.jpg 011_frontal.jpg 0.666105**

**129_cam7_3.jpg 059_frontal.jpg 0.445496**

**130_cam7_3.jpg 124_frontal.jpg 0.543487**

**Final Score NIR-VIS SSR**

**Actual Image Recognized Image Score**

**001_cam1_1.bmp 001_frontal.bmp 0.096201**

**002_cam1_1.bmp 053_frontal.bmp 0.119282**

**003_cam1_1.bmp 562_frontal.bmp 0.062988**

**004_cam1_1.bmp 004_frontal.bmp 0.062002**

**005_cam1_1.bmp 569_frontal.bmp 0.051498**

**006_cam1_1.bmp 132_frontal.bmp 0.100012**

**007_cam1_1.bmp 007_frontal.bmp 0.059676**

**008_cam1_1.bmp 732_frontal.bmp 0.071826**

**009_cam1_1.bmp 009_frontal.bmp 0.156656**

**010_cam1_1.bmp 423_frontal.bmp 0.062003**

**011_cam1_1.bmp 317_frontal.bmp 0.059366**

**012_cam1_1.bmp 342_frontal.bmp 0.086646**

**013_cam1_1.bmp 035_frontal.bmp 0.062827**

**014_cam1_1.bmp 691_frontal.bmp 0.107361**

**015_cam1_1.bmp 015_frontal.bmp 0.115531**

**016_cam1_1.bmp 336_frontal.bmp 0.068201**

**017_cam1_1.bmp 174_frontal.bmp 0.093581**

**018_cam1_1.bmp 313_frontal.bmp 0.126308**

**019_cam1_1.bmp 697_frontal.bmp 0.117148**

**020_cam1_1.bmp 188_frontal.bmp 0.100467**

**021_cam1_1.bmp 423_frontal.bmp 0.068613**

**022_cam1_1.bmp 186_frontal.bmp 0.080810**

**023_cam1_1.bmp 134_frontal.bmp 0.060298**

**024_cam1_1.bmp 173_frontal.bmp 0.084255**

**025_cam1_1.bmp 313_frontal.bmp 0.058169**

**026_cam1_1.bmp 053_frontal.bmp 0.070942**

**027_cam1_1.bmp 155_frontal.bmp 0.082147**

**028_cam1_1.bmp 104_frontal.bmp 0.074563**

**029_cam1_1.bmp 029_frontal.bmp 0.087307**

**030_cam1_1.bmp 697_frontal.bmp 0.088215**

**031_cam1_1.bmp 524_frontal.bmp 0.074427**

**032_cam1_1.bmp 555_frontal.bmp 0.073381**

**033_cam1_1.bmp 174_frontal.bmp 0.063381**

**034_cam1_1.bmp 174_frontal.bmp 0.105953**

**035_cam1_1.bmp 035_frontal.bmp 0.054592**

**036_cam1_1.bmp 017_frontal.bmp 0.120219**

**037_cam1_1.bmp 569_frontal.bmp 0.064006**

**038_cam1_1.bmp 415_frontal.bmp 0.080148**

**039_cam1_1.bmp 313_frontal.bmp 0.066094**

**040_cam1_1.bmp 174_frontal.bmp 0.072788**

**041_cam1_1.bmp 009_frontal.bmp 0.074734**

**042_cam1_1.bmp 042_frontal.bmp 0.125391**

**043_cam1_1.bmp 521_frontal.bmp 0.070557**

**044_cam1_1.bmp 190_frontal.bmp 0.095169**

**045_cam1_1.bmp 336_frontal.bmp 0.098782**

**046_cam1_1.bmp 155_frontal.bmp 0.101653**

**047_cam1_1.bmp 174_frontal.bmp 0.081486**

**048_cam1_1.bmp 018_frontal.bmp 0.061516**

**049_cam1_1.bmp 147_frontal.bmp 0.065549**

**050_cam1_1.bmp 050_frontal.bmp 0.077042**

**051_cam1_1.bmp 051_frontal.bmp 0.076346**

**052_cam1_1.bmp 155_frontal.bmp 0.097946**

**053_cam1_1.bmp 339_frontal.bmp 0.110521**

**054_cam1_1.bmp 691_frontal.bmp 0.071393**

**055_cam1_1.bmp 174_frontal.bmp 0.091058**

**056_cam1_1.bmp 423_frontal.bmp 0.117139**

**057_cam1_1.bmp 188_frontal.bmp 0.093007**

**058_cam1_1.bmp 271_frontal.bmp 0.085701**

**059_cam1_1.bmp 313_frontal.bmp 0.051481**

**060_cam1_1.bmp 188_frontal.bmp 0.096406**

**061_cam1_1.bmp 078_frontal.bmp 0.090304**

**062_cam1_1.bmp 601_frontal.bmp 0.096242**

**063_cam1_1.bmp 691_frontal.bmp 0.088015**

**064_cam1_1.bmp 274_frontal.bmp 0.106598**

**065_cam1_1.bmp 732_frontal.bmp 0.090180**

**066_cam1_1.bmp 066_frontal.bmp 0.129773**

**067_cam1_1.bmp 339_frontal.bmp 0.082530**

**068_cam1_1.bmp 286_frontal.bmp 0.052031**

**069_cam1_1.bmp 011_frontal.bmp 0.067610**

**070_cam1_1.bmp 562_frontal.bmp 0.086764**

**071_cam1_1.bmp 343_frontal.bmp 0.059057**

**072_cam1_1.bmp 523_frontal.bmp 0.073598**

**073_cam1_1.bmp 324_frontal.bmp 0.064535**

**074_cam1_1.bmp 330_frontal.bmp 0.087895**

**075_cam1_1.bmp 075_frontal.bmp 0.081250**

**076_cam1_1.bmp 475_frontal.bmp 0.093415**

**077_cam1_1.bmp 171_frontal.bmp 0.066940**

**078_cam1_1.bmp 708_frontal.bmp 0.070912**

**079_cam1_1.bmp 324_frontal.bmp 0.095595**

**080_cam1_1.bmp 691_frontal.bmp 0.079024**

**081_cam1_1.bmp 081_frontal.bmp 0.083675**

**082_cam1_1.bmp 082_frontal.bmp 0.101243**

**083_cam1_1.bmp 326_frontal.bmp 0.072807**

**084_cam1_1.bmp 084_frontal.bmp 0.073167**

**085_cam1_1.bmp 737_frontal.bmp 0.057296**

**086_cam1_1.bmp 337_frontal.bmp 0.088227**

**087_cam1_1.bmp 147_frontal.bmp 0.095299**

**088_cam1_1.bmp 155_frontal.bmp 0.160507**

**089_cam1_1.bmp 557_frontal.bmp 0.080310**

**090_cam1_1.bmp 090_frontal.bmp 0.129397**

**091_cam1_1.bmp 555_frontal.bmp 0.068486**

**092_cam1_1.bmp 231_frontal.bmp 0.090132**

**093_cam1_1.bmp 328_frontal.bmp 0.053828**

**094_cam1_1.bmp 584_frontal.bmp 0.071071**

**095_cam1_1.bmp 235_frontal.bmp 0.058166**

**096_cam1_1.bmp 555_frontal.bmp 0.059143**

**097_cam1_1.bmp 018_frontal.bmp 0.067479**

**098_cam1_1.bmp 326_frontal.bmp 0.088982**

**099_cam1_1.bmp 174_frontal.bmp 0.151242**

**100_cam1_1.bmp 732_frontal.bmp 0.072049**

**101_cam1_1.bmp 691_frontal.bmp 0.064472**

**102_cam1_1.bmp 538_frontal.bmp 0.095711**

**103_cam1_1.bmp 155_frontal.bmp 0.124890**

**104_cam1_1.bmp 524_frontal.bmp 0.079252**

**105_cam1_1.bmp 155_frontal.bmp 0.084326**

**106_cam1_1.bmp 544_frontal.bmp 0.085798**

**107_cam1_1.bmp 523_frontal.bmp 0.080394**

**108_cam1_1.bmp 562_frontal.bmp 0.095111**

**109_cam1_1.bmp 348_frontal.bmp 0.122139**

**110_cam1_1.bmp 320_frontal.bmp 0.079872**

**111_cam1_1.bmp 111_frontal.bmp 0.095738**

**112_cam1_1.bmp 174_frontal.bmp 0.097520**

**113_cam1_1.bmp 326_frontal.bmp 0.078033**

**114_cam1_1.bmp 313_frontal.bmp 0.085945**

**115_cam1_1.bmp 017_frontal.bmp 0.138999**

**116_cam1_1.bmp 155_frontal.bmp 0.101485**

**117_cam1_1.bmp 732_frontal.bmp 0.068041**

**118_cam1_1.bmp 174_frontal.bmp 0.141906**

**119_cam1_1.bmp 336_frontal.bmp 0.082753**

**120_cam1_1.bmp 691_frontal.bmp 0.093975**

**121_cam1_1.bmp 271_frontal.bmp 0.078858**

**122_cam1_1.bmp 155_frontal.bmp 0.103654**

**123_cam1_1.bmp 209_frontal.bmp 0.156462**

**124_cam1_1.bmp 124_frontal.bmp 0.130562**

**125_cam1_1.bmp 691_frontal.bmp 0.092362**

**126_cam1_1.bmp 691_frontal.bmp 0.120522**

**127_cam1_1.bmp 691_frontal.bmp 0.080351**

**128_cam1_1.bmp 271_frontal.bmp 0.060129**

**129_cam1_1.bmp 313_frontal.bmp 0.118422**

**130_cam1_1.bmp 339_frontal.bmp 0.048670**

**131_cam1_1.bmp 265_frontal.bmp 0.081257**

**132_cam1_1.bmp 317_frontal.bmp 0.069121**

**133_cam1_1.bmp 271_frontal.bmp 0.122864**

**134_cam1_1.bmp 134_frontal.bmp 0.090304**

**135_cam1_1.bmp 174_frontal.bmp 0.113162**

**136_cam1_1.bmp 585_frontal.bmp 0.075832**

**137_cam1_1.bmp 562_frontal.bmp 0.057584**

**138_cam1_1.bmp 220_frontal.bmp 0.048204**

**139_cam1_1.bmp 691_frontal.bmp 0.119459**

**140_cam1_1.bmp 691_frontal.bmp 0.076952**

**141_cam1_1.bmp 691_frontal.bmp 0.073330**

**142_cam1_1.bmp 723_frontal.bmp 0.087155**

**143_cam1_1.bmp 548_frontal.bmp 0.069813**

**144_cam1_1.bmp 313_frontal.bmp 0.079009**

**145_cam1_1.bmp 002_frontal.bmp 0.068227**

**146_cam1_1.bmp 073_frontal.bmp 0.077544**

**147_cam1_1.bmp 317_frontal.bmp 0.080737**

**148_cam1_1.bmp 211_frontal.bmp 0.078516**

**149_cam1_1.bmp 523_frontal.bmp 0.091828**

**150_cam1_1.bmp 339_frontal.bmp 0.059529**

**151_cam1_1.bmp 166_frontal.bmp 0.086554**

**152_cam1_1.bmp 342_frontal.bmp 0.059239**

**153_cam1_1.bmp 153_frontal.bmp 0.061092**

**154_cam1_1.bmp 155_frontal.bmp 0.109504**

**155_cam1_1.bmp 155_frontal.bmp 0.195615**

**156_cam1_1.bmp 257_frontal.bmp 0.047046**

**157_cam1_1.bmp 271_frontal.bmp 0.113847**

**158_cam1_1.bmp 174_frontal.bmp 0.071929**

**159_cam1_1.bmp 159_frontal.bmp 0.124766**

**160_cam1_1.bmp 591_frontal.bmp 0.066850**

**161_cam1_1.bmp 161_frontal.bmp 0.105686**

**162_cam1_1.bmp 136_frontal.bmp 0.070848**

**163_cam1_1.bmp 163_frontal.bmp 0.069887**

**164_cam1_1.bmp 523_frontal.bmp 0.053641**

**165_cam1_1.bmp 165_frontal.bmp 0.102827**

**166_cam1_1.bmp 155_frontal.bmp 0.155190**

**167_cam1_1.bmp 056_frontal.bmp 0.136195**

**168_cam1_1.bmp 155_frontal.bmp 0.112232**

**169_cam1_1.bmp 169_frontal.bmp 0.129975**

**170_cam1_1.bmp 180_frontal.bmp 0.079894**

**171_cam1_1.bmp 171_frontal.bmp 0.098166**

**172_cam1_1.bmp 313_frontal.bmp 0.062125**

**173_cam1_1.bmp 180_frontal.bmp 0.105216**

**174_cam1_1.bmp 174_frontal.bmp 0.201532**

**175_cam1_1.bmp 271_frontal.bmp 0.096202**

**176_cam1_1.bmp 585_frontal.bmp 0.090033**

**177_cam1_1.bmp 555_frontal.bmp 0.061684**

**178_cam1_1.bmp 073_frontal.bmp 0.062713**

**179_cam1_1.bmp 056_frontal.bmp 0.098982**

**180_cam1_1.bmp 180_frontal.bmp 0.111156**

**181_cam1_1.bmp 184_frontal.bmp 0.097635**

**182_cam1_1.bmp 174_frontal.bmp 0.100829**

**183_cam1_1.bmp 066_frontal.bmp 0.158737**

**184_cam1_1.bmp 448_frontal.bmp 0.083879**

**185_cam1_1.bmp 185_frontal.bmp 0.094342**

**186_cam1_1.bmp 186_frontal.bmp 0.102653**

**187_cam1_1.bmp 174_frontal.bmp 0.080325**

**188_cam1_1.bmp 190_frontal.bmp 0.090160**

**189_cam1_1.bmp 559_frontal.bmp 0.059909**

**190_cam1_1.bmp 190_frontal.bmp 0.097333**

**191_cam1_1.bmp 056_frontal.bmp 0.097166**

**192_cam1_1.bmp 192_frontal.bmp 0.084460**

**193_cam1_1.bmp 235_frontal.bmp 0.071820**

**194_cam1_1.bmp 174_frontal.bmp 0.062372**

**195_cam1_1.bmp 035_frontal.bmp 0.057411**

**196_cam1_1.bmp 174_frontal.bmp 0.074869**

**197_cam1_1.bmp 271_frontal.bmp 0.068084**

**198_cam1_1.bmp 267_frontal.bmp 0.062242**

**199_cam1_1.bmp 559_frontal.bmp 0.040963**

**200_cam1_1.bmp 174_frontal.bmp 0.062675**

**201_cam1_1.bmp 339_frontal.bmp 0.062907**

**202_cam1_1.bmp 372_frontal.bmp 0.062143**

**203_cam1_1.bmp 334_frontal.bmp 0.179847**

**204_cam1_1.bmp 253_frontal.bmp 0.188761**

**205_cam1_1.bmp 313_frontal.bmp 0.050007**

**206_cam1_1.bmp 281_frontal.bmp 0.134792**

**207_cam1_1.bmp 568_frontal.bmp 0.210145**

**208_cam1_1.bmp 350_frontal.bmp 0.131743**

**209_cam1_1.bmp 209_frontal.bmp 0.117806**

**210_cam1_1.bmp 284_frontal.bmp 0.203827**

**211_cam1_1.bmp 253_frontal.bmp 0.151581**

**212_cam1_1.bmp 691_frontal.bmp 0.090382**

**213_cam1_1.bmp 213_frontal.bmp 0.179516**

**214_cam1_1.bmp 214_frontal.bmp 0.069878**

**215_cam1_1.bmp 348_frontal.bmp 0.055990**

**216_cam1_1.bmp 285_frontal.bmp 0.069245**

**217_cam1_1.bmp 389_frontal.bmp 0.055182**

**218_cam1_1.bmp 691_frontal.bmp 0.083330**

**219_cam1_1.bmp 598_frontal.bmp 0.128782**

**220_cam1_1.bmp 220_frontal.bmp 0.126572**

**221_cam1_1.bmp 221_frontal.bmp 0.078837**

**222_cam1_1.bmp 155_frontal.bmp 0.089655**

**223_cam1_1.bmp 223_frontal.bmp 0.080205**

**224_cam1_1.bmp 417_frontal.bmp 0.068865**

**225_cam1_1.bmp 417_frontal.bmp 0.070324**

**226_cam1_1.bmp 334_frontal.bmp 0.127924**

**227_cam1_1.bmp 670_frontal.bmp 0.180359**

**228_cam1_1.bmp 356_frontal.bmp 0.084447**

**229_cam1_1.bmp 229_frontal.bmp 0.072902**

**230_cam1_1.bmp 230_frontal.bmp 0.114876**

**231_cam1_1.bmp 231_frontal.bmp 0.118035**

**232_cam1_1.bmp 342_frontal.bmp 0.069488**

**233_cam1_1.bmp 158_frontal.bmp 0.045050**

**234_cam1_1.bmp 234_frontal.bmp 0.099863**

**235_cam1_1.bmp 314_frontal.bmp 0.103658**

**236_cam1_1.bmp 235_frontal.bmp 0.114534**

**237_cam1_1.bmp 237_frontal.bmp 0.125467**

**238_cam1_1.bmp 362_frontal.bmp 0.058565**

**239_cam1_1.bmp 224_frontal.bmp 0.067019**

**240_cam1_1.bmp 290_frontal.bmp 0.055453**

**241_cam1_1.bmp 244_frontal.bmp 0.202732**

**242_cam1_1.bmp 242_frontal.bmp 0.059667**

**243_cam1_1.bmp 253_frontal.bmp 0.121082**

**244_cam1_1.bmp 244_frontal.bmp 0.145441**

**245_cam1_1.bmp 245_frontal.bmp 0.126037**

**246_cam1_1.bmp 263_frontal.bmp 0.159709**

**247_cam1_1.bmp 204_frontal.bmp 0.136728**

**248_cam1_1.bmp 248_frontal.bmp 0.235778**

**249_cam1_1.bmp 249_frontal.bmp 0.175834**

**250_cam1_1.bmp 691_frontal.bmp 0.108383**

**251_cam1_1.bmp 379_frontal.bmp 0.099577**

**252_cam1_1.bmp 562_frontal.bmp 0.041364**

**253_cam1_1.bmp 253_frontal.bmp 0.320808**

**254_cam1_1.bmp 254_frontal.bmp 0.145608**

**255_cam1_1.bmp 711_frontal.bmp 0.067593**

**256_cam1_1.bmp 256_frontal.bmp 0.181157**

**257_cam1_1.bmp 257_frontal.bmp 0.089519**

**258_cam1_1.bmp 281_frontal.bmp 0.131775**

**259_cam1_1.bmp 725_frontal.bmp 0.052976**

**260_cam1_1.bmp 260_frontal.bmp 0.101235**

**261_cam1_1.bmp 273_frontal.bmp 0.183668**

**262_cam1_1.bmp 088_frontal.bmp 0.059705**

**263_cam1_1.bmp 227_frontal.bmp 0.220057**

**264_cam1_1.bmp 372_frontal.bmp 0.062202**

**265_cam1_1.bmp 265_frontal.bmp 0.173532**

**266_cam1_1.bmp 266_frontal.bmp 0.147678**

**267_cam1_1.bmp 155_frontal.bmp 0.065337**

**268_cam1_1.bmp 342_frontal.bmp 0.195047**

**269_cam1_1.bmp 314_frontal.bmp 0.099715**

**270_cam1_1.bmp 270_frontal.bmp 0.099405**

**271_cam1_1.bmp 691_frontal.bmp 0.090874**

**272_cam1_1.bmp 253_frontal.bmp 0.151741**

**273_cam1_1.bmp 320_frontal.bmp 0.102909**

**274_cam1_1.bmp 334_frontal.bmp 0.100488**

**275_cam1_1.bmp 275_frontal.bmp 0.134260**

**276_cam1_1.bmp 276_frontal.bmp 0.064698**

**277_cam1_1.bmp 277_frontal.bmp 0.074325**

**278_cam1_1.bmp 278_frontal.bmp 0.062164**

**279_cam1_1.bmp 279_frontal.bmp 0.068005**

**280_cam1_1.bmp 273_frontal.bmp 0.209723**

**281_cam1_1.bmp 281_frontal.bmp 0.179058**

**282_cam1_1.bmp 243_frontal.bmp 0.198974**

**283_cam1_1.bmp 283_frontal.bmp 0.135036**

**284_cam1_1.bmp 284_frontal.bmp 0.179336**

**285_cam1_1.bmp 349_frontal.bmp 0.060223**

**286_cam1_1.bmp 253_frontal.bmp 0.162419**

**287_cam1_1.bmp 484_frontal.bmp 0.167779**

**288_cam1_1.bmp 556_frontal.bmp 0.055204**

**289_cam1_1.bmp 691_frontal.bmp 0.062383**

**290_cam1_1.bmp 290_frontal.bmp 0.122610**

**291_cam1_1.bmp 281_frontal.bmp 0.172275**

**292_cam1_1.bmp 208_frontal.bmp 0.094243**

**293_cam1_1.bmp 709_frontal.bmp 0.059110**

**294_cam1_1.bmp 287_frontal.bmp 0.149432**

**295_cam1_1.bmp 334_frontal.bmp 0.182615**

**296_cam1_1.bmp 556_frontal.bmp 0.061889**

**297_cam1_1.bmp 662_frontal.bmp 0.115728**

**298_cam1_1.bmp 359_frontal.bmp 0.055979**

**299_cam1_1.bmp 287_frontal.bmp 0.147734**

**300_cam1_1.bmp 281_frontal.bmp 0.112376**

**301_cam1_1.bmp 350_frontal.bmp 0.160066**

**302_cam1_1.bmp 263_frontal.bmp 0.216964**

**303_cam1_1.bmp 544_frontal.bmp 0.220199**

**304_cam1_1.bmp 675_frontal.bmp 0.055140**

**305_cam1_1.bmp 521_frontal.bmp 0.056307**

**306_cam1_1.bmp 155_frontal.bmp 0.088746**

**307_cam1_1.bmp 307_frontal.bmp 0.104559**

**308_cam1_1.bmp 308_frontal.bmp 0.133895**

**309_cam1_1.bmp 314_frontal.bmp 0.053281**

**310_cam1_1.bmp 310_frontal.bmp 0.110219**

**311_cam1_1.bmp 155_frontal.bmp 0.091536**

**312_cam1_1.bmp 312_frontal.bmp 0.132330**

**313_cam1_1.bmp 313_frontal.bmp 0.130522**

**314_cam1_1.bmp 313_frontal.bmp 0.043429**

**315_cam1_1.bmp 315_frontal.bmp 0.065643**

**316_cam1_1.bmp 316_frontal.bmp 0.075283**

**317_cam1_1.bmp 245_frontal.bmp 0.231904**

**318_cam1_1.bmp 670_frontal.bmp 0.069964**

**319_cam1_1.bmp 319_frontal.bmp 0.095350**

**320_cam1_1.bmp 320_frontal.bmp 0.107863**

**321_cam1_1.bmp 711_frontal.bmp 0.077773**

**322_cam1_1.bmp 322_frontal.bmp 0.094569**

**323_cam1_1.bmp 290_frontal.bmp 0.087225**

**324_cam1_1.bmp 324_frontal.bmp 0.170710**

**325_cam1_1.bmp 563_frontal.bmp 0.037152**

**326_cam1_1.bmp 326_frontal.bmp 0.079979**

**327_cam1_1.bmp 327_frontal.bmp 0.094130**

**328_cam1_1.bmp 328_frontal.bmp 0.095443**

**329_cam1_1.bmp 329_frontal.bmp 0.142285**

**330_cam1_1.bmp 330_frontal.bmp 0.169327**

**331_cam1_1.bmp 359_frontal.bmp 0.078405**

**332_cam1_1.bmp 662_frontal.bmp 0.071407**

**333_cam1_1.bmp 333_frontal.bmp 0.073314**

**334_cam1_1.bmp 334_frontal.bmp 0.085961**

**335_cam1_1.bmp 335_frontal.bmp 0.197236**

**336_cam1_1.bmp 531_frontal.bmp 0.080405**

**337_cam1_1.bmp 337_frontal.bmp 0.128733**

**338_cam1_1.bmp 338_frontal.bmp 0.085232**

**339_cam1_1.bmp 339_frontal.bmp 0.157883**

**340_cam1_1.bmp 340_frontal.bmp 0.151466**

**341_cam1_1.bmp 341_frontal.bmp 0.159154**

**342_cam1_1.bmp 324_frontal.bmp 0.071355**

**343_cam1_1.bmp 343_frontal.bmp 0.113326**

**344_cam1_1.bmp 344_frontal.bmp 0.096263**

**345_cam1_1.bmp 345_frontal.bmp 0.116293**

**346_cam1_1.bmp 155_frontal.bmp 0.111997**

**347_cam1_1.bmp 348_frontal.bmp 0.114193**

**348_cam1_1.bmp 348_frontal.bmp 0.065888**

**349_cam1_1.bmp 349_frontal.bmp 0.119355**

**350_cam1_1.bmp 692_frontal.bmp 0.149198**

**351_cam1_1.bmp 598_frontal.bmp 0.124262**

**352_cam1_1.bmp 662_frontal.bmp 0.072915**

**353_cam1_1.bmp 353_frontal.bmp 0.106259**

**354_cam1_1.bmp 354_frontal.bmp 0.097492**

**355_cam1_1.bmp 250_frontal.bmp 0.096543**

**356_cam1_1.bmp 411_frontal.bmp 0.053769**

**357_cam1_1.bmp 415_frontal.bmp 0.117727**

**358_cam1_1.bmp 697_frontal.bmp 0.075618**

**359_cam1_1.bmp 359_frontal.bmp 0.190076**

**360_cam1_1.bmp 601_frontal.bmp 0.058767**

**361_cam1_1.bmp 361_frontal.bmp 0.104473**

**362_cam1_1.bmp 362_frontal.bmp 0.111639**

**363_cam1_1.bmp 363_frontal.bmp 0.086260**

**364_cam1_1.bmp 364_frontal.bmp 0.117765**

**365_cam1_1.bmp 365_frontal.bmp 0.105161**

**366_cam1_1.bmp 314_frontal.bmp 0.067690**

**367_cam1_1.bmp 314_frontal.bmp 0.092458**

**368_cam1_1.bmp 423_frontal.bmp 0.084616**

**369_cam1_1.bmp 584_frontal.bmp 0.046806**

**370_cam1_1.bmp 320_frontal.bmp 0.070514**

**371_cam1_1.bmp 371_frontal.bmp 0.074602**

**372_cam1_1.bmp 691_frontal.bmp 0.153295**

**373_cam1_1.bmp 610_frontal.bmp 0.145756**

**374_cam1_1.bmp 598_frontal.bmp 0.083390**

**375_cam1_1.bmp 485_frontal.bmp 0.168552**

**376_cam1_1.bmp 376_frontal.bmp 0.092789**

**377_cam1_1.bmp 377_frontal.bmp 0.085002**

**378_cam1_1.bmp 670_frontal.bmp 0.106318**

**379_cam1_1.bmp 697_frontal.bmp 0.093407**

**380_cam1_1.bmp 326_frontal.bmp 0.065487**

**381_cam1_1.bmp 011_frontal.bmp 0.068407**

**382_cam1_1.bmp 314_frontal.bmp 0.077051**

**383_cam1_1.bmp 262_frontal.bmp 0.078174**

**384_cam1_1.bmp 711_frontal.bmp 0.069081**

**385_cam1_1.bmp 455_frontal.bmp 0.063786**

**386_cam1_1.bmp 691_frontal.bmp 0.060851**

**387_cam1_1.bmp 522_frontal.bmp 0.070043**

**388_cam1_1.bmp 691_frontal.bmp 0.080358**

**389_cam1_1.bmp 389_frontal.bmp 0.115232**

**390_cam1_1.bmp 362_frontal.bmp 0.065046**

**391_cam1_1.bmp 697_frontal.bmp 0.061334**

**392_cam1_1.bmp 048_frontal.bmp 0.071551**

**393_cam1_1.bmp 697_frontal.bmp 0.046537**

**394_cam1_1.bmp 394_frontal.bmp 0.044152**

**395_cam1_1.bmp 691_frontal.bmp 0.079993**

**396_cam1_1.bmp 691_frontal.bmp 0.074780**

**397_cam1_1.bmp 556_frontal.bmp 0.032207**

**398_cam1_1.bmp 240_frontal.bmp 0.058484**

**399_cam1_1.bmp 313_frontal.bmp 0.051189**

**400_cam1_1.bmp 421_frontal.bmp 0.062523**

**401_cam1_1.bmp 497_frontal.bmp 0.057843**

**402_cam1_1.bmp 281_frontal.bmp 0.129406**

**403_cam1_1.bmp 231_frontal.bmp 0.047015**

**404_cam1_1.bmp 123_frontal.bmp 0.048877**

**405_cam1_1.bmp 245_frontal.bmp 0.195612**

**406_cam1_1.bmp 566_frontal.bmp 0.061479**

**407_cam1_1.bmp 314_frontal.bmp 0.087785**

**408_cam1_1.bmp 010_frontal.bmp 0.033628**

**409_cam1_1.bmp 053_frontal.bmp 0.053325**

**410_cam1_1.bmp 110_frontal.bmp 0.066353**

**411_cam1_1.bmp 224_frontal.bmp 0.062829**

**412_cam1_1.bmp 724_frontal.bmp 0.038360**

**413_cam1_1.bmp 224_frontal.bmp 0.051864**

**414_cam1_1.bmp 350_frontal.bmp 0.174537**

**415_cam1_1.bmp 415_frontal.bmp 0.071458**

**416_cam1_1.bmp 540_frontal.bmp 0.052488**

**417_cam1_1.bmp 312_frontal.bmp 0.058752**

**418_cam1_1.bmp 012_frontal.bmp 0.060511**

**419_cam1_1.bmp 624_frontal.bmp 0.036095**

**420_cam1_1.bmp 598_frontal.bmp 0.069581**

**421_cam1_1.bmp 421_frontal.bmp 0.061383**

**422_cam1_1.bmp 313_frontal.bmp 0.049271**

**423_cam1_1.bmp 314_frontal.bmp 0.105020**

**424_cam1_1.bmp 042_frontal.bmp 0.048169**

**425_cam1_1.bmp 735_frontal.bmp 0.047405**

**426_cam1_1.bmp 262_frontal.bmp 0.064768**

**427_cam1_1.bmp 350_frontal.bmp 0.114026**

**428_cam1_1.bmp 711_frontal.bmp 0.035338**

**429_cam1_1.bmp 240_frontal.bmp 0.082350**

**430_cam1_1.bmp 550_frontal.bmp 0.160717**

**431_cam1_1.bmp 313_frontal.bmp 0.055595**

**432_cam1_1.bmp 362_frontal.bmp 0.047685**

**433_cam1_1.bmp 433_frontal.bmp 0.359068**

**434_cam1_1.bmp 273_frontal.bmp 0.318847**

**435_cam1_1.bmp 610_frontal.bmp 0.146099**

**436_cam1_1.bmp 691_frontal.bmp 0.064688**

**437_cam1_1.bmp 691_frontal.bmp 0.057701**

**438_cam1_1.bmp 078_frontal.bmp 0.051201**

**439_cam1_1.bmp 469_frontal.bmp 0.044517**

**440_cam1_1.bmp 313_frontal.bmp 0.033373**

**441_cam1_1.bmp 349_frontal.bmp 0.044964**

**442_cam1_1.bmp 582_frontal.bmp 0.075400**

**443_cam1_1.bmp 337_frontal.bmp 0.039043**

**444_cam1_1.bmp 711_frontal.bmp 0.038606**

**445_cam1_1.bmp 339_frontal.bmp 0.060992**

**446_cam1_1.bmp 349_frontal.bmp 0.054305**

**447_cam1_1.bmp 455_frontal.bmp 0.062150**

**448_cam1_1.bmp 448_frontal.bmp 0.088837**

**449_cam1_1.bmp 235_frontal.bmp 0.066467**

**450_cam1_1.bmp 282_frontal.bmp 0.062528**

**451_cam1_1.bmp 559_frontal.bmp 0.061337**

**452_cam1_1.bmp 314_frontal.bmp 0.101498**

**453_cam1_1.bmp 670_frontal.bmp 0.101925**

**454_cam1_1.bmp 053_frontal.bmp 0.058440**

**455_cam1_1.bmp 455_frontal.bmp 0.071585**

**456_cam1_1.bmp 174_frontal.bmp 0.086761**

**457_cam1_1.bmp 329_frontal.bmp 0.038887**

**458_cam1_1.bmp 432_frontal.bmp 0.097848**

**459_cam1_1.bmp 151_frontal.bmp 0.084008**

**460_cam1_1.bmp 570_frontal.bmp 0.055253**

**461_cam1_1.bmp 313_frontal.bmp 0.056441**

**462_cam1_1.bmp 348_frontal.bmp 0.126525**

**463_cam1_1.bmp 624_frontal.bmp 0.048657**

**464_cam1_1.bmp 426_frontal.bmp 0.045571**

**465_cam1_1.bmp 350_frontal.bmp 0.063017**

**466_cam1_1.bmp 314_frontal.bmp 0.093641**

**467_cam1_1.bmp 253_frontal.bmp 0.189365**

**468_cam1_1.bmp 662_frontal.bmp 0.053435**

**469_cam1_1.bmp 310_frontal.bmp 0.088793**

**470_cam1_1.bmp 697_frontal.bmp 0.069666**

**471_cam1_1.bmp 662_frontal.bmp 0.125574**

**472_cam1_1.bmp 290_frontal.bmp 0.056771**

**473_cam1_1.bmp 155_frontal.bmp 0.099226**

**474_cam1_1.bmp 486_frontal.bmp 0.080631**

**475_cam1_1.bmp 558_frontal.bmp 0.048843**

**476_cam1_1.bmp 221_frontal.bmp 0.077145**

**477_cam1_1.bmp 284_frontal.bmp 0.255250**

**478_cam1_1.bmp 249_frontal.bmp 0.296749**

**479_cam1_1.bmp 558_frontal.bmp 0.047948**

**480_cam1_1.bmp 348_frontal.bmp 0.084461**

**481_cam1_1.bmp 323_frontal.bmp 0.044567**

**482_cam1_1.bmp 711_frontal.bmp 0.035715**

**483_cam1_1.bmp 624_frontal.bmp 0.077442**

**484_cam1_1.bmp 588_frontal.bmp 0.045979**

**485_cam1_1.bmp 235_frontal.bmp 0.048956**

**486_cam1_1.bmp 334_frontal.bmp 0.052112**

**487_cam1_1.bmp 711_frontal.bmp 0.072225**

**488_cam1_1.bmp 563_frontal.bmp 0.050249**

**489_cam1_1.bmp 489_frontal.bmp 0.052284**

**490_cam1_1.bmp 591_frontal.bmp 0.047404**

**491_cam1_1.bmp 334_frontal.bmp 0.064860**

**492_cam1_1.bmp 492_frontal.bmp 0.044000**

**493_cam1_1.bmp 670_frontal.bmp 0.086330**

**494_cam1_1.bmp 209_frontal.bmp 0.053199**

**495_cam1_1.bmp 174_frontal.bmp 0.042969**

**496_cam1_1.bmp 324_frontal.bmp 0.043018**

**497_cam1_1.bmp 349_frontal.bmp 0.047643**

**498_cam1_1.bmp 379_frontal.bmp 0.048165**

**499_cam1_1.bmp 579_frontal.bmp 0.064475**

**500_cam1_1.bmp 314_frontal.bmp 0.065155**

**501_cam1_1.bmp 158_frontal.bmp 0.066773**

**502_cam1_1.bmp 562_frontal.bmp 0.050020**

**503_cam1_1.bmp 224_frontal.bmp 0.057739**

**504_cam1_1.bmp 559_frontal.bmp 0.046490**

**505_cam1_1.bmp 320_frontal.bmp 0.044504**

**506_cam1_1.bmp 506_frontal.bmp 0.051643**

**507_cam1_1.bmp 253_frontal.bmp 0.142262**

**508_cam1_1.bmp 350_frontal.bmp 0.188386**

**509_cam1_1.bmp 155_frontal.bmp 0.045848**

**510_cam1_1.bmp 313_frontal.bmp 0.084308**

**511_cam1_1.bmp 511_frontal.bmp 0.067499**

**512_cam1_1.bmp 691_frontal.bmp 0.113561**

**513_cam1_1.bmp 624_frontal.bmp 0.042530**

**514_cam1_1.bmp 204_frontal.bmp 0.062948**

**515_cam1_1.bmp 253_frontal.bmp 0.169755**

**516_cam1_1.bmp 314_frontal.bmp 0.091808**

**517_cam1_1.bmp 281_frontal.bmp 0.223440**

**518_cam1_1.bmp 286_frontal.bmp 0.069093**

**519_cam1_1.bmp 519_frontal.bmp 0.077982**

**520_cam1_1.bmp 528_frontal.bmp 0.136186**

**521_cam1_1.bmp 521_frontal.bmp 0.113340**

**522_cam1_1.bmp 244_frontal.bmp 0.231389**

**523_cam1_1.bmp 523_frontal.bmp 0.057889**

**524_cam1_1.bmp 524_frontal.bmp 0.205976**

**525_cam1_1.bmp 525_frontal.bmp 0.043411**

**526_cam1_1.bmp 322_frontal.bmp 0.062658**

**527_cam1_1.bmp 527_frontal.bmp 0.072695**

**528_cam1_1.bmp 528_frontal.bmp 0.083074**

**529_cam1_1.bmp 529_frontal.bmp 0.104262**

**530_cam1_1.bmp 557_frontal.bmp 0.064745**

**531_cam1_1.bmp 531_frontal.bmp 0.084930**

**532_cam1_1.bmp 5325_frontal.bmp 0.060897**

**533_cam1_1.bmp 533_frontal.bmp 0.201032**

**534_cam1_1.bmp 670_frontal.bmp 0.091903**

**535_cam1_1.bmp 486_frontal.bmp 0.083449**

**536_cam1_1.bmp 053_frontal.bmp 0.050405**

**537_cam1_1.bmp 691_frontal.bmp 0.089026**

**538_cam1_1.bmp 538_frontal.bmp 0.116275**

**539_cam1_1.bmp 415_frontal.bmp 0.097308**

**540_cam1_1.bmp 540_frontal.bmp 0.094515**

**541_cam1_1.bmp 541_frontal.bmp 0.085187**

**542_cam1_1.bmp 314_frontal.bmp 0.105953**

**543_cam1_1.bmp 543_frontal.bmp 0.079843**

**544_cam1_1.bmp 573_frontal.bmp 0.074323**

**545_cam1_1.bmp 722_frontal.bmp 0.043715**

**546_cam1_1.bmp 546_frontal.bmp 0.081915**

**547_cam1_1.bmp 320_frontal.bmp 0.089762**

**548_cam1_1.bmp 548_frontal.bmp 0.064382**

**549_cam1_1.bmp 549_frontal.bmp 0.059247**

**550_cam1_1.bmp 314_frontal.bmp 0.049974**

**551_cam1_1.bmp 310_frontal.bmp 0.055167**

**552_cam1_1.bmp 697_frontal.bmp 0.077049**

**553_cam1_1.bmp 365_frontal.bmp 0.047510**

**554_cam1_1.bmp 554_frontal.bmp 0.060149**

**555_cam1_1.bmp 555_frontal.bmp 0.087316**

**556_cam1_1.bmp 556_frontal.bmp 0.074511**

**557_cam1_1.bmp 172_frontal.bmp 0.067514**

**558_cam1_1.bmp 558_frontal.bmp 0.080711**

**559_cam1_1.bmp 559_frontal.bmp 0.058571**

**560_cam1_1.bmp 560_frontal.bmp 0.064978**

**561_cam1_1.bmp 561_frontal.bmp 0.052154**

**562_cam1_1.bmp 562_frontal.bmp 0.057671**

**563_cam1_1.bmp 313_frontal.bmp 0.063031**

**564_cam1_1.bmp 691_frontal.bmp 0.070070**

**565_cam1_1.bmp 565_frontal.bmp 0.103662**

**566_cam1_1.bmp 566_frontal.bmp 0.143728**

**567_cam1_1.bmp 567_frontal.bmp 0.079522**

**568_cam1_1.bmp 675_frontal.bmp 0.112689**

**569_cam1_1.bmp 691_frontal.bmp 0.072721**

**570_cam1_1.bmp 709_frontal.bmp 0.075974**

**571_cam1_1.bmp 568_frontal.bmp 0.073201**

**572_cam1_1.bmp 326_frontal.bmp 0.052166**

**573_cam1_1.bmp 573_frontal.bmp 0.149432**

**574_cam1_1.bmp 574_frontal.bmp 0.046238**

**575_cam1_1.bmp 575_frontal.bmp 0.146546**

**576_cam1_1.bmp 709_frontal.bmp 0.075930**

**577_cam1_1.bmp 577_frontal.bmp 0.148419**

**578_cam1_1.bmp 578_frontal.bmp 0.087022**

**579_cam1_1.bmp 566_frontal.bmp 0.096565**

**580_cam1_1.bmp 563_frontal.bmp 0.066319**

**581_cam1_1.bmp 581_frontal.bmp 0.154908**

**582_cam1_1.bmp 582_frontal.bmp 0.110643**

**583_cam1_1.bmp 583_frontal.bmp 0.123214**

**584_cam1_1.bmp 584_frontal.bmp 0.145672**

**585_cam1_1.bmp 585_frontal.bmp 0.083396**

**586_cam1_1.bmp 586_frontal.bmp 0.039196**

**587_cam1_1.bmp 587_frontal.bmp 0.120561**

**588_cam1_1.bmp 337_frontal.bmp 0.083992**

**589_cam1_1.bmp 324_frontal.bmp 0.065255**

**590_cam1_1.bmp 590_frontal.bmp 0.108035**

**591_cam1_1.bmp 415_frontal.bmp 0.146546**

**592_cam1_1.bmp 372_frontal.bmp 0.058938**

**593_cam1_1.bmp 359_frontal.bmp 0.062585**

**594_cam1_1.bmp 711_frontal.bmp 0.064193**

**595_cam1_1.bmp 595_frontal.bmp 0.055366**

**596_cam1_1.bmp 568_frontal.bmp 0.060744**

**597_cam1_1.bmp 240_frontal.bmp 0.073552**

**598_cam1_1.bmp 313_frontal.bmp 0.052105**

**599_cam1_1.bmp 134_frontal.bmp 0.057149**

**600_cam1_1.bmp 600_frontal.bmp 0.096221**

**601_cam1_1.bmp 585_frontal.bmp 0.075928**

**602_cam1_1.bmp 231_frontal.bmp 0.071592**

**603_cam1_1.bmp 415_frontal.bmp 0.075501**

**604_cam1_1.bmp 574_frontal.bmp 0.052713**

**605_cam1_1.bmp 691_frontal.bmp 0.089940**

**606_cam1_1.bmp 313_frontal.bmp 0.052525**

**607_cam1_1.bmp 326_frontal.bmp 0.047233**

**608_cam1_1.bmp 691_frontal.bmp 0.069286**

**609_cam1_1.bmp 372_frontal.bmp 0.066625**

**610_cam1_1.bmp 326_frontal.bmp 0.079047**

**611_cam1_1.bmp 521_frontal.bmp 0.045643**

**612_cam1_1.bmp 456_frontal.bmp 0.049794**

**613_cam1_1.bmp 313_frontal.bmp 0.059032**

**614_cam1_1.bmp 313_frontal.bmp 0.072233**

**615_cam1_1.bmp 615_frontal.bmp 0.083948**

**616_cam1_1.bmp 174_frontal.bmp 0.067605**

**617_cam1_1.bmp 339_frontal.bmp 0.056173**

**618_cam1_1.bmp 575_frontal.bmp 0.067832**

**619_cam1_1.bmp 670_frontal.bmp 0.058697**

**620_cam1_1.bmp 243_frontal.bmp 0.070424**

**621_cam1_1.bmp 691_frontal.bmp 0.059574**

**622_cam1_1.bmp 372_frontal.bmp 0.063636**

**623_cam1_1.bmp 697_frontal.bmp 0.047891**

**624_cam1_1.bmp 624_frontal.bmp 0.063290**

**625_cam1_1.bmp 124_frontal.bmp 0.053623**

**626_cam1_1.bmp 288_frontal.bmp 0.045835**

**627_cam1_1.bmp 310_frontal.bmp 0.060099**

**628_cam1_1.bmp 695_frontal.bmp 0.038459**

**629_cam1_1.bmp 151_frontal.bmp 0.052428**

**630_cam1_1.bmp 174_frontal.bmp 0.056275**

**631_cam1_1.bmp 597_frontal.bmp 0.042787**

**632_cam1_1.bmp 336_frontal.bmp 0.048024**

**633_cam1_1.bmp 313_frontal.bmp 0.066985**

**634_cam1_1.bmp 557_frontal.bmp 0.085708**

**635_cam1_1.bmp 104_frontal.bmp 0.045835**

**636_cam1_1.bmp 337_frontal.bmp 0.046911**

**637_cam1_1.bmp 590_frontal.bmp 0.051703**

**638_cam1_1.bmp 155_frontal.bmp 0.058486**

**639_cam1_1.bmp 460_frontal.bmp 0.064999**

**640_cam1_1.bmp 559_frontal.bmp 0.046705**

**641_cam1_1.bmp 337_frontal.bmp 0.067940**

**642_cam1_1.bmp 670_frontal.bmp 0.153332**

**643_cam1_1.bmp 697_frontal.bmp 0.068724**

**644_cam1_1.bmp 387_frontal.bmp 0.048201**

**645_cam1_1.bmp 645_frontal.bmp 0.056794**

**646_cam1_1.bmp 313_frontal.bmp 0.049364**

**647_cam1_1.bmp 647_frontal.bmp 0.053863**

**648_cam1_1.bmp 387_frontal.bmp 0.069136**

**649_cam1_1.bmp 565_frontal.bmp 0.054533**

**650_cam1_1.bmp 362_frontal.bmp 0.063934**

**651_cam1_1.bmp 126_frontal.bmp 0.059669**

**652_cam1_1.bmp 337_frontal.bmp 0.061588**

**653_cam1_1.bmp 691_frontal.bmp 0.072718**

**654_cam1_1.bmp 691_frontal.bmp 0.076242**

**655_cam1_1.bmp 691_frontal.bmp 0.052866**

**656_cam1_1.bmp 322_frontal.bmp 0.048559**

**657_cam1_1.bmp 598_frontal.bmp 0.056821**

**658_cam1_1.bmp 134_frontal.bmp 0.048752**

**659_cam1_1.bmp 359_frontal.bmp 0.052447**

**660_cam1_1.bmp 339_frontal.bmp 0.045623**

**661_cam1_1.bmp 661_frontal.bmp 0.038659**

**662_cam1_1.bmp 662_frontal.bmp 0.056691**

**663_cam1_1.bmp 353_frontal.bmp 0.059735**

**664_cam1_1.bmp 670_frontal.bmp 0.138116**

**665_cam1_1.bmp 211_frontal.bmp 0.059903**

**666_cam1_1.bmp 691_frontal.bmp 0.087023**

**667_cam1_1.bmp 246_frontal.bmp 0.091263**

**668_cam1_1.bmp 573_frontal.bmp 0.050847**

**669_cam1_1.bmp 313_frontal.bmp 0.066200**

**670_cam1_1.bmp 188_frontal.bmp 0.048534**

**671_cam1_1.bmp 568_frontal.bmp 0.120493**

**672_cam1_1.bmp 691_frontal.bmp 0.069815**

**673_cam1_1.bmp 344_frontal.bmp 0.053368**

**674_cam1_1.bmp 314_frontal.bmp 0.097725**

**675_cam1_1.bmp 155_frontal.bmp 0.068212**

**676_cam1_1.bmp 053_frontal.bmp 0.076592**

**677_cam1_1.bmp 691_frontal.bmp 0.065774**

**678_cam1_1.bmp 568_frontal.bmp 0.069969**

**679_cam1_1.bmp 565_frontal.bmp 0.043344**

**680_cam1_1.bmp 379_frontal.bmp 0.036296**

**681_cam1_1.bmp 313_frontal.bmp 0.102017**

**682_cam1_1.bmp 538_frontal.bmp 0.123255**

**683_cam1_1.bmp 267_frontal.bmp 0.071096**

**684_cam1_1.bmp 684_frontal.bmp 0.093584**

**685_cam1_1.bmp 155_frontal.bmp 0.106280**

**686_cam1_1.bmp 697_frontal.bmp 0.051953**

**687_cam1_1.bmp 285_frontal.bmp 0.094191**

**688_cam1_1.bmp 422_frontal.bmp 0.059152**

**689_cam1_1.bmp 211_frontal.bmp 0.185864**

**690_cam1_1.bmp 250_frontal.bmp 0.163202**

**691_cam1_1.bmp 691_frontal.bmp 0.104659**

**692_cam1_1.bmp 002_frontal.bmp 0.048373**

**693_cam1_1.bmp 693_frontal.bmp 0.074566**

**694_cam1_1.bmp 528_frontal.bmp 0.233029**

**695_cam1_1.bmp 213_frontal.bmp 0.134031**

**696_cam1_1.bmp 349_frontal.bmp 0.028701**

**697_cam1_1.bmp 284_frontal.bmp 0.263554**

**698_cam1_1.bmp 169_frontal.bmp 0.107041**

**699_cam1_1.bmp 699_frontal.bmp 0.128208**

**700_cam1_1.bmp 642_frontal.bmp 0.083163**

**701_cam1_1.bmp 244_frontal.bmp 0.181111**

**702_cam1_1.bmp 245_frontal.bmp 0.215742**

**703_cam1_1.bmp 528_frontal.bmp 0.086170**

**704_cam1_1.bmp 511_frontal.bmp 0.034692**

**705_cam1_1.bmp 405_frontal.bmp 0.202464**

**706_cam1_1.bmp 245_frontal.bmp 0.153849**

**707_cam1_1.bmp 372_frontal.bmp 0.071640**

**708_cam1_1.bmp 009_frontal.bmp 0.103081**

**709_cam1_1.bmp 709_frontal.bmp 0.071085**

**710_cam1_1.bmp 275_frontal.bmp 0.140610**

**711_cam1_1.bmp 691_frontal.bmp 0.103978**

**712_cam1_1.bmp 053_frontal.bmp 0.110304**

**713_cam1_1.bmp 256_frontal.bmp 0.145331**

**714_cam1_1.bmp 253_frontal.bmp 0.171334**

**715_cam1_1.bmp 253_frontal.bmp 0.103373**

**716_cam1_1.bmp 716_frontal.bmp 0.060586**

**717_cam1_1.bmp 691_frontal.bmp 0.076083**

**718_cam1_1.bmp 287_frontal.bmp 0.219437**

**719_cam1_1.bmp 284_frontal.bmp 0.166801**

**720_cam1_1.bmp 558_frontal.bmp 0.076125**

**721_cam1_1.bmp 273_frontal.bmp 0.209206**

**722_cam1_1.bmp 455_frontal.bmp 0.116166**

**723_cam1_1.bmp 250_frontal.bmp 0.123370**

**724_cam1_1.bmp 566_frontal.bmp 0.123978**

**725_cam1_1.bmp 725_frontal.bmp 0.070586**

**726_cam1_1.bmp 227_frontal.bmp 0.208855**

**727_cam1_1.bmp 544_frontal.bmp 0.108246**

**728_cam1_1.bmp 691_frontal.bmp 0.080733**

**729_cam1_1.bmp 253_frontal.bmp 0.114326**

**730_cam1_1.bmp 435_frontal.bmp 0.102962**

**731_cam1_1.bmp 731_frontal.bmp 0.053452**

**732_cam1_1.bmp 732_frontal.bmp 0.086467**

**733_cam1_1.bmp 245_frontal.bmp 0.152818**

**734_cam1_1.bmp 253_frontal.bmp 0.180097**

**735_cam1_1.bmp 227_frontal.bmp 0.237472**

**736_cam1_1.bmp 281_frontal.bmp 0.224334**

**737_cam1_1.bmp 737_frontal.bmp 0.043025**

**738_cam1_1.bmp 547_frontal.bmp 0.162474**

**739_cam1_1.bmp 739_frontal.bmp 0.111948**

**740_cam1_1.bmp 253_frontal.bmp 0.176772**

**Final Score NIR-VIS FSR**

**Actual Image Recognized Image Score**

**001_cam1_1.bmp 001_frontal.bmp 0.083546**

**002_cam1_1.bmp 053_frontal.bmp 0.118324**

**003_cam1_1.bmp 559_frontal.bmp 0.058980**

**004_cam1_1.bmp 004_frontal.bmp 0.055951**

**005_cam1_1.bmp 350_frontal.bmp 0.050786**

**006_cam1_1.bmp 132_frontal.bmp 0.082840**

**007_cam1_1.bmp 007_frontal.bmp 0.052689**

**008_cam1_1.bmp 732_frontal.bmp 0.072812**

**009_cam1_1.bmp 009_frontal.bmp 0.133981**

**010_cam1_1.bmp 317_frontal.bmp 0.064423**

**011_cam1_1.bmp 555_frontal.bmp 0.058506**

**012_cam1_1.bmp 342_frontal.bmp 0.071528**

**013_cam1_1.bmp 035_frontal.bmp 0.060330**

**014_cam1_1.bmp 691_frontal.bmp 0.101960**

**015_cam1_1.bmp 015_frontal.bmp 0.100365**

**016_cam1_1.bmp 555_frontal.bmp 0.061995**

**017_cam1_1.bmp 174_frontal.bmp 0.088849**

**018_cam1_1.bmp 313_frontal.bmp 0.107175**

**019_cam1_1.bmp 697_frontal.bmp 0.122253**

**020_cam1_1.bmp 188_frontal.bmp 0.092987**

**021_cam1_1.bmp 423_frontal.bmp 0.055880**

**022_cam1_1.bmp 186_frontal.bmp 0.075868**

**023_cam1_1.bmp 566_frontal.bmp 0.069250**

**024_cam1_1.bmp 173_frontal.bmp 0.077386**

**025_cam1_1.bmp 320_frontal.bmp 0.055745**

**026_cam1_1.bmp 691_frontal.bmp 0.070904**

**027_cam1_1.bmp 155_frontal.bmp 0.080825**

**028_cam1_1.bmp 104_frontal.bmp 0.073539**

**029_cam1_1.bmp 029_frontal.bmp 0.076227**

**030_cam1_1.bmp 697_frontal.bmp 0.101719**

**031_cam1_1.bmp 697_frontal.bmp 0.085701**

**032_cam1_1.bmp 555_frontal.bmp 0.078528**

**033_cam1_1.bmp 174_frontal.bmp 0.058933**

**034_cam1_1.bmp 174_frontal.bmp 0.094617**

**035_cam1_1.bmp 185_frontal.bmp 0.050209**

**036_cam1_1.bmp 017_frontal.bmp 0.108328**

**037_cam1_1.bmp 569_frontal.bmp 0.065846**

**038_cam1_1.bmp 155_frontal.bmp 0.077270**

**039_cam1_1.bmp 313_frontal.bmp 0.062282**

**040_cam1_1.bmp 174_frontal.bmp 0.067758**

**041_cam1_1.bmp 042_frontal.bmp 0.078410**

**042_cam1_1.bmp 042_frontal.bmp 0.122582**

**043_cam1_1.bmp 569_frontal.bmp 0.062917**

**044_cam1_1.bmp 211_frontal.bmp 0.084912**

**045_cam1_1.bmp 336_frontal.bmp 0.094242**

**046_cam1_1.bmp 155_frontal.bmp 0.096622**

**047_cam1_1.bmp 174_frontal.bmp 0.071336**

**048_cam1_1.bmp 199_frontal.bmp 0.063842**

**049_cam1_1.bmp 147_frontal.bmp 0.055633**

**050_cam1_1.bmp 697_frontal.bmp 0.073917**

**051_cam1_1.bmp 317_frontal.bmp 0.079716**

**052_cam1_1.bmp 691_frontal.bmp 0.092830**

**053_cam1_1.bmp 339_frontal.bmp 0.099515**

**054_cam1_1.bmp 691_frontal.bmp 0.072053**

**055_cam1_1.bmp 174_frontal.bmp 0.082015**

**056_cam1_1.bmp 056_frontal.bmp 0.105394**

**057_cam1_1.bmp 188_frontal.bmp 0.086176**

**058_cam1_1.bmp 348_frontal.bmp 0.083554**

**059_cam1_1.bmp 555_frontal.bmp 0.051895**

**060_cam1_1.bmp 090_frontal.bmp 0.086465**

**061_cam1_1.bmp 078_frontal.bmp 0.079612**

**062_cam1_1.bmp 601_frontal.bmp 0.085242**

**063_cam1_1.bmp 691_frontal.bmp 0.088115**

**064_cam1_1.bmp 575_frontal.bmp 0.096952**

**065_cam1_1.bmp 732_frontal.bmp 0.087766**

**066_cam1_1.bmp 066_frontal.bmp 0.123275**

**067_cam1_1.bmp 339_frontal.bmp 0.074111**

**068_cam1_1.bmp 575_frontal.bmp 0.046900**

**069_cam1_1.bmp 069_frontal.bmp 0.059220**

**070_cam1_1.bmp 320_frontal.bmp 0.079956**

**071_cam1_1.bmp 313_frontal.bmp 0.050550**

**072_cam1_1.bmp 732_frontal.bmp 0.072211**

**073_cam1_1.bmp 324_frontal.bmp 0.068076**

**074_cam1_1.bmp 330_frontal.bmp 0.089548**

**075_cam1_1.bmp 075_frontal.bmp 0.079622**

**076_cam1_1.bmp 475_frontal.bmp 0.083744**

**077_cam1_1.bmp 171_frontal.bmp 0.064371**

**078_cam1_1.bmp 708_frontal.bmp 0.073009**

**079_cam1_1.bmp 324_frontal.bmp 0.097127**

**080_cam1_1.bmp 691_frontal.bmp 0.079536**

**081_cam1_1.bmp 534_frontal.bmp 0.078220**

**082_cam1_1.bmp 082_frontal.bmp 0.095596**

**083_cam1_1.bmp 328_frontal.bmp 0.064438**

**084_cam1_1.bmp 084_frontal.bmp 0.069847**

**085_cam1_1.bmp 737_frontal.bmp 0.054290**

**086_cam1_1.bmp 569_frontal.bmp 0.072744**

**087_cam1_1.bmp 147_frontal.bmp 0.081376**

**088_cam1_1.bmp 155_frontal.bmp 0.149092**

**089_cam1_1.bmp 697_frontal.bmp 0.094322**

**090_cam1_1.bmp 090_frontal.bmp 0.119947**

**091_cam1_1.bmp 348_frontal.bmp 0.077024**

**092_cam1_1.bmp 231_frontal.bmp 0.077749**

**093_cam1_1.bmp 328_frontal.bmp 0.051400**

**094_cam1_1.bmp 035_frontal.bmp 0.063102**

**095_cam1_1.bmp 523_frontal.bmp 0.054027**

**096_cam1_1.bmp 555_frontal.bmp 0.062414**

**097_cam1_1.bmp 313_frontal.bmp 0.061172**

**098_cam1_1.bmp 326_frontal.bmp 0.076985**

**099_cam1_1.bmp 174_frontal.bmp 0.139507**

**100_cam1_1.bmp 732_frontal.bmp 0.074365**

**101_cam1_1.bmp 691_frontal.bmp 0.068091**

**102_cam1_1.bmp 559_frontal.bmp 0.087821**

**103_cam1_1.bmp 155_frontal.bmp 0.114596**

**104_cam1_1.bmp 524_frontal.bmp 0.077892**

**105_cam1_1.bmp 155_frontal.bmp 0.077269**

**106_cam1_1.bmp 544_frontal.bmp 0.083722**

**107_cam1_1.bmp 523_frontal.bmp 0.078467**

**108_cam1_1.bmp 562_frontal.bmp 0.089398**

**109_cam1_1.bmp 348_frontal.bmp 0.126285**

**110_cam1_1.bmp 320_frontal.bmp 0.075401**

**111_cam1_1.bmp 324_frontal.bmp 0.090810**

**112_cam1_1.bmp 174_frontal.bmp 0.086819**

**113_cam1_1.bmp 326_frontal.bmp 0.064490**

**114_cam1_1.bmp 313_frontal.bmp 0.076741**

**115_cam1_1.bmp 056_frontal.bmp 0.155803**

**116_cam1_1.bmp 155_frontal.bmp 0.101419**

**117_cam1_1.bmp 199_frontal.bmp 0.065537**

**118_cam1_1.bmp 174_frontal.bmp 0.131107**

**119_cam1_1.bmp 732_frontal.bmp 0.071901**

**120_cam1_1.bmp 691_frontal.bmp 0.085557**

**121_cam1_1.bmp 271_frontal.bmp 0.069302**

**122_cam1_1.bmp 155_frontal.bmp 0.104238**

**123_cam1_1.bmp 209_frontal.bmp 0.133196**

**124_cam1_1.bmp 124_frontal.bmp 0.115256**

**125_cam1_1.bmp 691_frontal.bmp 0.087259**

**126_cam1_1.bmp 691_frontal.bmp 0.115123**

**127_cam1_1.bmp 691_frontal.bmp 0.079494**

**128_cam1_1.bmp 271_frontal.bmp 0.056867**

**129_cam1_1.bmp 313_frontal.bmp 0.105848**

**130_cam1_1.bmp 555_frontal.bmp 0.050199**

**131_cam1_1.bmp 265_frontal.bmp 0.073581**

**132_cam1_1.bmp 317_frontal.bmp 0.073359**

**133_cam1_1.bmp 271_frontal.bmp 0.109137**

**134_cam1_1.bmp 134_frontal.bmp 0.087483**

**135_cam1_1.bmp 174_frontal.bmp 0.102773**

**136_cam1_1.bmp 555_frontal.bmp 0.073646**

**137_cam1_1.bmp 317_frontal.bmp 0.058286**

**138_cam1_1.bmp 555_frontal.bmp 0.049999**

**139_cam1_1.bmp 691_frontal.bmp 0.115027**

**140_cam1_1.bmp 691_frontal.bmp 0.074986**

**141_cam1_1.bmp 691_frontal.bmp 0.073968**

**142_cam1_1.bmp 723_frontal.bmp 0.093416**

**143_cam1_1.bmp 342_frontal.bmp 0.063805**

**144_cam1_1.bmp 313_frontal.bmp 0.071330**

**145_cam1_1.bmp 002_frontal.bmp 0.060710**

**146_cam1_1.bmp 555_frontal.bmp 0.077721**

**147_cam1_1.bmp 317_frontal.bmp 0.081336**

**148_cam1_1.bmp 555_frontal.bmp 0.080495**

**149_cam1_1.bmp 523_frontal.bmp 0.085383**

**150_cam1_1.bmp 555_frontal.bmp 0.055750**

**151_cam1_1.bmp 166_frontal.bmp 0.081821**

**152_cam1_1.bmp 559_frontal.bmp 0.052642**

**153_cam1_1.bmp 153_frontal.bmp 0.061970**

**154_cam1_1.bmp 155_frontal.bmp 0.104899**

**155_cam1_1.bmp 155_frontal.bmp 0.194737**

**156_cam1_1.bmp 202_frontal.bmp 0.044150**

**157_cam1_1.bmp 271_frontal.bmp 0.102576**

**158_cam1_1.bmp 691_frontal.bmp 0.063219**

**159_cam1_1.bmp 155_frontal.bmp 0.111937**

**160_cam1_1.bmp 555_frontal.bmp 0.066567**

**161_cam1_1.bmp 161_frontal.bmp 0.106117**

**162_cam1_1.bmp 370_frontal.bmp 0.057211**

**163_cam1_1.bmp 163_frontal.bmp 0.062041**

**164_cam1_1.bmp 555_frontal.bmp 0.054280**

**165_cam1_1.bmp 165_frontal.bmp 0.100160**

**166_cam1_1.bmp 155_frontal.bmp 0.151669**

**167_cam1_1.bmp 056_frontal.bmp 0.129289**

**168_cam1_1.bmp 155_frontal.bmp 0.110642**

**169_cam1_1.bmp 169_frontal.bmp 0.120171**

**170_cam1_1.bmp 180_frontal.bmp 0.068632**

**171_cam1_1.bmp 171_frontal.bmp 0.085550**

**172_cam1_1.bmp 561_frontal.bmp 0.056380**

**173_cam1_1.bmp 348_frontal.bmp 0.107959**

**174_cam1_1.bmp 174_frontal.bmp 0.184298**

**175_cam1_1.bmp 155_frontal.bmp 0.093975**

**176_cam1_1.bmp 585_frontal.bmp 0.083245**

**177_cam1_1.bmp 555_frontal.bmp 0.064704**

**178_cam1_1.bmp 073_frontal.bmp 0.057163**

**179_cam1_1.bmp 056_frontal.bmp 0.113170**

**180_cam1_1.bmp 697_frontal.bmp 0.106756**

**181_cam1_1.bmp 184_frontal.bmp 0.082665**

**182_cam1_1.bmp 155_frontal.bmp 0.101586**

**183_cam1_1.bmp 066_frontal.bmp 0.143172**

**184_cam1_1.bmp 448_frontal.bmp 0.080605**

**185_cam1_1.bmp 691_frontal.bmp 0.095900**

**186_cam1_1.bmp 186_frontal.bmp 0.093862**

**187_cam1_1.bmp 174_frontal.bmp 0.072913**

**188_cam1_1.bmp 697_frontal.bmp 0.107898**

**189_cam1_1.bmp 559_frontal.bmp 0.056404**

**190_cam1_1.bmp 697_frontal.bmp 0.085490**

**191_cam1_1.bmp 056_frontal.bmp 0.098361**

**192_cam1_1.bmp 192_frontal.bmp 0.075674**

**193_cam1_1.bmp 235_frontal.bmp 0.057902**

**194_cam1_1.bmp 174_frontal.bmp 0.057390**

**195_cam1_1.bmp 575_frontal.bmp 0.055950**

**196_cam1_1.bmp 174_frontal.bmp 0.072307**

**197_cam1_1.bmp 271_frontal.bmp 0.064853**

**198_cam1_1.bmp 555_frontal.bmp 0.054886**

**199_cam1_1.bmp 199_frontal.bmp 0.036832**

**200_cam1_1.bmp 174_frontal.bmp 0.058682**

**201_cam1_1.bmp 339_frontal.bmp 0.059525**

**202_cam1_1.bmp 372_frontal.bmp 0.054234**

**203_cam1_1.bmp 334_frontal.bmp 0.173843**

**204_cam1_1.bmp 253_frontal.bmp 0.175191**

**205_cam1_1.bmp 313_frontal.bmp 0.045733**

**206_cam1_1.bmp 281_frontal.bmp 0.124583**

**207_cam1_1.bmp 568_frontal.bmp 0.224068**

**208_cam1_1.bmp 598_frontal.bmp 0.122770**

**209_cam1_1.bmp 209_frontal.bmp 0.103174**

**210_cam1_1.bmp 284_frontal.bmp 0.202733**

**211_cam1_1.bmp 253_frontal.bmp 0.134225**

**212_cam1_1.bmp 691_frontal.bmp 0.087333**

**213_cam1_1.bmp 213_frontal.bmp 0.158882**

**214_cam1_1.bmp 214_frontal.bmp 0.063143**

**215_cam1_1.bmp 348_frontal.bmp 0.058953**

**216_cam1_1.bmp 285_frontal.bmp 0.059681**

**217_cam1_1.bmp 330_frontal.bmp 0.053418**

**218_cam1_1.bmp 691_frontal.bmp 0.081789**

**219_cam1_1.bmp 598_frontal.bmp 0.122342**

**220_cam1_1.bmp 220_frontal.bmp 0.115641**

**221_cam1_1.bmp 243_frontal.bmp 0.078086**

**222_cam1_1.bmp 155_frontal.bmp 0.085507**

**223_cam1_1.bmp 223_frontal.bmp 0.068893**

**224_cam1_1.bmp 224_frontal.bmp 0.064587**

**225_cam1_1.bmp 417_frontal.bmp 0.064869**

**226_cam1_1.bmp 334_frontal.bmp 0.121499**

**227_cam1_1.bmp 670_frontal.bmp 0.154682**

**228_cam1_1.bmp 155_frontal.bmp 0.086257**

**229_cam1_1.bmp 730_frontal.bmp 0.058208**

**230_cam1_1.bmp 230_frontal.bmp 0.100246**

**231_cam1_1.bmp 231_frontal.bmp 0.102369**

**232_cam1_1.bmp 186_frontal.bmp 0.066756**

**233_cam1_1.bmp 310_frontal.bmp 0.039523**

**234_cam1_1.bmp 234_frontal.bmp 0.091051**

**235_cam1_1.bmp 314_frontal.bmp 0.093049**

**236_cam1_1.bmp 235_frontal.bmp 0.098918**

**237_cam1_1.bmp 237_frontal.bmp 0.108958**

**238_cam1_1.bmp 362_frontal.bmp 0.052113**

**239_cam1_1.bmp 224_frontal.bmp 0.060524**

**240_cam1_1.bmp 712_frontal.bmp 0.053363**

**241_cam1_1.bmp 244_frontal.bmp 0.185647**

**242_cam1_1.bmp 242_frontal.bmp 0.054893**

**243_cam1_1.bmp 281_frontal.bmp 0.120807**

**244_cam1_1.bmp 244_frontal.bmp 0.147955**

**245_cam1_1.bmp 245_frontal.bmp 0.133333**

**246_cam1_1.bmp 263_frontal.bmp 0.147534**

**247_cam1_1.bmp 572_frontal.bmp 0.151806**

**248_cam1_1.bmp 248_frontal.bmp 0.252941**

**249_cam1_1.bmp 249_frontal.bmp 0.172044**

**250_cam1_1.bmp 691_frontal.bmp 0.103343**

**251_cam1_1.bmp 379_frontal.bmp 0.090612**

**252_cam1_1.bmp 562_frontal.bmp 0.036992**

**253_cam1_1.bmp 253_frontal.bmp 0.295332**

**254_cam1_1.bmp 254_frontal.bmp 0.126033**

**255_cam1_1.bmp 711_frontal.bmp 0.066418**

**256_cam1_1.bmp 256_frontal.bmp 0.160246**

**257_cam1_1.bmp 257_frontal.bmp 0.076479**

**258_cam1_1.bmp 281_frontal.bmp 0.138848**

**259_cam1_1.bmp 725_frontal.bmp 0.054712**

**260_cam1_1.bmp 260_frontal.bmp 0.087330**

**261_cam1_1.bmp 273_frontal.bmp 0.186532**

**262_cam1_1.bmp 088_frontal.bmp 0.053202**

**263_cam1_1.bmp 227_frontal.bmp 0.194441**

**264_cam1_1.bmp 372_frontal.bmp 0.053160**

**265_cam1_1.bmp 265_frontal.bmp 0.176447**

**266_cam1_1.bmp 266_frontal.bmp 0.129704**

**267_cam1_1.bmp 575_frontal.bmp 0.068669**

**268_cam1_1.bmp 342_frontal.bmp 0.171262**

**269_cam1_1.bmp 348_frontal.bmp 0.098560**

**270_cam1_1.bmp 270_frontal.bmp 0.085534**

**271_cam1_1.bmp 691_frontal.bmp 0.088241**

**272_cam1_1.bmp 253_frontal.bmp 0.134214**

**273_cam1_1.bmp 320_frontal.bmp 0.086006**

**274_cam1_1.bmp 334_frontal.bmp 0.096528**

**275_cam1_1.bmp 275_frontal.bmp 0.122242**

**276_cam1_1.bmp 276_frontal.bmp 0.052295**

**277_cam1_1.bmp 384_frontal.bmp 0.067456**

**278_cam1_1.bmp 370_frontal.bmp 0.051238**

**279_cam1_1.bmp 279_frontal.bmp 0.063159**

**280_cam1_1.bmp 273_frontal.bmp 0.209752**

**281_cam1_1.bmp 281_frontal.bmp 0.206179**

**282_cam1_1.bmp 243_frontal.bmp 0.201536**

**283_cam1_1.bmp 283_frontal.bmp 0.109077**

**284_cam1_1.bmp 284_frontal.bmp 0.180039**

**285_cam1_1.bmp 697_frontal.bmp 0.054873**

**286_cam1_1.bmp 253_frontal.bmp 0.148118**

**287_cam1_1.bmp 484_frontal.bmp 0.151863**

**288_cam1_1.bmp 691_frontal.bmp 0.049306**

**289_cam1_1.bmp 691_frontal.bmp 0.057496**

**290_cam1_1.bmp 290_frontal.bmp 0.103019**

**291_cam1_1.bmp 281_frontal.bmp 0.172072**

**292_cam1_1.bmp 208_frontal.bmp 0.108593**

**293_cam1_1.bmp 709_frontal.bmp 0.055054**

**294_cam1_1.bmp 287_frontal.bmp 0.126811**

**295_cam1_1.bmp 334_frontal.bmp 0.175072**

**296_cam1_1.bmp 556_frontal.bmp 0.054905**

**297_cam1_1.bmp 662_frontal.bmp 0.107594**

**298_cam1_1.bmp 359_frontal.bmp 0.052796**

**299_cam1_1.bmp 287_frontal.bmp 0.133745**

**300_cam1_1.bmp 281_frontal.bmp 0.123230**

**301_cam1_1.bmp 350_frontal.bmp 0.143355**

**302_cam1_1.bmp 263_frontal.bmp 0.225099**

**303_cam1_1.bmp 544_frontal.bmp 0.230434**

**304_cam1_1.bmp 282_frontal.bmp 0.050209**

**305_cam1_1.bmp 521_frontal.bmp 0.051234**

**306_cam1_1.bmp 155_frontal.bmp 0.087942**

**307_cam1_1.bmp 307_frontal.bmp 0.120956**

**308_cam1_1.bmp 308_frontal.bmp 0.123426**

**309_cam1_1.bmp 314_frontal.bmp 0.053668**

**310_cam1_1.bmp 310_frontal.bmp 0.114142**

**311_cam1_1.bmp 155_frontal.bmp 0.090496**

**312_cam1_1.bmp 312_frontal.bmp 0.113963**

**313_cam1_1.bmp 313_frontal.bmp 0.106973**

**314_cam1_1.bmp 269_frontal.bmp 0.041810**

**315_cam1_1.bmp 090_frontal.bmp 0.056265**

**316_cam1_1.bmp 316_frontal.bmp 0.069588**

**317_cam1_1.bmp 245_frontal.bmp 0.231697**

**318_cam1_1.bmp 670_frontal.bmp 0.061990**

**319_cam1_1.bmp 319_frontal.bmp 0.087399**

**320_cam1_1.bmp 320_frontal.bmp 0.100317**

**321_cam1_1.bmp 711_frontal.bmp 0.070766**

**322_cam1_1.bmp 322_frontal.bmp 0.077790**

**323_cam1_1.bmp 290_frontal.bmp 0.075141**

**324_cam1_1.bmp 324_frontal.bmp 0.146961**

**325_cam1_1.bmp 563_frontal.bmp 0.034825**

**326_cam1_1.bmp 326_frontal.bmp 0.068676**

**327_cam1_1.bmp 327_frontal.bmp 0.090942**

**328_cam1_1.bmp 328_frontal.bmp 0.079160**

**329_cam1_1.bmp 348_frontal.bmp 0.123465**

**330_cam1_1.bmp 330_frontal.bmp 0.162844**

**331_cam1_1.bmp 320_frontal.bmp 0.071017**

**332_cam1_1.bmp 031_frontal.bmp 0.065305**

**333_cam1_1.bmp 333_frontal.bmp 0.067861**

**334_cam1_1.bmp 334_frontal.bmp 0.084443**

**335_cam1_1.bmp 335_frontal.bmp 0.188059**

**336_cam1_1.bmp 531_frontal.bmp 0.071161**

**337_cam1_1.bmp 337_frontal.bmp 0.115206**

**338_cam1_1.bmp 338_frontal.bmp 0.081946**

**339_cam1_1.bmp 339_frontal.bmp 0.136056**

**340_cam1_1.bmp 340_frontal.bmp 0.141067**

**341_cam1_1.bmp 341_frontal.bmp 0.150948**

**342_cam1_1.bmp 324_frontal.bmp 0.066402**

**343_cam1_1.bmp 343_frontal.bmp 0.100935**

**344_cam1_1.bmp 344_frontal.bmp 0.090575**

**345_cam1_1.bmp 345_frontal.bmp 0.108791**

**346_cam1_1.bmp 346_frontal.bmp 0.109744**

**347_cam1_1.bmp 348_frontal.bmp 0.124065**

**348_cam1_1.bmp 348_frontal.bmp 0.070872**

**349_cam1_1.bmp 349_frontal.bmp 0.103621**

**350_cam1_1.bmp 692_frontal.bmp 0.162484**

**351_cam1_1.bmp 598_frontal.bmp 0.112199**

**352_cam1_1.bmp 662_frontal.bmp 0.062161**

**353_cam1_1.bmp 353_frontal.bmp 0.088793**

**354_cam1_1.bmp 354_frontal.bmp 0.084757**

**355_cam1_1.bmp 250_frontal.bmp 0.085109**

**356_cam1_1.bmp 314_frontal.bmp 0.045365**

**357_cam1_1.bmp 415_frontal.bmp 0.111556**

**358_cam1_1.bmp 415_frontal.bmp 0.069335**

**359_cam1_1.bmp 359_frontal.bmp 0.172270**

**360_cam1_1.bmp 601_frontal.bmp 0.054332**

**361_cam1_1.bmp 361_frontal.bmp 0.094610**

**362_cam1_1.bmp 362_frontal.bmp 0.100736**

**363_cam1_1.bmp 363_frontal.bmp 0.077835**

**364_cam1_1.bmp 364_frontal.bmp 0.106165**

**365_cam1_1.bmp 365_frontal.bmp 0.095764**

**366_cam1_1.bmp 314_frontal.bmp 0.061819**

**367_cam1_1.bmp 348_frontal.bmp 0.097294**

**368_cam1_1.bmp 423_frontal.bmp 0.073267**

**369_cam1_1.bmp 584_frontal.bmp 0.040669**

**370_cam1_1.bmp 310_frontal.bmp 0.066923**

**371_cam1_1.bmp 155_frontal.bmp 0.071162**

**372_cam1_1.bmp 691_frontal.bmp 0.148394**

**373_cam1_1.bmp 610_frontal.bmp 0.145904**

**374_cam1_1.bmp 598_frontal.bmp 0.078243**

**375_cam1_1.bmp 485_frontal.bmp 0.172498**

**376_cam1_1.bmp 376_frontal.bmp 0.074142**

**377_cam1_1.bmp 377_frontal.bmp 0.070316**

**378_cam1_1.bmp 056_frontal.bmp 0.101034**

**379_cam1_1.bmp 697_frontal.bmp 0.079782**

**380_cam1_1.bmp 534_frontal.bmp 0.064836**

**381_cam1_1.bmp 697_frontal.bmp 0.089405**

**382_cam1_1.bmp 314_frontal.bmp 0.074574**

**383_cam1_1.bmp 262_frontal.bmp 0.068647**

**384_cam1_1.bmp 711_frontal.bmp 0.069967**

**385_cam1_1.bmp 455_frontal.bmp 0.057177**

**386_cam1_1.bmp 691_frontal.bmp 0.056377**

**387_cam1_1.bmp 522_frontal.bmp 0.068835**

**388_cam1_1.bmp 691_frontal.bmp 0.078464**

**389_cam1_1.bmp 389_frontal.bmp 0.105230**

**390_cam1_1.bmp 362_frontal.bmp 0.056016**

**391_cam1_1.bmp 697_frontal.bmp 0.052685**

**392_cam1_1.bmp 048_frontal.bmp 0.066946**

**393_cam1_1.bmp 275_frontal.bmp 0.043652**

**394_cam1_1.bmp 394_frontal.bmp 0.041143**

**395_cam1_1.bmp 691_frontal.bmp 0.075282**

**396_cam1_1.bmp 691_frontal.bmp 0.070415**

**397_cam1_1.bmp 556_frontal.bmp 0.028923**

**398_cam1_1.bmp 689_frontal.bmp 0.053665**

**399_cam1_1.bmp 313_frontal.bmp 0.047140**

**400_cam1_1.bmp 053_frontal.bmp 0.055542**

**401_cam1_1.bmp 497_frontal.bmp 0.052314**

**402_cam1_1.bmp 281_frontal.bmp 0.128121**

**403_cam1_1.bmp 231_frontal.bmp 0.041213**

**404_cam1_1.bmp 056_frontal.bmp 0.048098**

**405_cam1_1.bmp 245_frontal.bmp 0.188288**

**406_cam1_1.bmp 566_frontal.bmp 0.059307**

**407_cam1_1.bmp 314_frontal.bmp 0.085792**

**408_cam1_1.bmp 010_frontal.bmp 0.030059**

**409_cam1_1.bmp 053_frontal.bmp 0.050233**

**410_cam1_1.bmp 110_frontal.bmp 0.055999**

**411_cam1_1.bmp 224_frontal.bmp 0.057568**

**412_cam1_1.bmp 724_frontal.bmp 0.038406**

**413_cam1_1.bmp 224_frontal.bmp 0.046733**

**414_cam1_1.bmp 350_frontal.bmp 0.179668**

**415_cam1_1.bmp 387_frontal.bmp 0.063792**

**416_cam1_1.bmp 540_frontal.bmp 0.044527**

**417_cam1_1.bmp 312_frontal.bmp 0.055117**

**418_cam1_1.bmp 012_frontal.bmp 0.050515**

**419_cam1_1.bmp 624_frontal.bmp 0.034357**

**420_cam1_1.bmp 598_frontal.bmp 0.070258**

**421_cam1_1.bmp 053_frontal.bmp 0.057069**

**422_cam1_1.bmp 712_frontal.bmp 0.051605**

**423_cam1_1.bmp 348_frontal.bmp 0.104852**

**424_cam1_1.bmp 042_frontal.bmp 0.043514**

**425_cam1_1.bmp 735_frontal.bmp 0.048060**

**426_cam1_1.bmp 262_frontal.bmp 0.061773**

**427_cam1_1.bmp 350_frontal.bmp 0.098395**

**428_cam1_1.bmp 711_frontal.bmp 0.036027**

**429_cam1_1.bmp 240_frontal.bmp 0.074526**

**430_cam1_1.bmp 550_frontal.bmp 0.167235**

**431_cam1_1.bmp 313_frontal.bmp 0.052643**

**432_cam1_1.bmp 362_frontal.bmp 0.043299**

**433_cam1_1.bmp 433_frontal.bmp 0.343893**

**434_cam1_1.bmp 273_frontal.bmp 0.315586**

**435_cam1_1.bmp 610_frontal.bmp 0.145841**

**436_cam1_1.bmp 691_frontal.bmp 0.060685**

**437_cam1_1.bmp 691_frontal.bmp 0.054840**

**438_cam1_1.bmp 550_frontal.bmp 0.049912**

**439_cam1_1.bmp 469_frontal.bmp 0.040057**

**440_cam1_1.bmp 258_frontal.bmp 0.033129**

**441_cam1_1.bmp 362_frontal.bmp 0.041698**

**442_cam1_1.bmp 582_frontal.bmp 0.070034**

**443_cam1_1.bmp 691_frontal.bmp 0.038053**

**444_cam1_1.bmp 711_frontal.bmp 0.038614**

**445_cam1_1.bmp 339_frontal.bmp 0.051897**

**446_cam1_1.bmp 730_frontal.bmp 0.061993**

**447_cam1_1.bmp 455_frontal.bmp 0.057519**

**448_cam1_1.bmp 448_frontal.bmp 0.086495**

**449_cam1_1.bmp 733_frontal.bmp 0.060838**

**450_cam1_1.bmp 282_frontal.bmp 0.060122**

**451_cam1_1.bmp 559_frontal.bmp 0.055461**

**452_cam1_1.bmp 314_frontal.bmp 0.093286**

**453_cam1_1.bmp 056_frontal.bmp 0.102275**

**454_cam1_1.bmp 053_frontal.bmp 0.062582**

**455_cam1_1.bmp 455_frontal.bmp 0.063603**

**456_cam1_1.bmp 174_frontal.bmp 0.080657**

**457_cam1_1.bmp 211_frontal.bmp 0.033399**

**458_cam1_1.bmp 538_frontal.bmp 0.085867**

**459_cam1_1.bmp 151_frontal.bmp 0.074098**

**460_cam1_1.bmp 570_frontal.bmp 0.061793**

**461_cam1_1.bmp 313_frontal.bmp 0.051751**

**462_cam1_1.bmp 348_frontal.bmp 0.121637**

**463_cam1_1.bmp 624_frontal.bmp 0.046346**

**464_cam1_1.bmp 426_frontal.bmp 0.040965**

**465_cam1_1.bmp 350_frontal.bmp 0.055692**

**466_cam1_1.bmp 314_frontal.bmp 0.084170**

**467_cam1_1.bmp 253_frontal.bmp 0.176917**

**468_cam1_1.bmp 349_frontal.bmp 0.045971**

**469_cam1_1.bmp 310_frontal.bmp 0.089613**

**470_cam1_1.bmp 697_frontal.bmp 0.062926**

**471_cam1_1.bmp 598_frontal.bmp 0.114748**

**472_cam1_1.bmp 290_frontal.bmp 0.052371**

**473_cam1_1.bmp 155_frontal.bmp 0.096111**

**474_cam1_1.bmp 486_frontal.bmp 0.071283**

**475_cam1_1.bmp 560_frontal.bmp 0.047027**

**476_cam1_1.bmp 221_frontal.bmp 0.071144**

**477_cam1_1.bmp 284_frontal.bmp 0.278139**

**478_cam1_1.bmp 249_frontal.bmp 0.295015**

**479_cam1_1.bmp 317_frontal.bmp 0.041770**

**480_cam1_1.bmp 348_frontal.bmp 0.091290**

**481_cam1_1.bmp 323_frontal.bmp 0.043747**

**482_cam1_1.bmp 711_frontal.bmp 0.032850**

**483_cam1_1.bmp 624_frontal.bmp 0.075310**

**484_cam1_1.bmp 578_frontal.bmp 0.051174**

**485_cam1_1.bmp 262_frontal.bmp 0.044681**

**486_cam1_1.bmp 334_frontal.bmp 0.050784**

**487_cam1_1.bmp 711_frontal.bmp 0.068025**

**488_cam1_1.bmp 563_frontal.bmp 0.051389**

**489_cam1_1.bmp 489_frontal.bmp 0.048556**

**490_cam1_1.bmp 387_frontal.bmp 0.043902**

**491_cam1_1.bmp 334_frontal.bmp 0.061539**

**492_cam1_1.bmp 310_frontal.bmp 0.045829**

**493_cam1_1.bmp 670_frontal.bmp 0.075641**

**494_cam1_1.bmp 209_frontal.bmp 0.049094**

**495_cam1_1.bmp 174_frontal.bmp 0.040866**

**496_cam1_1.bmp 324_frontal.bmp 0.032680**

**497_cam1_1.bmp 349_frontal.bmp 0.042115**

**498_cam1_1.bmp 379_frontal.bmp 0.042867**

**499_cam1_1.bmp 348_frontal.bmp 0.058325**

**500_cam1_1.bmp 323_frontal.bmp 0.062290**

**501_cam1_1.bmp 310_frontal.bmp 0.061472**

**502_cam1_1.bmp 562_frontal.bmp 0.047497**

**503_cam1_1.bmp 224_frontal.bmp 0.055070**

**504_cam1_1.bmp 559_frontal.bmp 0.039547**

**505_cam1_1.bmp 320_frontal.bmp 0.038399**

**506_cam1_1.bmp 562_frontal.bmp 0.045940**

**507_cam1_1.bmp 281_frontal.bmp 0.139233**

**508_cam1_1.bmp 350_frontal.bmp 0.194884**

**509_cam1_1.bmp 155_frontal.bmp 0.046099**

**510_cam1_1.bmp 313_frontal.bmp 0.074579**

**511_cam1_1.bmp 511_frontal.bmp 0.069009**

**512_cam1_1.bmp 691_frontal.bmp 0.105346**

**513_cam1_1.bmp 561_frontal.bmp 0.041257**

**514_cam1_1.bmp 330_frontal.bmp 0.053925**

**515_cam1_1.bmp 253_frontal.bmp 0.146074**

**516_cam1_1.bmp 314_frontal.bmp 0.086412**

**517_cam1_1.bmp 281_frontal.bmp 0.240854**

**518_cam1_1.bmp 090_frontal.bmp 0.067226**

**519_cam1_1.bmp 519_frontal.bmp 0.068742**

**520_cam1_1.bmp 528_frontal.bmp 0.124149**

**521_cam1_1.bmp 521_frontal.bmp 0.098940**

**522_cam1_1.bmp 281_frontal.bmp 0.236040**

**523_cam1_1.bmp 523_frontal.bmp 0.051896**

**524_cam1_1.bmp 524_frontal.bmp 0.181326**

**525_cam1_1.bmp 566_frontal.bmp 0.044666**

**526_cam1_1.bmp 322_frontal.bmp 0.051202**

**527_cam1_1.bmp 527_frontal.bmp 0.058751**

**528_cam1_1.bmp 528_frontal.bmp 0.087353**

**529_cam1_1.bmp 529_frontal.bmp 0.096440**

**530_cam1_1.bmp 530_frontal.bmp 0.070305**

**531_cam1_1.bmp 531_frontal.bmp 0.078248**

**532_cam1_1.bmp 532_frontal.bmp 0.068973**

**533_cam1_1.bmp 533_frontal.bmp 0.186934**

**534_cam1_1.bmp 670_frontal.bmp 0.078326**

**535_cam1_1.bmp 566_frontal.bmp 0.071184**

**536_cam1_1.bmp 053_frontal.bmp 0.050988**

**537_cam1_1.bmp 691_frontal.bmp 0.089953**

**538_cam1_1.bmp 538_frontal.bmp 0.115288**

**539_cam1_1.bmp 415_frontal.bmp 0.086173**

**540_cam1_1.bmp 540_frontal.bmp 0.086088**

**541_cam1_1.bmp 541_frontal.bmp 0.072757**

**542_cam1_1.bmp 314_frontal.bmp 0.097514**

**543_cam1_1.bmp 543_frontal.bmp 0.076803**

**544_cam1_1.bmp 155_frontal.bmp 0.072664**

**545_cam1_1.bmp 722_frontal.bmp 0.038130**

**546_cam1_1.bmp 546_frontal.bmp 0.071877**

**547_cam1_1.bmp 320_frontal.bmp 0.082773**

**548_cam1_1.bmp 548_frontal.bmp 0.054905**

**549_cam1_1.bmp 549_frontal.bmp 0.052094**

**550_cam1_1.bmp 314_frontal.bmp 0.046253**

**551_cam1_1.bmp 056_frontal.bmp 0.056779**

**552_cam1_1.bmp 697_frontal.bmp 0.070908**

**553_cam1_1.bmp 365_frontal.bmp 0.043157**

**554_cam1_1.bmp 554_frontal.bmp 0.048000**

**555_cam1_1.bmp 555_frontal.bmp 0.092483**

**556_cam1_1.bmp 556_frontal.bmp 0.064726**

**557_cam1_1.bmp 172_frontal.bmp 0.059247**

**558_cam1_1.bmp 558_frontal.bmp 0.072472**

**559_cam1_1.bmp 559_frontal.bmp 0.056046**

**560_cam1_1.bmp 560_frontal.bmp 0.067813**

**561_cam1_1.bmp 561_frontal.bmp 0.054986**

**562_cam1_1.bmp 562_frontal.bmp 0.051650**

**563_cam1_1.bmp 563_frontal.bmp 0.059242**

**564_cam1_1.bmp 691_frontal.bmp 0.065887**

**565_cam1_1.bmp 565_frontal.bmp 0.098079**

**566_cam1_1.bmp 566_frontal.bmp 0.130461**

**567_cam1_1.bmp 567_frontal.bmp 0.075606**

**568_cam1_1.bmp 675_frontal.bmp 0.101143**

**569_cam1_1.bmp 691_frontal.bmp 0.080032**

**570_cam1_1.bmp 570_frontal.bmp 0.089386**

**571_cam1_1.bmp 568_frontal.bmp 0.072540**

**572_cam1_1.bmp 326_frontal.bmp 0.050297**

**573_cam1_1.bmp 573_frontal.bmp 0.149833**

**574_cam1_1.bmp 574_frontal.bmp 0.038450**

**575_cam1_1.bmp 575_frontal.bmp 0.149608**

**576_cam1_1.bmp 709_frontal.bmp 0.067344**

**577_cam1_1.bmp 577_frontal.bmp 0.133596**

**578_cam1_1.bmp 578_frontal.bmp 0.090374**

**579_cam1_1.bmp 566_frontal.bmp 0.101874**

**580_cam1_1.bmp 563_frontal.bmp 0.061081**

**581_cam1_1.bmp 581_frontal.bmp 0.148883**

**582_cam1_1.bmp 582_frontal.bmp 0.095814**

**583_cam1_1.bmp 583_frontal.bmp 0.110197**

**584_cam1_1.bmp 584_frontal.bmp 0.120370**

**585_cam1_1.bmp 585_frontal.bmp 0.076121**

**586_cam1_1.bmp 586_frontal.bmp 0.034744**

**587_cam1_1.bmp 587_frontal.bmp 0.125954**

**588_cam1_1.bmp 337_frontal.bmp 0.074958**

**589_cam1_1.bmp 324_frontal.bmp 0.058609**

**590_cam1_1.bmp 590_frontal.bmp 0.088849**

**591_cam1_1.bmp 691_frontal.bmp 0.138806**

**592_cam1_1.bmp 372_frontal.bmp 0.047187**

**593_cam1_1.bmp 359_frontal.bmp 0.058361**

**594_cam1_1.bmp 090_frontal.bmp 0.057556**

**595_cam1_1.bmp 595_frontal.bmp 0.053750**

**596_cam1_1.bmp 568_frontal.bmp 0.059935**

**597_cam1_1.bmp 240_frontal.bmp 0.065056**

**598_cam1_1.bmp 313_frontal.bmp 0.046762**

**599_cam1_1.bmp 134_frontal.bmp 0.050955**

**600_cam1_1.bmp 600_frontal.bmp 0.085076**

**601_cam1_1.bmp 585_frontal.bmp 0.075184**

**602_cam1_1.bmp 231_frontal.bmp 0.062534**

**603_cam1_1.bmp 415_frontal.bmp 0.069066**

**604_cam1_1.bmp 557_frontal.bmp 0.048900**

**605_cam1_1.bmp 691_frontal.bmp 0.081221**

**606_cam1_1.bmp 313_frontal.bmp 0.047893**

**607_cam1_1.bmp 326_frontal.bmp 0.042968**

**608_cam1_1.bmp 691_frontal.bmp 0.064635**

**609_cam1_1.bmp 174_frontal.bmp 0.063613**

**610_cam1_1.bmp 326_frontal.bmp 0.068373**

**611_cam1_1.bmp 691_frontal.bmp 0.042361**

**612_cam1_1.bmp 709_frontal.bmp 0.052086**

**613_cam1_1.bmp 313_frontal.bmp 0.052310**

**614_cam1_1.bmp 313_frontal.bmp 0.063639**

**615_cam1_1.bmp 615_frontal.bmp 0.079326**

**616_cam1_1.bmp 174_frontal.bmp 0.061065**

**617_cam1_1.bmp 339_frontal.bmp 0.047079**

**618_cam1_1.bmp 575_frontal.bmp 0.066030**

**619_cam1_1.bmp 601_frontal.bmp 0.051885**

**620_cam1_1.bmp 243_frontal.bmp 0.065806**

**621_cam1_1.bmp 691_frontal.bmp 0.058154**

**622_cam1_1.bmp 372_frontal.bmp 0.054303**

**623_cam1_1.bmp 313_frontal.bmp 0.040425**

**624_cam1_1.bmp 624_frontal.bmp 0.062598**

**625_cam1_1.bmp 124_frontal.bmp 0.047972**

**626_cam1_1.bmp 288_frontal.bmp 0.039132**

**627_cam1_1.bmp 310_frontal.bmp 0.066528**

**628_cam1_1.bmp 014_frontal.bmp 0.036603**

**629_cam1_1.bmp 151_frontal.bmp 0.047298**

**630_cam1_1.bmp 310_frontal.bmp 0.050955**

**631_cam1_1.bmp 481_frontal.bmp 0.040548**

**632_cam1_1.bmp 336_frontal.bmp 0.046412**

**633_cam1_1.bmp 313_frontal.bmp 0.059954**

**634_cam1_1.bmp 557_frontal.bmp 0.093868**

**635_cam1_1.bmp 104_frontal.bmp 0.048764**

**636_cam1_1.bmp 218_frontal.bmp 0.043755**

**637_cam1_1.bmp 439_frontal.bmp 0.046168**

**638_cam1_1.bmp 155_frontal.bmp 0.061578**

**639_cam1_1.bmp 460_frontal.bmp 0.056208**

**640_cam1_1.bmp 709_frontal.bmp 0.044964**

**641_cam1_1.bmp 337_frontal.bmp 0.059748**

**642_cam1_1.bmp 670_frontal.bmp 0.129445**

**643_cam1_1.bmp 691_frontal.bmp 0.059571**

**644_cam1_1.bmp 387_frontal.bmp 0.047239**

**645_cam1_1.bmp 645_frontal.bmp 0.051064**

**646_cam1_1.bmp 565_frontal.bmp 0.043295**

**647_cam1_1.bmp 359_frontal.bmp 0.050925**

**648_cam1_1.bmp 387_frontal.bmp 0.067745**

**649_cam1_1.bmp 712_frontal.bmp 0.052178**

**650_cam1_1.bmp 359_frontal.bmp 0.057144**

**651_cam1_1.bmp 126_frontal.bmp 0.056290**

**652_cam1_1.bmp 697_frontal.bmp 0.064523**

**653_cam1_1.bmp 691_frontal.bmp 0.074487**

**654_cam1_1.bmp 691_frontal.bmp 0.069812**

**655_cam1_1.bmp 691_frontal.bmp 0.049334**

**656_cam1_1.bmp 322_frontal.bmp 0.041132**

**657_cam1_1.bmp 598_frontal.bmp 0.051489**

**658_cam1_1.bmp 134_frontal.bmp 0.046543**

**659_cam1_1.bmp 359_frontal.bmp 0.053262**

**660_cam1_1.bmp 310_frontal.bmp 0.041797**

**661_cam1_1.bmp 334_frontal.bmp 0.036550**

**662_cam1_1.bmp 525_frontal.bmp 0.054263**

**663_cam1_1.bmp 353_frontal.bmp 0.051232**

**664_cam1_1.bmp 670_frontal.bmp 0.120179**

**665_cam1_1.bmp 211_frontal.bmp 0.055023**

**666_cam1_1.bmp 691_frontal.bmp 0.082827**

**667_cam1_1.bmp 246_frontal.bmp 0.094129**

**668_cam1_1.bmp 573_frontal.bmp 0.049193**

**669_cam1_1.bmp 359_frontal.bmp 0.058289**

**670_cam1_1.bmp 188_frontal.bmp 0.046043**

**671_cam1_1.bmp 568_frontal.bmp 0.118617**

**672_cam1_1.bmp 359_frontal.bmp 0.069504**

**673_cam1_1.bmp 344_frontal.bmp 0.048686**

**674_cam1_1.bmp 314_frontal.bmp 0.088089**

**675_cam1_1.bmp 243_frontal.bmp 0.068503**

**676_cam1_1.bmp 585_frontal.bmp 0.074596**

**677_cam1_1.bmp 691_frontal.bmp 0.063187**

**678_cam1_1.bmp 624_frontal.bmp 0.069350**

**679_cam1_1.bmp 565_frontal.bmp 0.040461**

**680_cam1_1.bmp 379_frontal.bmp 0.030648**

**681_cam1_1.bmp 313_frontal.bmp 0.090347**

**682_cam1_1.bmp 538_frontal.bmp 0.131955**

**683_cam1_1.bmp 707_frontal.bmp 0.067803**

**684_cam1_1.bmp 684_frontal.bmp 0.080383**

**685_cam1_1.bmp 155_frontal.bmp 0.099363**

**686_cam1_1.bmp 557_frontal.bmp 0.050263**

**687_cam1_1.bmp 155_frontal.bmp 0.085817**

**688_cam1_1.bmp 422_frontal.bmp 0.050294**

**689_cam1_1.bmp 211_frontal.bmp 0.181516**

**690_cam1_1.bmp 281_frontal.bmp 0.168044**

**691_cam1_1.bmp 691_frontal.bmp 0.104744**

**692_cam1_1.bmp 692_frontal.bmp 0.048555**

**693_cam1_1.bmp 693_frontal.bmp 0.069805**

**694_cam1_1.bmp 528_frontal.bmp 0.230669**

**695_cam1_1.bmp 213_frontal.bmp 0.124070**

**696_cam1_1.bmp 711_frontal.bmp 0.030423**

**697_cam1_1.bmp 284_frontal.bmp 0.281750**

**698_cam1_1.bmp 169_frontal.bmp 0.094305**

**699_cam1_1.bmp 699_frontal.bmp 0.119278**

**700_cam1_1.bmp 642_frontal.bmp 0.076565**

**701_cam1_1.bmp 244_frontal.bmp 0.178074**

**702_cam1_1.bmp 245_frontal.bmp 0.190238**

**703_cam1_1.bmp 528_frontal.bmp 0.080930**

**704_cam1_1.bmp 511_frontal.bmp 0.034778**

**705_cam1_1.bmp 433_frontal.bmp 0.169463**

**706_cam1_1.bmp 245_frontal.bmp 0.159357**

**707_cam1_1.bmp 584_frontal.bmp 0.061288**

**708_cam1_1.bmp 697_frontal.bmp 0.095730**

**709_cam1_1.bmp 670_frontal.bmp 0.064118**

**710_cam1_1.bmp 275_frontal.bmp 0.126184**

**711_cam1_1.bmp 691_frontal.bmp 0.100123**

**712_cam1_1.bmp 053_frontal.bmp 0.116117**

**713_cam1_1.bmp 713_frontal.bmp 0.173676**

**714_cam1_1.bmp 253_frontal.bmp 0.159523**

**715_cam1_1.bmp 736_frontal.bmp 0.100299**

**716_cam1_1.bmp 716_frontal.bmp 0.056052**

**717_cam1_1.bmp 691_frontal.bmp 0.077318**

**718_cam1_1.bmp 281_frontal.bmp 0.224712**

**719_cam1_1.bmp 284_frontal.bmp 0.176977**

**720_cam1_1.bmp 348_frontal.bmp 0.075204**

**721_cam1_1.bmp 273_frontal.bmp 0.217366**

**722_cam1_1.bmp 455_frontal.bmp 0.103539**

**723_cam1_1.bmp 250_frontal.bmp 0.102909**

**724_cam1_1.bmp 566_frontal.bmp 0.119031**

**725_cam1_1.bmp 725_frontal.bmp 0.064329**

**726_cam1_1.bmp 227_frontal.bmp 0.189848**

**727_cam1_1.bmp 544_frontal.bmp 0.098545**

**728_cam1_1.bmp 213_frontal.bmp 0.077190**

**729_cam1_1.bmp 253_frontal.bmp 0.095568**

**730_cam1_1.bmp 730_frontal.bmp 0.111148**

**731_cam1_1.bmp 731_frontal.bmp 0.048127**

**732_cam1_1.bmp 732_frontal.bmp 0.084985**

**733_cam1_1.bmp 245_frontal.bmp 0.154171**

**734_cam1_1.bmp 253_frontal.bmp 0.163025**

**735_cam1_1.bmp 227_frontal.bmp 0.217104**

**736_cam1_1.bmp 281_frontal.bmp 0.227625**

**737_cam1_1.bmp 737_frontal.bmp 0.035712**

**738_cam1_1.bmp 547_frontal.bmp 0.156547**

**739_cam1_1.bmp 739_frontal.bmp 0.095880**

**740_cam1_1.bmp 253_frontal.bmp 0.167174**
